# Genetic polymorphisms affecting telomere length and their association with cardiovascular disease in the Heinz-Nixdorf-Recall study

**DOI:** 10.1371/journal.pone.0303357

**Published:** 2024-05-14

**Authors:** Nico Tannemann, Raimund Erbel, Markus M. Nöthen, Karl-Heinz Jöckel, Sonali Pechlivanis

**Affiliations:** 1 Institute for Medical Informatics, Biometry and Epidemiology, University Hospital of Essen, University Duisburg-Essen, Essen, Germany; 2 Department of Genomics, Life & Brain Center, University of Bonn, Bonn, Germany; 3 Helmholtz Zentrum München, German Research Center for Environmental Health, Institute of Asthma and Allergy Prevention, Neuherberg, Germany; Obafemi Awolowo University, NIGERIA

## Abstract

Short telomeres are associated with cardiovascular disease (CVD). We aimed to investigate, if genetically determined telomere-length effects CVD-risk in the Heinz-Nixdorf-Recall study (HNRS) population. We selected 14 single-nucleotide polymorphisms (SNPs) associated with telomere-length (p<10^−8^) from the literature and after exclusion 9 SNPs were included in the analyses. Additionally, a genetic risk score (GRS) using these 9 SNPs was calculated. Incident CVD was defined as fatal and non-fatal myocardial infarction, stroke, and coronary death. We included 3874 HNRS participants with available genetic data and had no known history of CVD at baseline. Cox proportional-hazards regression was used to test the association between the SNPs/GRS and incident CVD-risk adjusting for common CVD risk-factors. The analyses were further stratified by CVD risk-factors. During follow-up (12.1±4.31 years), 466 participants experienced CVD-events. No association between SNPs/GRS and CVD was observed in the adjusted analyses. However, the GRS, rs10936599, rs2487999 and rs8105767 increase the CVD-risk in current smoker. Few SNPs (rs10936599, rs2487999, and rs7675998) showed an increased CVD-risk, whereas rs10936599, rs677228 and rs4387287 a decreased CVD-risk, in further strata. The results of our study suggest different effects of SNPs/GRS on CVD-risk depending on the CVD risk-factor strata, highlighting the importance of stratified analyses in CVD risk-factors.

## Introduction

In 2019, cardiovascular disease (CVD) was the global leading cause of death, with an estimated death of 18.6 million [1]. Ischemic heart disease, including acute and chronic coronary syndromes, and strokes contributed the largest proportion to cardiovascular deaths [1]. As widely known, there are multiple cardiovascular risk factors, namely smoking, age, male sex, high blood pressure, and metabolic factors. Additionally, some genetic risk factors also contribute to the risk of CVD [2].

An elevation of cardiovascular risk has been shown to be associated with short telomeres [3]. Telomeres can be elongated by a protein called telomerase, which is composed of telomerase reverse transcriptase (TERT) and the telomerase RNA component (TERC) [3]. The organization of telomeres is mainly accomplished by the shelterin complex, which consists of up to six proteins [4]. The shelterin complex is important for the protection of the telomeres and telomerase regulation [4].

Telomere length decreases with the number of cell divisions; therefore, it is shorter in older people. Many cardiovascular risk factors effect telomere length. Male sex, low physical activity, smoking, and psychological stress are associated with shorter telomere length [3]. An estimated 34–82% of interindividual variability in telomere length is caused by genetic determinations [5]. The regulation of telomere length is quite complex, most of the genes coding for telomere length are involved in cell cycle control or DNA reparation [6]. In previous studies, single nucleotide polymorphisms (SNPs) associated with telomere length have been identified on different chromosomes [7–11] and some of these SNPs showed an association with various diseases [7–11]. The usage of a genetic risk score (GRS) as a proxy for a polygenic trait, such as telomere length, is rather helpful to assess the cumulative effect of genetically determined telomere length on the disease outcome [12]. A main advantage of a GRS is the possibility to evaluate the overall risk of multiple SNPs with small individual effects on a disease. Furthermore, GRS have a higher accuracy of risk prediction [12].

As telomere length is important for senescence and cell cycle control, the association between telomere length and age-related diseases, especially CVD and cancer, have been observed in various studies [6, 7, 13]. Several SNPs associated with short telomeres have been shown to be associated with idiopathic pulmonary fibrosis [7], coronary artery disease [14] and an increased risk for CVD [7]. Additionally, short telomeres have been shown to be associated with diabetes mellitus type 2 [3] and premature myocardial infarction [15]. Moreover, genetically determined short telomeres have been shown to be associated with decreased risk of thyroid cancer and lymphoma [6], while a SNP on the *TERT* locus associated with long telomeres is associated with an increased risk of breast and ovarian cancer and with a decreased risk of prostate cancer [9].

Thus, the aim of this study is to investigate the effect of genetic determinants of telomere length on CVD risk using the data of the population-based Heinz-Nixdorf-Recall study (HNRS). Since, no studies have investigated the association of genetic determinants of telomere length in different strata of CVD risk factors, we further investigated the effect of genetic determinants of telomere length in different CVD risk factor strata.

## Materials and methods

### Study population

We used the data from the Heinz-Nixdorf-Recall study, which is described in detail elsewhere [16]. Briefly, 4814 participants aged 45–75 years, were randomly recruited from the registration lists of the German metropolitan cities Essen, Bochum, and Mülheim between December 2000 and August 2003 [16]. The first and second follow-up examinations took place after 5 and 10 years. We accessed the data on February 25, 2019.

For this study, we excluded 327 participants having prior CVD at baseline, namely history of prior myocardial infarction, bypass surgery and/or percutaneous coronary intervention [17]. The final data was made up of 3874 HNRS participants with complete CVD data and SNPs. The study has been approved by the ethical committee at the University Hospital Essen, Germany and is conducted in accordance with the principles expressed in the Declaration of Helsinki. The approval number is 99-69-1200. The study was certified and recertified according to DIN EN ISO 9001:2000/2008. All study participants gave their written informed consent. In order to access the data for the present study, the authors have obtained permission from the internal committee of the Heinz Nixdorf Recall study.

#### Cardiovascular disease

Medical reports concerning primary end-points were obtained from general practitioners, hospitals and nursing homes [18]. Primary end-points of the HNRS are nonfatal myocardial infarction and cardiac death [16]. Furthermore, death certificates and interviews with general practitioners, relatives and witness were collected [18] as well as hospital and nursing home records with ECGs, laboratory values and pathology reports [19]. In this study the outcome incident CVD was defined as fatal and nonfatal myocardial infarction, stroke and coronary death [18]. We included CVD events that occurred between the baseline and the beginning of this study in February 2019 (n = 466, 12.03%).

### Cardiovascular risk factors

Medical history regarding cardiovascular risk factors was documented at baseline. Smoking status has been assessed as current smoker, former smoker and never smoker [20]. The measurement of the body mass index (BMI) was calculated as weight divided by height square in kg/m². Furthermore, the waist circumference was measured in cm. Lipid profile was measured using the standardized enzymatic methods (ADVIA 1650, Siemens Medical Solutions, Erlangen, Germany), fasting serum glucose was measured enzymatically by the hexokinase method [21]. The resting blood pressure was measured thrice, with the participants seated by using an automated oscillometric blood pressure device. The mean of the second and third value was calculated and used in the study [22]. Diabetes was defined as having any of following 4 criteria: (1) participants reported a history of clinically diagnosed diabetes, (2) participants took glucose-lowering drugs, (3) participants had fasting glucose levels of greater than 125 mg/dL, or (4) participants had non-fasting glucose levels of 200 mg/dL or greater [21].

### Assessment of coronary artery calcification

An assessment of coronary artery calcification was done by non-enhanced electron-beam computed tomography (C-100 or C-150 scanner, GE Imatron, San Francisco, CA, USA) [16]. Layers of 3 mm thickness from the pulmonary bifurcation to the apex of the heart were acquired every 100 ms [23]. The prospective ECG-triggering was conducted at 80% of the RR-interval [23]. The computation of the CAC score was conducted using the methods of Agatston et al. by virtuoso workstation (Siemens Medical Solutions, Forchheim, Germany) [24].

### Genotyping

Lymphocyte DNA was separated and genotyped using the Illumina GWAS chips (Omni1-Quad, Omni1S, OmniExpressv1.0, HumanCoreExomev1.0, and HumanCoreExomev1.1) and the Metabochip [25, 26]. The data for each chip was imputed by IMPUTE v. 2.3.1. For the Metabochip 1000 Genomes Phase 1 release March 2012 was used and 1000 Genomes Phase 3 release October 2014 for the other chips. Before imputation, quality control was performed for each chip at the subject level and then on SNPs [25, 27, 28]. Participants with heterozygote frequency greater than 5 standard deviations above the mean, >5% genotype data and outliers identified by principal component analysis were excluded [29]. SNPs with a minor allele frequency (MAF) <1%, a missing genotype frequency >5% or violation of Hardy-Weinberg equilibrium (HWE) (p<10^−5^) were excluded.

### Genetic determinants of telomere length

For this study, we selected 14 SNPs associated with telomere length (p<10^−8^) which were identified in the previous genome wide association studies (GWAS) [7–11]. Further, missing call rate and the Hardy-Weinberg-Equilibrium (HWE) [30] for each SNP as well as the linkage disequilibrium (LD) between the SNPs on the same chromosome using Ldlink [31] was calculated. If the SNPs were in high LD (r^2^≥0.8 and D’ = 1) [32], only the SNP with the lowest missing call rate was included in the analyses, if there were SNPs with equal missing call rate, the one with more citation was chosen [33] (S1 Table). SNPs with missing call rate exceeding 5% and having MAF <1% were also excluded from the analyses. None of the SNPs violated the HWE. 25.48% of the participants had missing data for rs7726159 and hence was excluded from the further analyses. The SNPs rs10936599, rs12696304 and rs1317082 on chromosome 3 as well as rs9419958, rs9420907 and rs4387287 on chromosome 10 were in high LD (S3 Table), only rs10936599 and rs4387287 were included for further analyses. S1 Table shows the risk allele, minor allele, minor allele frequency (MAF) and the effect size of the decrease in telomere length per risk allele from the literature. At end, we used 9 SNPs for our analyses. Additionally, a genetic risk score (GRS) was constructed using 9 SNPs by multiplying the estimated effect of the SNP on telomere length (beta estimates for decreased telomere length in base pairs from the literature) with the number of risk alleles for each SNP and summing up the products. This value was then divided by the total number of SNPs. Missing values were imputed based on the sample allele frequency. The PLINK scoring routine (—score) was used to calculate the GRS [34]. Thereafter, we calculated the z-score to standardize the score by subtracting the mean and dividing by the standard deviation of the score [32] using the scale method in R [35].

### Statistical methods

The continuous data are presented as mean ± standard deviation or median (first quartile and third quartile) for substantially skewed data. Nominal scaled data are presented as frequency and percentage. Differences between the group of participants with and without CVD event are given as Wilcoxon test for continuous data and χ² test for count data.

Cox proportional hazard regression was used to investigate the association between the SNPs respectively and the GRS with CVD [36]. We first performed a crude analysis, and then a multivariate Cox model adjusting for the cardiovascular risk factors including age, sex, total cholesterol, HDL, LDL, triglycerides, diabetes, smoking, blood pressure, high sensitive CRP (hsCRP), BMI, waist circumference, and coronary calcification was performed. In addition, we stratified the analyses in CVD risk factors (age, sex, total cholesterol, HDL, LDL, triglycerides, diabetes, smoking, blood pressure, hsCRP, BMI, waist circumference) as mentioned above and further adjusting for other CVD risk factors and the coronary artery calcification score. For the CVD risk factors stratified analysis we did not adjust for the corresponding CVD risk factor. We used clinical cut off values for the stratification as shown in the supplementary material (S2 Table) [37–43].

We controlled for multiple testing at 5% for the association between 9 SNPs and respectively the GRS with CVD risk in the crude, adjusted, and 27 stratified models thus testing 290 statistical tests that translate into α_BF_ = 1.72×10^−4^ using the Bonferroni procedure.

The statistical analysis was performed using the R software [35] with the packages Survival [44, 45] and survminer [46].

## Results

### Descriptive statistics

After a mean follow-up of 12.1±4.31 years incident CVD was 466 (12.03%). Of them 155 (4.00%) had acute myocardial infarctions, 61 (1.57%) SHDs, 30 (0.77%) deadly CHD, 120 (3.10%) ischemic strokes, and 16 (0.41%) haemorrhagic strokes.

The study characteristics are shown in Table 1. The mean age of the cohort at baseline was 59.62±7.81 years and the proportion of female participants was 50.03% (n = 1938). Before standardization, the mean of the GRS was 45.73±9.18.

**Table 1:** Descriptive statistics of the study population.

	All (n = 3874)	CVD (n = 466, 12.03%)	No CVD (n = 3408, 87.97%)	p-Value
Age, years ^a^	59.62±7.81	63.98±7.57	59.02±7.65	<0.001
Males^c^	1936 (49.97)	319 (68.45)	1617 (47.45)	<0.001
BMI, kg/m^2^ ^b^	27.4 (24.8;30.3)	28.3 (26;30.9)	27.2 (24.7;30.2)	<0.001
Waist circumference, cm ^a^	94.41±13.31	98.24±12.27	93.89±13.37	<0.001
Systolic blood pressure, mmHg ^a^	133.25±20.82	140.48±23.41	132.26±20.25	<0.001
Diastolic blood pressure, mmHg ^a^	81.53±10.82	82.57±12.27	81.39±10.59	0.049
Ideal blood pressure ^c^	930 (24.01)	71 (15.24)	859 (25.21)	<0.001
Normal blood pressure ^c^	694 (17.91)	70 (15.02)	624 (18.31)	<0.001
Elevated normal blood pressure ^c^	723 (18.66)	92 (19.74)	631 (18.52)	<0.001
Hypertension ^c^	1516 (39.13)	232 (49.79)	1284 (37.68)	<0.001
Glucose, mg/dl ^b^	106 (98;115)	110 (102;124)	105 (98;114)	<0.001
HbA_1c_, % ^b^	5.4 (5.1;5.7)	5.5 (5.2;6.1)	5.4 (5.1;5.7)	<0.001
Diabetes ^c^	526 (13.58)	120 (25.76)	406 (11.92)	<0.001
hsCRP, mg/dl ^b^	0.15 (0.07;0.33)	0.2 (0.09;0.44)	0.15 (0.07;0.32)	<0.001
Total cholesterol, mg/dl ^a^	229.04±39.28	224.13±41.14	229.71±38.98	0.006
HDL, mg/dl ^b^	56 (46;69)	51 (41.25;62)	56 (46.75;69)	<0.001
LDL, mg/dl ^a^	145.02±36.31	141.77±36.26	145.47±36.3	0.039
Triglycerides, mg/dl ^b^	125 (90;178)	144.5 (105.25;198)	122 (88;176)	<0.001
CAC score ^b^	2.98 (0;5.14)	5.53 (3.68;6.93)	2.53 (0;4.78)	<0.001
Never smoker ^c^	1616 (41.71)	156 (33.48)	1460 (42.84)	<0.001
Former smoker ^c^	1340 (34.59)	189 (40.56)	1151 (33.77)	<0.001
Current smoker ^c^	912 (23.54)	120 (25.75)	792 (23.24)	<0.001
GRS ^a^	45.73±9.18	45.47±9.26	45.77±9.17	0.499

BMI: body-mass-index, CAC: coronary artery calcification, GRS: genetic risk score, HDL: high density lipoprotein, hsCRP: high sensitive c-reactive protein, LDL: low-density lipoprotein.

^a^ Data presented as mean±SD.

^b^ Data presented as median (Q1;Q3).

^c^ Data presented as n (%)

### Association analysis between SNPs and cardiovascular risk

**Fig 1:**
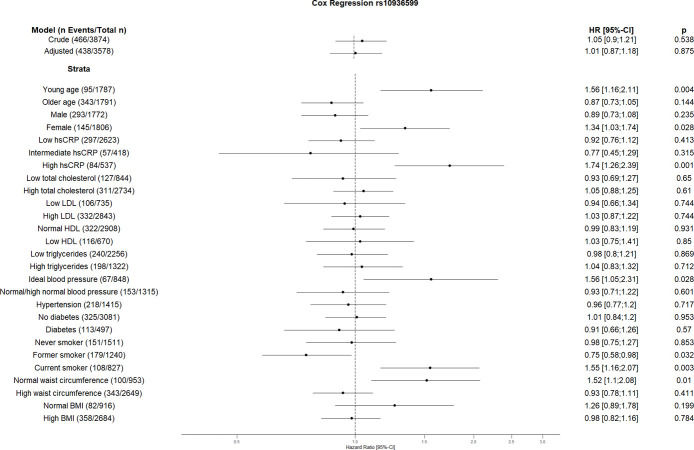
Effect of rs10936599 with incident cardiovascular disease. The results are presented as crude model as well as models adjusted for age, sex, total cholesterol, LDL cholesterol, HDL cholesterol, triglycerides, diabetes, smoking status (never, former, and current), systolic blood pressure, diastolic blood pressure, hsCRP, physical activity, waist circumference, BMI and coronary artery calcification. For the CVD risk factors stratified analyses, we did not adjust for the corresponding CVD risk factor. For the blood pressure stratified analysis, we excluded the variables systolic and diastolic blood pressure. Number of events/total Number given in parentheses.

**Fig 2:**
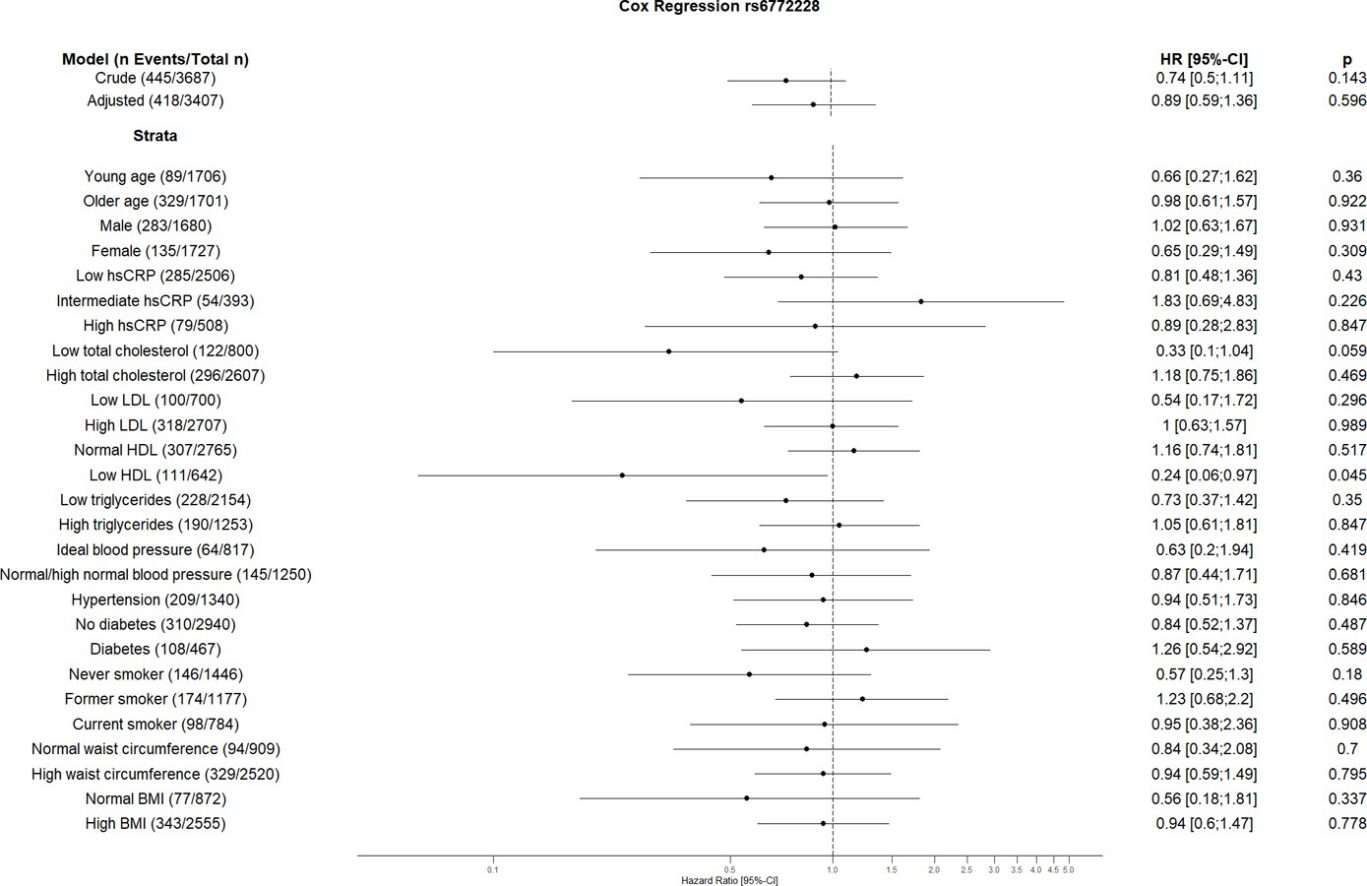
Effect of rs6772228 with incident cardiovascular disease. The results are presented as crude model as well as models adjusted for age, sex, total cholesterol, LDL cholesterol, HDL cholesterol, triglycerides, diabetes, smoking status (never, former, and current), systolic blood pressure, diastolic blood pressure, hsCRP, physical activity, waist circumference, BMI and coronary artery calcification. For the CVD risk factors stratified analyses, we did not adjust for the corresponding CVD risk factor. For the blood pressure stratified analysis, we excluded the variables systolic and diastolic blood pressure. Number of events/total Number given in parentheses.

**Fig 3:**
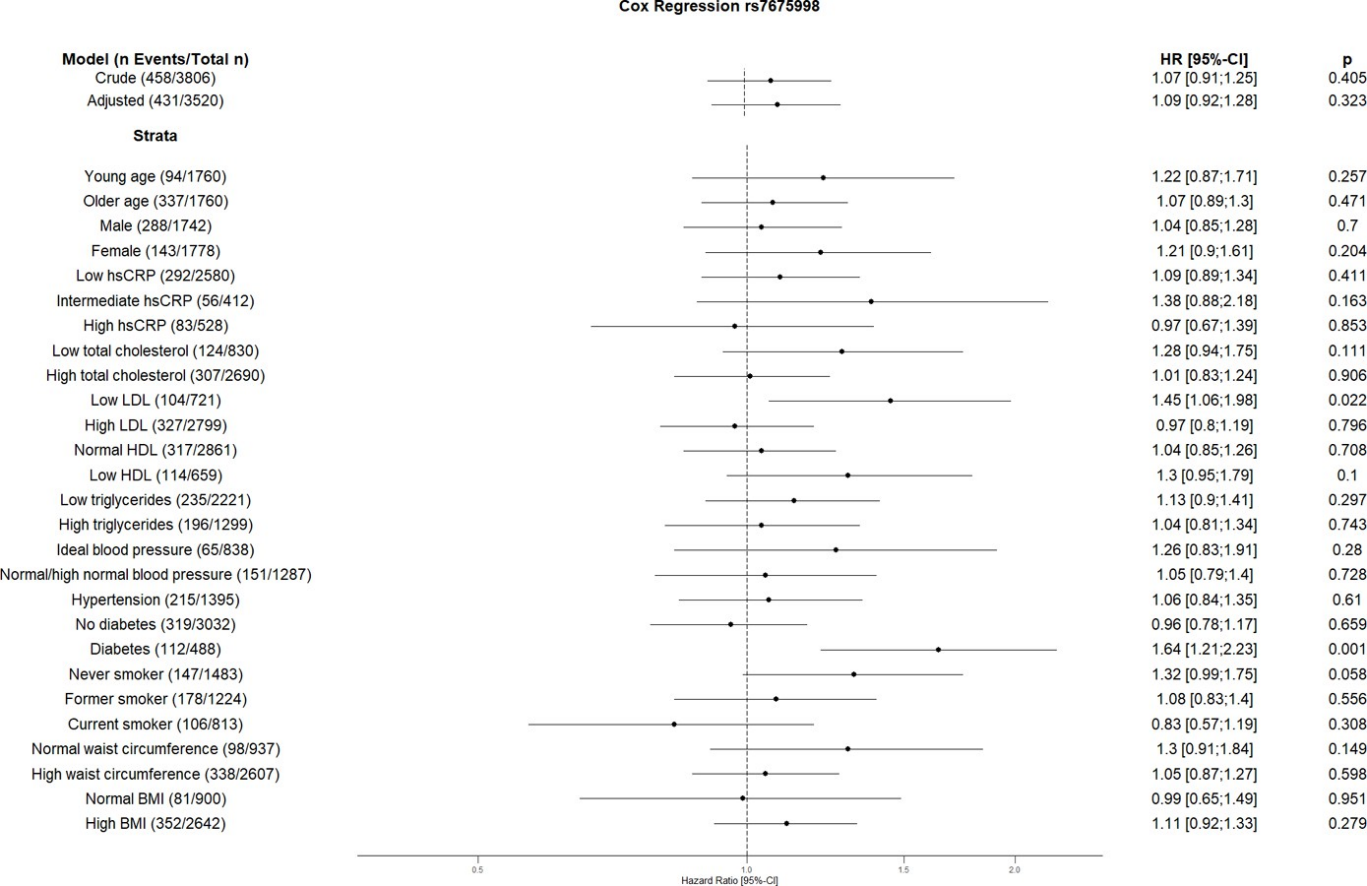
Effect of rs7675998 with incident cardiovascular disease. The results are presented as crude model as well as models adjusted for age, sex, total cholesterol, LDL cholesterol, HDL cholesterol, triglycerides, diabetes, smoking status (never, former, and current), systolic blood pressure, diastolic blood pressure, hsCRP, physical activity, waist circumference, BMI and coronary artery calcification. For the CVD risk factors stratified analyses, we did not adjust for the corresponding CVD risk factor. For the blood pressure stratified analysis, we excluded the variables systolic and diastolic blood pressure. Number of events/total Number given in parentheses.

**Fig 4:**
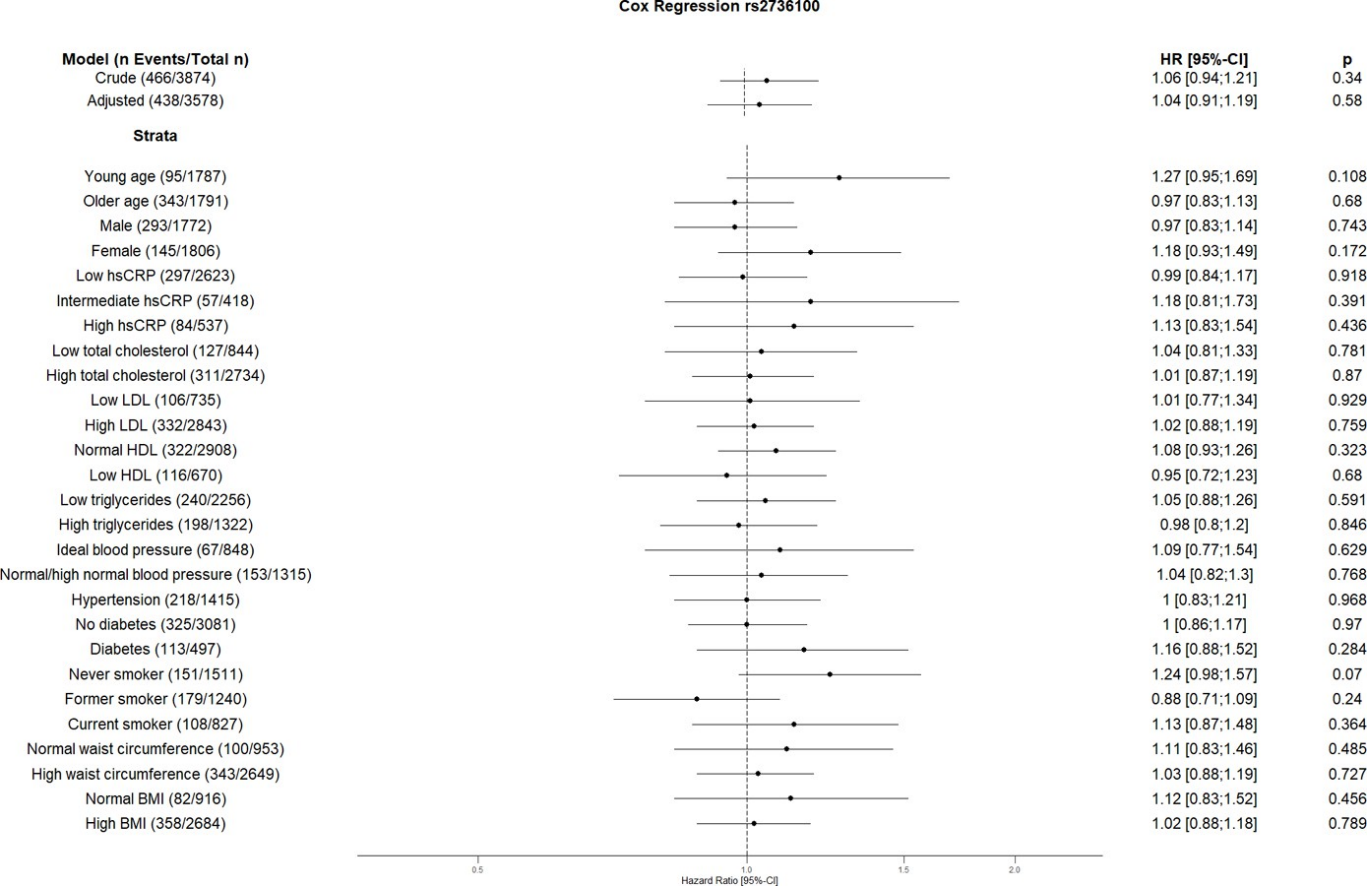
Effect of rs2736100 with incident cardiovascular disease. The results are presented as crude model as well as models adjusted for age, sex, total cholesterol, LDL cholesterol, HDL cholesterol, triglycerides, diabetes, smoking status (never, former, and current), systolic blood pressure, diastolic blood pressure, hsCRP, physical activity, waist circumference, BMI and coronary artery calcification. For the CVD risk factors stratified analyses, we did not adjust for the corresponding CVD risk factor. For the blood pressure stratified analysis, we excluded the variables systolic and diastolic blood pressure. Number of events/total Number given in parentheses.

**Fig 5:**
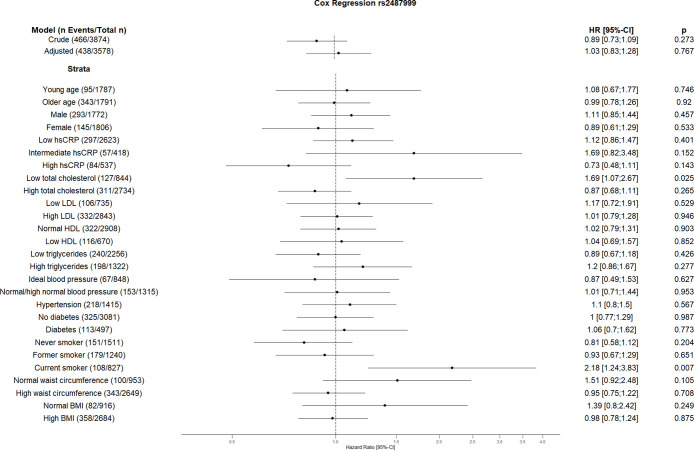
Effect of rs2487999 with incident cardiovascular disease. The results are presented as crude model as well as models adjusted for age, sex, total cholesterol, LDL cholesterol, HDL cholesterol, triglycerides, diabetes, smoking status (never, former, and current), systolic blood pressure, diastolic blood pressure, hsCRP, physical activity, waist circumference, BMI and coronary artery calcification. For the CVD risk factors stratified analyses, we did not adjust for the corresponding CVD risk factor. For the blood pressure stratified analysis, we excluded the variables systolic and diastolic blood pressure. Number of events/total Number given in parentheses.

**Fig 6:**
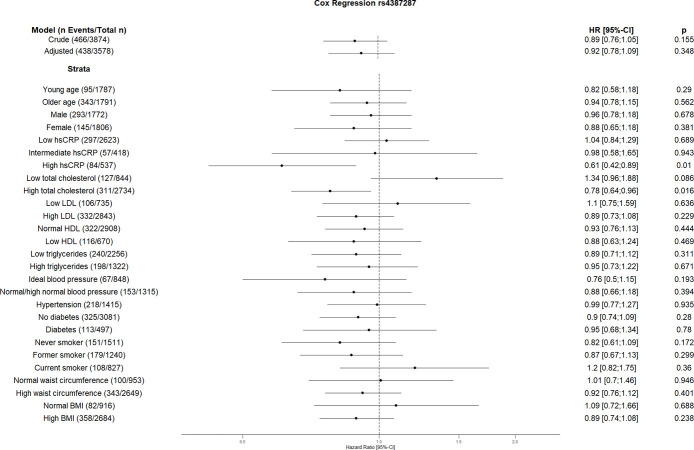
Effect of rs4387287 with incident cardiovascular disease. The results are presented as crude model as well as models adjusted for age, sex, total cholesterol, LDL cholesterol, HDL cholesterol, triglycerides, diabetes, smoking status (never, former, and current), systolic blood pressure, diastolic blood pressure, hsCRP, physical activity, waist circumference, BMI and coronary artery calcification. For the CVD risk factors stratified analyses, we did not adjust for the corresponding CVD risk factor. For the blood pressure stratified analysis, we excluded the variables systolic and diastolic blood pressure. Number of events/total Number given in parentheses.

**Fig 7:**
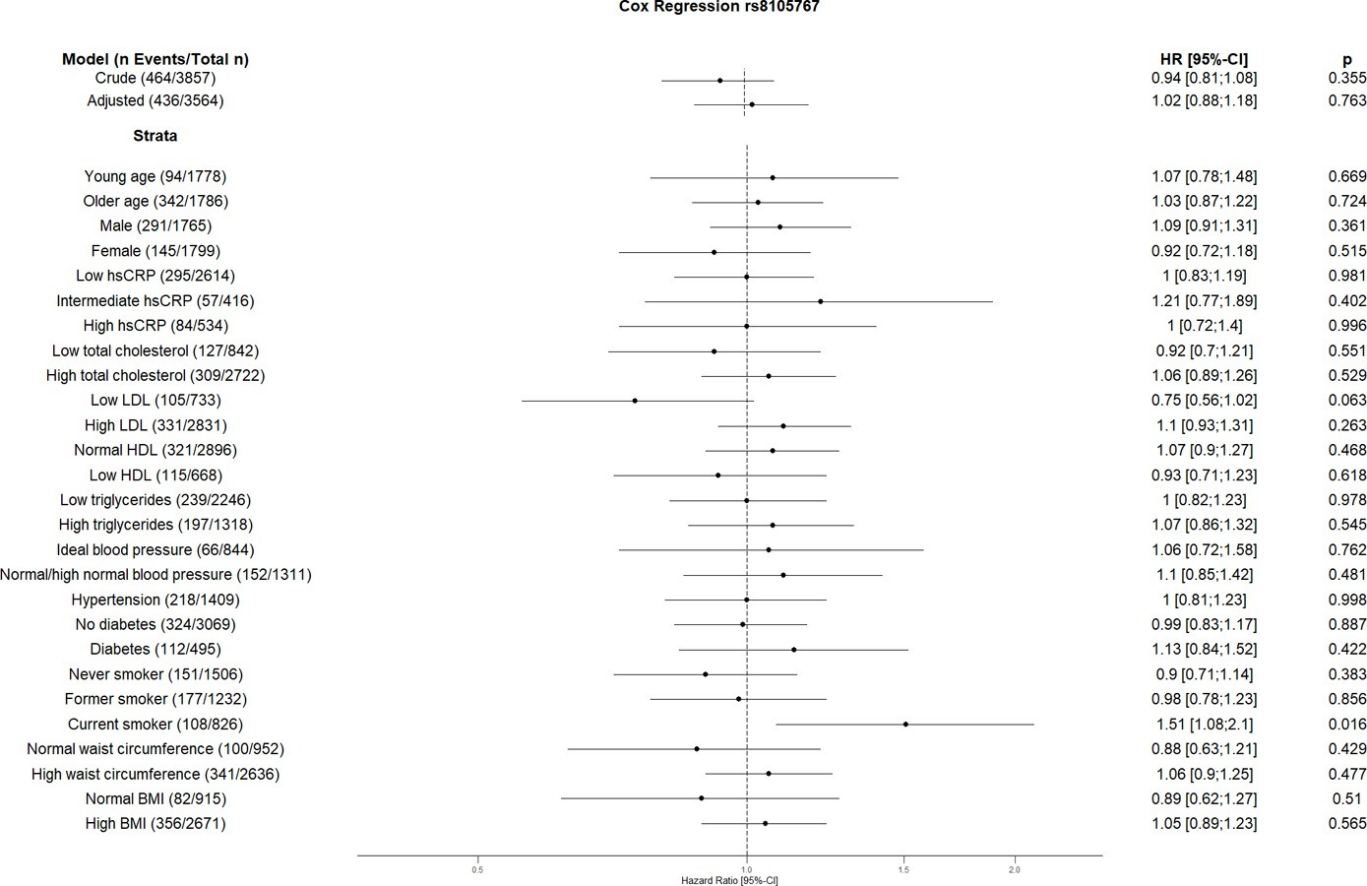
Effect of rs8105767 with incident cardiovascular disease. The results are presented as crude model as well as models adjusted for age, sex, total cholesterol, LDL cholesterol, HDL cholesterol, triglycerides, diabetes, smoking status (never, former, and current), systolic blood pressure, diastolic blood pressure, hsCRP, physical activity, waist circumference, BMI and coronary artery calcification. For the CVD risk factors stratified analyses, we did not adjust for the corresponding CVD risk factor. For the blood pressure stratified analysis, we excluded the variables systolic and diastolic blood pressure. Number of events/total Number given in parentheses.

**Fig 8:**
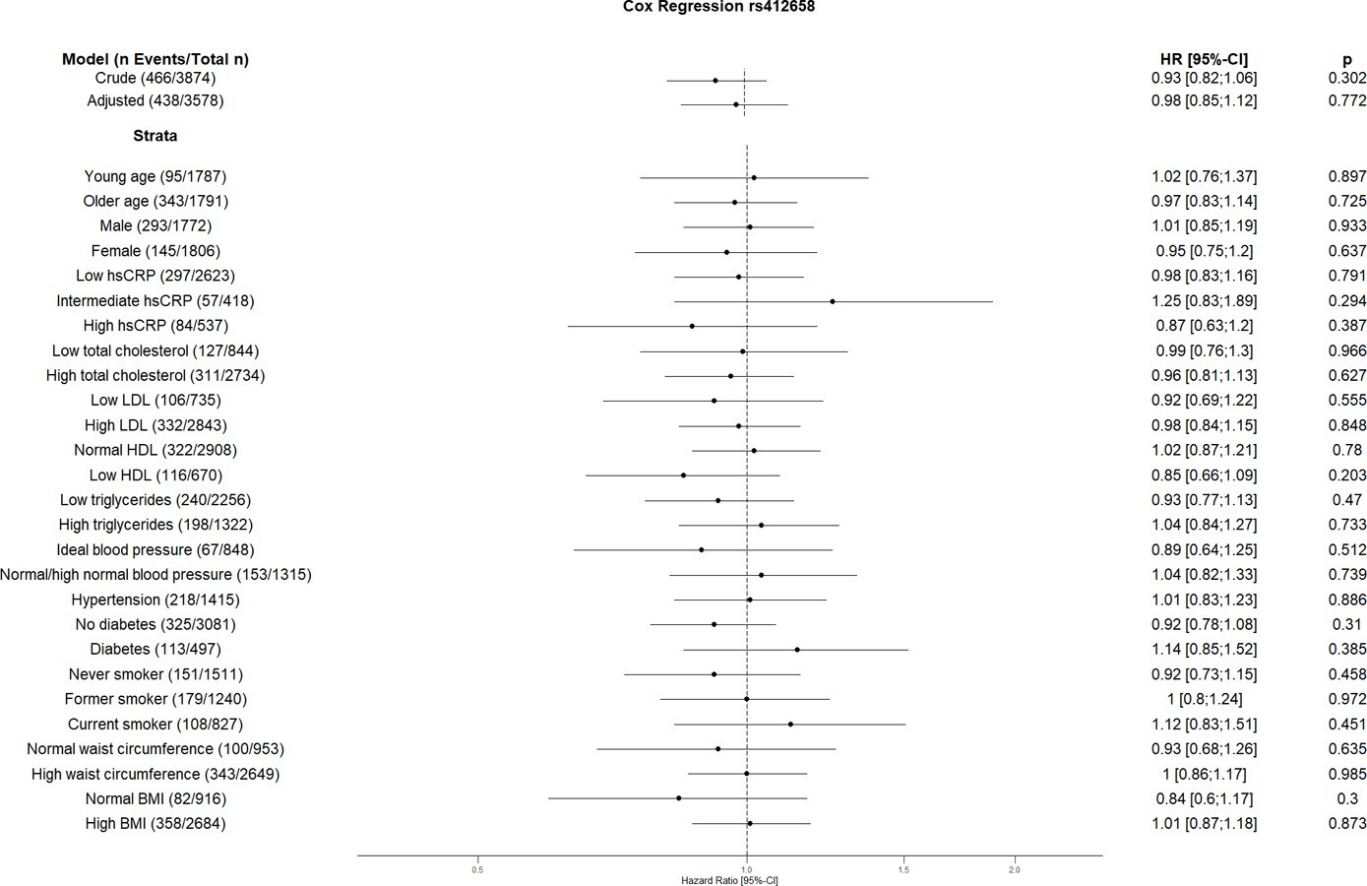
Effect of rs412658 with incident cardiovascular disease. The results are presented as crude model as well as models adjusted for age, sex, total cholesterol, LDL cholesterol, HDL cholesterol, triglycerides, diabetes, smoking status (never, former, and current), systolic blood pressure, diastolic blood pressure, hsCRP, physical activity, waist circumference, BMI and coronary artery calcification. For the CVD risk factors stratified analyses, we did not adjust for the corresponding CVD risk factor. For the blood pressure stratified analysis, we excluded the variables systolic and diastolic blood pressure. Number of events/total Number given in parentheses.

**Fig 9:**
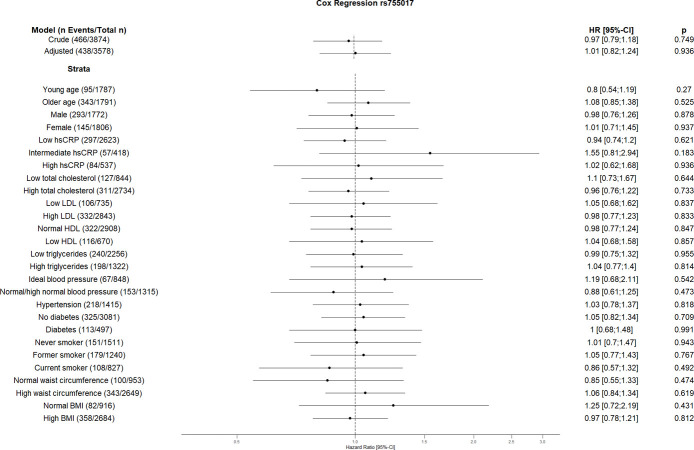
Effect of rs755017 with incident cardiovascular disease. The results are presented as crude model as well as models adjusted for age, sex, total cholesterol, LDL cholesterol, HDL cholesterol, triglycerides, diabetes, smoking status (never, former, and current), systolic blood pressure, diastolic blood pressure, hsCRP, physical activity, waist circumference, BMI and coronary artery calcification. For the CVD risk factors stratified analyses, we did not adjust for the corresponding CVD risk factor. For the blood pressure stratified analysis, we excluded the variables systolic and diastolic blood pressure. Number of events/total Number given in parentheses.

**Fig 10:**
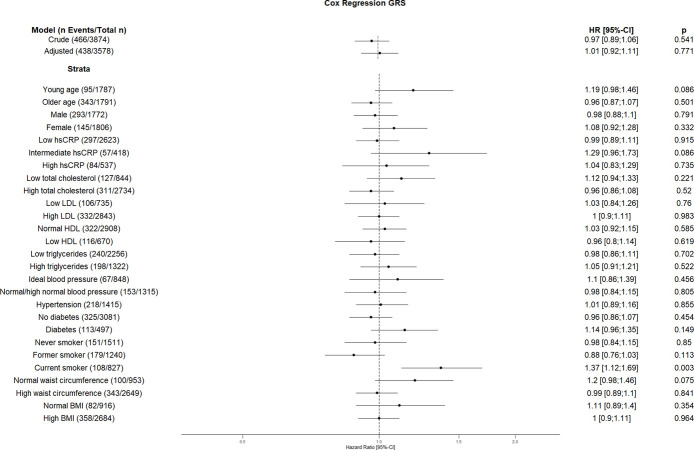
Effect of the genetic risk score with incident cardiovascular disease. The results are presented as crude model as well as models adjusted for age, sex, total cholesterol, LDL cholesterol, HDL cholesterol, triglycerides, diabetes, smoking status (never, former, and current), systolic blood pressure, diastolic blood pressure, hsCRP, physical activity, waist circumference, BMI and coronary artery calcification. For the CVD risk factors stratified analyses, we did not adjust for the corresponding CVD risk factor. For the blood pressure stratified analysis, we excluded the variables systolic and diastolic blood pressure. Number of events/total Number given in parentheses.

The crude Cox regression and CVD risk factors (age, sex, hsCRP, total cholesterol, HDL, LDL, triglycerides, blood pressure, diabetes, smoking, BMI, waist circumference, hsCRP and coronary artery calcification) adjusted model did not show any association between individual SNPs and CVD. However, the CVD risk factors stratified analyses suggest an association between some SNPs and incident CVD (Figs 1–10). The risk allele of rs10936599 associated with shorter telomeres located on chromosome 3 increased the risk of incident CVD risk in younger participants (HR [95%Confident interval (95%CI)]: 1.56 [1.16;2.11]), female (1.34 [1.03;1.74]), hsCRP >0.5 mg/dl (1.74 [1.26;2.39]), ideal blood pressure (1.56 [1.05;2.31]), current smokers (1.55 [1.16;2.07]) and normal waist circumference (1.52 [1.1;2.08]). Whereas the risk is reduced in former smokers (HR = 0.75 [0.58;0.98]). The risk allele of another SNP rs6772228 located on chromosome 3, showed protective effect on CVD in participants with low HDL (0.24 [0.06;0.97]). The only included SNP from chromosome 4, rs7675998 increased the CVD risk (1.64 [1.21;2.23]) in diabetes and low LDL (1.45 [1.06;1.98]) stratum. No association was observed for the SNPs on chromosome 5.

The risk allele of rs2487999 located at the *OBFC1* on chromosome 10 was associated with CVD risk in the stratum low total cholesterol (1.69 [1.07;2.67]) and current smokers (2.18 [1.24;3.83]). A risk reduction due to risk allele of rs4387287 located at chromosome 10 was observed in high total cholesterol (0.78 [0.64;0.96]) and CRP >0.5 mg/dl (0.61 [0.42;0.89]) stratum. SNP rs8105767 located at chromosome 19 increases the CVD risk for current smokers (1.51 [1.08;2.1]). The GRS showed increased risk of CVD only in current smokers (1.37 [1.12;1.69]).

We could not see any association of rs2736100 at the *TERT* locus on chromosome 5 with CVD risk in any strata, the same is true for rs4126585 at *ZNF676* on chromosome 19 and rs755017 at *RTEL1* on chromosome 20.

## Discussion and conclusions

In the present study, although we did not see any significant association between telomere associated single SNPs and the GRS with incident CVD in the crude and adjusted analysis, however, few SNPs and respectively the GRS showed nominally significant association with incident CVD risk in certain CVD risk factors strata. None of these findings were robust after multiple testing. Since none of the SNPs has direct impact on the CVD risk in the overall population and the results do not withstand multiple testing, these findings in numerous subgroups must be interpreted cautiously. However, we would like to emphasize that our findings could be biologically plausible.

The results of our study showed increased risk of the GRS on CVD in current smokers. Furthermore, three individual SNPs (rs10936599, rs2487999, and rs8105767) showed similar increased risk on CVD in this stratum. According to various studies, smoking is associated with short telomeres [47, 48]. This effect seems to be associated with the number of smoked cigarettes [48]. Shorter telomeres in smokers can possibly be explained by increased rate of telomere attrition because of oxidative stress caused by smoking [49]. Moreover, it could be hypothesized that additional telomere attrition due to smoking in people with genetically determined short telomeres might increase the CVD risk. Although unexpected, rs10936599 acts as a protective factor for CVD in former smokers. The effects of smoking cessation on telomeric biology are quite complex and are not yet completely understood [50]. Furthermore, rs10936599 showed higher risk in the stratum with hsCRP ≥0.5 mg/dl. Since high hsCRP levels are associated with short telomeres [51], one can assume an additional effect of rs10936599 on CVD. Similar to Brouilette et al. we showed rs10936599 increases the CVD risk in younger participants [15], as well as in some other CVD low risk factor strata like female, ideal blood pressure and normal waist circumference. Another SNP reducing the cardiovascular risk in this study is rs6772228 in group with low HDL levels. Besides, rs7675998 increases the CVD risk in group with low LDL as well as in participants with diabetes which has been shown to affect telomere length [52]. Ding et al. also showed the association of rs7675998 with CVD [53]. While rs2487999 increases the CVD risk in current smokers, as mentioned above, and stratum with low total cholesterol, rs4387287 decreases the risk in stratum high total cholesterol and hsCRP above or equal 0.5 mg/dl. The risk allele of rs4387287 has been shown by Surendran et al. to reduce the blood pressure [54]. Similarly, one could speculate similar risk reduction of rs4387287 in stratum associated with high CVD risk factor i.e. high total cholesterol and high hsCRP. High blood pressure is associated with high CRP levels [55]. This association seems to be bidirectional. On one hand, hypertension causes cellular stress and from there inflammation, which leads to an elevation of CRP levels. High CRP levels and systemic inflammation were shown to increase the hypertension risk, especially by increasing vascular stiffness due to molecular pathways [55]. Further, high total cholesterol is associated with hypertension as well [56]. Moreover, Chen et al. found that the effects of total cholesterol on systolic blood pressure are driven by its effects on vascular stiffness [57] Similar to the studies by Codd et al. and Ding et al. we did not see the association between rs8105767 and CVD risk in our study [7, 53], however, we found that rs8105767 risk allele is associated with CVD in current smokers.

Although we did not find an association of individual SNPs/GRS with CVD but an association of several SNPs and GRS with incident CVD in CVD risk factor strata was observed. Consistent with our findings Codd et al. [7] also did not find any significant association between single SNPs and CVD risk, however, they showed an association between a GRS and CVD risk.

Telomere length is not only associated with CVD, but several other age-related diseases, particularly with different cancer entities [6]. A genetic score for short telomeres, constructed using few of the SNPs used in our study, was shown to decrease the risk of breast cancer [58]. Zhang et al. [13] found an association of a GRS for long telomeres with lung adenocarcinoma. This GRS also included few of the SNPs from our study. Since long telomeres could cause a higher number of cell cycles in the life span of a cell, the chances of cancerogenic mutations and their accumulation might be increased [13]. On the other hand, short telomeres could cause genome instability and increase cancer risk [59]. A meta-analysis indicates an increased risk of urogenital cancers, smoking-related cancers, and cancers in the gastrointestinal system [59]. Cancer and CVD share many risk factors (e.g. diabetes, obesity, low physical activity) [60]. Moreover, CVD and cancer are both highly associated with systemic inflammation and oxidative stress [60]. Smoking is a risk factor for CVD and cancer as well as it can cause inflammation and oxidative stress [61]. It becomes clear, that the development of CVD and cancer, but also the mechanisms which lead to a higher CVD and cancer risk in smokers, have a lot in common and have all certain intersections with telomeric biology. Therefore, a similar effect of genetically determined telomere length on CVD and cancer, especially in risk strata linked to systemic inflammation and oxidative stress, such as smoking, seems plausible.

To ensure the quality of our genetic data, we compared the minor allele frequencies of the SNPs from our study to those of the European population in the 1000 Genomes Project [62] and did not find any major differences (https://www.ncbi.nlm.nih.gov/snp/) [63].

The strength of the present study is the large number of participants recruited from the Ruhr area in Germany and the availability of cardiovascular risk factor data, which made the stratified analysis possible, which is a main strength of this study. The clearly predefined end-point criteria and the existence of the external end-point committee are additional strengths of this study. As already mentioned, the development of CVD is a long-term process as well as cardiovascular risk-factors need time to have an effect [64]. Therefore, the long follow-up time of 12.1±4.31 years is a key strength of this study. Furthermore, using survival analysis gave us the chance to address censorship. The study has its limitations. The SNPs included in the present study have been shown to be associated with telomere length in previous studies, however, we did not measure the telomere length in our study participants. Hence, we could not look at the association between SNPs and telomere length in our study. Therefore, conclusions drawn on association between SNPs associated with short telomeres and CVD risk in this study are based on previously published data [7–11] and have to be verified in other studies. The excessive testing in numerous subgroups leading to potentially spurious results, is another limitation of the study. Nonetheless, it points out, which subgroups could be of importance for further stratified analysis in other larger studies.

In conclusion, we highlight the importance of stratified analysis to assess the association between genetic determinants of telomere length and risk for CVD. As there are many different, partly contradictory, or surprising findings in the literature due to the complex relation between genetic and telomeric biology as well as between telomere length and CVD, this study could encourage further investigation in more specific subgroups.

## Supporting information

S1 Table Summary statistics of the SNPs association with the short telomere length from the literature. β: base pair decrease in telomere length by risk allele, Chr: chromosome, HWE: Hardy-Weinberg equilibrium, MAF: minor allele frequency, SNP: single nucleotide polymorphism. *SNPs used in the study.
(DOCX)

S2 Table Cut-off values for stratification. (DOCX)

S3 Table Linkage disequilibrium of SNPs on chromosome 3 (a), chromosome 5 (b), chromosome 10 (c) and chromosome 19 (d). Linkage disequilibrium for the SNPs on the corresponding chromosome portrayed as D`/R2 Marked values: D`= 1.0 und R2>0.8.
(DOCX)

S4 Table Results of Cox regression for SNPs. coef: coefficient, HR: Hazard Ratio, se: Standard error, lower/upper: lower/upper boundarie of the 95% confidence interval. a) crude, b) adjusted, c1) young age, c2) older age, d1) male, d2) female, e1) low hsCRP, e2) intermediate hsCRP, e3) high hsCRP, f1) low total cholesterol, f2) high total cholesterol, g1) low LDL, g2) high LDL, h1) normal HDL, h2) high HDL, i1) low triglycerides, i2) high triglycerides, j1) ideal blood pressure, j2) normal/high normal blood pressure, j3) hypertension, k1) no diabetes, k2) diabetes, l1) never smoker, l2) former smoker, l3) current smoker, m1) normal waist circumference, m2) high waist circumference, n1) normal BMI, n2) high BMI.
(PDF)

S1 File Formulas as used in R. (DOCX)
