# Supplementary material for: Genetic polymorphisms affecting telomere length and their association with cardiovascular disease in the Heinz-Nixdorf-Recall study
Source: PLoS One. 2024 May 14;19(5):e0303357. doi: 10.1371/journal.pone.0303357 (PMC11093374; doi:10.1371/journal.pone.0303357)
Supplement: S1 Table — β: base pair decrease in telomere length by risk allele, Chr: chromosome, HWE: Hardy-Weinberg equilibrium, MAF: minor allele frequency, SNP: single nucleotide polymorphism. *SNPs used in the study. (DOCX) [file pone.0303357.s001.docx]

**S1 Table: Summary statistics of the SNPs association with the short telomere length from the literature.**

|  |  |  |  | Association between *SNP*  and telomere length | | | |  | Statistics | | |
| --- | --- | --- | --- | --- | --- | --- | --- | --- | --- | --- | --- |
| Chr | Position | rsID | Gene | Risk  allele | Minor  allele | β | p-value |  | MAF | Missing  (%) | p-value  HWE |
| 3 | 169492101 | rs10936599 [1] * | *TERC* | T | T | 117.3 | 2.5×10^-31^ |  | 0.250 | 0 | 0.669 |
| 3 | 169481271 | rs12696304 [2] | *TERC* | G | G | 75.0 | 3.7×10^-14^ |  | 0.276 | 0.05 | 0.172 |
| 3 | 58376019 | rs6772228 [3] * | *PXK* | A | A | 120.0 | 3.9×10^-10^ |  | 0.038 | 4.83 | 0.490 |
| 3 | 16949758 | rs1317082 [3] | *MYNN* | G | G | 77.0 | 3.0×10^-19^ |  | 0.250 | 0 | 0.732 |
| 4 | 164007820 | rs7675998 [1] * | *NAF1* | A | A | 89.7 | 4.3×10^-16^ |  | 0.207 | 1.76 | 0.767 |
| 5 | 1286516 | rs2736100 [1] * | *TERT* | A | C | 94.2 | 4.4×10^-19^ |  | 0.495 | 0 | 0.822 |
| 5 | 1282319 | rs7726159 [3] | *TERT* | C | A | 73.0 | 4.7×10^-17^ |  | 0.323 | 25.48 | 0.496 |
| 10 | 105677897 | rs2487999 [3] * | *OBFC1* | C | T | 100.0 | 4.2×10^-14^ |  | 0.105 | 0 | 0.733 |
| 10 | 105676465 | rs4387287 [4] * | *OBFC1* | C | A | 100.0 | 2.3×10^-11^ |  | 0.176 | 0 | 0.471 |
| 10 | 105675946 | rs9420907 [1] | *OBFC1* | A | C | 82.8 | 6.9×10^-11^ |  | 0.146 | 0.26 | 0.245 |
| 10 | 105659826 | rs9419958 [5] | *OBFC1* | C | T | 82.9 | 9.1×10^-11^ |  | 0.146 | 0.18 | 0.246 |
| 19 | 22215441 | rs8105767 [1] * | *ZNF208* | A | G | 58.0 | 1.1×10^-09^ |  | 0.279 | 0.44 | 0.262 |
| 19 | 22359440 | rs412658 [5] * | *ZNF676* | C | T | 49.7 | 9.8×10^-09^ |  | 0.354 | 0 | 0.106 |
| 20 | 62421622 | rs755017 [1] * | *RTEL1* | A | G | 74.1 | 6.7×10^-09^ |  | 0.113 | 0 | 0.229 |

1. Codd V, Nelson CP, Albrecht E, Mangino M, Deelen J, Buxton JL, et al. Identification of seven loci affecting mean telomere length and their association with disease. Nat Genet. 2013;45(4):422-7, 7e1-2.

2. Codd V, Mangino M, van der Harst P, Braund PS, Kaiser M, Beveridge AJ, et al. Common variants near TERC are associated with mean telomere length. Nat Genet. 2010;42(3):197-9.

3. Pooley KA, Bojesen SE, Weischer M, Nielsen SF, Thompson D, Amin Al Olama A, et al. A genome-wide association scan (GWAS) for mean telomere length within the COGS project: identified loci show little association with hormone-related cancer risk. Hum Mol Genet. 2013;22(24):5056-64.

4. Levy D, Neuhausen SL, Hunt SC, Kimura M, Hwang SJ, Chen W, et al. Genome-wide association identifies OBFC1 as a locus involved in human leukocyte telomere biology. Proc Natl Acad Sci U S A. 2010;107(20):9293-8.

5. Mangino M, Hwang SJ, Spector TD, Hunt SC, Kimura M, Fitzpatrick AL, et al. Genome-wide meta-analysis points to CTC1 and ZNF676 as genes regulating telomere homeostasis in humans. Hum Mol Genet. 2012;21(24):5385-94.
