# Supplementary material for: Genetic polymorphisms affecting telomere length and their association with cardiovascular disease in the Heinz-Nixdorf-Recall study
Source: PLoS One. 2024 May 14;19(5):e0303357. doi: 10.1371/journal.pone.0303357 (PMC11093374; doi:10.1371/journal.pone.0303357)
Supplement: S2 Table — (DOCX) [file pone.0303357.s002.docx]

**S2 Table: Cut-off values for stratification**

| Riskfactor | Strata |
| --- | --- |
| Age [1] | female: <65 vs. ≥65 years; male: <55 vs. ≥55 years |
| Sex | female; male |
| hsCRP [2] | ≤0.3 mg/dl; >0.3 -0.5 mg/dl; >0.5 mg/dl |
| Total cholesterol [3] | ≤200 mg/dl; >200mg/dl |
| HDL [4] | female: ≥50 mg/dl vs. <50mg/dl; male ≥40 mg/dl vs <40 mg/dl |
| LDL [5] | ≤115 mg/dl; >115 mg/dl |
| Triglycerides [5] | <150 mg/dl; ≥150 mg/dl |
| Blood pressure [6] | ideal; normal/high normal; hypertension |
| Diabetes | no; yes |
| Smoking | never smoker; former smoker; current smoker |
| Taillenumfang [7] | female: <80 cm vs. ≥80 cm; male: <94 cm vs. ≥94cm |
| BMI [7] | <25 kg/m^2^; ≥25 kg/m² |
| **BMI:** Body-Mass-Index, **HDL:** High density lipoproteine,  **hsCRP:** highsensitive c-reactive proteine, **LDL:** Low density lipoproteine | |

1. Nasir K, Budoff MJ, Wong ND, Scheuner M, Herrington D, Arnett DK, et al. Family History of Premature Coronary Heart Disease and Coronary Artery Calcification. Circulation. 2007;116(6):619-26.

2. Ridker PM. C-reactive protein, inflammation, and cardiovascular disease: clinical update. Tex Heart Inst J. 2005;32(3):384-6.

3. Mozaffarian D, Benjamin EJ, Go AS, Arnett DK, Blaha MJ, Cushman M, et al. Heart Disease and Stroke Statistics&#x2014;2016 Update. Circulation. 2016;133(4):e38-e360.

4. Grundy SM, Brewer HB, Cleeman JI, Smith SC, Lenfant C. Definition of Metabolic Syndrome. Circulation. 2004;109(3):433-8.

5. Mach F, Baigent C, Catapano AL, Koskinas KC, Casula M, Badimon L, et al. 2019 ESC/EAS Guidelines for the management of dyslipidaemias: lipid modification to reduce cardiovascular risk: The Task Force for the management of dyslipidaemias of the European Society of Cardiology (ESC) and European Atherosclerosis Society (EAS). European Heart Journal. 2020;41(1):111-88.

6. Mahfoud F, Böhm M, Bongarth CM, Bosch R, Schmieder RE, Schunkert H, et al. Kommentar zu den Leitlinien (2018) der Europäischen Gesellschaft für Kardiologie (ESC) und der Europäischen Gesellschaft für Hypertonie (ESH) für das Management der arteriellen Hypertonie. Der Kardiologe. 2019;13(1):17-23.

7. World Health Organisation. Obesity: preventing and managing the global epidemic. Report of a WHO consultation. World Health Organ Tech Rep Ser. 2000;894:i-xii, 1-253.
