# Supplementary material for: Genetic polymorphisms affecting telomere length and their association with cardiovascular disease in the Heinz-Nixdorf-Recall study
Source: PLoS One. 2024 May 14;19(5):e0303357. doi: 10.1371/journal.pone.0303357 (PMC11093374; doi:10.1371/journal.pone.0303357)
Supplement: S3 Table — Linkage disequilibrium of SNPs on chromosome 3 (a), chromosome 5 (b), chromosome 10 (c) and chromosome 19 (d). Linkage disequilibrium for the SNPs on the corresponding chromosome portrayed as D`/R2 Marked values: D`= 1.0 und R2>0.8. (DOCX) [file pone.0303357.s003.docx]

**S3 Table:** **Linkage disequilibrium of SNPs on chromosome 3 (a), chromosome 5 (b), chromosome 10 (c) and chromosome 19 (d)**

a)

|  | rs6772228 | rs12696304 | rs10936599 | rs1317082 |
| --- | --- | --- | --- | --- |
| rs6772228 | 1.00/1.00 | 0.11/0.000 | 0.02/0.000 | 0.024/0.00 |
| rs12696304 | 0.11/0.00 | 1.00/1.000 | 0.99/0.88 | 0.99/0.88 |
| rs10936599 | 0.02/0.00 | 0.99/0.876 | 1.00/1.00 | 1.00/1.00 |
| rs1317082 | 0.02/0.00 | 0.99/0.876 | 1.00/1.00 | 1.00/1.00 |

b)

|  | rs7726159 | rs2736100 |
| --- | --- | --- |
| rs7726159 | 1.00/1.00 | 0.98/0.52 |
| rs2736100 | 0.98/0.52 | 1.00/1.00 |

c)

|  | rs2487999 | rs9419958 | rs9420907 | rs4387287 |
| --- | --- | --- | --- | --- |
| rs2487999 | 1.00/1.00 | 0.98/0.64 | 0.98/0.64 | 0.97/0.53 |
| rs9419958 | 0.98/0.64 | 1.00/1.00 | 1.00/1.00 | 1.00/0.82 |
| rs9420907 | 0.98/0.64 | 1.00/1.00 | 1.00/1.00 | 1.00/0.82 |
| rs4387287 | 0.98/0.53 | 1.00/0.82 | 1.00/0.82 | 1.00/1.00 |

d)

|  | rs8105767 | rs412658 |
| --- | --- | --- |
| rs8105767 | 1.00/1.000 | 0.86/0.53 |
| rs412658 | 0.86/0.534 | 1.00/1.00 |

Linkage disequilibrium for the SNPs on the corresponding chromosome portrayed as D`/R2 Marked values: D` = 1.0 und R2>0.8
