# Supplementary material for: Genetic polymorphisms affecting telomere length and their association with cardiovascular disease in the Heinz-Nixdorf-Recall study
Source: PLoS One. 2024 May 14;19(5):e0303357. doi: 10.1371/journal.pone.0303357 (PMC11093374; doi:10.1371/journal.pone.0303357)
Supplement: S4 Table — coef: coefficient, HR: Hazard Ratio, se: Standard error, lower/upper: lower/upper boundarie of the 95% confidence interval. a) crude, b) adjusted, c1) young age, c2) older age, d1) male, d2) female, e1) low hsCRP, e2) intermediate hsCRP, e3) high hsCRP, f1) low total cholesterol, f2) high total cholesterol, g1) low LDL, g2) high LDL, h1) normal HDL, h2) high HDL, i1) low triglycerides, i2) high triglycerides, j1) ideal blood pressure, j2) normal/high normal blood pressure, j3) hypertension, k1) no diabetes, k2) diabetes, l1) never smoker, l2) former smoker, l3) current smoker, m1) normal waist circumference, m2) high waist circumference, n1) normal BMI, n2) high BMI. (PDF) [file pone.0303357.s004.pdf]

S4A Table. Results of Cox regression models for rs10936599.
coef: coefficient, HR: Hazard Ratio, se: Standard error,
lower/upper: lower/upper boundarie of the 95% confidence interval
a) crude, b) adjusted, c1) young age, c2) older age, d1) male, d2) female,
e1) low hsCRP, e2) intermediate hsCRP, e3) high hsCRP, f1) low total cholesterol,
f2) high total cholesterol, g1) low LDL, g2) high LDL, h1) normal HDL, h2) high HDL,
i1) low triglycerides, i2) high triglycerides, j1) ideal blood pressure,
j2) normal/high normal blood pressure, j3) hypertension, k1) no diabetes, k2) diabetes,
l1) never smoker, l2) former smoker, l3) current smoker, m1) normal waist circumference,
m2) high waist circumference, n1) normal BMI, n2) high BMI

a)

	coef	HR	se	p	lower	upper
rs10936599	0.0457	1.0468	0.0743	0.5381	0.905	1.2108

b)

	coef	HR	se	p	lower	upper
rs10936599	0.0124	1.0125	0.0787	0.8748	0.8678	1.1812
sex	−0.1113	0.8947	0.1489	0.455	0.6682	1.1980
age	0.0593	1.0611	0.0081	< 0.0001	1.0444	1.0781
total cholesterol	−0.0022	0.9978	0.0038	0.5574	0.9904	1.0052
HDL	−0.0044	0.9956	0.0050	0.377	0.9860	1.0054
LDL	−0.0010	0.9990	0.0038	0.7972	0.9915	1.0066
triglycerides	0.0011	1.0011	0.0006	0.0895	0.9998	1.0024
diabetes	0.1548	1.1675	0.0799	0.0527	0.9982	1.3654
smoking	0.1815	1.1990	0.0695	0.0091	1.0462	1.3740
systolic bloodpressure	0.0071	1.0071	0.0034	0.0369	1.0004	1.0138
diastolic bloodpressure	−0.0049	0.9951	0.0065	0.4499	0.9824	1.0079
CRP	0.0015	1.0015	0.0702	0.983	0.8728	1.1491
BMI	0.0044	1.0044	0.0222	0.8445	0.9616	1.0490
physical activity	0.1605	1.1741	0.0983	0.1025	0.9684	1.4237
waist circumference	−0.0083	0.9918	0.0087	0.3436	0.9749	1.0089
coronary artery calcification	0.2752	1.3168	0.0250	< 0.0001	1.2538	1.3828

c1)

	coef	HR	se	p	lower	upper
rs10936599	0.4452	1.5609	0.1527	0.0035	1.1573	2.1053
sex	−0.0966	0.9079	0.2877	0.7369	0.5166	1.5956
total cholesterol	0.0030	1.0030	0.0067	0.6527	0.9900	1.0162
HDL	−0.0153	0.9848	0.0100	0.1279	0.9657	1.0044
LDL	−0.0030	0.9970	0.0068	0.6658	0.9838	1.0105
triglycerides	0.0002	1.0002	0.0013	0.8622	0.9976	1.0029
diabetes	0.0316	1.0321	0.1979	0.8732	0.7003	1.5212
smoking	0.3052	1.3569	0.1353	0.0241	1.0408	1.7690
systolic bloodpressure	−0.0031	0.9969	0.0089	0.7274	0.9796	1.0145
diastolic bloodpressure	0.0171	1.0173	0.0163	0.2932	0.9853	1.0502
CRP	0.1058	1.1116	0.1583	0.5038	0.8151	1.5160
BMI	0.0129	1.0130	0.0468	0.7823	0.9243	1.1102
physical activity	0.2045	1.2269	0.2138	0.3388	0.8069	1.8655
waist circumference	−0.0149	0.9852	0.0182	0.4128	0.9507	1.0210
coronary artery calcification	0.2907	1.3374	0.0460	< 0.0001	1.2220	1.4637

c2)

	coef	HR	se	p	lower	upper
rs10936599	−0.1361	0.8727	0.0932	0.1441	0.7270	1.0476
sex	0.3254	1.3846	0.1751	0.0632	0.9823	1.9516
total cholesterol	−0.0045	0.9955	0.0041	0.2695	0.9875	1.0035
HDL	−0.0002	0.9998	0.0044	0.9698	0.9913	1.0084
LDL	0.0000	1.0000	0.0042	0.9967	0.9917	1.0084
triglycerides	0.0011	1.0011	0.0007	0.0926	0.9998	1.0024
diabetes	0.1880	1.2069	0.0883	0.0332	1.0151	1.4348
smoking	0.0813	1.0847	0.0819	0.3209	0.9238	1.2735
systolic bloodpressure	0.0138	1.0139	0.0036	0.0001	1.0069	1.0211
diastolic bloodpressure	−0.0182	0.9819	0.0070	0.0097	0.9685	0.9956
CRP	−0.0092	0.9909	0.0741	0.9016	0.8570	1.1457
BMI	−0.0070	0.9931	0.0258	0.7876	0.9440	1.0447
physical activity	0.1367	1.1464	0.1112	0.2193	0.9219	1.4257
waist circumference	−0.0023	0.9977	0.0100	0.8158	0.9782	1.0175
coronary artery calcification	0.2904	1.3369	0.0294	< 0.0001	1.2621	1.4162

d1)

	coef	HR	se	p	lower	upper
rs10936599	−0.1169	0.8897	0.0984	0.235	0.7336	1.0790
age	0.0436	1.0446	0.0096	< 0.0001	1.0251	1.0645
total cholesterol	−0.0061	0.9940	0.0048	0.208	0.9846	1.0034
HDL	0.0022	1.0022	0.0061	0.7214	0.9902	1.0143
LDL	0.0028	1.0028	0.0049	0.5696	0.9932	1.0124
triglycerides	0.0015	1.0015	0.0008	0.0536	1.0000	1.0030
diabetes	0.1749	1.1912	0.0908	0.0541	0.9969	1.4232
smoking	0.1107	1.1171	0.0891	0.2142	0.9380	1.3303
systolic bloodpressure	0.0048	1.0048	0.0042	0.2571	0.9965	1.0132
diastolic bloodpressure	−0.0027	0.9973	0.0080	0.7324	0.9818	1.0130
CRP	0.0004	1.0004	0.0788	0.9957	0.8572	1.1676
BMI	0.0387	1.0394	0.0294	0.1882	0.9813	1.1010
physical activity	0.1628	1.1768	0.1208	0.1778	0.9287	1.4913
waist circumference	−0.0140	0.9861	0.0113	0.2167	0.9645	1.0082
coronary artery calcification	0.2986	1.3480	0.0333	< 0.0001	1.2629	1.4388

d2)

	coef	HR	se	p	lower	upper
rs10936599	0.2926	1.3399	0.1332	0.028	1.0320	1.7397
age	0.1003	1.1055	0.0152	< 0.0001	1.0729	1.1390
total cholesterol	0.0026	1.0026	0.0055	0.6403	0.9918	1.0134
HDL	−0.0155	0.9846	0.0081	0.0553	0.9690	1.0004
LDL	−0.0066	0.9934	0.0056	0.2382	0.9825	1.0044
triglycerides	0.0011	1.0011	0.0013	0.4169	0.9985	1.0036
diabetes	0.1163	1.1233	0.1715	0.4977	0.8026	1.5722
smoking	0.3307	1.3919	0.1096	0.0026	1.1228	1.7255
systolic bloodpressure	0.0097	1.0098	0.0057	0.0873	0.9986	1.0211
diastolic bloodpressure	−0.0075	0.9926	0.0118	0.5274	0.9699	1.0158
CRP	0.0157	1.0159	0.1442	0.9131	0.7658	1.3476
BMI	−0.0307	0.9698	0.0349	0.3803	0.9056	1.0386
physical activity	0.1480	1.1595	0.1709	0.3865	0.8294	1.6210
waist circumference	−0.0069	0.9931	0.0140	0.6213	0.9662	1.0208
coronary artery calcification	0.2344	1.2641	0.0387	< 0.0001	1.1717	1.3639

e1)

	coef	HR	se	p	lower	upper
rs10936599	−0.0800	0.9231	0.0979	0.4134	0.7620	1.1182
sex	−0.1825	0.8331	0.1817	0.315	0.5835	1.1895
age	0.0684	1.0708	0.0098	< 0.0001	1.0504	1.0916
total cholesterol	−0.0001	0.9999	0.0043	0.9898	0.9915	1.0084
HDL	−0.0061	0.9939	0.0058	0.2878	0.9827	1.0052
LDL	−0.0038	0.9962	0.0044	0.3944	0.9876	1.0049
triglycerides	0.0011	1.0011	0.0007	0.1477	0.9996	1.0025
diabetes	0.1334	1.1427	0.1011	0.1869	0.9373	1.3931
smoking	0.1778	1.1946	0.0858	0.0383	1.0096	1.4134
systolic bloodpressure	0.0078	1.0079	0.0043	0.0659	0.9995	1.0163
diastolic bloodpressure	−0.0044	0.9956	0.0083	0.5955	0.9795	1.0119
BMI	0.0392	1.0400	0.0280	0.1618	0.9844	1.0988
physical activity	0.1777	1.1945	0.1183	0.1331	0.9473	1.5062
waist circumference	−0.0169	0.9832	0.0105	0.1064	0.9632	1.0036
coronary artery calcification	0.2723	1.3130	0.0301	< 0.0001	1.2378	1.3928

e2)

	coef	HR	se	p	lower	upper
rs10936599	−0.2679	0.765	0.2664	0.3146	0.4538	1.2895
sex	0.6093	1.8391	0.4478	0.1737	0.7646	4.4237
age	0.0933	1.0978	0.0248	0.0002	1.0457	1.1525
total cholesterol	−0.0055	0.9945	0.0129	0.667	0.9697	1.0199
HDL	−0.0076	0.9924	0.0166	0.6482	0.9606	1.0253
LDL	0.0057	1.0057	0.0121	0.641	0.982	1.0299
triglycerides	−0.0037	0.9963	0.0026	0.1562	0.9911	1.0014
diabetes	0.3603	1.4338	0.1936	0.0627	0.9811	2.0953
smoking	0.1676	1.1825	0.2126	0.4305	0.7795	1.7937
systolic bloodpressure	−0.0029	0.9971	0.0094	0.76	0.979	1.0156
diastolic bloodpressure	0.0088	1.0088	0.0172	0.6097	0.9754	1.0433
BMI	−0.1185	0.8883	0.0722	0.1009	0.7711	1.0233
physical activity	−0.5343	0.5861	0.2856	0.0614	0.3348	1.0258
waist circumference	0.0556	1.0572	0.0287	0.0527	0.9993	1.1184
coronary artery calcification	0.285	1.3298	0.0745	0.0001	1.1491	1.5388

e3)

	coef	HR	se	p	lower	upper
rs10936599	0.5533	1.7390	0.1630	0.0007	1.2635	2.3935
sex	−0.2527	0.7767	0.3516	0.4723	0.3900	1.5471
age	0.0075	1.0075	0.0187	0.6877	0.9713	1.0452
total cholesterol	−0.0035	0.9965	0.0065	0.59	0.9838	1.0093
HDL	0.0007	1.0007	0.0038	0.8619	0.9932	1.0082
LDL	0.0016	1.0016	0.0068	0.8186	0.9883	1.0150
triglycerides	0.0022	1.0022	0.0012	0.0786	0.9998	1.0046
diabetes	0.1775	1.1942	0.1760	0.3134	0.8457	1.6863
smoking	0.1102	1.1166	0.1600	0.4907	0.8161	1.5277
systolic bloodpressure	0.0086	1.0087	0.0072	0.2267	0.9946	1.0229
diastolic bloodpressure	−0.0226	0.9776	0.0150	0.1306	0.9494	1.0067
BMI	−0.0502	0.9511	0.0486	0.3018	0.8646	1.0461
physical activity	0.5299	1.6988	0.2441	0.03	1.0528	2.7411
waist circumference	−0.0147	0.9854	0.0200	0.4625	0.9476	1.0248
coronary artery calcification	0.3016	1.3520	0.0589	< 0.0001	1.2047	1.5173

f1)

	coef	HR	se	p	lower	upper
rs10936599	−0.0711	0.9314	0.1567	0.65	0.6851	1.2661
sex	0.3993	1.4909	0.2712	0.1409	0.8761	2.5370
age	0.0733	1.0760	0.0162	< 0.0001	1.0425	1.1107
HDL	−0.0062	0.9938	0.0085	0.4684	0.9773	1.0106
LDL	−0.0048	0.9952	0.0048	0.3149	0.9860	1.0046
triglycerides	0.0007	1.0007	0.0013	0.6145	0.9981	1.0033
diabetes	0.2057	1.2284	0.1495	0.1687	0.9165	1.6465
smoking	−0.0071	0.9929	0.1320	0.9568	0.7666	1.2860
systolic bloodpressure	0.0034	1.0034	0.0068	0.6109	0.9902	1.0168
diastolic bloodpressure	−0.0024	0.9976	0.0132	0.8564	0.9722	1.0237
CRP	−0.0407	0.9602	0.1238	0.7426	0.7533	1.2238
BMI	−0.1030	0.9022	0.0437	0.0184	0.8282	0.9828
physical activity	0.2568	1.2927	0.1916	0.1802	0.8880	1.8819
waist circumference	0.0294	1.0298	0.0161	0.0688	0.9977	1.0629
coronary artery calcification	0.3007	1.3508	0.0481	< 0.0001	1.2293	1.4843

f2)

	coef	HR	se	p	lower	upper
rs10936599	0.0467	1.0478	0.0915	0.6098	0.8758	1.2535
sex	−0.3023	0.7391	0.1773	0.0882	0.5221	1.0463
age	0.0555	1.0571	0.0094	< 0.0001	1.0377	1.0768
HDL	−0.0060	0.9940	0.0044	0.171	0.9854	1.0026
LDL	−0.0030	0.9970	0.0019	0.1252	0.9932	1.0008
triglycerides	0.0007	1.0007	0.0005	0.1308	0.9998	1.0017
diabetes	0.1239	1.1319	0.0954	0.1943	0.9388	1.3647
smoking	0.2602	1.2973	0.0828	0.0017	1.1029	1.5258
systolic bloodpressure	0.0080	1.0080	0.0040	0.0444	1.0002	1.0159
diastolic bloodpressure	−0.0065	0.9935	0.0076	0.3952	0.9787	1.0085
CRP	0.0151	1.0152	0.0852	0.8596	0.8590	1.1997
BMI	0.0466	1.0477	0.0255	0.0677	0.9966	1.1014
physical activity	0.1211	1.1288	0.1166	0.2988	0.8982	1.4185
waist circumference	−0.0219	0.9784	0.0101	0.0312	0.9591	0.9980
coronary artery calcification	0.2661	1.3048	0.0297	< 0.0001	1.2311	1.3830

g1)

	coef	HR	se	p	lower	upper
rs10936599	−0.0587	0.9430	0.1794	0.7436	0.6635	1.3403
sex	0.1672	1.1820	0.2935	0.5689	0.6649	2.1011
age	0.0819	1.0853	0.0176	< 0.0001	1.0486	1.1233
total cholesterol	−0.0021	0.9979	0.0046	0.6472	0.9889	1.0070
HDL	−0.0009	0.9991	0.0068	0.8946	0.9859	1.0124
triglycerides	0.0018	1.0018	0.0007	0.0123	1.0004	1.0032
diabetes	0.2174	1.2428	0.1675	0.1942	0.8951	1.7256
smoking	0.1857	1.2041	0.1431	0.1943	0.9096	1.5939
systolic bloodpressure	0.0016	1.0016	0.0073	0.8286	0.9873	1.0161
diastolic bloodpressure	−0.0057	0.9943	0.0139	0.6809	0.9676	1.0217
CRP	−0.2786	0.7569	0.2334	0.2327	0.4790	1.1960
BMI	−0.0749	0.9278	0.0459	0.103	0.8479	1.0153
physical activity	0.4897	1.6319	0.2102	0.0198	1.0810	2.4636
waist circumference	0.0215	1.0218	0.0173	0.2138	0.9877	1.0571
coronary artery calcification	0.2696	1.3095	0.0517	< 0.0001	1.1833	1.4491

g2)

	coef	HR	se	p	lower	upper
rs10936599	0.0288	1.0292	0.0881	0.744	0.8660	1.2232
sex	−0.1837	0.8322	0.1720	0.2855	0.5940	1.1658
age	0.0543	1.0558	0.0092	< 0.0001	1.0369	1.0750
total cholesterol	−0.0032	0.9968	0.0019	0.0826	0.9931	1.0004
HDL	−0.0041	0.9960	0.0047	0.3843	0.9869	1.0051
triglycerides	0.0010	1.0010	0.0007	0.1672	0.9996	1.0023
diabetes	0.1334	1.1427	0.0921	0.1477	0.9539	1.3688
smoking	0.1990	1.2202	0.0801	0.013	1.0429	1.4275
systolic bloodpressure	0.0079	1.0079	0.0039	0.0427	1.0003	1.0156
diastolic bloodpressure	−0.0042	0.9958	0.0075	0.5749	0.9813	1.0105
CRP	0.0883	1.0923	0.0846	0.2968	0.9254	1.2894
BMI	0.0249	1.0253	0.0250	0.3191	0.9762	1.0768
physical activity	0.0811	1.0845	0.1127	0.4715	0.8696	1.3525
waist circumference	−0.0166	0.9835	0.0099	0.0927	0.9646	1.0028
coronary artery calcification	0.2763	1.3183	0.0290	< 0.0001	1.2455	1.3953

h1)

	coef	HR	se	p	lower	upper
rs10936599	−0.0079	0.9921	0.0912	0.931	0.8298	1.1862
sex	−0.1296	0.8784	0.1727	0.4529	0.6262	1.2323
age	0.0700	1.0725	0.0098	< 0.0001	1.0520	1.0933
total cholesterol	−0.0019	0.9981	0.0035	0.5902	0.9914	1.0049
LDL	−0.0005	0.9995	0.0036	0.8844	0.9925	1.0065
triglycerides	−0.0001	0.9999	0.0008	0.8599	0.9982	1.0015
diabetes	0.1712	1.1867	0.0981	0.0811	0.9791	1.4384
smoking	0.2384	1.2692	0.0826	0.0039	1.0794	1.4924
systolic bloodpressure	0.0094	1.0095	0.0039	0.0155	1.0018	1.0172
diastolic bloodpressure	−0.0040	0.9960	0.0076	0.5982	0.9813	1.0110
CRP	−0.0795	0.9236	0.0995	0.4245	0.7599	1.1225
BMI	0.0200	1.0202	0.0260	0.4428	0.9694	1.0736
physical activity	0.1793	1.1964	0.1147	0.1179	0.9556	1.4979
waist circumference	−0.0072	0.9929	0.0104	0.4918	0.9728	1.0133
coronary artery calcification	0.2552	1.2907	0.0285	< 0.0001	1.2206	1.3648

h2)

	coef	HR	se	p	lower	upper
rs10936599	0.0301	1.0305	0.1591	0.8501	0.7545	1.4075
sex	−0.2627	0.7690	0.2817	0.3512	0.4427	1.3358
age	0.0345	1.0351	0.0147	0.0187	1.0057	1.0652
total cholesterol	−0.0046	0.9954	0.0058	0.4321	0.9841	1.0069
LDL	0.0006	1.0006	0.0063	0.9202	0.9884	1.0130
triglycerides	0.0021	1.0021	0.0008	0.0117	1.0005	1.0037
diabetes	0.0691	1.0716	0.1418	0.6259	0.8115	1.4150
smoking	0.0426	1.0435	0.1344	0.7515	0.8018	1.3580
systolic bloodpressure	0.0006	1.0006	0.0069	0.9333	0.9872	1.0142
diastolic bloodpressure	−0.0061	0.9939	0.0133	0.6466	0.9684	1.0201
CRP	0.1697	1.1849	0.1169	0.1465	0.9424	1.4899
BMI	−0.0344	0.9662	0.0431	0.4251	0.8880	1.0513
physical activity	0.1173	1.1244	0.1917	0.5407	0.7722	1.6374
waist circumference	−0.0150	0.9851	0.0164	0.3603	0.9539	1.0173
coronary artery calcification	0.3324	1.3943	0.0525	< 0.0001	1.2578	1.5456

i1)

	coef	HR	se	p	lower	upper
rs10936599	−0.0176	0.9826	0.1065	0.8687	0.7975	1.2105
sex	−0.0981	0.9066	0.2007	0.6251	0.6117	1.3436
age	0.0732	1.0760	0.0113	< 0.0001	1.0523	1.1002
total cholesterol	−0.0014	0.9986	0.0052	0.7921	0.9884	1.0089
HDL	−0.0024	0.9976	0.0064	0.7038	0.9852	1.0101
LDL	−0.0028	0.9972	0.0054	0.6023	0.9867	1.0078
diabetes	0.0416	1.0425	0.1258	0.7407	0.8147	1.3340
smoking	0.3057	1.3575	0.0942	0.0012	1.1286	1.6329
systolic bloodpressure	0.0045	1.0045	0.0046	0.3303	0.9954	1.0137
diastolic bloodpressure	0.0127	1.0128	0.0091	0.1618	0.9949	1.0310
CRP	−0.0540	0.9474	0.0894	0.5456	0.7951	1.1289
BMI	0.0286	1.0290	0.0312	0.3586	0.9681	1.0938
physical activity	0.3494	1.4183	0.1335	0.0089	1.0917	1.8425
waist circumference	−0.0151	0.9850	0.0119	0.2056	0.9623	1.0083
coronary artery calcification	0.2782	1.3208	0.0330	< 0.0001	1.2381	1.4091

i2)

	coef	HR	se	p	lower	upper
rs10936599	0.0438	1.0448	0.1185	0.7118	0.8282	1.3180
sex	−0.1173	0.8893	0.2264	0.6045	0.5706	1.3862
age	0.0445	1.0455	0.0116	0.0001	1.0220	1.0696
total cholesterol	0.0019	1.0019	0.0034	0.564	0.9954	1.0086
HDL	−0.0176	0.9825	0.0070	0.0122	0.9691	0.9962
LDL	−0.0039	0.9961	0.0035	0.275	0.9893	1.0031
diabetes	0.2490	1.2827	0.1061	0.019	1.0418	1.5794
smoking	0.0556	1.0572	0.1051	0.5968	0.8604	1.2989
systolic bloodpressure	0.0098	1.0099	0.0049	0.0451	1.0002	1.0196
diastolic bloodpressure	−0.0245	0.9758	0.0098	0.012	0.9573	0.9946
CRP	0.1185	1.1258	0.1350	0.3802	0.8640	1.4668
BMI	−0.0240	0.9763	0.0326	0.4621	0.9158	1.0408
physical activity	−0.1205	0.8865	0.1458	0.4085	0.6662	1.1796
waist circumference	0.0005	1.0005	0.0129	0.971	0.9754	1.0262
coronary artery calcification	0.2671	1.3061	0.0386	< 0.0001	1.2109	1.4089

j1)

	coef	HR	se	p	lower	upper
rs10936599	0.4432	1.5576	0.2020	0.0282	1.0485	2.3141
sex	−0.0775	0.9255	0.3663	0.8325	0.4514	1.8975
age	0.0606	1.0625	0.0188	0.0013	1.0240	1.1023
total cholesterol	0.0162	1.0163	0.0109	0.1377	0.9948	1.0382
HDL	−0.0287	0.9717	0.0135	0.0336	0.9463	0.9978
LDL	−0.0171	0.9831	0.0105	0.1038	0.9631	1.0035
triglycerides	0.0017	1.0017	0.0025	0.4832	0.9969	1.0066
diabetes	−0.1115	0.8945	0.2235	0.6179	0.5772	1.3862
smoking	0.2138	1.2384	0.1773	0.2277	0.8749	1.7529
CRP	0.1358	1.1455	0.1519	0.3711	0.8506	1.5426
BMI	0.0083	1.0083	0.0595	0.8893	0.8973	1.1331
physical activity	0.2886	1.3345	0.2637	0.2739	0.7959	2.2377
waist circumference	−0.0146	0.9855	0.0236	0.5348	0.9409	1.0321
coronary artery calcification	0.3601	1.4335	0.0619	< 0.0001	1.2697	1.6184

j2)

	coef	HR	se	p	lower	upper
rs10936599	−0.0723	0.9302	0.1383	0.6009	0.7094	1.2198
sex	−0.5789	0.5605	0.2637	0.0281	0.3343	0.9398
age	0.0710	1.0736	0.0125	< 0.0001	1.0475	1.1003
total cholesterol	0.0013	1.0013	0.0054	0.8182	0.9906	1.0120
HDL	−0.0096	0.9905	0.0083	0.2503	0.9744	1.0068
LDL	−0.0036	0.9964	0.0055	0.5183	0.9856	1.0073
triglycerides	0.0004	1.0004	0.0012	0.7279	0.9981	1.0028
diabetes	0.2623	1.3000	0.1314	0.0459	1.0048	1.6819
smoking	0.2417	1.2735	0.1171	0.039	1.0123	1.6020
CRP	0.0611	1.0630	0.1657	0.7125	0.7681	1.4710
BMI	0.0655	1.0677	0.0369	0.0757	0.9932	1.1477
physical activity	0.2108	1.2346	0.1648	0.201	0.8938	1.7055
waist circumference	−0.0350	0.9656	0.0147	0.0171	0.9383	0.9938
coronary artery calcification	0.2133	1.2377	0.0394	< 0.0001	1.1458	1.3371

j3)

	coef	HR	se	p	lower	upper
rs10936599	−0.0409	0.9600	0.1126	0.7167	0.7698	1.1971
sex	0.1452	1.1563	0.2071	0.4833	0.7704	1.7353
age	0.0614	1.0634	0.0115	< 0.0001	1.0397	1.0876
total cholesterol	−0.0073	0.9927	0.0044	0.0963	0.9841	1.0013
HDL	0.0019	1.0019	0.0021	0.362	0.9978	1.0062
LDL	0.0025	1.0025	0.0047	0.5966	0.9933	1.0118
triglycerides	0.0014	1.0014	0.0007	0.0354	1.0001	1.0028
diabetes	0.1395	1.1497	0.1127	0.2159	0.9218	1.4339
smoking	0.1610	1.1747	0.1021	0.1149	0.9616	1.4349
CRP	−0.0505	0.9507	0.0963	0.5995	0.7872	1.1481
BMI	−0.0336	0.9670	0.0323	0.2986	0.9076	1.0302
physical activity	0.0631	1.0651	0.1410	0.6546	0.8079	1.4043
waist circumference	0.0073	1.0073	0.0125	0.5583	0.9830	1.0322
coronary artery calcification	0.2982	1.3474	0.0380	< 0.0001	1.2506	1.4517

k1)

	coef	HR	se	p	lower	upper
rs10936599	0.0054	1.0054	0.0909	0.9527	0.8414	1.2014
sex	−0.1073	0.8982	0.1707	0.5294	0.6429	1.2550
age	0.0650	1.0672	0.0094	< 0.0001	1.0478	1.0869
total cholesterol	−0.0001	0.9999	0.0042	0.9859	0.9917	1.0083
HDL	−0.0073	0.9927	0.0058	0.2121	0.9815	1.0042
LDL	−0.0022	0.9978	0.0043	0.6097	0.9894	1.0062
triglycerides	0.0002	1.0002	0.0009	0.838	0.9984	1.0019
smoking	0.2919	1.3390	0.0796	0.0002	1.1456	1.5650
systolic bloodpressure	0.0040	1.0040	0.0042	0.3363	0.9958	1.0122
diastolic bloodpressure	0.0036	1.0036	0.0080	0.6577	0.9879	1.0195
CRP	−0.1109	0.8950	0.1141	0.3311	0.7156	1.1193
BMI	0.0238	1.0241	0.0267	0.3716	0.9720	1.0791
physical activity	0.2038	1.2260	0.1138	0.0733	0.9809	1.5323
waist circumference	−0.0089	0.9911	0.0102	0.3815	0.9715	1.0111
coronary artery calcification	0.2628	1.3006	0.0283	< 0.0001	1.2305	1.3747

k2)

	coef	HR	se	p	lower	upper
rs10936599	−0.0932	0.9110	0.1641	0.57	0.6604	1.2567
sex	−0.3931	0.6749	0.3231	0.2237	0.3583	1.2714
age	0.0475	1.0487	0.0168	0.0046	1.0147	1.0837
total cholesterol	−0.0053	0.9947	0.0091	0.5604	0.9770	1.0127
HDL	0.0057	1.0057	0.0101	0.5762	0.9859	1.0258
LDL	0.0011	1.0011	0.0092	0.9039	0.9832	1.0194
triglycerides	0.0021	1.0021	0.0011	0.0649	0.9999	1.0044
smoking	−0.1155	0.8910	0.1561	0.4595	0.6561	1.2098
systolic bloodpressure	0.0107	1.0107	0.0061	0.0809	0.9987	1.0229
diastolic bloodpressure	−0.0183	0.9819	0.0117	0.117	0.9597	1.0046
CRP	0.1133	1.1200	0.0836	0.1753	0.9507	1.3194
BMI	−0.0159	0.9842	0.0443	0.7193	0.9023	1.0735
physical activity	−0.0974	0.9072	0.2012	0.6284	0.6115	1.3458
waist circumference	−0.0161	0.9840	0.0177	0.3644	0.9504	1.0188
coronary artery calcification	0.3221	1.3800	0.0570	< 0.0001	1.2341	1.5431

I1)

	coef	HR	se	p	lower	upper
rs10936599	−0.0252	0.9752	0.1359	0.8532	0.7471	1.2729
sex	−0.1570	0.8547	0.2335	0.5013	0.5409	1.3507
age	0.0875	1.0915	0.0155	< 0.0001	1.0589	1.1251
total cholesterol	−0.0038	0.9962	0.0067	0.5732	0.9831	1.0095
HDL	−0.0099	0.9902	0.0090	0.2745	0.9728	1.0079
LDL	−0.0015	0.9985	0.0068	0.8196	0.9852	1.0118
triglycerides	0.0014	1.0014	0.0015	0.3595	0.9984	1.0045
diabetes	0.3799	1.4621	0.1412	0.0071	1.1086	1.9283
systolic bloodpressure	0.0090	1.0090	0.0060	0.1357	0.9972	1.0209
diastolic bloodpressure	−0.0067	0.9934	0.0119	0.5748	0.9705	1.0168
CRP	0.0165	1.0166	0.1089	0.8797	0.8212	1.2586
BMI	−0.0088	0.9913	0.0346	0.8002	0.9262	1.0609
physical activity	0.0223	1.0225	0.1705	0.896	0.7321	1.4282
waist circumference	−0.0029	0.9972	0.0139	0.8375	0.9704	1.0247
coronary artery calcification	0.2532	1.2882	0.0408	< 0.0001	1.1893	1.3953

l2)

	coef	HR	se	p	lower	upper
rs10936599	−0.2844	0.7524	0.1329	0.0323	0.5799	0.9763
sex	−0.4996	0.6068	0.2797	0.074	0.3507	1.0497
age	0.0555	1.0570	0.0126	< 0.0001	1.0312	1.0835
total cholesterol	−0.0021	0.9979	0.0064	0.7445	0.9854	1.0105
HDL	0.0049	1.0050	0.0079	0.5315	0.9895	1.0206
LDL	−0.0007	0.9993	0.0064	0.9176	0.9868	1.0120
triglycerides	0.0014	1.0014	0.0010	0.191	0.9993	1.0034
diabetes	0.0980	1.1030	0.1194	0.4117	0.8728	1.3939
systolic bloodpressure	0.0059	1.0060	0.0051	0.2411	0.9960	1.0160
diastolic bloodpressure	−0.0089	0.9912	0.0098	0.3652	0.9723	1.0104
CRP	−0.0611	0.9408	0.1606	0.7038	0.6867	1.2888
BMI	0.0646	1.0667	0.0403	0.1088	0.9857	1.1543
physical activity	0.2346	1.2643	0.1538	0.1274	0.9352	1.7093
waist circumference	−0.0227	0.9775	0.0150	0.1293	0.9493	1.0067
coronary artery calcification	0.2759	1.3178	0.0402	< 0.0001	1.2178	1.4259

l3)

	coef	HR	se	p	lower	upper
rs10936599	0.4403	1.5531	0.1476	0.0028	1.1630	2.0740
sex	0.1980	1.2190	0.2896	0.4941	0.6911	2.1502
age	0.0378	1.0386	0.0161	0.0186	1.0063	1.0718
total cholesterol	−0.0007	0.9993	0.0066	0.9209	0.9866	1.0123
HDL	−0.0088	0.9913	0.0100	0.38	0.9721	1.0109
LDL	−0.0013	0.9987	0.0068	0.8501	0.9854	1.0122
triglycerides	0.0006	1.0006	0.0011	0.5857	0.9985	1.0027
diabetes	0.0425	1.0435	0.1767	0.8097	0.7381	1.4753
systolic bloodpressure	0.0083	1.0083	0.0077	0.2822	0.9932	1.0236
diastolic bloodpressure	−0.0062	0.9938	0.0141	0.6602	0.9667	1.0217
CRP	0.0101	1.0102	0.1123	0.928	0.8107	1.2588
BMI	−0.0486	0.9526	0.0451	0.2815	0.8720	1.0406
physical activity	0.1077	1.1137	0.2046	0.5985	0.7459	1.6631
waist circumference	0.0095	1.0096	0.0182	0.6004	0.9742	1.0462
coronary artery calcification	0.2957	1.3440	0.0507	< 0.0001	1.2169	1.4844

m1)

	coef	HR	se	p	lower	upper
rs10936599	0.4166	1.5167	0.1619	0.0101	1.1044	2.0830
sex	0.1882	1.2071	0.2825	0.5053	0.6939	2.0998
age	0.0432	1.0442	0.0167	0.0098	1.0105	1.0790
total cholesterol	0.0094	1.0094	0.0076	0.2163	0.9945	1.0246
HDL	−0.0082	0.9918	0.0096	0.3933	0.9732	1.0107
LDL	−0.0094	0.9907	0.0077	0.2273	0.9758	1.0058
triglycerides	0.0001	1.0001	0.0013	0.9655	0.9975	1.0027
diabetes	0.3204	1.3776	0.1908	0.0932	0.9478	2.0025
smoking	0.2014	1.2231	0.1402	0.1508	0.9293	1.6099
systolic bloodpressure	0.0166	1.0168	0.0071	0.0192	1.0027	1.0310
diastolic bloodpressure	−0.0171	0.9831	0.0157	0.2767	0.9533	1.0138
CRP	0.1957	1.2162	0.1543	0.2048	0.8987	1.6457
BMI	−0.0047	0.9954	0.0485	0.9235	0.9051	1.0947
physical activity	0.4946	1.6399	0.2126	0.02	1.0811	2.4873
coronary artery calcification	0.2922	1.3393	0.0507	< 0.0001	1.2126	1.4793

m2)

	coef	HR	se	p	lower	upper
rs10936599	−0.0743	0.9284	0.0904	0.4113	0.7776	1.1084
sex	−0.1130	0.8932	0.1395	0.418	0.6795	1.1741
age	0.0631	1.0652	0.0092	< 0.0001	1.0461	1.0845
total cholesterol	−0.0048	0.9952	0.0042	0.257	0.9870	1.0035
HDL	−0.0044	0.9956	0.0057	0.4444	0.9845	1.0069
LDL	0.0009	1.0009	0.0043	0.8338	0.9925	1.0094
triglycerides	0.0013	1.0013	0.0007	0.0868	0.9998	1.0027
diabetes	0.1160	1.1230	0.0843	0.1687	0.9520	1.3248
smoking	0.1496	1.1613	0.0809	0.0644	0.9911	1.3607
systolic bloodpressure	0.0049	1.0049	0.0038	0.1959	0.9975	1.0124
diastolic bloodpressure	−0.0003	0.9997	0.0072	0.9626	0.9856	1.0139
CRP	−0.0378	0.9629	0.0845	0.6547	0.8159	1.1364
BMI	0.0004	1.0004	0.0140	0.9778	0.9733	1.0282
physical activity	0.0345	1.0351	0.1108	0.7558	0.8330	1.2861
coronary artery calcification	0.2667	1.3056	0.0288	< 0.0001	1.2340	1.3814

n1)

	coef	HR	se	p	lower	upper
rs10936599	0.2275	1.2555	0.1771	0.1989	0.8873	1.7766
sex	0.0280	1.0284	0.3366	0.9336	0.5316	1.9895
age	0.0638	1.0659	0.0193	0.001	1.0262	1.1071
total cholesterol	0.0107	1.0108	0.0082	0.1905	0.9947	1.0271
HDL	−0.0156	0.9845	0.0104	0.1334	0.9646	1.0048
LDL	−0.0135	0.9866	0.0082	0.0991	0.9710	1.0025
triglycerides	−0.0006	0.9994	0.0015	0.6923	0.9965	1.0024
diabetes	0.2230	1.2498	0.2414	0.3557	0.7786	2.0061
smoking	0.2259	1.2534	0.1457	0.1212	0.9420	1.6678
systolic bloodpressure	0.0075	1.0075	0.0078	0.3334	0.9923	1.0230
diastolic bloodpressure	−0.0034	0.9967	0.0166	0.8402	0.9647	1.0296
CRP	−0.0032	0.9968	0.1189	0.9785	0.7895	1.2585
physical activity	0.5924	1.8084	0.2353	0.0118	1.1402	2.8680
waist circumference	−0.0244	0.9759	0.0183	0.1818	0.9415	1.0115
coronary artery calcification	0.3797	1.4619	0.0603	< 0.0001	1.2990	1.6452

n2)

	coef	HR	se	p	lower	upper
rs10936599	−0.0241	0.9762	0.0876	0.7837	0.8222	1.1591
sex	−0.1638	0.8489	0.1404	0.2432	0.6448	1.1177
age	0.0584	1.0602	0.0089	< 0.0001	1.0418	1.0788
total cholesterol	−0.0054	0.9946	0.0041	0.1926	0.9866	1.0027
HDL	−0.0018	0.9982	0.0056	0.7486	0.9874	1.0092
LDL	0.0019	1.0019	0.0042	0.6451	0.9937	1.0102
triglycerides	0.0015	1.0015	0.0007	0.0332	1.0001	1.0029
diabetes	0.1313	1.1403	0.0828	0.1127	0.9695	1.3411
smoking	0.1661	1.1806	0.0787	0.0349	1.0118	1.3777
systolic bloodpressure	0.0062	1.0062	0.0038	0.1014	0.9988	1.0136
diastolic bloodpressure	−0.0058	0.9943	0.0072	0.4211	0.9804	1.0083
CRP	0.0038	1.0038	0.0852	0.9644	0.8495	1.1862
physical activity	0.0434	1.0444	0.1087	0.6897	0.8439	1.2924
waist circumference	−0.0042	0.9958	0.0057	0.4643	0.9848	1.0070
coronary artery calcification	0.2514	1.2858	0.0275	< 0.0001	1.2183	1.3569

S4B Table. Results of Cox regression models for rs6772228.
coef: coefficient, HR: Hazard Ratio, se: Standard error,
lower/upper: lower/upper boundarie of the 95% confidence interval
a) crude, b) adjusted, c1) young age, c2) older age, d1) male, d2) female,
e1) low hsCRP, e2) intermediate hsCRP, e3) high hsCRP, f1) low total cholesterol,
f2) high total cholesterol, g1) low LDL, g2) high LDL, h1) normal HDL, h2) high HDL,
i1) low triglycerides, i2) high triglycerides, j1) ideal blood pressure,
j2) normal/high normal blood pressure, j3) hypertension, k1) no diabetes, k2) diabetes,
l1) never smoker, l2) former smoker, l3) current smoker, m1) normal waist circumference,
m2) high waist circumference, n1) normal BMI, n2) high BMI

a)

	coef	HR	se	p	lower	upper
rs6772228	−0.2995	0.7412	0.2043	0.1427	0.4966	1.1063

b)

	coef	HR	se	p	lower	upper
rs6772228	−0.1137	0.8926	0.2142	0.5956	0.5866	1.3581
sex	−0.1589	0.8530	0.1533	0.2997	0.6317	1.1519
age	0.0588	1.0605	0.0083	< 0.0001	1.0434	1.0780
total cholesterol	−0.0026	0.9974	0.0039	0.5106	0.9899	1.0051
HDL	−0.0034	0.9966	0.0051	0.5012	0.9867	1.0066
LDL	−0.0007	0.9993	0.0039	0.8632	0.9916	1.0071
triglycerides	0.0012	1.0012	0.0007	0.0689	0.9999	1.0025
diabetes	0.1668	1.1815	0.0817	0.0412	1.0067	1.3867
smoking	0.1520	1.1642	0.0717	0.034	1.0115	1.3398
systolic bloodpressure	0.0063	1.0063	0.0034	0.0678	0.9995	1.0131
diastolic bloodpressure	−0.0046	0.9954	0.0067	0.4916	0.9824	1.0086
CRP	−0.0163	0.9838	0.0747	0.827	0.8499	1.1389
BMI	0.0049	1.0049	0.0231	0.8324	0.9604	1.0514
physical activity	0.1479	1.1593	0.1006	0.1415	0.9519	1.4119
waist circumference	−0.0064	0.9936	0.0090	0.4749	0.9763	1.0112
coronary artery calcification	0.2813	1.3249	0.0255	< 0.0001	1.2602	1.3929

c1)

	coef	HR	se	p	lower	upper
rs6772228	−0.4220	0.6558	0.4606	0.3596	0.2659	1.6172
sex	−0.1340	0.8746	0.2963	0.651	0.4893	1.5631
total cholesterol	0.0011	1.0011	0.0069	0.8736	0.9877	1.0146
HDL	−0.0140	0.9861	0.0105	0.181	0.9661	1.0065
LDL	−0.0013	0.9987	0.0070	0.8549	0.9851	1.0126
triglycerides	0.0004	1.0004	0.0013	0.7447	0.9978	1.0031
diabetes	−0.0173	0.9829	0.2036	0.9323	0.6595	1.4648
smoking	0.2601	1.2971	0.1391	0.0614	0.9876	1.7035
systolic bloodpressure	−0.0092	0.9908	0.0094	0.3281	0.9727	1.0093
diastolic bloodpressure	0.0239	1.0242	0.0169	0.1569	0.9909	1.0586
CRP	0.0378	1.0385	0.1959	0.847	0.7074	1.5246
BMI	0.0017	1.0017	0.0483	0.9717	0.9112	1.1012
physical activity	0.1147	1.1215	0.2206	0.603	0.7279	1.7281
waist circumference	−0.0101	0.9900	0.0189	0.5926	0.9540	1.0272
coronary artery calcification	0.3128	1.3672	0.0478	< 0.0001	1.2449	1.5016

c2)

	coef	HR	se	p	lower	upper
rs6772228	−0.0237	0.9766	0.2417	0.9219	0.6081	1.5684
sex	0.3067	1.3590	0.1781	0.0851	0.9585	1.9269
total cholesterol	−0.0043	0.9957	0.0041	0.3015	0.9877	1.0038
HDL	0.0003	1.0003	0.0041	0.9492	0.9923	1.0083
LDL	0.0001	1.0001	0.0043	0.9733	0.9918	1.0085
triglycerides	0.0012	1.0012	0.0007	0.072	0.9999	1.0025
diabetes	0.2041	1.2265	0.0909	0.0247	1.0263	1.4657
smoking	0.0522	1.0535	0.0840	0.5347	0.8936	1.2421
systolic bloodpressure	0.0134	1.0135	0.0036	0.0002	1.0063	1.0207
diastolic bloodpressure	−0.0178	0.9824	0.0072	0.014	0.9685	0.9964
CRP	−0.0193	0.9809	0.0774	0.8032	0.8428	1.1416
BMI	−0.0126	0.9874	0.0266	0.6342	0.9373	1.0402
physical activity	0.1504	1.1623	0.1136	0.1858	0.9302	1.4523
waist circumference	0.0012	1.0012	0.0102	0.9032	0.9814	1.0215
coronary artery calcification	0.2908	1.3375	0.0299	< 0.0001	1.2613	1.4184

d1)

	coef	HR	se	p	lower	upper
rs6772228	0.0217	1.0219	0.2505	0.9311	0.6254	1.6697
age	0.0416	1.0425	0.0099	< 0.0001	1.0225	1.0628
total cholesterol	−0.0062	0.9939	0.0050	0.2146	0.9842	1.0036
HDL	0.0042	1.0043	0.0063	0.5032	0.9919	1.0168
LDL	0.0029	1.0029	0.0050	0.5613	0.9931	1.0128
triglycerides	0.0016	1.0016	0.0008	0.0344	1.0001	1.0031
diabetes	0.2090	1.2324	0.0920	0.0231	1.0292	1.4759
smoking	0.0791	1.0823	0.0913	0.386	0.9051	1.2943
systolic bloodpressure	0.0047	1.0047	0.0043	0.2771	0.9962	1.0132
diastolic bloodpressure	−0.0030	0.9970	0.0081	0.7136	0.9812	1.0130
CRP	−0.0096	0.9904	0.0818	0.9066	0.8438	1.1626
BMI	0.0471	1.0482	0.0306	0.1235	0.9872	1.1130
physical activity	0.1621	1.1760	0.1228	0.1867	0.9245	1.4958
waist circumference	−0.0140	0.9861	0.0115	0.2225	0.9641	1.0085
coronary artery calcification	0.2998	1.3496	0.0337	< 0.0001	1.2633	1.4417

d2)

	coef	HR	se	p	lower	upper
rs6772228	−0.4273	0.6522	0.4198	0.3087	0.2865	1.4850
age	0.1051	1.1108	0.0159	< 0.0001	1.0767	1.1459
total cholesterol	0.0010	1.0010	0.0058	0.8637	0.9897	1.0125
HDL	−0.0154	0.9847	0.0085	0.0697	0.9684	1.0012
LDL	−0.0047	0.9953	0.0059	0.4282	0.9838	1.0069
triglycerides	0.0010	1.0010	0.0015	0.4869	0.9982	1.0039
diabetes	0.0495	1.0507	0.1839	0.7879	0.7328	1.5065
smoking	0.3088	1.3618	0.1138	0.0066	1.0896	1.7020
systolic bloodpressure	0.0069	1.0069	0.0059	0.2419	0.9954	1.0186
diastolic bloodpressure	−0.0037	0.9963	0.0122	0.7631	0.9728	1.0204
CRP	−0.0298	0.9706	0.1689	0.86	0.6971	1.3516
BMI	−0.0464	0.9547	0.0364	0.2027	0.8890	1.0253
physical activity	0.1169	1.1240	0.1771	0.5093	0.7944	1.5903
waist circumference	−0.0005	0.9995	0.0146	0.9707	0.9713	1.0285
coronary artery calcification	0.2460	1.2789	0.0401	< 0.0001	1.1824	1.3834

e1)

	coef	HR	se	p	lower	upper
rs6772228	−0.2092	0.8112	0.2652	0.4302	0.4824	1.3642
sex	−0.1997	0.8190	0.1862	0.2836	0.5686	1.1797
age	0.0673	1.0696	0.0100	< 0.0001	1.0488	1.0909
total cholesterol	−0.0003	0.9997	0.0045	0.9445	0.9909	1.0085
HDL	−0.0060	0.9940	0.0060	0.3139	0.9824	1.0057
LDL	−0.0034	0.9966	0.0046	0.4621	0.9878	1.0056
triglycerides	0.0011	1.0011	0.0007	0.1258	0.9997	1.0026
diabetes	0.1387	1.1488	0.1036	0.1809	0.9376	1.4075
smoking	0.1578	1.1710	0.0877	0.072	0.9860	1.3907
systolic bloodpressure	0.0081	1.0081	0.0043	0.0617	0.9996	1.0167
diastolic bloodpressure	−0.0035	0.9965	0.0085	0.6764	0.9800	1.0132
BMI	0.0359	1.0366	0.0287	0.2105	0.9799	1.0966
physical activity	0.1882	1.2070	0.1208	0.1195	0.9525	1.5296
waist circumference	−0.0147	0.9854	0.0107	0.1703	0.9648	1.0063
coronary artery calcification	0.2757	1.3175	0.0306	< 0.0001	1.2408	1.3989

e2)

	coef	HR	se	p	lower	upper
rs6772228	0.6018	1.8253	0.497	0.226	0.6891	4.8349
sex	0.5616	1.7534	0.4625	0.2247	0.7082	4.3412
age	0.0943	1.0989	0.0251	0.0002	1.046	1.1544
total cholesterol	0.0001	1.0001	0.013	0.9922	0.975	1.0259
HDL	−0.0158	0.9843	0.0172	0.3578	0.9517	1.018
LDL	0.0038	1.0038	0.0121	0.7552	0.9803	1.0278
triglycerides	−0.005	0.995	0.0028	0.0705	0.9896	1.0004
diabetes	0.3477	1.4159	0.1961	0.0762	0.9641	2.0793
smoking	0.145	1.156	0.2163	0.5026	0.7566	1.7663
systolic bloodpressure	−0.0065	0.9935	0.0097	0.5064	0.9748	1.0127
diastolic bloodpressure	0.0115	1.0116	0.0179	0.52	0.9767	1.0476
BMI	−0.1408	0.8686	0.0739	0.0568	0.7515	1.0041
physical activity	−0.7461	0.4742	0.2945	0.0113	0.2662	0.8446
waist circumference	0.0704	1.0729	0.029	0.0152	1.0136	1.1357
coronary artery calcification	0.2838	1.3281	0.0774	0.0002	1.1411	1.5458

e3)

	coef	HR	se	p	lower	upper
rs6772228	−0.1137	0.8925	0.5897	0.8471	0.2810	2.8350
sex	−0.4079	0.6650	0.3683	0.2681	0.3231	1.3689
age	0.0078	1.0078	0.0194	0.6887	0.9702	1.0469
total cholesterol	−0.0049	0.9951	0.0067	0.4631	0.9822	1.0082
HDL	0.0010	1.0010	0.0033	0.7532	0.9946	1.0076
LDL	0.0026	1.0026	0.0070	0.7094	0.9889	1.0165
triglycerides	0.0024	1.0024	0.0013	0.0589	0.9999	1.0049
diabetes	0.1890	1.2080	0.1775	0.287	0.8531	1.7107
smoking	0.0097	1.0097	0.1676	0.9541	0.7269	1.4025
systolic bloodpressure	0.0036	1.0036	0.0075	0.629	0.9890	1.0185
diastolic bloodpressure	−0.0239	0.9764	0.0152	0.1164	0.9477	1.0059
BMI	−0.0298	0.9706	0.0504	0.5545	0.8792	1.0715
physical activity	0.4796	1.6155	0.2481	0.0532	0.9933	2.6274
waist circumference	−0.0176	0.9826	0.0208	0.3984	0.9432	1.0235
coronary artery calcification	0.3148	1.3700	0.0605	< 0.0001	1.2168	1.5426

f1)

	coef	HR	se	p	lower	upper
rs6772228	−1.1074	0.3304	0.5867	0.0591	0.1046	1.0435
sex	0.2352	1.2651	0.2803	0.4014	0.7304	2.1915
age	0.0687	1.0711	0.0166	< 0.0001	1.0369	1.1065
HDL	−0.0033	0.9967	0.0089	0.7109	0.9795	1.0142
LDL	−0.0038	0.9962	0.0049	0.4438	0.9866	1.0059
triglycerides	0.0011	1.0011	0.0013	0.428	0.9984	1.0037
diabetes	0.1407	1.1511	0.1532	0.3584	0.8525	1.5544
smoking	−0.0384	0.9623	0.1357	0.7769	0.7376	1.2554
systolic bloodpressure	0.0028	1.0028	0.0069	0.6848	0.9894	1.0164
diastolic bloodpressure	−0.0045	0.9955	0.0135	0.7396	0.9696	1.0222
CRP	−0.0695	0.9329	0.1343	0.605	0.7170	1.2138
BMI	−0.0952	0.9092	0.0450	0.0341	0.8325	0.9929
physical activity	0.2818	1.3255	0.1956	0.1496	0.9035	1.9446
waist circumference	0.0288	1.0292	0.0163	0.0763	0.9970	1.0626
coronary artery calcification	0.2898	1.3361	0.0487	< 0.0001	1.2145	1.4699

f2)

	coef	HR	se	p	lower	upper
rs6772228	0.1675	1.1823	0.2311	0.4687	0.7516	1.8597
sex	−0.3238	0.7234	0.1826	0.0762	0.5058	1.0347
age	0.0565	1.0581	0.0097	< 0.0001	1.0381	1.0785
HDL	−0.0060	0.9940	0.0045	0.1839	0.9853	1.0028
LDL	−0.0029	0.9971	0.0020	0.1467	0.9932	1.0010
triglycerides	0.0008	1.0008	0.0005	0.1054	0.9998	1.0017
diabetes	0.1639	1.1781	0.0975	0.0928	0.9732	1.4261
smoking	0.2300	1.2586	0.0851	0.0069	1.0652	1.4870
systolic bloodpressure	0.0069	1.0069	0.0040	0.087	0.9990	1.0149
diastolic bloodpressure	−0.0055	0.9946	0.0078	0.4866	0.9794	1.0100
CRP	−0.0042	0.9958	0.0921	0.9634	0.8313	1.1928
BMI	0.0416	1.0425	0.0265	0.1164	0.9897	1.0981
physical activity	0.0977	1.1027	0.1196	0.4138	0.8723	1.3939
waist circumference	−0.0188	0.9814	0.0105	0.0738	0.9613	1.0018
coronary artery calcification	0.2801	1.3233	0.0305	< 0.0001	1.2465	1.4048

g1)

	coef	HR	se	p	lower	upper
rs6772228	−0.6171	0.5395	0.5908	0.2962	0.1695	1.7174
sex	−0.0483	0.9529	0.3075	0.8753	0.5215	1.7410
age	0.0756	1.0786	0.0182	< 0.0001	1.0409	1.1177
total cholesterol	−0.0016	0.9984	0.0045	0.7195	0.9897	1.0072
HDL	−0.0001	0.9999	0.0058	0.9804	0.9885	1.0114
triglycerides	0.0017	1.0017	0.0007	0.0131	1.0004	1.0031
diabetes	0.1412	1.1516	0.1721	0.4119	0.8220	1.6136
smoking	0.1481	1.1596	0.1489	0.32	0.8661	1.5526
systolic bloodpressure	0.0013	1.0013	0.0075	0.8613	0.9866	1.0162
diastolic bloodpressure	−0.0063	0.9938	0.0144	0.6632	0.9661	1.0222
CRP	−0.3883	0.6782	0.2967	0.1906	0.3792	1.2131
BMI	−0.0506	0.9507	0.0495	0.3074	0.8627	1.0476
physical activity	0.5433	1.7217	0.2156	0.0117	1.1284	2.6270
waist circumference	0.0190	1.0192	0.0178	0.2846	0.9843	1.0554
coronary artery calcification	0.2567	1.2926	0.0528	< 0.0001	1.1656	1.4334

g2)

	coef	HR	se	p	lower	upper
rs6772228	−0.0031	0.9969	0.2305	0.9892	0.6346	1.5660
sex	−0.1920	0.8253	0.1764	0.2765	0.5841	1.1662
age	0.0551	1.0567	0.0095	< 0.0001	1.0373	1.0764
total cholesterol	−0.0034	0.9966	0.0019	0.0792	0.9929	1.0004
HDL	−0.0038	0.9962	0.0048	0.4264	0.9869	1.0056
triglycerides	0.0011	1.0011	0.0007	0.1285	0.9997	1.0024
diabetes	0.1669	1.1817	0.0941	0.0761	0.9826	1.4210
smoking	0.1659	1.1805	0.0822	0.0435	1.0049	1.3868
systolic bloodpressure	0.0070	1.0070	0.0039	0.0763	0.9993	1.0148
diastolic bloodpressure	−0.0038	0.9962	0.0077	0.6195	0.9813	1.0113
CRP	0.0672	1.0695	0.0876	0.4427	0.9009	1.2698
BMI	0.0192	1.0194	0.0258	0.4571	0.9690	1.0724
physical activity	0.0455	1.0465	0.1151	0.693	0.8351	1.3115
waist circumference	−0.0136	0.9865	0.0102	0.1817	0.9670	1.0064
coronary artery calcification	0.2876	1.3332	0.0297	< 0.0001	1.2579	1.4130

h1)

	coef	HR	se	p	lower	upper
rs6772228	0.1472	1.1586	0.2272	0.5169	0.7423	1.8084
sex	−0.1795	0.8357	0.1782	0.3139	0.5893	1.1851
age	0.0711	1.0737	0.0101	< 0.0001	1.0527	1.0951
total cholesterol	−0.0017	0.9983	0.0036	0.6341	0.9914	1.0053
LDL	−0.0008	0.9992	0.0037	0.8296	0.9920	1.0064
triglycerides	0.0001	1.0001	0.0008	0.8768	0.9985	1.0017
diabetes	0.1966	1.2172	0.1004	0.0502	0.9999	1.4818
smoking	0.2148	1.2396	0.0854	0.0119	1.0486	1.4655
systolic bloodpressure	0.0085	1.0085	0.0040	0.0326	1.0007	1.0164
diastolic bloodpressure	−0.0025	0.9975	0.0078	0.7497	0.9824	1.0129
CRP	−0.0868	0.9168	0.1042	0.4049	0.7474	1.1247
BMI	0.0241	1.0244	0.0273	0.3764	0.9711	1.0807
physical activity	0.1808	1.1982	0.1173	0.1233	0.9521	1.5079
waist circumference	−0.0059	0.9942	0.0107	0.5845	0.9735	1.0153
coronary artery calcification	0.2574	1.2936	0.0290	< 0.0001	1.2220	1.3693

h2)

	coef	HR	se	p	lower	upper
rs6772228	−1.4353	0.2380	0.7155	0.0449	0.0586	0.9676
sex	−0.2621	0.7694	0.2888	0.3641	0.4369	1.3552
age	0.0285	1.0289	0.0152	0.0605	0.9987	1.0599
total cholesterol	−0.0048	0.9953	0.0060	0.427	0.9836	1.0070
LDL	0.0003	1.0003	0.0065	0.9648	0.9876	1.0131
triglycerides	0.0020	1.0020	0.0009	0.019	1.0003	1.0037
diabetes	0.0625	1.0645	0.1440	0.6644	0.8027	1.4116
smoking	0.0079	1.0079	0.1362	0.9538	0.7718	1.3163
systolic bloodpressure	−0.0010	0.9990	0.0071	0.8834	0.9852	1.0129
diastolic bloodpressure	−0.0077	0.9923	0.0137	0.5735	0.9660	1.0193
CRP	0.1284	1.1370	0.1229	0.296	0.8937	1.4467
BMI	−0.0480	0.9531	0.0440	0.2758	0.8743	1.0391
physical activity	0.0472	1.0483	0.1958	0.8097	0.7142	1.5387
waist circumference	−0.0110	0.9891	0.0166	0.5082	0.9575	1.0217
coronary artery calcification	0.3528	1.4231	0.0540	< 0.0001	1.2801	1.5820

i1)

	coef	HR	se	p	lower	upper
rs6772228	−0.3185	0.7272	0.3410	0.3503	0.3727	1.4189
sex	−0.0986	0.9061	0.2061	0.6323	0.6050	1.3570
age	0.0735	1.0763	0.0117	< 0.0001	1.0519	1.1013
total cholesterol	−0.0023	0.9977	0.0051	0.6515	0.9877	1.0077
HDL	−0.0014	0.9986	0.0060	0.8145	0.9870	1.0104
LDL	−0.0026	0.9974	0.0053	0.623	0.9872	1.0078
diabetes	0.0745	1.0774	0.1277	0.5594	0.8389	1.3837
smoking	0.2635	1.3014	0.0971	0.0066	1.0759	1.5742
systolic bloodpressure	0.0041	1.0042	0.0047	0.3816	0.9949	1.0135
diastolic bloodpressure	0.0125	1.0126	0.0093	0.1789	0.9943	1.0312
CRP	−0.0564	0.9452	0.0917	0.5385	0.7898	1.1312
BMI	0.0253	1.0256	0.0319	0.4274	0.9635	1.0917
physical activity	0.3249	1.3839	0.1368	0.0176	1.0584	1.8095
waist circumference	−0.0125	0.9876	0.0122	0.3039	0.9643	1.0114
coronary artery calcification	0.2814	1.3249	0.0340	< 0.0001	1.2395	1.4162

i2)

	coef	HR	se	p	lower	upper
rs6772228	0.0530	1.0545	0.2753	0.8473	0.6147	1.8089
sex	−0.2138	0.8075	0.2325	0.3578	0.5119	1.2737
age	0.0428	1.0438	0.0119	0.0003	1.0197	1.0684
total cholesterol	0.0022	1.0022	0.0035	0.5236	0.9954	1.0090
HDL	−0.0161	0.9841	0.0072	0.0254	0.9703	0.9980
LDL	−0.0036	0.9964	0.0036	0.317	0.9893	1.0035
diabetes	0.2431	1.2752	0.1088	0.0254	1.0304	1.5782
smoking	0.0439	1.0449	0.1082	0.6851	0.8451	1.2918
systolic bloodpressure	0.0093	1.0093	0.0050	0.0627	0.9995	1.0193
diastolic bloodpressure	−0.0248	0.9755	0.0101	0.0135	0.9564	0.9949
CRP	0.0768	1.0798	0.1412	0.5865	0.8188	1.4239
BMI	−0.0263	0.9740	0.0341	0.4401	0.9110	1.0414
physical activity	−0.1239	0.8835	0.1489	0.4055	0.6598	1.1830
waist circumference	0.0034	1.0034	0.0133	0.7996	0.9776	1.0299
coronary artery calcification	0.2762	1.3181	0.0394	< 0.0001	1.2202	1.4238

j1)

	coef	HR	se	p	lower	upper
rs6772228	−0.4654	0.6279	0.5753	0.4185	0.2033	1.9390
sex	−0.1097	0.8961	0.3652	0.7639	0.4381	1.8331
age	0.0551	1.0566	0.0192	0.0042	1.0175	1.0973
total cholesterol	0.0116	1.0116	0.0112	0.3031	0.9896	1.0342
HDL	−0.0224	0.9778	0.0137	0.1007	0.9520	1.0044
LDL	−0.0129	0.9872	0.0108	0.2317	0.9666	1.0083
triglycerides	0.0032	1.0032	0.0025	0.1961	0.9983	1.0081
diabetes	−0.0507	0.9505	0.2265	0.8228	0.6097	1.4818
smoking	0.1946	1.2149	0.1809	0.282	0.8522	1.7319
CRP	0.1193	1.1267	0.1488	0.4228	0.8417	1.5083
BMI	−0.0032	0.9968	0.0618	0.9592	0.8831	1.1253
physical activity	0.2339	1.2635	0.2718	0.3896	0.7416	2.1526
waist circumference	−0.0145	0.9856	0.0244	0.5526	0.9397	1.0338
coronary artery calcification	0.3728	1.4518	0.0642	< 0.0001	1.2801	1.6464

j2)

	coef	HR	se	p	lower	upper
rs6772228	−0.1425	0.8672	0.3467	0.6811	0.4396	1.7109
sex	−0.6018	0.5478	0.2721	0.027	0.3214	0.9338
age	0.0700	1.0726	0.0130	< 0.0001	1.0456	1.1002
total cholesterol	0.0010	1.0010	0.0057	0.8586	0.9899	1.0122
HDL	−0.0096	0.9904	0.0086	0.2637	0.9739	1.0073
LDL	−0.0034	0.9966	0.0057	0.558	0.9855	1.0079
triglycerides	0.0004	1.0004	0.0012	0.7176	0.9981	1.0028
diabetes	0.2762	1.3181	0.1346	0.0403	1.0123	1.7161
smoking	0.2054	1.2280	0.1208	0.0891	0.9691	1.5560
CRP	0.0343	1.0349	0.1921	0.8583	0.7102	1.5080
BMI	0.0617	1.0637	0.0376	0.1011	0.9880	1.1451
physical activity	0.2370	1.2675	0.1694	0.1617	0.9094	1.7666
waist circumference	−0.0314	0.9691	0.0150	0.0361	0.9410	0.9980
coronary artery calcification	0.2151	1.2400	0.0400	< 0.0001	1.1465	1.3411

j3)

	coef	HR	se	p	lower	upper
rs6772228	−0.0604	0.9414	0.3114	0.8462	0.5113	1.7332
sex	0.0963	1.1011	0.2141	0.6528	0.7237	1.6753
age	0.0605	1.0623	0.0117	< 0.0001	1.0382	1.0871
total cholesterol	−0.0067	0.9934	0.0044	0.1344	0.9847	1.0021
HDL	0.0022	1.0022	0.0021	0.2936	0.9981	1.0062
LDL	0.0020	1.0020	0.0048	0.673	0.9927	1.0114
triglycerides	0.0013	1.0013	0.0007	0.0504	1.0000	1.0027
diabetes	0.1534	1.1658	0.1157	0.1848	0.9293	1.4625
smoking	0.1342	1.1437	0.1049	0.2007	0.9311	1.4047
CRP	−0.0740	0.9286	0.1026	0.4706	0.7594	1.1355
BMI	−0.0308	0.9696	0.0342	0.3667	0.9069	1.0368
physical activity	0.0220	1.0222	0.1438	0.8784	0.7712	1.3551
waist circumference	0.0090	1.0091	0.0129	0.4822	0.9840	1.0348
coronary artery calcification	0.3063	1.3584	0.0390	< 0.0001	1.2585	1.4662

k1)

	coef	HR	se	p	lower	upper
rs6772228	−0.1730	0.8411	0.2488	0.4868	0.5165	1.3698
sex	−0.1098	0.8960	0.1754	0.5314	0.6354	1.2636
age	0.0642	1.0664	0.0097	< 0.0001	1.0464	1.0867
total cholesterol	−0.0011	0.9989	0.0044	0.8061	0.9904	1.0075
HDL	−0.0065	0.9935	0.0060	0.2764	0.9818	1.0053
LDL	−0.0017	0.9983	0.0044	0.7062	0.9898	1.0070
triglycerides	0.0005	1.0005	0.0009	0.5829	0.9987	1.0022
smoking	0.2417	1.2734	0.0816	0.0031	1.0851	1.4943
systolic bloodpressure	0.0038	1.0038	0.0042	0.3685	0.9955	1.0122
diastolic bloodpressure	0.0025	1.0025	0.0082	0.7616	0.9865	1.0188
CRP	−0.1428	0.8669	0.1231	0.2458	0.6811	1.1034
BMI	0.0239	1.0241	0.0273	0.383	0.9707	1.0805
physical activity	0.1866	1.2051	0.1167	0.1099	0.9587	1.5148
waist circumference	−0.0072	0.9928	0.0105	0.4922	0.9727	1.0134
coronary artery calcification	0.2707	1.3109	0.0289	< 0.0001	1.2387	1.3873

k2)

	coef	HR	se	p	lower	upper
rs6772228	0.2311	1.2600	0.4281	0.5892	0.5445	2.9156
sex	−0.5336	0.5865	0.3324	0.1084	0.3057	1.1251
age	0.0470	1.0481	0.0170	0.0058	1.0137	1.0837
total cholesterol	−0.0050	0.9950	0.0094	0.5902	0.9768	1.0134
HDL	0.0074	1.0074	0.0104	0.478	0.9871	1.0281
LDL	0.0023	1.0023	0.0094	0.804	0.9840	1.0211
triglycerides	0.0021	1.0021	0.0012	0.0769	0.9998	1.0044
smoking	−0.0827	0.9206	0.1623	0.6104	0.6698	1.2654
systolic bloodpressure	0.0090	1.0090	0.0062	0.148	0.9968	1.0214
diastolic bloodpressure	−0.0154	0.9848	0.0121	0.2045	0.9617	1.0084
CRP	0.1138	1.1205	0.0879	0.1957	0.9431	1.3313
BMI	−0.0262	0.9742	0.0467	0.5749	0.8890	1.0675
physical activity	−0.0895	0.9144	0.2057	0.6635	0.6111	1.3683
waist circumference	−0.0113	0.9888	0.0182	0.5344	0.9541	1.0247
coronary artery calcification	0.3219	1.3797	0.0581	< 0.0001	1.2312	1.5462

I1)

	coef	HR	se	p	lower	upper
rs6772228	−0.5630	0.5695	0.4197	0.1798	0.2501	1.2964
sex	−0.1579	0.8540	0.2373	0.5059	0.5363	1.3597
age	0.0873	1.0913	0.0157	< 0.0001	1.0583	1.1253
total cholesterol	−0.0055	0.9945	0.0069	0.426	0.9811	1.0081
HDL	−0.0091	0.9909	0.0092	0.319	0.9733	1.0089
LDL	0.0002	1.0002	0.0070	0.9736	0.9866	1.0140
triglycerides	0.0016	1.0016	0.0016	0.2985	0.9985	1.0047
diabetes	0.3420	1.4077	0.1452	0.0185	1.0591	1.8711
systolic bloodpressure	0.0081	1.0082	0.0060	0.1775	0.9963	1.0202
diastolic bloodpressure	−0.0065	0.9935	0.0120	0.5885	0.9704	1.0172
CRP	0.0149	1.0150	0.1074	0.8895	0.8223	1.2529
BMI	−0.0224	0.9778	0.0358	0.5308	0.9116	1.0488
physical activity	0.0458	1.0468	0.1741	0.7926	0.7442	1.4727
waist circumference	0.0013	1.0013	0.0142	0.9258	0.9739	1.0296
coronary artery calcification	0.2603	1.2973	0.0415	< 0.0001	1.1960	1.4072

l2)

	coef	HR	se	p	lower	upper
rs6772228	0.2030	1.2251	0.2981	0.4959	0.6830	2.1973
sex	−0.5163	0.5967	0.2841	0.0692	0.3419	1.0413
age	0.0534	1.0548	0.0127	< 0.0001	1.0289	1.0815
total cholesterol	−0.0008	0.9992	0.0067	0.9035	0.9861	1.0125
HDL	0.0034	1.0034	0.0082	0.6767	0.9874	1.0197
LDL	−0.0010	0.9990	0.0067	0.8771	0.9860	1.0121
triglycerides	0.0012	1.0012	0.0011	0.2547	0.9991	1.0033
diabetes	0.1014	1.1067	0.1224	0.4073	0.8707	1.4067
systolic bloodpressure	0.0061	1.0061	0.0051	0.2311	0.9961	1.0162
diastolic bloodpressure	−0.0064	0.9936	0.0100	0.5225	0.9743	1.0133
CRP	−0.0612	0.9406	0.1614	0.7043	0.6856	1.2905
BMI	0.0630	1.0650	0.0405	0.12	0.9837	1.1529
physical activity	0.2440	1.2763	0.1563	0.1185	0.9396	1.7338
waist circumference	−0.0200	0.9802	0.0150	0.1846	0.9518	1.0096
coronary artery calcification	0.2736	1.3147	0.0405	< 0.0001	1.2143	1.4235

I3)

	coef	HR	se	p	lower	upper
rs6772228	−0.0539	0.9475	0.4656	0.9078	0.3805	2.3598
sex	0.0913	1.0956	0.3109	0.7691	0.5957	2.0151
age	0.0396	1.0404	0.0173	0.0223	1.0057	1.0764
total cholesterol	−0.0028	0.9972	0.0068	0.6781	0.9839	1.0106
HDL	−0.0019	0.9981	0.0105	0.8536	0.9777	1.0189
LDL	0.0015	1.0015	0.0071	0.8338	0.9877	1.0155
triglycerides	0.0011	1.0012	0.0011	0.2969	0.9990	1.0033
diabetes	0.0914	1.0957	0.1796	0.6109	0.7705	1.5581
systolic bloodpressure	0.0037	1.0037	0.0081	0.652	0.9878	1.0198
diastolic bloodpressure	−0.0058	0.9942	0.0148	0.6952	0.9657	1.0235
CRP	−0.0561	0.9454	0.1389	0.6862	0.7201	1.2413
BMI	−0.0200	0.9802	0.0499	0.6877	0.8889	1.0808
physical activity	0.0198	1.0200	0.2135	0.926	0.6712	1.5501
waist circumference	0.0033	1.0033	0.0197	0.8654	0.9654	1.0428
coronary artery calcification	0.3269	1.3866	0.0540	< 0.0001	1.2475	1.5413

m1)

	coef	HR	se	p	lower	upper
rs6772228	−0.1790	0.8361	0.4641	0.6998	0.3367	2.0766
sex	0.1438	1.1547	0.2917	0.622	0.6519	2.0453
age	0.0428	1.0437	0.0172	0.013	1.0091	1.0796
total cholesterol	0.0073	1.0074	0.0080	0.3592	0.9917	1.0233
HDL	−0.0084	0.9916	0.0102	0.4059	0.9720	1.0115
LDL	−0.0084	0.9917	0.0081	0.303	0.9760	1.0076
triglycerides	0.0004	1.0004	0.0013	0.7386	0.9978	1.0031
diabetes	0.3687	1.4458	0.1924	0.0554	0.9916	2.1081
smoking	0.1235	1.1315	0.1458	0.3968	0.8503	1.5056
systolic bloodpressure	0.0125	1.0126	0.0072	0.0827	0.9984	1.0271
diastolic bloodpressure	−0.0121	0.9880	0.0160	0.4491	0.9575	1.0194
CRP	0.1716	1.1872	0.1531	0.2623	0.8794	1.6028
BMI	−0.0112	0.9889	0.0512	0.8272	0.8944	1.0934
physical activity	0.5130	1.6703	0.2201	0.0198	1.0849	2.5715
coronary artery calcification	0.3172	1.3733	0.0532	< 0.0001	1.2373	1.5244

m2)

	coef	HR	se	p	lower	upper
rs6772228	−0.0612	0.9407	0.2356	0.7951	0.5928	1.4927
sex	−0.2012	0.8177	0.1428	0.1587	0.6181	1.0818
age	0.0619	1.0638	0.0094	< 0.0001	1.0444	1.0836
total cholesterol	−0.0051	0.9949	0.0044	0.2439	0.9865	1.0035
HDL	−0.0027	0.9973	0.0059	0.649	0.9859	1.0089
LDL	0.0012	1.0012	0.0044	0.7789	0.9926	1.0100
triglycerides	0.0014	1.0014	0.0007	0.0669	0.9999	1.0028
diabetes	0.1249	1.1330	0.0863	0.148	0.9567	1.3419
smoking	0.1297	1.1385	0.0830	0.1181	0.9676	1.3395
systolic bloodpressure	0.0046	1.0047	0.0039	0.2282	0.9971	1.0123
diastolic bloodpressure	−0.0004	0.9996	0.0074	0.954	0.9852	1.0142
CRP	−0.0614	0.9404	0.0909	0.499	0.7870	1.1237
BMI	0.0052	1.0053	0.0146	0.7199	0.9768	1.0345
physical activity	0.0159	1.0160	0.1131	0.8883	0.8139	1.2683
coronary artery calcification	0.2654	1.3039	0.0292	< 0.0001	1.2313	1.3808

n1)

	coef	HR	se	p	lower	upper
rs6772228	−0.5724	0.5642	0.5957	0.3365	0.1755	1.8131
sex	−0.0114	0.9887	0.3526	0.9742	0.4954	1.9731
age	0.0667	1.0690	0.0198	0.0008	1.0282	1.1114
total cholesterol	0.0108	1.0109	0.0083	0.193	0.9945	1.0275
HDL	−0.0197	0.9805	0.0107	0.0652	0.9602	1.0012
LDL	−0.0136	0.9865	0.0083	0.1019	0.9706	1.0027
triglycerides	−0.0005	0.9995	0.0015	0.7634	0.9966	1.0025
diabetes	0.3341	1.3966	0.2420	0.1674	0.8692	2.2443
smoking	0.1998	1.2211	0.1526	0.1905	0.9054	1.6469
systolic bloodpressure	0.0032	1.0032	0.0081	0.6907	0.9874	1.0193
diastolic bloodpressure	−0.0020	0.9981	0.0172	0.9098	0.9649	1.0323
CRP	−0.0100	0.9900	0.1274	0.9372	0.7712	1.2709
physical activity	0.6471	1.9100	0.2444	0.0081	1.1831	3.0833
waist circumference	−0.0290	0.9715	0.0194	0.1355	0.9352	1.0091
coronary artery calcification	0.4063	1.5013	0.0629	< 0.0001	1.3272	1.6983

n2)

	coef	HR	se	p	lower	upper
rs6772228	−0.0648	0.9372	0.2298	0.7778	0.5974	1.4704
sex	−0.2203	0.8023	0.1435	0.1248	0.6055	1.0629
age	0.0575	1.0592	0.0091	< 0.0001	1.0404	1.0783
total cholesterol	−0.0058	0.9942	0.0043	0.1717	0.9859	1.0025
HDL	0.0004	1.0004	0.0057	0.9393	0.9893	1.0117
LDL	0.0023	1.0023	0.0043	0.5899	0.9939	1.0108
triglycerides	0.0016	1.0016	0.0007	0.0228	1.0002	1.0030
diabetes	0.1405	1.1508	0.0848	0.0975	0.9746	1.3589
smoking	0.1419	1.1525	0.0806	0.0782	0.9841	1.3497
systolic bloodpressure	0.0059	1.0059	0.0038	0.1203	0.9985	1.0135
diastolic bloodpressure	−0.0052	0.9948	0.0073	0.4811	0.9807	1.0092
CRP	−0.0190	0.9812	0.0904	0.8339	0.8218	1.1715
physical activity	0.0171	1.0172	0.1111	0.8778	0.8182	1.2646
waist circumference	−0.0010	0.9990	0.0058	0.8573	0.9876	1.0104
coronary artery calcification	0.2533	1.2883	0.0280	< 0.0001	1.2196	1.3609

S4C Table. Results of Cox regression models for rs7675998

coef: coefficient, HR: Hazard Ratio, se: Standard error,
lower/upper: lower/upper boundarie of the 95% confidence interval
a) crude, b) adjusted, c1) young age, c2) older age, d1) male, d2) female,
e1) low hsCRP, e2) intermediate hsCRP, e3) high hsCRP, f1) low total cholesterol,
f2) high total cholesterol, g1) low LDL, g2) high LDL, h1) normal HDL, h2) high HDL,
i1) low triglycerides, i2) high triglycerides, j1) ideal blood pressure,
j2) normal/high normal blood pressure, j3) hypertension, k1) no diabetes, k2) diabetes,
l1) never smoker, l2) former smoker, l3) current smoker, m1) normal waist circumference,
m2) high waist circumference, n1) normal BMI, n2) high BMI

a)

	coef	HR	se	p	lower	upper
rs7675998	0.0668	1.0691	0.0803	0.4053	0.9134	1.2512

b)

	coef	HR	se	p	lower	upper
rs7675998	0.0838	1.0874	0.0847	0.3227	0.9210	1.2838
sex	−0.1097	0.8961	0.1498	0.4641	0.6681	1.2020
age	0.0592	1.0610	0.0081	< 0.0001	1.0442	1.0781
total cholesterol	−0.0019	0.9981	0.0038	0.6159	0.9908	1.0055
HDL	−0.0037	0.9963	0.0050	0.4528	0.9866	1.0060
LDL	−0.0012	0.9988	0.0038	0.761	0.9913	1.0064
triglycerides	0.0011	1.0011	0.0006	0.0745	0.9999	1.0024
diabetes	0.1671	1.1819	0.0800	0.0368	1.0103	1.3826
smoking	0.1891	1.2082	0.0702	0.007	1.0530	1.3863
systolic bloodpressure	0.0070	1.0070	0.0034	0.0399	1.0003	1.0137
diastolic bloodpressure	−0.0052	0.9948	0.0066	0.4248	0.9820	1.0077
CRP	0.0312	1.0317	0.0791	0.6935	0.8835	1.2047
BMI	0.0032	1.0032	0.0224	0.8869	0.9601	1.0482
physical activity	0.1624	1.1763	0.0993	0.1022	0.9682	1.4292
waist circumference	−0.0078	0.9923	0.0088	0.3775	0.9753	1.0095
coronary artery calcification	0.2740	1.3152	0.0251	< 0.0001	1.2519	1.3816

c1)

	coef	HR	se	p	lower	upper
rs7675998	0.1971	1.2178	0.1739	0.257	0.8661	1.7123
sex	−0.1109	0.8951	0.2873	0.6996	0.5097	1.5717
total cholesterol	0.0025	1.0025	0.0067	0.7111	0.9895	1.0156
HDL	−0.0140	0.9861	0.0102	0.1687	0.9665	1.0060
LDL	−0.0016	0.9984	0.0068	0.8129	0.9852	1.0118
triglycerides	0.0003	1.0003	0.0013	0.8301	0.9977	1.0029
diabetes	−0.0013	0.9987	0.1978	0.9949	0.6778	1.4716
smoking	0.2803	1.3235	0.1354	0.0384	1.0150	1.7257
systolic bloodpressure	−0.0044	0.9957	0.0089	0.6266	0.9784	1.0133
diastolic bloodpressure	0.0187	1.0188	0.0163	0.2515	0.9869	1.0519
CRP	0.0792	1.0824	0.1806	0.661	0.7597	1.5423
BMI	0.0081	1.0081	0.0466	0.8617	0.9202	1.1044
physical activity	0.2321	1.2612	0.2156	0.2818	0.8265	1.9247
waist circumference	−0.0130	0.9871	0.0183	0.4762	0.9524	1.0230
coronary artery calcification	0.2974	1.3463	0.0466	< 0.0001	1.2287	1.4752

c2)

	coef	HR	se	p	lower	upper
rs7675998	0.0701	1.0726	0.0972	0.4712	0.8865	1.2978
sex	0.3514	1.4211	0.1756	0.0453	1.0073	2.0048
total cholesterol	−0.0041	0.9959	0.0041	0.3129	0.9880	1.0039
HDL	0.0000	1.0000	0.0042	0.999	0.9918	1.0083
LDL	−0.0003	0.9997	0.0042	0.951	0.9915	1.0080
triglycerides	0.0012	1.0012	0.0007	0.0723	0.9999	1.0025
diabetes	0.1959	1.2164	0.0886	0.027	1.0225	1.4472
smoking	0.0989	1.1040	0.0827	0.2317	0.9388	1.2983
systolic bloodpressure	0.0137	1.0138	0.0036	0.0001	1.0067	1.0210
diastolic bloodpressure	−0.0186	0.9816	0.0071	0.0086	0.9681	0.9953
CRP	0.0306	1.0311	0.0812	0.706	0.8795	1.2088
BMI	−0.0114	0.9886	0.0261	0.6614	0.9394	1.0405
physical activity	0.1365	1.1462	0.1125	0.2251	0.9194	1.4291
waist circumference	−0.0010	0.9990	0.0101	0.9199	0.9794	1.0190
coronary artery calcification	0.2837	1.3280	0.0296	< 0.0001	1.2532	1.4072

d1)

	coef	HR	se	p	lower	upper
rs7675998	0.0402	1.0410	0.1045	0.7005	0.8482	1.2777
age	0.0429	1.0438	0.0097	< 0.0001	1.0242	1.0638
total cholesterol	−0.0059	0.9941	0.0048	0.2211	0.9848	1.0036
HDL	0.0029	1.0029	0.0062	0.6385	0.9908	1.0151
LDL	0.0026	1.0026	0.0049	0.5912	0.9931	1.0123
triglycerides	0.0015	1.0015	0.0008	0.0417	1.0001	1.0030
diabetes	0.1874	1.2061	0.0912	0.04	1.0086	1.4421
smoking	0.1277	1.1362	0.0901	0.1564	0.9523	1.3557
systolic bloodpressure	0.0048	1.0048	0.0043	0.2615	0.9964	1.0133
diastolic bloodpressure	−0.0029	0.9971	0.0080	0.7202	0.9816	1.0129
CRP	0.0433	1.0442	0.0904	0.6319	0.8747	1.2466
BMI	0.0329	1.0335	0.0294	0.2629	0.9756	1.0948
physical activity	0.1590	1.1723	0.1223	0.1938	0.9224	1.4900
waist circumference	−0.0121	0.9879	0.0113	0.2847	0.9662	1.0101
coronary artery calcification	0.2876	1.3332	0.0333	< 0.0001	1.2490	1.4230

d2)

	coef	HR	se	p	lower	upper
rs7675998	0.1877	1.2065	0.1477	0.2037	0.9033	1.6114
age	0.0994	1.1045	0.0154	< 0.0001	1.0717	1.1383
total cholesterol	0.0021	1.0021	0.0054	0.694	0.9916	1.0128
HDL	−0.0135	0.9866	0.0081	0.0948	0.9711	1.0023
LDL	−0.0057	0.9943	0.0055	0.3017	0.9836	1.0051
triglycerides	0.0013	1.0013	0.0013	0.3494	0.9986	1.0039
diabetes	0.1146	1.1214	0.1713	0.5036	0.8015	1.5689
smoking	0.3235	1.3819	0.1107	0.0035	1.1123	1.7169
systolic bloodpressure	0.0089	1.0090	0.0057	0.1142	0.9978	1.0202
diastolic bloodpressure	−0.0063	0.9937	0.0118	0.5943	0.9711	1.0170
CRP	0.0113	1.0114	0.1533	0.9413	0.7489	1.3658
BMI	−0.0292	0.9712	0.0353	0.4086	0.9063	1.0408
physical activity	0.1595	1.1729	0.1723	0.3546	0.8368	1.6441
waist circumference	−0.0066	0.9934	0.0143	0.6428	0.9660	1.0216
coronary artery calcification	0.2450	1.2777	0.0390	< 0.0001	1.1835	1.3793

e1)

	coef	HR	se	p	lower	upper
rs7675998	0.0873	1.0912	0.1062	0.411	0.8862	1.3437
sex	−0.1662	0.8469	0.1829	0.3635	0.5917	1.2120
age	0.0672	1.0695	0.0098	< 0.0001	1.0491	1.0903
total cholesterol	0.0004	1.0004	0.0043	0.9276	0.9920	1.0088
HDL	−0.0066	0.9935	0.0058	0.2575	0.9822	1.0048
LDL	−0.0041	0.9959	0.0044	0.3521	0.9874	1.0045
triglycerides	0.0011	1.0011	0.0007	0.1383	0.9997	1.0025
diabetes	0.1529	1.1652	0.1011	0.1304	0.9558	1.4206
smoking	0.1950	1.2153	0.0868	0.0247	1.0251	1.4408
systolic bloodpressure	0.0076	1.0077	0.0043	0.0754	0.9992	1.0162
diastolic bloodpressure	−0.0052	0.9948	0.0084	0.5332	0.9786	1.0112
BMI	0.0401	1.0409	0.0284	0.1578	0.9846	1.1005
physical activity	0.1720	1.1876	0.1196	0.1504	0.9395	1.5013
waist circumference	−0.0172	0.9830	0.0106	0.1052	0.9628	1.0036
coronary artery calcification	0.2686	1.3082	0.0303	< 0.0001	1.2328	1.3881

e2)

	coef	HR	se	p	lower	upper
rs7675998	0.3232	1.3815	0.2317	0.1631	0.8772	2.1758
sex	0.7294	2.0739	0.4531	0.1074	0.8533	5.0405
age	0.0988	1.1039	0.0251	0.0001	1.0509	1.1595
total cholesterol	−0.0102	0.9899	0.0135	0.4514	0.9641	1.0164
HDL	−0.0033	0.9967	0.0171	0.8471	0.964	1.0306
LDL	0.0105	1.0105	0.0127	0.412	0.9856	1.0361
triglycerides	−0.0032	0.9968	0.0026	0.2312	0.9917	1.002
diabetes	0.3373	1.4012	0.1944	0.0827	0.9572	2.0511
smoking	0.1917	1.2113	0.2124	0.3668	0.7988	1.8369
systolic bloodpressure	−0.0016	0.9984	0.0095	0.8676	0.98	1.0172
diastolic bloodpressure	0.0098	1.0098	0.0175	0.5753	0.9758	1.045
BMI	−0.1475	0.8629	0.0731	0.0437	0.7477	0.9958
physical activity	−0.5839	0.5577	0.2848	0.0403	0.3192	0.9745
waist circumference	0.0658	1.068	0.0293	0.0247	1.0084	1.1312
coronary artery calcification	0.2833	1.3275	0.0744	0.0001	1.1474	1.536

e3)

	coef	HR	se	p	lower	upper
rs7675998	−0.0345	0.9661	0.1861	0.8529	0.6708	1.3914
sex	−0.3274	0.7208	0.3554	0.3569	0.3592	1.4464
age	0.0087	1.0087	0.0187	0.6431	0.9724	1.0464
total cholesterol	−0.0024	0.9976	0.0067	0.7147	0.9846	1.0107
HDL	0.0010	1.0010	0.0031	0.752	0.9949	1.0071
LDL	0.0009	1.0009	0.0070	0.8981	0.9872	1.0147
triglycerides	0.0021	1.0021	0.0013	0.103	0.9996	1.0046
diabetes	0.1655	1.1800	0.1752	0.3449	0.8370	1.6634
smoking	0.1423	1.1529	0.1620	0.3799	0.8392	1.5838
systolic bloodpressure	0.0068	1.0068	0.0073	0.3501	0.9926	1.0212
diastolic bloodpressure	−0.0193	0.9809	0.0149	0.1952	0.9527	1.0099
BMI	−0.0336	0.9670	0.0482	0.4863	0.8798	1.0628
physical activity	0.5478	1.7295	0.2446	0.0251	1.0709	2.7933
waist circumference	−0.0138	0.9863	0.0203	0.4947	0.9479	1.0262
coronary artery calcification	0.3006	1.3506	0.0593	< 0.0001	1.2025	1.5170

f1)

	coef	HR	se	p	lower	upper
rs7675998	0.2497	1.2837	0.1567	0.111	0.9442	1.7452
sex	0.4018	1.4945	0.2747	0.1436	0.8723	2.5605
age	0.0749	1.0778	0.0164	< 0.0001	1.0437	1.1131
HDL	−0.0052	0.9948	0.0087	0.5462	0.9780	1.0118
LDL	−0.0043	0.9957	0.0048	0.3726	0.9864	1.0051
triglycerides	0.0007	1.0007	0.0013	0.6039	0.9981	1.0033
diabetes	0.2038	1.2260	0.1525	0.1816	0.9092	1.6532
smoking	−0.0082	0.9919	0.1354	0.952	0.7607	1.2934
systolic bloodpressure	0.0040	1.0040	0.0068	0.5559	0.9907	1.0175
diastolic bloodpressure	−0.0041	0.9959	0.0133	0.7566	0.9702	1.0222
CRP	−0.0558	0.9457	0.1293	0.6661	0.7340	1.2185
BMI	−0.1022	0.9028	0.0444	0.0212	0.8277	0.9849
physical activity	0.2351	1.2650	0.1951	0.2282	0.8631	1.8540
waist circumference	0.0298	1.0302	0.0163	0.0681	0.9978	1.0637
coronary artery calcification	0.3034	1.3544	0.0490	< 0.0001	1.2304	1.4909

f2)

	coef	HR	se	p	lower	upper
rs7675998	0.0121	1.0121	0.1021	0.906	0.8285	1.2364
sex	−0.2951	0.7444	0.1781	0.0974	0.5251	1.0554
age	0.0542	1.0557	0.0095	< 0.0001	1.0362	1.0755
HDL	−0.0050	0.9950	0.0044	0.262	0.9864	1.0037
LDL	−0.0028	0.9972	0.0020	0.1452	0.9933	1.0010
triglycerides	0.0008	1.0008	0.0005	0.093	0.9999	1.0017
diabetes	0.1310	1.1399	0.0954	0.1696	0.9456	1.3742
smoking	0.2647	1.3030	0.0830	0.0014	1.1075	1.5331
systolic bloodpressure	0.0077	1.0077	0.0040	0.0546	0.9998	1.0156
diastolic bloodpressure	−0.0070	0.9930	0.0077	0.3625	0.9782	1.0081
CRP	0.0974	1.1023	0.1079	0.3663	0.8923	1.3618
BMI	0.0417	1.0426	0.0257	0.1045	0.9914	1.0965
physical activity	0.1312	1.1402	0.1174	0.2638	0.9058	1.4352
waist circumference	−0.0202	0.9800	0.0102	0.0483	0.9605	0.9998
coronary artery calcification	0.2631	1.3010	0.0298	< 0.0001	1.2272	1.3791

g1)

	coef	HR	se	p	lower	upper
rs7675998	0.3693	1.4467	0.1610	0.0218	1.0552	1.9834
sex	0.1342	1.1437	0.2973	0.6516	0.6386	2.0482
age	0.0817	1.0852	0.0179	< 0.0001	1.0477	1.1240
total cholesterol	−0.0016	0.9984	0.0045	0.7291	0.9896	1.0073
HDL	−0.0010	0.9990	0.0073	0.8898	0.9849	1.0133
triglycerides	0.0017	1.0017	0.0007	0.0145	1.0003	1.0031
diabetes	0.2296	1.2580	0.1678	0.1713	0.9055	1.7479
smoking	0.1905	1.2098	0.1470	0.1952	0.9069	1.6139
systolic bloodpressure	0.0019	1.0019	0.0073	0.7934	0.9877	1.0163
diastolic bloodpressure	−0.0096	0.9904	0.0141	0.4944	0.9635	1.0181
CRP	−0.3245	0.7229	0.2563	0.2054	0.4374	1.1946
BMI	−0.0580	0.9436	0.0473	0.2195	0.8602	1.0352
physical activity	0.4706	1.6010	0.2142	0.028	1.0521	2.4363
waist circumference	0.0164	1.0165	0.0178	0.358	0.9816	1.0526
coronary artery calcification	0.2816	1.3252	0.0530	< 0.0001	1.1945	1.4703

g2)

	coef	HR	se	p	lower	upper
rs7675998	−0.0262	0.9741	0.1011	0.7956	0.7990	1.1877
sex	−0.1701	0.8436	0.1727	0.3247	0.6013	1.1834
age	0.0537	1.0552	0.0093	< 0.0001	1.0361	1.0745
total cholesterol	−0.0029	0.9971	0.0019	0.1254	0.9935	1.0008
HDL	−0.0039	0.9961	0.0047	0.4128	0.9870	1.0054
triglycerides	0.0010	1.0010	0.0007	0.1728	0.9996	1.0023
diabetes	0.1363	1.1461	0.0927	0.1412	0.9557	1.3743
smoking	0.2039	1.2262	0.0806	0.0114	1.0471	1.4359
systolic bloodpressure	0.0078	1.0078	0.0039	0.0458	1.0001	1.0155
diastolic bloodpressure	−0.0044	0.9956	0.0075	0.5559	0.9810	1.0104
CRP	0.0962	1.1009	0.0840	0.2522	0.9339	1.2979
BMI	0.0207	1.0209	0.0252	0.4113	0.9717	1.0727
physical activity	0.0853	1.0891	0.1135	0.4524	0.8718	1.3605
waist circumference	−0.0151	0.9850	0.0100	0.1303	0.9659	1.0045
coronary artery calcification	0.2727	1.3135	0.0291	< 0.0001	1.2407	1.3905

h1)

	coef	HR	se	p	lower	upper
rs7675998	0.0376	1.0384	0.1004	0.7076	0.8529	1.2641
sex	−0.1301	0.8780	0.1739	0.4544	0.6244	1.2347
age	0.0705	1.0730	0.0099	< 0.0001	1.0524	1.0940
total cholesterol	−0.0009	0.9991	0.0035	0.7893	0.9922	1.0059
LDL	−0.0011	0.9989	0.0036	0.7659	0.9919	1.0060
triglycerides	−0.0001	0.9999	0.0008	0.868	0.9982	1.0015
diabetes	0.1835	1.2014	0.0981	0.0615	0.9912	1.4562
smoking	0.2357	1.2657	0.0834	0.0047	1.0750	1.4904
systolic bloodpressure	0.0091	1.0092	0.0039	0.0197	1.0015	1.0169
diastolic bloodpressure	−0.0042	0.9958	0.0077	0.5847	0.9810	1.0109
CRP	−0.0524	0.9489	0.1124	0.6408	0.7613	1.1827
BMI	0.0166	1.0168	0.0263	0.5273	0.9657	1.0705
physical activity	0.1848	1.2029	0.1157	0.1103	0.9589	1.5091
waist circumference	−0.0058	0.9942	0.0105	0.58	0.9739	1.0149
coronary artery calcification	0.2542	1.2894	0.0287	< 0.0001	1.2189	1.3640

h2)

	coef	HR	se	p	lower	upper
rs7675998	0.2662	1.3050	0.1618	0.1	0.9503	1.7921
sex	−0.2370	0.7890	0.2805	0.3982	0.4554	1.3671
age	0.0321	1.0326	0.0147	0.029	1.0033	1.0628
total cholesterol	−0.0051	0.9949	0.0058	0.3756	0.9837	1.0062
LDL	0.0009	1.0009	0.0062	0.8859	0.9888	1.0132
triglycerides	0.0021	1.0021	0.0008	0.0086	1.0005	1.0037
diabetes	0.0723	1.0750	0.1429	0.6129	0.8124	1.4224
smoking	0.0750	1.0779	0.1368	0.5834	0.8245	1.4092
systolic bloodpressure	0.0013	1.0013	0.0069	0.8532	0.9879	1.0148
diastolic bloodpressure	−0.0085	0.9915	0.0132	0.5207	0.9661	1.0176
CRP	0.1909	1.2103	0.1175	0.1042	0.9614	1.5237
BMI	−0.0292	0.9713	0.0436	0.5035	0.8917	1.0579
physical activity	0.1035	1.1090	0.1951	0.596	0.7565	1.6257
waist circumference	−0.0172	0.9829	0.0164	0.2932	0.9519	1.0150
coronary artery calcification	0.3323	1.3942	0.0527	< 0.0001	1.2574	1.5458

i1)

	coef	HR	se	p	lower	upper
rs7675998	0.1188	1.1262	0.1141	0.2974	0.9006	1.4083
sex	−0.0947	0.9096	0.2029	0.6406	0.6112	1.3539
age	0.0738	1.0765	0.0115	< 0.0001	1.0526	1.1010
total cholesterol	−0.0003	0.9997	0.0053	0.9473	0.9894	1.0100
HDL	−0.0024	0.9976	0.0064	0.7117	0.9851	1.0103
LDL	−0.0036	0.9965	0.0054	0.5116	0.9859	1.0071
diabetes	0.0486	1.0498	0.1259	0.6997	0.8202	1.3435
smoking	0.3091	1.3622	0.0954	0.0012	1.1299	1.6424
systolic bloodpressure	0.0042	1.0042	0.0047	0.3675	0.9951	1.0135
diastolic bloodpressure	0.0126	1.0127	0.0092	0.1696	0.9946	1.0310
CRP	−0.0091	0.9909	0.1060	0.9316	0.8051	1.2197
BMI	0.0262	1.0265	0.0316	0.4072	0.9649	1.0922
physical activity	0.3629	1.4376	0.1352	0.0072	1.1030	1.8736
waist circumference	−0.0144	0.9857	0.0121	0.2314	0.9627	1.0092
coronary artery calcification	0.2794	1.3223	0.0333	< 0.0001	1.2386	1.4116

i2)

	coef	HR	se	p	lower	upper
rs7675998	0.0416	1.0425	0.1268	0.7429	0.8131	1.3366
sex	−0.1221	0.8851	0.2257	0.5886	0.5687	1.3776
age	0.0439	1.0448	0.0117	0.0002	1.0212	1.0690
total cholesterol	0.0019	1.0019	0.0033	0.5678	0.9954	1.0085
HDL	−0.0164	0.9837	0.0070	0.0194	0.9703	0.9974
LDL	−0.0039	0.9961	0.0035	0.268	0.9893	1.0030
diabetes	0.2681	1.3075	0.1065	0.0118	1.0611	1.6109
smoking	0.0755	1.0784	0.1059	0.4762	0.8762	1.3272
systolic bloodpressure	0.0103	1.0104	0.0049	0.035	1.0007	1.0202
diastolic bloodpressure	−0.0257	0.9746	0.0097	0.0083	0.9562	0.9934
CRP	0.1102	1.1165	0.1356	0.4166	0.8558	1.4565
BMI	−0.0217	0.9785	0.0328	0.508	0.9175	1.0435
physical activity	−0.1358	0.8730	0.1468	0.355	0.6546	1.1641
waist circumference	0.0002	1.0002	0.0130	0.9852	0.9751	1.0261
coronary artery calcification	0.2602	1.2971	0.0387	< 0.0001	1.2024	1.3993

j1)

	coef	HR	se	p	lower	upper
rs7675998	0.2306	1.2593	0.2136	0.2803	0.8286	1.9139
sex	−0.1027	0.9024	0.3686	0.7806	0.4382	1.8584
age	0.0596	1.0614	0.0190	0.0017	1.0226	1.1017
total cholesterol	0.0149	1.0150	0.0109	0.1724	0.9935	1.0370
HDL	−0.0228	0.9775	0.0133	0.0859	0.9524	1.0032
LDL	−0.0138	0.9863	0.0105	0.1872	0.9663	1.0067
triglycerides	0.0025	1.0025	0.0025	0.3268	0.9975	1.0074
diabetes	−0.0797	0.9234	0.2311	0.7301	0.5871	1.4523
smoking	0.1857	1.2041	0.1789	0.2992	0.8480	1.7098
CRP	0.1117	1.1182	0.1544	0.4692	0.8263	1.5133
BMI	−0.0020	0.9980	0.0614	0.9741	0.8848	1.1257
physical activity	0.2693	1.3091	0.2686	0.3161	0.7732	2.2162
waist circumference	−0.0122	0.9879	0.0243	0.6169	0.9419	1.0361
coronary artery calcification	0.3706	1.4485	0.0630	< 0.0001	1.2804	1.6388

j2)

	coef	HR	se	p	lower	upper
rs7675998	0.0503	1.0516	0.1444	0.7275	0.7923	1.3957
sex	−0.5562	0.5734	0.2652	0.036	0.3409	0.9643
age	0.0691	1.0716	0.0126	< 0.0001	1.0454	1.0983
total cholesterol	0.0021	1.0021	0.0054	0.6981	0.9916	1.0127
HDL	−0.0109	0.9891	0.0084	0.192	0.9730	1.0055
LDL	−0.0043	0.9957	0.0055	0.4333	0.9851	1.0065
triglycerides	0.0004	1.0004	0.0012	0.7634	0.9980	1.0027
diabetes	0.2664	1.3052	0.1316	0.043	1.0084	1.6893
smoking	0.2300	1.2586	0.1181	0.0515	0.9985	1.5865
CRP	0.0685	1.0709	0.1634	0.675	0.7774	1.4752
BMI	0.0619	1.0638	0.0372	0.0964	0.9890	1.1443
physical activity	0.2335	1.2630	0.1661	0.16	0.9119	1.7491
waist circumference	−0.0345	0.9661	0.0147	0.0193	0.9386	0.9944
coronary artery calcification	0.2159	1.2410	0.0397	< 0.0001	1.1481	1.3414

j3)

	coef	HR	se	p	lower	upper
rs7675998	0.0621	1.0640	0.1216	0.6098	0.8384	1.3505
sex	0.1647	1.1791	0.2069	0.4259	0.7860	1.7687
age	0.0610	1.0629	0.0115	< 0.0001	1.0392	1.0871
total cholesterol	−0.0068	0.9932	0.0044	0.1207	0.9846	1.0018
HDL	0.0020	1.0020	0.0020	0.3136	0.9981	1.0060
LDL	0.0020	1.0020	0.0047	0.6744	0.9928	1.0113
triglycerides	0.0014	1.0014	0.0007	0.0369	1.0001	1.0027
diabetes	0.1643	1.1786	0.1129	0.1455	0.9446	1.4706
smoking	0.1954	1.2158	0.1028	0.0573	0.9940	1.4870
CRP	−0.0115	0.9886	0.1092	0.9164	0.7981	1.2245
BMI	−0.0332	0.9673	0.0323	0.304	0.9079	1.0306
physical activity	0.0473	1.0485	0.1427	0.7401	0.7927	1.3867
waist circumference	0.0074	1.0074	0.0125	0.5562	0.9830	1.0324
coronary artery calcification	0.2913	1.3381	0.0381	< 0.0001	1.2419	1.4418

k1)

	coef	HR	se	p	lower	upper
rs7675998	−0.0453	0.9557	0.1028	0.6592	0.7813	1.1690
sex	−0.1023	0.9028	0.1720	0.5521	0.6445	1.2647
age	0.0647	1.0669	0.0094	< 0.0001	1.0473	1.0868
total cholesterol	0.0005	1.0005	0.0043	0.9103	0.9922	1.0089
HDL	−0.0074	0.9926	0.0059	0.2092	0.9812	1.0042
LDL	−0.0024	0.9976	0.0043	0.5813	0.9892	1.0061
triglycerides	0.0002	1.0002	0.0009	0.8558	0.9984	1.0019
smoking	0.2976	1.3466	0.0802	0.0002	1.1506	1.5759
systolic bloodpressure	0.0037	1.0037	0.0042	0.3755	0.9955	1.0120
diastolic bloodpressure	0.0032	1.0032	0.0081	0.6901	0.9875	1.0192
CRP	−0.0926	0.9115	0.1244	0.4565	0.7144	1.1632
BMI	0.0220	1.0222	0.0268	0.413	0.9698	1.0774
physical activity	0.2117	1.2357	0.1150	0.0657	0.9864	1.5481
waist circumference	−0.0077	0.9923	0.0103	0.4543	0.9725	1.0125
coronary artery calcification	0.2622	1.2997	0.0285	< 0.0001	1.2292	1.3743

k2)

	coef	HR	se	p	lower	upper
rs7675998	0.4969	1.6436	0.1545	0.0013	1.2141	2.2251
sex	−0.4317	0.6494	0.3230	0.1813	0.3448	1.2230
age	0.0432	1.0442	0.0167	0.0098	1.0105	1.0790
total cholesterol	−0.0058	0.9943	0.0089	0.5198	0.9770	1.0118
HDL	0.0082	1.0082	0.0100	0.414	0.9886	1.0283
LDL	0.0017	1.0018	0.0092	0.8486	0.9839	1.0199
triglycerides	0.0022	1.0022	0.0011	0.049	1.0000	1.0043
smoking	−0.0965	0.9080	0.1543	0.5315	0.6710	1.2286
systolic bloodpressure	0.0125	1.0126	0.0060	0.0374	1.0007	1.0246
diastolic bloodpressure	−0.0238	0.9764	0.0119	0.0442	0.9540	0.9994
CRP	0.1218	1.1296	0.0837	0.1456	0.9586	1.3309
BMI	0.0004	1.0004	0.0455	0.9932	0.9150	1.0938
physical activity	−0.1658	0.8472	0.2035	0.4153	0.5685	1.2625
waist circumference	−0.0232	0.9771	0.0182	0.2042	0.9428	1.0127
coronary artery calcification	0.3224	1.3804	0.0579	< 0.0001	1.2324	1.5462

l1)

	coef	HR	se	p	lower	upper
rs7675998	0.2746	1.3160	0.1450	0.0583	0.9904	1.7487
sex	−0.1193	0.8875	0.2388	0.6172	0.5558	1.4171
age	0.0850	1.0887	0.0156	< 0.0001	1.0560	1.1224
total cholesterol	−0.0044	0.9956	0.0067	0.5068	0.9826	1.0087
HDL	−0.0075	0.9925	0.0091	0.4104	0.9750	1.0104
LDL	−0.0007	0.9993	0.0068	0.9197	0.9862	1.0127
triglycerides	0.0020	1.0020	0.0016	0.2048	0.9989	1.0051
diabetes	0.3839	1.4681	0.1410	0.0065	1.1135	1.9355
systolic bloodpressure	0.0086	1.0086	0.0060	0.1562	0.9967	1.0206
diastolic bloodpressure	−0.0093	0.9908	0.0122	0.4466	0.9675	1.0147
CRP	0.0388	1.0396	0.1066	0.7156	0.8436	1.2812
BMI	−0.0103	0.9897	0.0356	0.7721	0.9230	1.0613
physical activity	0.0124	1.0125	0.1736	0.9431	0.7204	1.4229
waist circumference	−0.0017	0.9983	0.0144	0.9075	0.9706	1.0268
coronary artery calcification	0.2538	1.2889	0.0410	< 0.0001	1.1894	1.3969

l2)

	coef	HR	se	p	lower	upper
rs7675998	0.0778	1.0809	0.1321	0.5559	0.8343	1.4005
sex	−0.4574	0.6329	0.2799	0.1022	0.3657	1.0955
age	0.0559	1.0575	0.0127	< 0.0001	1.0315	1.0841
total cholesterol	−0.0021	0.9979	0.0064	0.7397	0.9853	1.0106
HDL	0.0053	1.0053	0.0080	0.5047	0.9897	1.0212
LDL	−0.0001	0.9999	0.0065	0.9899	0.9873	1.0127
triglycerides	0.0014	1.0014	0.0010	0.1694	0.9994	1.0035
diabetes	0.0893	1.0934	0.1207	0.4593	0.8631	1.3853
systolic bloodpressure	0.0064	1.0064	0.0051	0.2034	0.9965	1.0165
diastolic bloodpressure	−0.0084	0.9916	0.0098	0.389	0.9727	1.0108
CRP	−0.0447	0.9563	0.1583	0.7776	0.7012	1.3042
BMI	0.0544	1.0559	0.0404	0.1784	0.9755	1.1430
physical activity	0.2245	1.2517	0.1545	0.1462	0.9247	1.6944
waist circumference	−0.0196	0.9806	0.0150	0.1894	0.9522	1.0097
coronary artery calcification	0.2665	1.3054	0.0403	< 0.0001	1.2063	1.4127

l3)

	coef	HR	se	p	lower	upper
rs7675998	−0.1908	0.8263	0.1872	0.3081	0.5725	1.1926
sex	0.1233	1.1312	0.2934	0.6743	0.6365	2.0106
age	0.0428	1.0437	0.0163	0.0085	1.0110	1.0775
total cholesterol	−0.0006	0.9994	0.0068	0.9298	0.9862	1.0128
HDL	−0.0073	0.9927	0.0101	0.4706	0.9733	1.0126
LDL	−0.0009	0.9991	0.0070	0.8976	0.9855	1.0129
triglycerides	0.0006	1.0006	0.0011	0.5727	0.9985	1.0028
diabetes	0.0306	1.0310	0.1742	0.8608	0.7328	1.4506
systolic bloodpressure	0.0068	1.0068	0.0076	0.3706	0.9919	1.0220
diastolic bloodpressure	−0.0021	0.9979	0.0139	0.8789	0.9711	1.0254
CRP	0.0941	1.0987	0.1570	0.5489	0.8076	1.4946
BMI	−0.0352	0.9654	0.0463	0.447	0.8817	1.0571
physical activity	0.1485	1.1601	0.2093	0.4778	0.7698	1.7484
waist circumference	0.0051	1.0051	0.0187	0.7857	0.9690	1.0426
coronary artery calcification	0.2975	1.3465	0.0516	< 0.0001	1.2169	1.4899

m1)

	coef	HR	se	p	lower	upper
rs7675998	0.2592	1.2958	0.1796	0.1491	0.9113	1.8427
sex	0.0914	1.0957	0.2871	0.7502	0.6242	1.9234
age	0.0450	1.0460	0.0167	0.0071	1.0123	1.0809
total cholesterol	0.0102	1.0103	0.0076	0.1793	0.9953	1.0254
HDL	−0.0077	0.9923	0.0097	0.424	0.9736	1.0113
LDL	−0.0102	0.9899	0.0077	0.1846	0.9751	1.0049
triglycerides	0.0001	1.0001	0.0014	0.9149	0.9975	1.0028
diabetes	0.2998	1.3496	0.1878	0.1104	0.9340	1.9502
smoking	0.2119	1.2360	0.1431	0.1388	0.9336	1.6364
systolic bloodpressure	0.0147	1.0148	0.0070	0.0366	1.0009	1.0289
diastolic bloodpressure	−0.0189	0.9813	0.0157	0.2288	0.9517	1.0119
CRP	0.1885	1.2074	0.1558	0.2265	0.8896	1.6387
BMI	−0.0058	0.9942	0.0502	0.9075	0.9011	1.0969
physical activity	0.5234	1.6878	0.2151	0.015	1.1071	2.5730
coronary artery calcification	0.2948	1.3429	0.0511	< 0.0001	1.2148	1.4844

m2)

	coef	HR	se	p	lower	upper
rs7675998	0.0507	1.0520	0.0962	0.5982	0.8713	1.2702
sex	−0.1065	0.8990	0.1405	0.4484	0.6826	1.1840
age	0.0620	1.0640	0.0092	< 0.0001	1.0449	1.0834
total cholesterol	−0.0047	0.9953	0.0042	0.2679	0.9871	1.0036
HDL	−0.0039	0.9961	0.0058	0.5007	0.9849	1.0074
LDL	0.0009	1.0009	0.0043	0.8421	0.9924	1.0094
triglycerides	0.0013	1.0013	0.0007	0.0684	0.9999	1.0028
diabetes	0.1262	1.1345	0.0846	0.1359	0.9611	1.3391
smoking	0.1552	1.1679	0.0815	0.0569	0.9955	1.3701
systolic bloodpressure	0.0050	1.0050	0.0038	0.1866	0.9976	1.0126
diastolic bloodpressure	−0.0005	0.9995	0.0072	0.9468	0.9854	1.0138
CRP	−0.0124	0.9877	0.0908	0.8913	0.8267	1.1800
BMI	−0.0002	0.9998	0.0141	0.9906	0.9727	1.0278
physical activity	0.0319	1.0324	0.1118	0.7755	0.8292	1.2855
coronary artery calcification	0.2637	1.3017	0.0289	< 0.0001	1.2299	1.3777

n1)

	coef	HR	se	p	lower	upper
rs7675998	−0.0131	0.9870	0.2104	0.9505	0.6535	1.4907
sex	−0.0645	0.9375	0.3416	0.8502	0.4799	1.8313
age	0.0660	1.0683	0.0195	0.0007	1.0282	1.1099
total cholesterol	0.0128	1.0129	0.0079	0.1071	0.9972	1.0288
HDL	−0.0157	0.9844	0.0104	0.1314	0.9646	1.0047
LDL	−0.0151	0.9850	0.0079	0.0557	0.9699	1.0004
triglycerides	−0.0008	0.9992	0.0015	0.5958	0.9962	1.0022
diabetes	0.1932	1.2131	0.2404	0.4216	0.7573	1.9431
smoking	0.2182	1.2439	0.1474	0.1386	0.9319	1.6604
systolic bloodpressure	0.0072	1.0072	0.0076	0.3472	0.9922	1.0224
diastolic bloodpressure	−0.0006	0.9994	0.0163	0.9719	0.9679	1.0319
CRP	0.2056	1.2283	0.1988	0.3011	0.8318	1.8136
physical activity	0.6401	1.8967	0.2375	0.007	1.1908	3.0211
waist circumference	−0.0238	0.9765	0.0183	0.1947	0.9421	1.0122
coronary artery calcification	0.3788	1.4606	0.0609	< 0.0001	1.2963	1.6457

n2)

	coef	HR	se	p	lower	upper
rs7675998	0.1008	1.1060	0.0932	0.2794	0.9214	1.3275
sex	−0.1500	0.8607	0.1413	0.2885	0.6525	1.1354
age	0.0580	1.0597	0.0089	< 0.0001	1.0413	1.0785
total cholesterol	−0.0053	0.9948	0.0041	0.1988	0.9868	1.0028
HDL	−0.0013	0.9987	0.0056	0.8094	0.9878	1.0096
LDL	0.0019	1.0019	0.0042	0.6465	0.9937	1.0102
triglycerides	0.0016	1.0016	0.0007	0.0233	1.0002	1.0029
diabetes	0.1458	1.1569	0.0830	0.0789	0.9833	1.3612
smoking	0.1798	1.1970	0.0795	0.0237	1.0244	1.3988
systolic bloodpressure	0.0062	1.0062	0.0038	0.1026	0.9988	1.0136
diastolic bloodpressure	−0.0062	0.9938	0.0072	0.3868	0.9799	1.0079
CRP	0.0049	1.0049	0.0852	0.9541	0.8504	1.1875
physical activity	0.0340	1.0346	0.1098	0.7568	0.8342	1.2832
waist circumference	−0.0037	0.9963	0.0057	0.5189	0.9852	1.0076
coronary artery calcification	0.2471	1.2803	0.0276	< 0.0001	1.2127	1.3516

S4D Table. Results of Cox regression models for rs2736100.
coef: coefficient, HR: Hazard Ratio, se: Standard error,
lower/upper: lower/upper boundarie of the 95% confidence interval
a) crude, b) adjusted, c1) young age, c2) older age, d1) male, d2) female,
e1) low hsCRP, e2) intermediate hsCRP, e3) high hsCRP, f1) low total cholesterol,
f2) high total cholesterol, g1) low LDL, g2) high LDL, h1) normal HDL, h2) high HDL,
i1) low triglycerides, i2) high triglycerides, j1) ideal blood pressure,
j2) normal/high normal blood pressure, j3) hypertension, k1) no diabetes, k2) diabetes,
l1) never smoker, l2) former smoker, l3) current smoker, m1) normal waist circumference,
m2) high waist circumference, n1) normal BMI, n2) high BMI

a)

	coef	HR	se	p	lower	upper
rs2736100	0.0627	1.0647	0.0657	0.3402	0.936	1.211

b)

	coef	HR	se	p	lower	upper
rs2736100	0.0376	1.0383	0.0680	0.5803	0.9088	1.1862
sex	−0.1115	0.8945	0.1487	0.4534	0.6684	1.1972
age	0.0594	1.0612	0.0081	< 0.0001	1.0445	1.0782
total cholesterol	−0.0022	0.9978	0.0038	0.5537	0.9904	1.0052
HDL	−0.0044	0.9956	0.0050	0.3727	0.9860	1.0053
LDL	−0.0010	0.9990	0.0038	0.7927	0.9915	1.0066
triglycerides	0.0011	1.0011	0.0006	0.0887	0.9998	1.0024
diabetes	0.1549	1.1675	0.0799	0.0526	0.9983	1.3655
smoking	0.1830	1.2009	0.0696	0.0086	1.0477	1.3764
systolic bloodpressure	0.0071	1.0071	0.0034	0.036	1.0005	1.0138
diastolic bloodpressure	−0.0050	0.9950	0.0065	0.4458	0.9823	1.0079
CRP	0.0009	1.0009	0.0699	0.9896	0.8728	1.1479
BMI	0.0047	1.0047	0.0221	0.8325	0.9621	1.0492
physical activity	0.1607	1.1744	0.0983	0.1021	0.9685	1.4239
waist circumference	−0.0083	0.9918	0.0087	0.343	0.9750	1.0089
coronary artery calcification	0.2747	1.3161	0.0250	< 0.0001	1.2532	1.3821

c1)

	coef	HR	se	p	lower	upper
rs2736100	0.2366	1.2670	0.1474	0.1083	0.9491	1.6913
sex	−0.1076	0.8980	0.2851	0.7058	0.5136	1.5701
total cholesterol	0.0023	1.0023	0.0069	0.7397	0.9889	1.0159
HDL	−0.0149	0.9852	0.0102	0.1453	0.9657	1.0052
LDL	−0.0019	0.9981	0.0070	0.78	0.9845	1.0118
triglycerides	0.0002	1.0002	0.0014	0.8537	0.9976	1.0029
diabetes	−0.0069	0.9932	0.1975	0.9723	0.6744	1.4625
smoking	0.3061	1.3581	0.1339	0.0223	1.0446	1.7658
systolic bloodpressure	−0.0039	0.9961	0.0089	0.6603	0.9788	1.0137
diastolic bloodpressure	0.0180	1.0182	0.0162	0.267	0.9863	1.0510
CRP	0.1039	1.1095	0.1625	0.5227	0.8068	1.5257
BMI	0.0054	1.0054	0.0467	0.9075	0.9175	1.1018
physical activity	0.2087	1.2321	0.2139	0.3294	0.8101	1.8739
waist circumference	−0.0138	0.9863	0.0183	0.4519	0.9516	1.0223
coronary artery calcification	0.2925	1.3398	0.0465	< 0.0001	1.2232	1.4675

c2)

	coef	HR	se	p	lower	upper
rs2736100	−0.0319	0.9686	0.0774	0.6797	0.8323	1.1272
sex	0.3415	1.4071	0.1745	0.0503	0.9995	1.9810
total cholesterol	−0.0045	0.9955	0.0042	0.2876	0.9874	1.0038
HDL	−0.0004	0.9996	0.0046	0.9363	0.9906	1.0087
LDL	0.0001	1.0001	0.0043	0.9835	0.9917	1.0086
triglycerides	0.0011	1.0011	0.0007	0.0998	0.9998	1.0025
diabetes	0.1838	1.2018	0.0885	0.0378	1.0104	1.4294
smoking	0.0800	1.0833	0.0819	0.3285	0.9227	1.2719
systolic bloodpressure	0.0138	1.0138	0.0036	0.0001	1.0068	1.0210
diastolic bloodpressure	−0.0181	0.9821	0.0070	0.0103	0.9686	0.9958
CRP	−0.0054	0.9946	0.0737	0.942	0.8608	1.1493
BMI	−0.0106	0.9895	0.0257	0.6805	0.9408	1.0406
physical activity	0.1400	1.1503	0.1112	0.2081	0.9250	1.4304
waist circumference	−0.0013	0.9987	0.0100	0.8953	0.9793	1.0184
coronary artery calcification	0.2891	1.3353	0.0294	< 0.0001	1.2605	1.4145

d1)

	coef	HR	se	p	lower	upper
rs2736100	−0.0271	0.9733	0.0827	0.7434	0.8277	1.1445
age	0.0433	1.0442	0.0096	< 0.0001	1.0247	1.0641
total cholesterol	−0.0059	0.9942	0.0048	0.2238	0.9848	1.0036
HDL	0.0019	1.0019	0.0061	0.7512	0.9900	1.0141
LDL	0.0027	1.0027	0.0049	0.5821	0.9931	1.0123
triglycerides	0.0015	1.0015	0.0008	0.0556	1.0000	1.0030
diabetes	0.1715	1.1870	0.0910	0.0594	0.9932	1.4187
smoking	0.1078	1.1139	0.0891	0.226	0.9354	1.3263
systolic bloodpressure	0.0048	1.0048	0.0043	0.2601	0.9965	1.0132
diastolic bloodpressure	−0.0025	0.9975	0.0080	0.7535	0.9821	1.0132
CRP	0.0027	1.0027	0.0789	0.9727	0.8591	1.1703
BMI	0.0342	1.0348	0.0290	0.2388	0.9775	1.0954
physical activity	0.1647	1.1790	0.1208	0.173	0.9304	1.4941
waist circumference	−0.0126	0.9875	0.0112	0.2633	0.9661	1.0095
coronary artery calcification	0.2976	1.3466	0.0333	< 0.0001	1.2616	1.4373

d2)

	coef	HR	se	p	lower	upper
rs2736100	0.1648	1.1791	0.1207	0.1721	0.9308	1.4938
age	0.0985	1.1035	0.0152	< 0.0001	1.0710	1.1369
total cholesterol	0.0019	1.0019	0.0055	0.7323	0.9911	1.0128
HDL	−0.0137	0.9864	0.0081	0.0901	0.9710	1.0021
LDL	−0.0058	0.9942	0.0056	0.3003	0.9833	1.0052
triglycerides	0.0012	1.0012	0.0013	0.3776	0.9986	1.0038
diabetes	0.1003	1.1055	0.1725	0.5607	0.7885	1.5501
smoking	0.3389	1.4034	0.1098	0.002	1.1318	1.7402
systolic bloodpressure	0.0094	1.0095	0.0056	0.0943	0.9984	1.0207
diastolic bloodpressure	−0.0062	0.9938	0.0117	0.5943	0.9713	1.0168
CRP	0.0135	1.0136	0.1511	0.9288	0.7538	1.3629
BMI	−0.0280	0.9724	0.0349	0.4227	0.9082	1.0412
physical activity	0.1399	1.1501	0.1712	0.4139	0.8223	1.6087
waist circumference	−0.0059	0.9941	0.0141	0.674	0.9670	1.0219
coronary artery calcification	0.2347	1.2645	0.0385	< 0.0001	1.1726	1.3636

e1)

	coef	HR	se	p	lower	upper
rs2736100	−0.0085	0.9916	0.0826	0.9182	0.8434	1.1658
sex	−0.1748	0.8396	0.1814	0.3352	0.5884	1.1981
age	0.0683	1.0707	0.0098	< 0.0001	1.0503	1.0914
total cholesterol	0.0001	1.0001	0.0043	0.987	0.9916	1.0086
HDL	−0.0063	0.9937	0.0058	0.2747	0.9825	1.0050
LDL	−0.0038	0.9962	0.0044	0.3871	0.9876	1.0049
triglycerides	0.0011	1.0011	0.0007	0.1481	0.9996	1.0025
diabetes	0.1308	1.1397	0.1012	0.1964	0.9346	1.3899
smoking	0.1755	1.1918	0.0859	0.0412	1.0071	1.4105
systolic bloodpressure	0.0077	1.0077	0.0043	0.0705	0.9994	1.0162
diastolic bloodpressure	−0.0042	0.9958	0.0083	0.609	0.9797	1.0121
BMI	0.0382	1.0389	0.0280	0.1731	0.9834	1.0976
physical activity	0.1798	1.1970	0.1183	0.1286	0.9492	1.5093
waist circumference	−0.0165	0.9836	0.0105	0.115	0.9636	1.0040
coronary artery calcification	0.2712	1.3116	0.0301	< 0.0001	1.2365	1.3913

e2)

	coef	HR	se	p	lower	upper
rs2736100	0.1675	1.1823	0.1953	0.391	0.8063	1.7337
sex	0.662	1.9387	0.4537	0.1445	0.7968	4.7173
age	0.0939	1.0985	0.0247	0.0001	1.0465	1.1531
total cholesterol	−0.0046	0.9954	0.0132	0.7281	0.97	1.0215
HDL	−0.0119	0.9882	0.017	0.4853	0.9557	1.0218
LDL	0.005	1.005	0.0124	0.6892	0.9808	1.0298
triglycerides	−0.0038	0.9962	0.0026	0.1494	0.991	1.0014
diabetes	0.3497	1.4187	0.1928	0.0696	0.9723	2.0699
smoking	0.1812	1.1986	0.2106	0.3897	0.7932	1.8112
systolic bloodpressure	−0.0014	0.9986	0.0096	0.8851	0.9801	1.0175
diastolic bloodpressure	0.0065	1.0066	0.0175	0.7089	0.9726	1.0417
BMI	−0.1306	0.8776	0.0732	0.0745	0.7603	1.013
physical activity	−0.5462	0.5791	0.2837	0.0542	0.3321	1.0098
waist circumference	0.0606	1.0625	0.0293	0.0386	1.0032	1.1254
coronary artery calcification	0.2779	1.3204	0.0747	0.0002	1.1406	1.5284

e3)

	coef	HR	se	p	lower	upper
rs2736100	0.1225	1.1303	0.1572	0.4357	0.8307	1.5381
sex	−0.3235	0.7236	0.3545	0.3615	0.3612	1.4497
age	0.0086	1.0086	0.0186	0.6432	0.9726	1.0461
total cholesterol	−0.0053	0.9948	0.0066	0.4241	0.9820	1.0077
HDL	0.0008	1.0008	0.0035	0.8256	0.9939	1.0078
LDL	0.0036	1.0036	0.0068	0.6029	0.9902	1.0171
triglycerides	0.0023	1.0023	0.0012	0.0704	0.9998	1.0047
diabetes	0.1707	1.1861	0.1737	0.326	0.8438	1.6672
smoking	0.1302	1.1390	0.1602	0.4165	0.8321	1.5592
systolic bloodpressure	0.0059	1.0059	0.0073	0.4165	0.9917	1.0204
diastolic bloodpressure	−0.0183	0.9818	0.0150	0.2201	0.9535	1.0110
BMI	−0.0311	0.9694	0.0484	0.5205	0.8816	1.0659
physical activity	0.4927	1.6368	0.2414	0.0412	1.0198	2.6270
waist circumference	−0.0174	0.9827	0.0203	0.392	0.9444	1.0227
coronary artery calcification	0.2916	1.3386	0.0587	< 0.0001	1.1930	1.5019

f1)

	coef	HR	se	p	lower	upper
rs2736100	0.0355	1.0361	0.1279	0.7815	0.8063	1.3314
sex	0.4047	1.4988	0.2706	0.1348	0.8818	2.5475
age	0.0730	1.0757	0.0161	< 0.0001	1.0422	1.1103
HDL	−0.0067	0.9933	0.0086	0.4345	0.9766	1.0102
LDL	−0.0049	0.9951	0.0049	0.3095	0.9856	1.0046
triglycerides	0.0006	1.0006	0.0013	0.6286	0.9980	1.0033
diabetes	0.2002	1.2216	0.1504	0.1832	0.9097	1.6404
smoking	−0.0024	0.9976	0.1329	0.9858	0.7689	1.2945
systolic bloodpressure	0.0033	1.0033	0.0067	0.6273	0.9901	1.0166
diastolic bloodpressure	−0.0024	0.9976	0.0131	0.8544	0.9722	1.0236
CRP	−0.0383	0.9624	0.1228	0.7551	0.7566	1.2243
BMI	−0.1049	0.9004	0.0432	0.0153	0.8273	0.9801
physical activity	0.2587	1.2952	0.1916	0.177	0.8897	1.8856
waist circumference	0.0301	1.0306	0.0160	0.0592	0.9988	1.0633
coronary artery calcification	0.2983	1.3475	0.0480	< 0.0001	1.2266	1.4804

f2)

	coef	HR	se	p	lower	upper
rs2736100	0.0132	1.0133	0.0807	0.8699	0.8651	1.1869
sex	−0.3057	0.7366	0.1773	0.0846	0.5204	1.0426
age	0.0555	1.0571	0.0094	< 0.0001	1.0377	1.0769
HDL	−0.0059	0.9941	0.0044	0.1774	0.9856	1.0027
LDL	−0.0030	0.9970	0.0019	0.126	0.9932	1.0008
triglycerides	0.0007	1.0007	0.0005	0.1332	0.9998	1.0017
diabetes	0.1241	1.1322	0.0955	0.1935	0.9390	1.3651
smoking	0.2632	1.3010	0.0827	0.0015	1.1065	1.5298
systolic bloodpressure	0.0079	1.0080	0.0040	0.0453	1.0002	1.0159
diastolic bloodpressure	−0.0066	0.9935	0.0076	0.3902	0.9787	1.0084
CRP	0.0132	1.0133	0.0849	0.8762	0.8579	1.1968
BMI	0.0473	1.0484	0.0255	0.0638	0.9973	1.1022
physical activity	0.1210	1.1286	0.1166	0.2994	0.8981	1.4183
waist circumference	−0.0219	0.9783	0.0102	0.0308	0.9590	0.9980
coronary artery calcification	0.2660	1.3047	0.0297	< 0.0001	1.2309	1.3830

g1)

	coef	HR	se	p	lower	upper
rs2736100	0.0126	1.0126	0.1414	0.9292	0.7676	1.3360
sex	0.1688	1.1839	0.2942	0.5662	0.6651	2.1073
age	0.0818	1.0852	0.0176	< 0.0001	1.0485	1.1232
total cholesterol	−0.0020	0.9980	0.0047	0.6718	0.9890	1.0072
HDL	−0.0011	0.9989	0.0071	0.8719	0.9850	1.0129
triglycerides	0.0018	1.0018	0.0007	0.0134	1.0004	1.0032
diabetes	0.2104	1.2342	0.1674	0.2088	0.8890	1.7134
smoking	0.1867	1.2052	0.1440	0.1948	0.9089	1.5982
systolic bloodpressure	0.0014	1.0014	0.0073	0.852	0.9871	1.0158
diastolic bloodpressure	−0.0057	0.9943	0.0139	0.6826	0.9676	1.0218
CRP	−0.2706	0.7629	0.2306	0.2405	0.4855	1.1988
BMI	−0.0758	0.9270	0.0458	0.098	0.8475	1.0141
physical activity	0.4897	1.6319	0.2102	0.0198	1.0808	2.4639
waist circumference	0.0220	1.0222	0.0172	0.2016	0.9883	1.0573
coronary artery calcification	0.2680	1.3073	0.0518	< 0.0001	1.1811	1.4470

g2)

	coef	HR	se	p	lower	upper
rs2736100	0.0240	1.0243	0.0782	0.7592	0.8786	1.1940
sex	−0.1863	0.8300	0.1718	0.278	0.5927	1.1622
age	0.0543	1.0558	0.0092	< 0.0001	1.0369	1.0751
total cholesterol	−0.0032	0.9968	0.0019	0.0819	0.9931	1.0004
HDL	−0.0040	0.9960	0.0047	0.3926	0.9870	1.0052
triglycerides	0.0010	1.0010	0.0007	0.1668	0.9996	1.0023
diabetes	0.1332	1.1425	0.0922	0.1484	0.9537	1.3687
smoking	0.2000	1.2214	0.0801	0.0125	1.0439	1.4291
systolic bloodpressure	0.0078	1.0078	0.0039	0.0439	1.0002	1.0155
diastolic bloodpressure	−0.0042	0.9958	0.0075	0.571	0.9812	1.0105
CRP	0.0891	1.0932	0.0847	0.2925	0.9260	1.2906
BMI	0.0255	1.0259	0.0250	0.3068	0.9768	1.0773
physical activity	0.0810	1.0844	0.1127	0.472	0.8695	1.3524
waist circumference	−0.0167	0.9835	0.0099	0.0915	0.9646	1.0027
coronary artery calcification	0.2761	1.3180	0.0290	< 0.0001	1.2452	1.3952

h1)

	coef	HR	se	p	lower	upper
rs2736100	0.0782	1.0814	0.0792	0.3229	0.9260	1.2628
sex	−0.1279	0.8799	0.1725	0.4585	0.6275	1.2340
age	0.0700	1.0725	0.0098	< 0.0001	1.0520	1.0934
total cholesterol	−0.0019	0.9981	0.0035	0.5764	0.9913	1.0049
LDL	−0.0005	0.9995	0.0036	0.8913	0.9925	1.0066
triglycerides	−0.0001	0.9999	0.0008	0.8989	0.9983	1.0015
diabetes	0.1716	1.1872	0.0982	0.0804	0.9794	1.4391
smoking	0.2414	1.2730	0.0828	0.0036	1.0823	1.4974
systolic bloodpressure	0.0095	1.0096	0.0039	0.0141	1.0019	1.0173
diastolic bloodpressure	−0.0043	0.9957	0.0076	0.5727	0.9810	1.0107
CRP	−0.0798	0.9233	0.0987	0.4189	0.7609	1.1204
BMI	0.0202	1.0204	0.0260	0.4363	0.9698	1.0738
physical activity	0.1786	1.1956	0.1147	0.1193	0.9549	1.4968
waist circumference	−0.0070	0.9931	0.0104	0.5036	0.9730	1.0135
coronary artery calcification	0.2532	1.2882	0.0285	< 0.0001	1.2182	1.3622

h2)

	coef	HR	se	p	lower	upper
rs2736100	−0.0559	0.9456	0.1358	0.6803	0.7247	1.2338
sex	−0.2733	0.7609	0.2802	0.3294	0.4393	1.3177
age	0.0344	1.0350	0.0147	0.0192	1.0056	1.0652
total cholesterol	−0.0047	0.9953	0.0058	0.4227	0.9840	1.0068
LDL	0.0008	1.0008	0.0063	0.8952	0.9886	1.0132
triglycerides	0.0021	1.0021	0.0008	0.0111	1.0005	1.0037
diabetes	0.0715	1.0742	0.1416	0.6134	0.8138	1.4178
smoking	0.0413	1.0421	0.1342	0.7584	0.8012	1.3556
systolic bloodpressure	0.0008	1.0008	0.0069	0.9088	0.9873	1.0144
diastolic bloodpressure	−0.0066	0.9934	0.0133	0.6173	0.9679	1.0196
CRP	0.1720	1.1877	0.1157	0.1372	0.9467	1.4901
BMI	−0.0322	0.9683	0.0427	0.4504	0.8905	1.0528
physical activity	0.1120	1.1185	0.1922	0.56	0.7675	1.6301
waist circumference	−0.0158	0.9843	0.0162	0.3271	0.9536	1.0160
coronary artery calcification	0.3322	1.3940	0.0525	< 0.0001	1.2577	1.5451

i1)

	coef	HR	se	p	lower	upper
rs2736100	0.0496	1.0509	0.0924	0.591	0.8768	1.2595
sex	−0.0935	0.9107	0.2006	0.6411	0.6147	1.3493
age	0.0730	1.0758	0.0114	< 0.0001	1.0521	1.1000
total cholesterol	−0.0014	0.9986	0.0052	0.7862	0.9884	1.0089
HDL	−0.0025	0.9975	0.0064	0.6941	0.9850	1.0101
LDL	−0.0028	0.9972	0.0054	0.6052	0.9868	1.0078
diabetes	0.0407	1.0415	0.1258	0.7464	0.8139	1.3328
smoking	0.3070	1.3593	0.0944	0.0011	1.1297	1.6355
systolic bloodpressure	0.0046	1.0046	0.0046	0.3202	0.9955	1.0137
diastolic bloodpressure	0.0124	1.0125	0.0091	0.1726	0.9946	1.0307
CRP	−0.0529	0.9485	0.0885	0.5501	0.7974	1.1282
BMI	0.0281	1.0285	0.0311	0.3658	0.9677	1.0932
physical activity	0.3498	1.4187	0.1335	0.0088	1.0920	1.8431
waist circumference	−0.0148	0.9853	0.0119	0.2135	0.9626	1.0086
coronary artery calcification	0.2771	1.3193	0.0330	< 0.0001	1.2366	1.4075

i2)

	coef	HR	se	p	lower	upper
rs2736100	−0.0198	0.9804	0.1015	0.8456	0.8035	1.1962
sex	−0.1253	0.8822	0.2254	0.5783	0.5671	1.3723
age	0.0444	1.0454	0.0116	0.0001	1.0218	1.0695
total cholesterol	0.0019	1.0019	0.0034	0.5682	0.9954	1.0085
HDL	−0.0174	0.9827	0.0070	0.0129	0.9693	0.9963
LDL	−0.0038	0.9962	0.0035	0.2785	0.9893	1.0031
diabetes	0.2503	1.2845	0.1060	0.0182	1.0434	1.5812
smoking	0.0563	1.0579	0.1050	0.5916	0.8612	1.2995
systolic bloodpressure	0.0099	1.0100	0.0049	0.0431	1.0003	1.0197
diastolic bloodpressure	−0.0248	0.9755	0.0097	0.0108	0.9571	0.9943
CRP	0.1165	1.1236	0.1348	0.3873	0.8627	1.4634
BMI	−0.0231	0.9772	0.0325	0.4779	0.9168	1.0415
physical activity	−0.1211	0.8860	0.1457	0.406	0.6659	1.1788
waist circumference	0.0001	1.0001	0.0129	0.9965	0.9751	1.0257
coronary artery calcification	0.2676	1.3068	0.0386	< 0.0001	1.2114	1.4096

j1)

	coef	HR	se	p	lower	upper
rs2736100	0.0856	1.0894	0.1774	0.6294	0.7695	1.5423
sex	−0.1235	0.8838	0.3622	0.7331	0.4346	1.7975
age	0.0576	1.0593	0.0188	0.0022	1.0210	1.0990
total cholesterol	0.0142	1.0143	0.0107	0.1872	0.9931	1.0359
HDL	−0.0245	0.9758	0.0132	0.0639	0.9509	1.0014
LDL	−0.0145	0.9856	0.0103	0.1599	0.9660	1.0057
triglycerides	0.0023	1.0023	0.0024	0.3409	0.9975	1.0072
diabetes	−0.0607	0.9411	0.2253	0.7877	0.6052	1.4636
smoking	0.2277	1.2557	0.1756	0.1947	0.8900	1.7717
CRP	0.1270	1.1354	0.1468	0.3872	0.8515	1.5139
BMI	0.0090	1.0091	0.0597	0.8796	0.8977	1.1343
physical activity	0.2541	1.2892	0.2636	0.3351	0.7691	2.1612
waist circumference	−0.0164	0.9837	0.0237	0.4876	0.9392	1.0304
coronary artery calcification	0.3619	1.4360	0.0616	< 0.0001	1.2726	1.6205

j2)

	coef	HR	se	p	lower	upper
rs2736100	0.0346	1.0352	0.1172	0.7676	0.8228	1.3026
sex	−0.5734	0.5636	0.2645	0.0302	0.3356	0.9465
age	0.0708	1.0734	0.0126	< 0.0001	1.0473	1.1002
total cholesterol	0.0012	1.0012	0.0054	0.8226	0.9906	1.0120
HDL	−0.0095	0.9905	0.0083	0.2539	0.9745	1.0068
LDL	−0.0036	0.9964	0.0055	0.5123	0.9856	1.0073
triglycerides	0.0004	1.0004	0.0012	0.7085	0.9981	1.0028
diabetes	0.2586	1.2951	0.1318	0.0497	1.0003	1.6767
smoking	0.2411	1.2726	0.1175	0.0402	1.0109	1.6022
CRP	0.0665	1.0687	0.1636	0.6845	0.7756	1.4727
BMI	0.0650	1.0671	0.0369	0.0782	0.9927	1.1472
physical activity	0.2173	1.2427	0.1642	0.1857	0.9007	1.7146
waist circumference	−0.0344	0.9661	0.0147	0.0192	0.9387	0.9944
coronary artery calcification	0.2124	1.2367	0.0394	< 0.0001	1.1446	1.3361

j3)

	coef	HR	se	p	lower	upper
rs2736100	0.0039	1.0039	0.0961	0.9679	0.8315	1.2120
sex	0.1504	1.1623	0.2065	0.4665	0.7754	1.7422
age	0.0614	1.0633	0.0115	< 0.0001	1.0396	1.0876
total cholesterol	−0.0073	0.9927	0.0044	0.0961	0.9842	1.0013
HDL	0.0019	1.0019	0.0022	0.3663	0.9977	1.0062
LDL	0.0025	1.0025	0.0047	0.5883	0.9934	1.0118
triglycerides	0.0014	1.0014	0.0007	0.0345	1.0001	1.0028
diabetes	0.1407	1.1511	0.1130	0.2131	0.9224	1.4365
smoking	0.1612	1.1749	0.1021	0.1144	0.9618	1.4352
CRP	−0.0503	0.9509	0.0961	0.6008	0.7876	1.1481
BMI	−0.0350	0.9656	0.0321	0.2751	0.9068	1.0282
physical activity	0.0607	1.0626	0.1409	0.6666	0.8061	1.4007
waist circumference	0.0077	1.0077	0.0124	0.5348	0.9835	1.0325
coronary artery calcification	0.2973	1.3462	0.0380	< 0.0001	1.2497	1.4502

k1)

	coef	HR	se	p	lower	upper
rs2736100	0.0030	1.0030	0.0789	0.9702	0.8593	1.1706
sex	−0.1076	0.8980	0.1706	0.5281	0.6428	1.2544
age	0.0650	1.0672	0.0094	< 0.0001	1.0478	1.0869
total cholesterol	−0.0001	0.9999	0.0042	0.9841	0.9917	1.0082
HDL	−0.0073	0.9928	0.0058	0.2125	0.9815	1.0042
LDL	−0.0022	0.9978	0.0043	0.611	0.9895	1.0063
triglycerides	0.0002	1.0002	0.0009	0.8371	0.9984	1.0019
smoking	0.2923	1.3396	0.0795	0.0002	1.1462	1.5655
systolic bloodpressure	0.0040	1.0040	0.0042	0.336	0.9959	1.0122
diastolic bloodpressure	0.0035	1.0035	0.0080	0.6589	0.9879	1.0194
CRP	−0.1111	0.8948	0.1140	0.3298	0.7156	1.1189
BMI	0.0240	1.0243	0.0266	0.3674	0.9722	1.0791
physical activity	0.2036	1.2259	0.1138	0.0735	0.9808	1.5322
waist circumference	−0.0090	0.9911	0.0102	0.3788	0.9715	1.0111
coronary artery calcification	0.2628	1.3006	0.0283	< 0.0001	1.2305	1.3747

k2)

	coef	HR	se	p	lower	upper
rs2736100	0.1490	1.1607	0.1390	0.2837	0.8839	1.5242
sex	−0.3890	0.6777	0.3211	0.2257	0.3612	1.2717
age	0.0482	1.0494	0.0168	0.0042	1.0153	1.0846
total cholesterol	−0.0048	0.9952	0.0091	0.5999	0.9776	1.0132
HDL	0.0050	1.0050	0.0102	0.6246	0.9851	1.0252
LDL	0.0004	1.0004	0.0092	0.9633	0.9825	1.0187
triglycerides	0.0020	1.0020	0.0011	0.0776	0.9998	1.0043
smoking	−0.1073	0.8982	0.1569	0.4938	0.6605	1.2216
systolic bloodpressure	0.0110	1.0110	0.0061	0.0708	0.9991	1.0232
diastolic bloodpressure	−0.0175	0.9826	0.0117	0.1342	0.9603	1.0054
CRP	0.1172	1.1243	0.0836	0.1611	0.9544	1.3246
BMI	−0.0203	0.9799	0.0440	0.644	0.8990	1.0680
physical activity	−0.0744	0.9283	0.2023	0.713	0.6244	1.3801
waist circumference	−0.0142	0.9859	0.0175	0.4175	0.9526	1.0203
coronary artery calcification	0.3114	1.3653	0.0566	< 0.0001	1.2219	1.5256

I1)

	coef	HR	se	p	lower	upper
rs2736100	0.2160	1.2411	0.1191	0.0699	0.9826	1.5675
sex	−0.1735	0.8408	0.2335	0.4575	0.5321	1.3286
age	0.0889	1.0930	0.0156	< 0.0001	1.0602	1.1269
total cholesterol	−0.0033	0.9968	0.0067	0.6281	0.9837	1.0100
HDL	−0.0107	0.9893	0.0090	0.2346	0.9720	1.0070
LDL	−0.0021	0.9979	0.0068	0.7526	0.9847	1.0112
triglycerides	0.0014	1.0014	0.0015	0.3475	0.9984	1.0045
diabetes	0.3866	1.4720	0.1408	0.006	1.1171	1.9396
systolic bloodpressure	0.0096	1.0096	0.0060	0.1102	0.9978	1.0215
diastolic bloodpressure	−0.0068	0.9932	0.0120	0.5694	0.9702	1.0168
CRP	0.0243	1.0246	0.1084	0.8227	0.8284	1.2672
BMI	−0.0110	0.9891	0.0347	0.7514	0.9240	1.0587
physical activity	0.0260	1.0264	0.1703	0.8785	0.7351	1.4331
waist circumference	−0.0014	0.9986	0.0138	0.9194	0.9719	1.0261
coronary artery calcification	0.2483	1.2818	0.0405	< 0.0001	1.1840	1.3877

l2)

	coef	HR	se	p	lower	upper
rs2736100	−0.1259	0.8817	0.1071	0.2395	0.7148	1.0875
sex	−0.4747	0.6221	0.2802	0.0902	0.3592	1.0773
age	0.0553	1.0569	0.0126	< 0.0001	1.0310	1.0834
total cholesterol	−0.0014	0.9986	0.0065	0.8288	0.9860	1.0113
HDL	0.0043	1.0043	0.0080	0.5895	0.9887	1.0201
LDL	−0.0009	0.9991	0.0065	0.8897	0.9865	1.0119
triglycerides	0.0013	1.0013	0.0011	0.2073	0.9993	1.0034
diabetes	0.0903	1.0945	0.1197	0.4508	0.8656	1.3838
systolic bloodpressure	0.0064	1.0064	0.0051	0.2058	0.9965	1.0165
diastolic bloodpressure	−0.0087	0.9913	0.0098	0.3729	0.9724	1.0105
CRP	−0.0349	0.9657	0.1573	0.8242	0.7094	1.3144
BMI	0.0588	1.0606	0.0403	0.1441	0.9801	1.1476
physical activity	0.2413	1.2728	0.1541	0.1175	0.9410	1.7217
waist circumference	−0.0209	0.9793	0.0149	0.1596	0.9512	1.0083
coronary artery calcification	0.2757	1.3175	0.0404	< 0.0001	1.2172	1.4260

l3)

	coef	HR	se	p	lower	upper
rs2736100	0.1250	1.1332	0.1377	0.364	0.8651	1.4843
sex	0.1398	1.1501	0.2901	0.6297	0.6514	2.0306
age	0.0419	1.0428	0.0161	0.0094	1.0103	1.0763
total cholesterol	−0.0015	0.9985	0.0065	0.8165	0.9858	1.0114
HDL	−0.0073	0.9927	0.0099	0.4612	0.9737	1.0121
LDL	−0.0001	0.9999	0.0068	0.9918	0.9867	1.0133
triglycerides	0.0007	1.0007	0.0011	0.4946	0.9986	1.0029
diabetes	0.0093	1.0093	0.1750	0.9578	0.7163	1.4222
systolic bloodpressure	0.0073	1.0073	0.0076	0.3417	0.9923	1.0225
diastolic bloodpressure	−0.0034	0.9966	0.0139	0.8062	0.9698	1.0242
CRP	−0.0101	0.9899	0.1090	0.9261	0.7995	1.2257
BMI	−0.0317	0.9688	0.0455	0.4865	0.8861	1.0592
physical activity	0.0974	1.1023	0.2054	0.6352	0.7371	1.6487
waist circumference	0.0035	1.0035	0.0183	0.8495	0.9681	1.0401
coronary artery calcification	0.2997	1.3495	0.0512	< 0.0001	1.2207	1.4918

m1)

	coef	HR	se	p	lower	upper
rs2736100	0.1001	1.1053	0.1433	0.485	0.8346	1.4637
sex	0.1385	1.1485	0.2810	0.6222	0.6621	1.9922
age	0.0419	1.0428	0.0168	0.0128	1.0090	1.0778
total cholesterol	0.0085	1.0086	0.0077	0.2664	0.9935	1.0239
HDL	−0.0073	0.9927	0.0097	0.4517	0.9740	1.0118
LDL	−0.0092	0.9909	0.0078	0.238	0.9759	1.0061
triglycerides	0.0002	1.0002	0.0013	0.8861	0.9976	1.0028
diabetes	0.3124	1.3668	0.1885	0.0975	0.9445	1.9777
smoking	0.1909	1.2104	0.1400	0.1727	0.9199	1.5926
systolic bloodpressure	0.0154	1.0155	0.0070	0.027	1.0018	1.0295
diastolic bloodpressure	−0.0161	0.9841	0.0154	0.2969	0.9548	1.0142
CRP	0.1752	1.1915	0.1535	0.2537	0.8819	1.6098
BMI	0.0029	1.0029	0.0487	0.9522	0.9116	1.1034
physical activity	0.4893	1.6312	0.2121	0.021	1.0764	2.4719
coronary artery calcification	0.2933	1.3408	0.0507	< 0.0001	1.2139	1.4809

m2)

	coef	HR	se	p	lower	upper
rs2736100	0.0267	1.0270	0.0765	0.7274	0.8840	1.1933
sex	−0.1107	0.8952	0.1396	0.4277	0.6809	1.1769
age	0.0632	1.0652	0.0092	< 0.0001	1.0462	1.0846
total cholesterol	−0.0047	0.9953	0.0042	0.2627	0.9870	1.0036
HDL	−0.0047	0.9953	0.0057	0.4151	0.9842	1.0066
LDL	0.0008	1.0008	0.0043	0.8489	0.9924	1.0093
triglycerides	0.0013	1.0013	0.0007	0.086	0.9998	1.0027
diabetes	0.1144	1.1212	0.0844	0.1755	0.9502	1.3229
smoking	0.1495	1.1612	0.0810	0.0651	0.9907	1.3611
systolic bloodpressure	0.0049	1.0049	0.0038	0.1956	0.9975	1.0124
diastolic bloodpressure	−0.0003	0.9997	0.0072	0.9668	0.9857	1.0139
CRP	−0.0352	0.9654	0.0839	0.6746	0.8190	1.1379
BMI	−0.0002	0.9998	0.0140	0.9868	0.9728	1.0275
physical activity	0.0360	1.0366	0.1108	0.7454	0.8342	1.2882
coronary artery calcification	0.2653	1.3039	0.0288	< 0.0001	1.2322	1.3797

n1)

	coef	HR	se	p	lower	upper
rs2736100	0.1153	1.1222	0.1545	0.4557	0.8290	1.5191
sex	0.0234	1.0237	0.3377	0.9448	0.5281	1.9844
age	0.0633	1.0654	0.0195	0.0011	1.0255	1.1068
total cholesterol	0.0109	1.0110	0.0081	0.1766	0.9951	1.0272
HDL	−0.0162	0.9840	0.0104	0.1211	0.9640	1.0043
LDL	−0.0140	0.9861	0.0081	0.0828	0.9706	1.0018
triglycerides	−0.0005	0.9995	0.0015	0.7114	0.9965	1.0024
diabetes	0.2220	1.2486	0.2375	0.3498	0.7839	1.9888
smoking	0.2294	1.2578	0.1464	0.1171	0.9441	1.6758
systolic bloodpressure	0.0071	1.0071	0.0076	0.353	0.9922	1.0222
diastolic bloodpressure	−0.0016	0.9984	0.0164	0.9227	0.9669	1.0309
CRP	−0.0226	0.9776	0.1194	0.8498	0.7737	1.2354
physical activity	0.5878	1.8001	0.2347	0.0123	1.1363	2.8517
waist circumference	−0.0233	0.9770	0.0184	0.2053	0.9424	1.0128
coronary artery calcification	0.3762	1.4568	0.0602	< 0.0001	1.2946	1.6394

n2)

	coef	HR	se	p	lower	upper
rs2736100	0.0202	1.0204	0.0755	0.7889	0.8800	1.1832
sex	−0.1623	0.8502	0.1404	0.2477	0.6456	1.1195
age	0.0585	1.0602	0.0089	< 0.0001	1.0419	1.0789
total cholesterol	−0.0053	0.9947	0.0041	0.1954	0.9867	1.0027
HDL	−0.0019	0.9981	0.0056	0.73	0.9873	1.0090
LDL	0.0019	1.0019	0.0042	0.6538	0.9937	1.0102
triglycerides	0.0015	1.0015	0.0007	0.0333	1.0001	1.0029
diabetes	0.1306	1.1395	0.0828	0.1149	0.9688	1.3402
smoking	0.1669	1.1817	0.0789	0.0344	1.0123	1.3793
systolic bloodpressure	0.0062	1.0062	0.0038	0.1006	0.9988	1.0136
diastolic bloodpressure	−0.0057	0.9943	0.0072	0.4217	0.9804	1.0083
CRP	0.0044	1.0044	0.0850	0.9585	0.8502	1.1866
physical activity	0.0436	1.0445	0.1087	0.6887	0.8440	1.2926
waist circumference	−0.0041	0.9959	0.0057	0.4668	0.9848	1.0070
coronary artery calcification	0.2507	1.2850	0.0275	< 0.0001	1.2175	1.3561

S4E Table. Results of Cox regression models for rs2487999.

coef: coefficient, HR: Hazard Ratio, se: Standard error,
lower/upper: lower/upper boundarie of the 95% confidence interval
a) crude, b) adjusted, c1) young age, c2) older age, d1) male, d2) female,
e1) low hsCRP, e2) intermediate hsCRP, e3) high hsCRP, f1) low total cholesterol,
f2) high total cholesterol, g1) low LDL, g2) high LDL, h1) normal HDL, h2) high HDL,
i1) low triglycerides, i2) high triglycerides, j1) ideal blood pressure,
j2) normal/high normal blood pressure, j3) hypertension, k1) no diabetes, k2) diabetes,
l1) never smoker, l2) former smoker, l3) current smoker, m1) normal waist circumference,
m2) high waist circumference, n1) normal BMI, n2) high BMI

a)

	coef	HR	se	p	lower	upper
rs2487999	−0.1118	0.8942	0.102	0.273	0.7321	1.0921

b)

	coef	HR	se	p	lower	upper
rs2487999	0.0326	1.0331	0.1099	0.7668	0.8329	1.2815
sex	−0.1135	0.8927	0.1487	0.4454	0.6670	1.1948
age	0.0594	1.0612	0.0081	< 0.0001	1.0445	1.0782
total cholesterol	−0.0023	0.9977	0.0038	0.548	0.9904	1.0052
HDL	−0.0044	0.9956	0.0050	0.3783	0.9860	1.0054
LDL	−0.0009	0.9991	0.0038	0.8072	0.9916	1.0066
triglycerides	0.0011	1.0011	0.0006	0.0887	0.9998	1.0024
diabetes	0.1562	1.1691	0.0800	0.0508	0.9995	1.3675
smoking	0.1822	1.1999	0.0695	0.0088	1.0470	1.3750
systolic bloodpressure	0.0071	1.0071	0.0034	0.0363	1.0005	1.0138
diastolic bloodpressure	−0.0050	0.9950	0.0065	0.4475	0.9824	1.0079
CRP	0.0008	1.0008	0.0702	0.991	0.8722	1.1483
BMI	0.0048	1.0048	0.0221	0.8297	0.9621	1.0493
physical activity	0.1608	1.1744	0.0983	0.102	0.9686	1.4240
waist circumference	−0.0084	0.9916	0.0087	0.3326	0.9748	1.0087
coronary artery calcification	0.2753	1.3170	0.0250	< 0.0001	1.2541	1.3830

c1)

	coef	HR	se	p	lower	upper
rs2487999	0.0807	1.0841	0.2489	0.7457	0.6655	1.7658
sex	−0.1042	0.9011	0.2864	0.716	0.5140	1.5796
total cholesterol	0.0021	1.0021	0.0068	0.7554	0.9889	1.0155
HDL	−0.0143	0.9858	0.0102	0.1608	0.9664	1.0057
LDL	−0.0017	0.9983	0.0069	0.8078	0.9849	1.0119
triglycerides	0.0003	1.0003	0.0013	0.8328	0.9977	1.0029
diabetes	−0.0017	0.9983	0.1974	0.9932	0.6780	1.4701
smoking	0.3011	1.3513	0.1346	0.0253	1.0380	1.7591
systolic bloodpressure	−0.0043	0.9957	0.0089	0.6267	0.9784	1.0133
diastolic bloodpressure	0.0193	1.0195	0.0163	0.2354	0.9875	1.0525
CRP	0.1017	1.1070	0.1656	0.5393	0.8002	1.5315
BMI	0.0089	1.0089	0.0468	0.8498	0.9205	1.1057
physical activity	0.2082	1.2314	0.2139	0.3303	0.8098	1.8726
waist circumference	−0.0140	0.9861	0.0183	0.4435	0.9513	1.0221
coronary artery calcification	0.2919	1.3389	0.0463	< 0.0001	1.2227	1.4662

c2)

	coef	HR	se	p	lower	upper
rs2487999	−0.0123	0.9877	0.1231	0.9201	0.7761	1.2571
sex	0.3425	1.4085	0.1747	0.0499	1.0002	1.9834
total cholesterol	−0.0045	0.9956	0.0042	0.2905	0.9874	1.0038
HDL	−0.0004	0.9996	0.0046	0.9324	0.9906	1.0087
LDL	0.0000	1.0000	0.0043	0.9932	0.9916	1.0086
triglycerides	0.0011	1.0011	0.0007	0.1006	0.9998	1.0025
diabetes	0.1827	1.2005	0.0886	0.0393	1.0090	1.4283
smoking	0.0809	1.0843	0.0820	0.3235	0.9234	1.2733
systolic bloodpressure	0.0138	1.0139	0.0036	0.0001	1.0068	1.0210
diastolic bloodpressure	−0.0180	0.9821	0.0070	0.0104	0.9686	0.9958
CRP	−0.0057	0.9943	0.0736	0.9384	0.8607	1.1487
BMI	−0.0105	0.9896	0.0257	0.6843	0.9409	1.0408
physical activity	0.1398	1.1500	0.1112	0.2089	0.9247	1.4302
waist circumference	−0.0012	0.9988	0.0100	0.9075	0.9795	1.0186
coronary artery calcification	0.2886	1.3346	0.0294	< 0.0001	1.2599	1.4137

d1)

	coef	HR	se	p	lower	upper
rs2487999	0.1006	1.1058	0.1351	0.4567	0.8485	1.4411
age	0.0438	1.0447	0.0096	< 0.0001	1.0252	1.0646
total cholesterol	−0.0060	0.9940	0.0048	0.2101	0.9847	1.0034
HDL	0.0018	1.0018	0.0061	0.7691	0.9898	1.0140
LDL	0.0028	1.0028	0.0049	0.5633	0.9933	1.0125
triglycerides	0.0015	1.0015	0.0008	0.053	1.0000	1.0030
diabetes	0.1764	1.1929	0.0911	0.0529	0.9978	1.4261
smoking	0.1104	1.1168	0.0892	0.2155	0.9377	1.3300
systolic bloodpressure	0.0048	1.0048	0.0042	0.2609	0.9965	1.0132
diastolic bloodpressure	−0.0025	0.9975	0.0080	0.7544	0.9821	1.0132
CRP	0.0016	1.0016	0.0787	0.9837	0.8585	1.1686
BMI	0.0355	1.0361	0.0291	0.2225	0.9787	1.0969
physical activity	0.1648	1.1792	0.1208	0.1723	0.9306	1.4941
waist circumference	−0.0130	0.9871	0.0112	0.246	0.9656	1.0090
coronary artery calcification	0.2974	1.3463	0.0332	< 0.0001	1.2614	1.4370

d2)

	coef	HR	se	p	lower	upper
rs2487999	−0.1187	0.8880	0.1905	0.533	0.6114	1.2899
age	0.0989	1.1040	0.0152	< 0.0001	1.0716	1.1373
total cholesterol	0.0017	1.0017	0.0056	0.7647	0.9908	1.0126
HDL	−0.0135	0.9866	0.0081	0.0957	0.9710	1.0024
LDL	−0.0056	0.9944	0.0057	0.3247	0.9834	1.0056
triglycerides	0.0012	1.0012	0.0013	0.3768	0.9986	1.0038
diabetes	0.1199	1.1274	0.1713	0.4839	0.8059	1.5773
smoking	0.3338	1.3962	0.1096	0.0023	1.1264	1.7306
systolic bloodpressure	0.0092	1.0092	0.0057	0.1087	0.9980	1.0206
diastolic bloodpressure	−0.0063	0.9937	0.0117	0.5905	0.9712	1.0168
CRP	0.0123	1.0124	0.1478	0.9337	0.7578	1.3524
BMI	−0.0289	0.9715	0.0349	0.4072	0.9073	1.0402
physical activity	0.1504	1.1623	0.1708	0.3786	0.8316	1.6245
waist circumference	−0.0068	0.9933	0.0141	0.6321	0.9662	1.0211
coronary artery calcification	0.2360	1.2661	0.0386	< 0.0001	1.1738	1.3656

e1)

	coef	HR	se	p	lower	upper
rs2487999	0.1165	1.1235	0.1387	0.4011	0.8561	1.4745
sex	−0.1741	0.8402	0.1813	0.3371	0.5889	1.1988
age	0.0684	1.0708	0.0098	< 0.0001	1.0504	1.0915
total cholesterol	−0.0002	0.9998	0.0043	0.9704	0.9914	1.0084
HDL	−0.0063	0.9937	0.0058	0.2741	0.9825	1.0050
LDL	−0.0036	0.9964	0.0044	0.418	0.9878	1.0051
triglycerides	0.0011	1.0011	0.0007	0.1393	0.9996	1.0025
diabetes	0.1337	1.1431	0.1012	0.1861	0.9375	1.3938
smoking	0.1784	1.1953	0.0859	0.0379	1.0101	1.4144
systolic bloodpressure	0.0077	1.0077	0.0043	0.0703	0.9994	1.0162
diastolic bloodpressure	−0.0041	0.9959	0.0083	0.6181	0.9798	1.0122
BMI	0.0382	1.0390	0.0280	0.1716	0.9836	1.0975
physical activity	0.1804	1.1977	0.1183	0.1272	0.9499	1.5102
waist circumference	−0.0169	0.9832	0.0105	0.1064	0.9633	1.0036
coronary artery calcification	0.2719	1.3125	0.0301	< 0.0001	1.2374	1.3922

e2)

	coef	HR	se	p	lower	upper
rs2487999	0.526	1.6921	0.3674	0.1523	0.8235	3.4769
sex	0.6399	1.8963	0.4479	0.1531	0.7883	4.562
age	0.1025	1.108	0.0254	0.0001	1.0542	1.1644
total cholesterol	−0.0049	0.9951	0.013	0.7044	0.97	1.0208
HDL	−0.0115	0.9886	0.0167	0.4907	0.9567	1.0214
LDL	0.0043	1.0043	0.0124	0.7301	0.9801	1.0291
triglycerides	−0.004	0.996	0.0026	0.1321	0.9909	1.0012
diabetes	0.4246	1.529	0.2003	0.034	1.0325	2.2643
smoking	0.1846	1.2028	0.212	0.3837	0.7939	1.8222
systolic bloodpressure	0	1	0.0093	0.9957	0.9819	1.0183
diastolic bloodpressure	0.0048	1.0048	0.0171	0.7776	0.9718	1.039
BMI	−0.1351	0.8736	0.073	0.0641	0.7571	1.008
physical activity	−0.5887	0.555	0.2833	0.0377	0.3185	0.9671
waist circumference	0.0641	1.0662	0.0294	0.0291	1.0066	1.1295
coronary artery calcification	0.2707	1.3109	0.0746	0.0003	1.1326	1.5172

e3)

	coef	HR	se	p	lower	upper
rs2487999	−0.3173	0.7281	0.2168	0.1434	0.4760	1.1137
sex	−0.2669	0.7658	0.3563	0.4538	0.3809	1.5394
age	0.0086	1.0086	0.0185	0.6439	0.9726	1.0459
total cholesterol	−0.0048	0.9952	0.0066	0.46	0.9825	1.0080
HDL	0.0010	1.0010	0.0033	0.7719	0.9945	1.0074
LDL	0.0031	1.0031	0.0069	0.6553	0.9897	1.0166
triglycerides	0.0022	1.0022	0.0013	0.0791	0.9997	1.0047
diabetes	0.1792	1.1962	0.1735	0.3017	0.8514	1.6808
smoking	0.1402	1.1505	0.1588	0.3773	0.8428	1.5706
systolic bloodpressure	0.0063	1.0063	0.0073	0.3887	0.9920	1.0209
diastolic bloodpressure	−0.0177	0.9825	0.0149	0.2372	0.9542	1.0117
BMI	−0.0368	0.9638	0.0486	0.448	0.8763	1.0601
physical activity	0.4597	1.5836	0.2423	0.0578	0.9849	2.5464
waist circumference	−0.0144	0.9857	0.0204	0.4789	0.9470	1.0259
coronary artery calcification	0.2879	1.3336	0.0587	< 0.0001	1.1887	1.4963

f1)

	coef	HR	se	p	lower	upper
rs2487999	0.5231	1.6873	0.2337	0.0252	1.0672	2.6678
sex	0.3703	1.4481	0.2724	0.1741	0.8490	2.4701
age	0.0768	1.0798	0.0161	< 0.0001	1.0463	1.1144
HDL	−0.0089	0.9912	0.0086	0.3	0.9747	1.0079
LDL	−0.0043	0.9957	0.0048	0.369	0.9865	1.0051
triglycerides	0.0004	1.0004	0.0013	0.739	0.9978	1.0030
diabetes	0.2366	1.2670	0.1524	0.1205	0.9398	1.7080
smoking	0.0006	1.0006	0.1329	0.9962	0.7711	1.2985
systolic bloodpressure	0.0031	1.0031	0.0067	0.6375	0.9901	1.0163
diastolic bloodpressure	−0.0026	0.9974	0.0130	0.8397	0.9723	1.0231
CRP	−0.0547	0.9467	0.1224	0.6548	0.7448	1.2034
BMI	−0.1013	0.9037	0.0438	0.0208	0.8293	0.9847
physical activity	0.2779	1.3204	0.1922	0.1482	0.9059	1.9244
waist circumference	0.0268	1.0272	0.0162	0.098	0.9951	1.0603
coronary artery calcification	0.3024	1.3531	0.0478	< 0.0001	1.2320	1.4861

f2)

	coef	HR	se	p	lower	upper
rs2487999	−0.1384	0.8708	0.1242	0.2654	0.6826	1.1108
sex	−0.3060	0.7364	0.1772	0.0842	0.5203	1.0422
age	0.0557	1.0572	0.0094	< 0.0001	1.0379	1.0770
HDL	−0.0059	0.9941	0.0044	0.1784	0.9856	1.0027
LDL	−0.0031	0.9969	0.0019	0.1147	0.9931	1.0007
triglycerides	0.0007	1.0007	0.0005	0.1364	0.9998	1.0017
diabetes	0.1211	1.1287	0.0956	0.2052	0.9359	1.3613
smoking	0.2610	1.2982	0.0827	0.0016	1.1040	1.5266
systolic bloodpressure	0.0078	1.0078	0.0040	0.0509	1.0000	1.0157
diastolic bloodpressure	−0.0063	0.9937	0.0076	0.4073	0.9789	1.0087
CRP	0.0133	1.0133	0.0853	0.8765	0.8573	1.1977
BMI	0.0474	1.0486	0.0255	0.0633	0.9974	1.1024
physical activity	0.1238	1.1318	0.1167	0.2887	0.9004	1.4225
waist circumference	−0.0220	0.9782	0.0102	0.0302	0.9589	0.9979
coronary artery calcification	0.2665	1.3054	0.0297	< 0.0001	1.2315	1.3837

g1)

	coef	HR	se	p	lower	upper
rs2487999	0.1571	1.1701	0.2494	0.5288	0.7177	1.9078
sex	0.1481	1.1596	0.2965	0.6175	0.6485	2.0735
age	0.0830	1.0865	0.0177	< 0.0001	1.0495	1.1248
total cholesterol	−0.0022	0.9978	0.0046	0.6406	0.9888	1.0070
HDL	−0.0014	0.9986	0.0074	0.8489	0.9843	1.0131
triglycerides	0.0018	1.0018	0.0007	0.0131	1.0004	1.0032
diabetes	0.2145	1.2392	0.1675	0.2004	0.8924	1.7207
smoking	0.1848	1.2030	0.1433	0.1974	0.9083	1.5932
systolic bloodpressure	0.0014	1.0014	0.0073	0.8497	0.9872	1.0157
diastolic bloodpressure	−0.0062	0.9938	0.0139	0.6534	0.9672	1.0212
CRP	−0.2730	0.7611	0.2300	0.2352	0.4849	1.1945
BMI	−0.0733	0.9293	0.0463	0.1132	0.8487	1.0176
physical activity	0.4977	1.6450	0.2104	0.018	1.0890	2.4848
waist circumference	0.0207	1.0209	0.0174	0.2354	0.9866	1.0563
coronary artery calcification	0.2675	1.3066	0.0515	< 0.0001	1.1812	1.4454

g2)

	coef	HR	se	p	lower	upper
rs2487999	0.0083	1.0083	0.1224	0.946	0.7933	1.2816
sex	−0.1872	0.8293	0.1718	0.2758	0.5922	1.1612
age	0.0543	1.0558	0.0092	< 0.0001	1.0369	1.0751
total cholesterol	−0.0032	0.9968	0.0019	0.0823	0.9931	1.0004
HDL	−0.0040	0.9960	0.0047	0.3913	0.9870	1.0051
triglycerides	0.0010	1.0010	0.0007	0.169	0.9996	1.0023
diabetes	0.1334	1.1427	0.0923	0.1482	0.9537	1.3692
smoking	0.1999	1.2213	0.0801	0.0126	1.0438	1.4289
systolic bloodpressure	0.0078	1.0078	0.0039	0.0437	1.0002	1.0155
diastolic bloodpressure	−0.0042	0.9958	0.0075	0.5747	0.9813	1.0105
CRP	0.0889	1.0930	0.0847	0.294	0.9257	1.2905
BMI	0.0256	1.0259	0.0250	0.3058	0.9769	1.0774
physical activity	0.0807	1.0840	0.1127	0.4741	0.8692	1.3518
waist circumference	−0.0168	0.9833	0.0099	0.0894	0.9645	1.0026
coronary artery calcification	0.2766	1.3186	0.0290	< 0.0001	1.2458	1.3957

h1)

	coef	HR	se	p	lower	upper
rs2487999	0.0158	1.0159	0.1297	0.9029	0.7879	1.3100
sex	−0.1303	0.8778	0.1729	0.451	0.6256	1.2318
age	0.0700	1.0725	0.0098	< 0.0001	1.0521	1.0934
total cholesterol	−0.0019	0.9981	0.0035	0.5855	0.9913	1.0049
LDL	−0.0005	0.9995	0.0036	0.8913	0.9925	1.0066
triglycerides	−0.0001	0.9999	0.0008	0.8678	0.9982	1.0015
diabetes	0.1717	1.1873	0.0983	0.0807	0.9793	1.4396
smoking	0.2383	1.2691	0.0826	0.0039	1.0794	1.4922
systolic bloodpressure	0.0094	1.0095	0.0039	0.0153	1.0018	1.0172
diastolic bloodpressure	−0.0040	0.9960	0.0076	0.5971	0.9813	1.0109
CRP	−0.0791	0.9239	0.0993	0.4256	0.7605	1.1225
BMI	0.0200	1.0202	0.0260	0.4428	0.9695	1.0735
physical activity	0.1797	1.1969	0.1147	0.117	0.9560	1.4985
waist circumference	−0.0072	0.9928	0.0104	0.4888	0.9727	1.0133
coronary artery calcification	0.2551	1.2905	0.0285	< 0.0001	1.2205	1.3646

h2)

	coef	HR	se	p	lower	upper
rs2487999	0.0392	1.0400	0.2098	0.8518	0.6893	1.5690
sex	−0.2672	0.7655	0.2798	0.3396	0.4424	1.3248
age	0.0344	1.0350	0.0146	0.0188	1.0057	1.0652
total cholesterol	−0.0047	0.9953	0.0058	0.4189	0.9840	1.0067
LDL	0.0007	1.0007	0.0063	0.9106	0.9885	1.0131
triglycerides	0.0021	1.0021	0.0008	0.0114	1.0005	1.0037
diabetes	0.0700	1.0725	0.1417	0.6214	0.8124	1.4159
smoking	0.0438	1.0447	0.1347	0.7453	0.8023	1.3604
systolic bloodpressure	0.0007	1.0007	0.0069	0.9165	0.9873	1.0143
diastolic bloodpressure	−0.0063	0.9937	0.0132	0.6327	0.9683	1.0198
CRP	0.1736	1.1895	0.1153	0.1324	0.9489	1.4913
BMI	−0.0327	0.9678	0.0426	0.4425	0.8902	1.0521
physical activity	0.1160	1.1230	0.1917	0.545	0.7713	1.6352
waist circumference	−0.0158	0.9844	0.0161	0.3288	0.9537	1.0160
coronary artery calcification	0.3328	1.3949	0.0526	< 0.0001	1.2582	1.5463

i1)

	coef	HR	se	p	lower	upper
rs2487999	−0.1158	0.8907	0.1454	0.4258	0.6698	1.1843
sex	−0.0956	0.9088	0.2004	0.6332	0.6136	1.3460
age	0.0728	1.0756	0.0114	< 0.0001	1.0519	1.0998
total cholesterol	−0.0012	0.9988	0.0052	0.8123	0.9886	1.0090
HDL	−0.0024	0.9976	0.0064	0.7098	0.9853	1.0102
LDL	−0.0029	0.9971	0.0054	0.5834	0.9866	1.0076
diabetes	0.0331	1.0337	0.1263	0.7932	0.8070	1.3240
smoking	0.3058	1.3577	0.0943	0.0012	1.1286	1.6333
systolic bloodpressure	0.0047	1.0047	0.0046	0.3147	0.9956	1.0139
diastolic bloodpressure	0.0125	1.0126	0.0091	0.1682	0.9947	1.0308
CRP	−0.0519	0.9494	0.0891	0.5599	0.7973	1.1305
BMI	0.0284	1.0288	0.0312	0.3631	0.9678	1.0937
physical activity	0.3492	1.4180	0.1335	0.0089	1.0914	1.8423
waist circumference	−0.0147	0.9854	0.0119	0.2177	0.9627	1.0087
coronary artery calcification	0.2777	1.3201	0.0330	< 0.0001	1.2375	1.4082

i2)

	coef	HR	se	p	lower	upper
rs2487999	0.1822	1.1998	0.1677	0.2773	0.8637	1.6667
sex	−0.1363	0.8726	0.2258	0.5461	0.5606	1.3583
age	0.0447	1.0457	0.0116	0.0001	1.0222	1.0698
total cholesterol	0.0017	1.0017	0.0034	0.6089	0.9951	1.0084
HDL	−0.0173	0.9828	0.0070	0.0135	0.9694	0.9964
LDL	−0.0036	0.9964	0.0035	0.3057	0.9895	1.0033
diabetes	0.2556	1.2912	0.1064	0.0163	1.0483	1.5905
smoking	0.0590	1.0608	0.1052	0.5748	0.8632	1.3036
systolic bloodpressure	0.0104	1.0104	0.0049	0.0345	1.0008	1.0202
diastolic bloodpressure	−0.0253	0.9750	0.0097	0.0093	0.9566	0.9938
CRP	0.1166	1.1237	0.1339	0.3839	0.8643	1.4609
BMI	−0.0222	0.9780	0.0326	0.495	0.9175	1.0425
physical activity	−0.1191	0.8877	0.1457	0.4137	0.6672	1.1811
waist circumference	−0.0002	0.9998	0.0129	0.9852	0.9748	1.0254
coronary artery calcification	0.2669	1.3059	0.0385	< 0.0001	1.2109	1.4084

j1)

	coef	HR	se	p	lower	upper
rs2487999	−0.1400	0.8693	0.2879	0.6267	0.4944	1.5285
sex	−0.1040	0.9012	0.3636	0.7749	0.4419	1.8379
age	0.0576	1.0592	0.0188	0.0022	1.0210	1.0989
total cholesterol	0.0141	1.0142	0.0108	0.1897	0.9930	1.0358
HDL	−0.0240	0.9763	0.0132	0.0688	0.9514	1.0018
LDL	−0.0144	0.9857	0.0103	0.1599	0.9660	1.0057
triglycerides	0.0024	1.0024	0.0025	0.3196	0.9976	1.0073
diabetes	−0.0452	0.9558	0.2245	0.8405	0.6156	1.4840
smoking	0.2273	1.2552	0.1759	0.1962	0.8892	1.7718
CRP	0.1246	1.1327	0.1482	0.4004	0.8472	1.5145
BMI	0.0084	1.0084	0.0597	0.8881	0.8971	1.1336
physical activity	0.2454	1.2781	0.2642	0.353	0.7616	2.1450
waist circumference	−0.0166	0.9836	0.0236	0.4831	0.9391	1.0302
coronary artery calcification	0.3632	1.4380	0.0619	< 0.0001	1.2737	1.6234

j2)

	coef	HR	se	p	lower	upper
rs2487999	0.0106	1.0107	0.1807	0.9531	0.7092	1.4403
sex	−0.5802	0.5598	0.2638	0.0279	0.3338	0.9388
age	0.0711	1.0737	0.0125	< 0.0001	1.0476	1.1004
total cholesterol	0.0012	1.0012	0.0054	0.8188	0.9906	1.0120
HDL	−0.0095	0.9906	0.0083	0.2546	0.9745	1.0069
LDL	−0.0036	0.9964	0.0055	0.514	0.9856	1.0073
triglycerides	0.0004	1.0004	0.0012	0.7135	0.9981	1.0028
diabetes	0.2612	1.2985	0.1320	0.0477	1.0026	1.6818
smoking	0.2387	1.2696	0.1172	0.0418	1.0089	1.5976
CRP	0.0650	1.0671	0.1644	0.6927	0.7732	1.4728
BMI	0.0656	1.0678	0.0369	0.0756	0.9933	1.1480
physical activity	0.2184	1.2441	0.1642	0.1834	0.9018	1.7164
waist circumference	−0.0349	0.9657	0.0147	0.0172	0.9383	0.9938
coronary artery calcification	0.2130	1.2373	0.0395	< 0.0001	1.1452	1.3368

j3)

	coef	HR	se	p	lower	upper
rs2487999	0.0923	1.0967	0.1611	0.5668	0.7998	1.5038
sex	0.1480	1.1595	0.2065	0.4737	0.7735	1.7381
age	0.0619	1.0639	0.0115	< 0.0001	1.0401	1.0882
total cholesterol	−0.0075	0.9925	0.0044	0.0883	0.9839	1.0011
HDL	0.0019	1.0019	0.0021	0.3649	0.9977	1.0062
LDL	0.0028	1.0028	0.0047	0.5555	0.9936	1.0121
triglycerides	0.0015	1.0015	0.0007	0.033	1.0001	1.0028
diabetes	0.1460	1.1572	0.1133	0.1974	0.9268	1.4449
smoking	0.1663	1.1810	0.1024	0.1044	0.9662	1.4436
CRP	−0.0504	0.9508	0.0959	0.5989	0.7879	1.1474
BMI	−0.0343	0.9663	0.0321	0.2854	0.9074	1.0290
physical activity	0.0611	1.0630	0.1408	0.6643	0.8067	1.4008
waist circumference	0.0073	1.0074	0.0124	0.5546	0.9831	1.0322
coronary artery calcification	0.2971	1.3460	0.0379	< 0.0001	1.2495	1.4499

k1)

	coef	HR	se	p	lower	upper
rs2487999	−0.0021	0.9979	0.1291	0.9869	0.7748	1.2852
sex	−0.1079	0.8978	0.1705	0.5271	0.6427	1.2541
age	0.0650	1.0672	0.0094	< 0.0001	1.0478	1.0869
total cholesterol	−0.0001	0.9999	0.0042	0.9849	0.9917	1.0082
HDL	−0.0073	0.9928	0.0058	0.2127	0.9815	1.0042
LDL	−0.0022	0.9978	0.0043	0.6108	0.9895	1.0063
triglycerides	0.0002	1.0002	0.0009	0.838	0.9984	1.0019
smoking	0.2922	1.3393	0.0794	0.0002	1.1463	1.5650
systolic bloodpressure	0.0040	1.0040	0.0042	0.3363	0.9958	1.0122
diastolic bloodpressure	0.0035	1.0036	0.0080	0.6581	0.9879	1.0195
CRP	−0.1111	0.8948	0.1141	0.3299	0.7155	1.1190
BMI	0.0240	1.0242	0.0266	0.3678	0.9722	1.0790
physical activity	0.2037	1.2260	0.1138	0.0734	0.9809	1.5323
waist circumference	−0.0090	0.9911	0.0102	0.3788	0.9715	1.0111
coronary artery calcification	0.2628	1.3006	0.0283	< 0.0001	1.2305	1.3747

k2)

	coef	HR	se	p	lower	upper
rs2487999	0.0616	1.0636	0.2141	0.7735	0.6991	1.6182
sex	−0.3769	0.6860	0.3225	0.2424	0.3646	1.2905
age	0.0476	1.0487	0.0169	0.0048	1.0146	1.0840
total cholesterol	−0.0054	0.9946	0.0092	0.552	0.9769	1.0126
HDL	0.0054	1.0054	0.0102	0.5953	0.9856	1.0256
LDL	0.0014	1.0014	0.0092	0.8808	0.9834	1.0197
triglycerides	0.0021	1.0021	0.0012	0.0636	0.9999	1.0044
smoking	−0.1030	0.9021	0.1552	0.5068	0.6655	1.2229
systolic bloodpressure	0.0109	1.0109	0.0061	0.0754	0.9989	1.0231
diastolic bloodpressure	−0.0186	0.9816	0.0117	0.1125	0.9593	1.0044
CRP	0.1092	1.1154	0.0831	0.1887	0.9478	1.3127
BMI	−0.0167	0.9834	0.0444	0.7063	0.9015	1.0728
physical activity	−0.0933	0.9109	0.2014	0.6433	0.6138	1.3519
waist circumference	−0.0160	0.9841	0.0178	0.3688	0.9504	1.0191
coronary artery calcification	0.3171	1.3731	0.0565	< 0.0001	1.2293	1.5338

l1)

	coef	HR	se	p	lower	upper
rs2487999	−0.2136	0.8077	0.1680	0.2037	0.5811	1.1227
sex	−0.1547	0.8567	0.2335	0.5075	0.5421	1.3538
age	0.0878	1.0917	0.0155	< 0.0001	1.0591	1.1253
total cholesterol	−0.0038	0.9962	0.0068	0.5779	0.9831	1.0096
HDL	−0.0097	0.9904	0.0091	0.2861	0.9730	1.0081
LDL	−0.0016	0.9984	0.0068	0.8152	0.9851	1.0119
triglycerides	0.0015	1.0015	0.0016	0.3491	0.9984	1.0045
diabetes	0.3676	1.4442	0.1399	0.0086	1.0979	1.8998
systolic bloodpressure	0.0087	1.0087	0.0060	0.1497	0.9969	1.0206
diastolic bloodpressure	−0.0058	0.9942	0.0119	0.6259	0.9713	1.0177
CRP	0.0199	1.0201	0.1098	0.8558	0.8227	1.2650
BMI	−0.0089	0.9911	0.0349	0.7986	0.9256	1.0613
physical activity	0.0195	1.0197	0.1702	0.9087	0.7305	1.4234
waist circumference	−0.0029	0.9971	0.0140	0.8363	0.9701	1.0249
coronary artery calcification	0.2559	1.2917	0.0410	< 0.0001	1.1920	1.3997

l2)

	coef	HR	se	p	lower	upper
rs2487999	−0.0762	0.9267	0.1685	0.6512	0.6660	1.2893
sex	−0.4532	0.6356	0.2820	0.108	0.3657	1.1046
age	0.0550	1.0565	0.0127	< 0.0001	1.0306	1.0830
total cholesterol	−0.0016	0.9984	0.0064	0.8055	0.9859	1.0111
HDL	0.0045	1.0045	0.0080	0.575	0.9889	1.0203
LDL	−0.0008	0.9992	0.0065	0.9066	0.9866	1.0120
triglycerides	0.0013	1.0014	0.0010	0.1956	0.9993	1.0034
diabetes	0.0863	1.0902	0.1204	0.4734	0.8610	1.3804
systolic bloodpressure	0.0062	1.0063	0.0051	0.217	0.9963	1.0163
diastolic bloodpressure	−0.0087	0.9914	0.0098	0.375	0.9725	1.0106
CRP	−0.0424	0.9584	0.1578	0.7879	0.7035	1.3057
BMI	0.0578	1.0595	0.0403	0.1517	0.9790	1.1467
physical activity	0.2384	1.2693	0.1540	0.1216	0.9385	1.7165
waist circumference	−0.0200	0.9802	0.0150	0.1822	0.9519	1.0094
coronary artery calcification	0.2720	1.3126	0.0403	< 0.0001	1.2130	1.4204

I3)

	coef	HR	se	p	lower	upper
rs2487999	0.7801	2.1816	0.2876	0.0067	1.2416	3.8333
sex	0.2195	1.2454	0.2907	0.4502	0.7045	2.2017
age	0.0393	1.0401	0.0161	0.0146	1.0078	1.0734
total cholesterol	−0.0017	0.9983	0.0068	0.8006	0.9851	1.0116
HDL	−0.0084	0.9916	0.0102	0.408	0.9719	1.0116
LDL	0.0005	1.0005	0.0069	0.9469	0.9869	1.0142
triglycerides	0.0007	1.0007	0.0011	0.5064	0.9986	1.0029
diabetes	0.0317	1.0322	0.1740	0.8553	0.7340	1.4517
systolic bloodpressure	0.0085	1.0086	0.0075	0.2578	0.9938	1.0235
diastolic bloodpressure	−0.0027	0.9973	0.0137	0.8453	0.9709	1.0245
CRP	−0.0108	0.9893	0.1112	0.9228	0.7956	1.2301
BMI	−0.0383	0.9624	0.0460	0.4046	0.8794	1.0532
physical activity	0.1170	1.1241	0.2047	0.5675	0.7527	1.6789
waist circumference	0.0052	1.0053	0.0184	0.7756	0.9696	1.0422
coronary artery calcification	0.3168	1.3728	0.0514	< 0.0001	1.2411	1.5184

m1)

	coef	HR	se	p	lower	upper
rs2487999	0.4104	1.5074	0.2530	0.1048	0.9181	2.4750
sex	0.1438	1.1546	0.2808	0.6087	0.6659	2.0021
age	0.0436	1.0445	0.0167	0.0092	1.0109	1.0793
total cholesterol	0.0087	1.0087	0.0077	0.2595	0.9936	1.0240
HDL	−0.0075	0.9926	0.0096	0.4377	0.9740	1.0115
LDL	−0.0091	0.9910	0.0077	0.2384	0.9761	1.0060
triglycerides	0.0001	1.0001	0.0013	0.9381	0.9975	1.0027
diabetes	0.3155	1.3709	0.1878	0.093	0.9488	1.9808
smoking	0.1954	1.2158	0.1391	0.1601	0.9257	1.5968
systolic bloodpressure	0.0156	1.0158	0.0069	0.024	1.0021	1.0297
diastolic bloodpressure	−0.0172	0.9829	0.0154	0.2642	0.9536	1.0131
CRP	0.1843	1.2023	0.1517	0.2245	0.8931	1.6186
BMI	0.0019	1.0019	0.0487	0.9685	0.9107	1.1023
physical activity	0.5029	1.6534	0.2119	0.0176	1.0915	2.5046
coronary artery calcification	0.2996	1.3493	0.0510	< 0.0001	1.2209	1.4911

m2)

	coef	HR	se	p	lower	upper
rs2487999	−0.0462	0.9549	0.1231	0.7077	0.7502	1.2155
sex	−0.1107	0.8952	0.1395	0.4277	0.6811	1.1768
age	0.0629	1.0649	0.0092	< 0.0001	1.0459	1.0843
total cholesterol	−0.0047	0.9953	0.0042	0.2693	0.9871	1.0036
HDL	−0.0046	0.9954	0.0057	0.4221	0.9843	1.0067
LDL	0.0008	1.0008	0.0043	0.8558	0.9923	1.0093
triglycerides	0.0013	1.0013	0.0007	0.0882	0.9998	1.0027
diabetes	0.1131	1.1198	0.0844	0.1803	0.9490	1.3213
smoking	0.1473	1.1587	0.0808	0.0684	0.9889	1.3576
systolic bloodpressure	0.0049	1.0049	0.0038	0.2014	0.9974	1.0124
diastolic bloodpressure	−0.0003	0.9997	0.0072	0.9661	0.9857	1.0139
CRP	−0.0351	0.9655	0.0842	0.6767	0.8187	1.1387
BMI	−0.0004	0.9996	0.0139	0.9745	0.9726	1.0272
physical activity	0.0356	1.0362	0.1108	0.7481	0.8339	1.2876
coronary artery calcification	0.2662	1.3050	0.0288	< 0.0001	1.2333	1.3808

n1)

	coef	HR	se	p	lower	upper
rs2487999	0.3278	1.3879	0.2842	0.2487	0.7952	2.4223
sex	−0.0134	0.9867	0.3376	0.9683	0.5091	1.9123
age	0.0637	1.0658	0.0194	0.001	1.0261	1.1070
total cholesterol	0.0113	1.0113	0.0080	0.1607	0.9955	1.0274
HDL	−0.0163	0.9838	0.0103	0.1126	0.9642	1.0038
LDL	−0.0141	0.9860	0.0079	0.0758	0.9707	1.0015
triglycerides	−0.0007	0.9993	0.0015	0.6498	0.9964	1.0022
diabetes	0.2155	1.2405	0.2412	0.3716	0.7732	1.9902
smoking	0.2228	1.2496	0.1458	0.1264	0.9390	1.6628
systolic bloodpressure	0.0069	1.0069	0.0076	0.3663	0.9920	1.0220
diastolic bloodpressure	−0.0021	0.9979	0.0163	0.898	0.9665	1.0303
CRP	−0.0179	0.9823	0.1187	0.8802	0.7784	1.2395
physical activity	0.6000	1.8221	0.2362	0.0111	1.1469	2.8948
waist circumference	−0.0260	0.9743	0.0183	0.1554	0.9400	1.0099
coronary artery calcification	0.3821	1.4653	0.0604	< 0.0001	1.3018	1.6493

n2)

	coef	HR	se	p	lower	upper
rs2487999	−0.0188	0.9813	0.1192	0.8745	0.7768	1.2397
sex	−0.1630	0.8496	0.1404	0.2456	0.6452	1.1187
age	0.0584	1.0601	0.0089	< 0.0001	1.0418	1.0788
total cholesterol	−0.0053	0.9947	0.0041	0.1984	0.9867	1.0028
HDL	−0.0019	0.9981	0.0056	0.7359	0.9873	1.0091
LDL	0.0019	1.0019	0.0042	0.6568	0.9936	1.0102
triglycerides	0.0015	1.0015	0.0007	0.0338	1.0001	1.0029
diabetes	0.1299	1.1387	0.0830	0.1173	0.9679	1.3398
smoking	0.1654	1.1799	0.0788	0.0357	1.0111	1.3768
systolic bloodpressure	0.0061	1.0062	0.0038	0.1019	0.9988	1.0136
diastolic bloodpressure	−0.0057	0.9943	0.0072	0.4224	0.9804	1.0083
CRP	0.0043	1.0043	0.0850	0.9597	0.8501	1.1865
physical activity	0.0435	1.0445	0.1087	0.6889	0.8440	1.2926
waist circumference	−0.0042	0.9958	0.0057	0.4628	0.9848	1.0070
coronary artery calcification	0.2510	1.2854	0.0275	< 0.0001	1.2180	1.3565

S4F Table. Results of Cox regression models for rs4387287.
coef: coefficient, HR: Hazard Ratio, se: Standard error,
lower/upper: lower/upper boundarie of the 95% confidence interval
a) crude, b) adjusted, c1) young age, c2) older age, d1) male, d2) female,
e1) low hsCRP, e2) intermediate hsCRP, e3) high hsCRP, f1) low total cholesterol,
f2) high total cholesterol, g1) low LDL, g2) high LDL, h1) normal HDL, h2) high HDL,
i1) low triglycerides, i2) high triglycerides, j1) ideal blood pressure,
j2) normal/high normal blood pressure, j3) hypertension, k1) no diabetes, k2) diabetes,
l1) never smoker, l2) former smoker, l3) current smoker, m1) normal waist circumference,
m2) high waist circumference, n1) normal BMI, n2) high BMI

a)

	coef	HR	se	p	lower	upper
rs4387287	−0.1173	0.8893	0.0824	0.1546	0.7567	1.0452

b)

	coef	HR	se	p	lower	upper
rs4387287	−0.0812	0.9220	0.0866	0.3481	0.7781	1.0925
sex	−0.1142	0.8921	0.1487	0.4426	0.6666	1.1940
age	0.0595	1.0613	0.0081	< 0.0001	1.0445	1.0783
total cholesterol	−0.0021	0.9979	0.0038	0.5774	0.9905	1.0053
HDL	−0.0044	0.9956	0.0050	0.3786	0.9860	1.0054
LDL	−0.0011	0.9989	0.0038	0.7765	0.9914	1.0065
triglycerides	0.0011	1.0011	0.0006	0.089	0.9998	1.0024
diabetes	0.1525	1.1648	0.0799	0.0563	0.9959	1.3623
smoking	0.1800	1.1972	0.0695	0.0096	1.0447	1.3719
systolic bloodpressure	0.0070	1.0070	0.0034	0.0394	1.0003	1.0137
diastolic bloodpressure	−0.0049	0.9951	0.0066	0.4504	0.9824	1.0079
CRP	0.0033	1.0033	0.0701	0.9621	0.8745	1.1512
BMI	0.0046	1.0047	0.0222	0.8338	0.9620	1.0492
physical activity	0.1591	1.1725	0.0983	0.1056	0.9669	1.4218
waist circumference	−0.0083	0.9917	0.0087	0.3424	0.9749	1.0089
coronary artery calcification	0.2748	1.3163	0.0250	< 0.0001	1.2534	1.3824

c1)

	coef	HR	se	p	lower	upper
rs4387287	−0.1927	0.8247	0.1821	0.2899	0.5771	1.1785
sex	−0.1123	0.8938	0.2862	0.6947	0.5100	1.5663
total cholesterol	0.0017	1.0017	0.0067	0.8033	0.9886	1.0150
HDL	−0.0142	0.9859	0.0101	0.1612	0.9665	1.0057
LDL	−0.0014	0.9986	0.0069	0.8393	0.9853	1.0121
triglycerides	0.0003	1.0003	0.0013	0.8135	0.9977	1.0029
diabetes	−0.0100	0.9901	0.1969	0.9596	0.6731	1.4564
smoking	0.2952	1.3434	0.1342	0.0278	1.0327	1.7477
systolic bloodpressure	−0.0046	0.9954	0.0089	0.6036	0.9781	1.0130
diastolic bloodpressure	0.0196	1.0198	0.0163	0.2296	0.9877	1.0530
CRP	0.0964	1.1012	0.1619	0.5517	0.8018	1.5124
BMI	0.0110	1.0110	0.0468	0.8145	0.9225	1.1081
physical activity	0.2206	1.2468	0.2139	0.3025	0.8198	1.8963
waist circumference	−0.0146	0.9855	0.0184	0.4264	0.9507	1.0216
coronary artery calcification	0.2913	1.3381	0.0463	< 0.0001	1.2220	1.4653

c2)

	coef	HR	se	p	lower	upper
rs4387287	−0.0577	0.9439	0.0994	0.5617	0.7768	1.1470
sex	0.3407	1.4059	0.1746	0.051	0.9986	1.9794
total cholesterol	−0.0043	0.9957	0.0042	0.3043	0.9875	1.0039
HDL	−0.0004	0.9996	0.0047	0.9301	0.9905	1.0088
LDL	−0.0001	0.9999	0.0043	0.9868	0.9915	1.0085
triglycerides	0.0011	1.0011	0.0007	0.1013	0.9998	1.0025
diabetes	0.1818	1.1993	0.0886	0.0401	1.0082	1.4266
smoking	0.0797	1.0830	0.0820	0.3309	0.9222	1.2717
systolic bloodpressure	0.0137	1.0138	0.0036	0.0001	1.0068	1.0210
diastolic bloodpressure	−0.0181	0.9821	0.0070	0.0102	0.9686	0.9957
CRP	−0.0043	0.9957	0.0736	0.9534	0.8620	1.1502
BMI	−0.0105	0.9896	0.0257	0.6838	0.9409	1.0408
physical activity	0.1388	1.1489	0.1112	0.2119	0.9239	1.4288
waist circumference	−0.0011	0.9989	0.0100	0.9086	0.9795	1.0186
coronary artery calcification	0.2880	1.3338	0.0294	< 0.0001	1.2591	1.4129

d1)

	coef	HR	se	p	lower	upper
rs4387287	−0.0443	0.9566	0.1068	0.6779	0.7760	1.1793
age	0.0433	1.0443	0.0096	< 0.0001	1.0247	1.0641
total cholesterol	−0.0058	0.9942	0.0048	0.2265	0.9849	1.0036
HDL	0.0019	1.0019	0.0061	0.7592	0.9899	1.0140
LDL	0.0026	1.0026	0.0049	0.5938	0.9930	1.0123
triglycerides	0.0015	1.0015	0.0008	0.0548	1.0000	1.0030
diabetes	0.1697	1.1849	0.0911	0.0627	0.9911	1.4167
smoking	0.1080	1.1141	0.0892	0.2259	0.9354	1.3268
systolic bloodpressure	0.0048	1.0048	0.0043	0.2577	0.9965	1.0132
diastolic bloodpressure	−0.0026	0.9974	0.0080	0.742	0.9819	1.0131
CRP	0.0037	1.0037	0.0786	0.9625	0.8604	1.1709
BMI	0.0340	1.0346	0.0290	0.2411	0.9774	1.0952
physical activity	0.1645	1.1788	0.1209	0.1736	0.9301	1.4939
waist circumference	−0.0125	0.9876	0.0112	0.2659	0.9661	1.0095
coronary artery calcification	0.2968	1.3455	0.0333	< 0.0001	1.2606	1.4362

d2)

	coef	HR	se	p	lower	upper
rs4387287	−0.1308	0.8774	0.1493	0.3811	0.6548	1.1757
age	0.0990	1.1041	0.0152	< 0.0001	1.0717	1.1375
total cholesterol	0.0018	1.0018	0.0056	0.747	0.9909	1.0128
HDL	−0.0136	0.9865	0.0081	0.0927	0.9709	1.0023
LDL	−0.0056	0.9944	0.0057	0.3267	0.9834	1.0056
triglycerides	0.0012	1.0012	0.0013	0.3846	0.9986	1.0038
diabetes	0.1203	1.1279	0.1714	0.4826	0.8061	1.5782
smoking	0.3282	1.3885	0.1096	0.0028	1.1200	1.7213
systolic bloodpressure	0.0091	1.0092	0.0057	0.1102	0.9979	1.0205
diastolic bloodpressure	−0.0063	0.9937	0.0117	0.5906	0.9712	1.0168
CRP	0.0158	1.0160	0.1470	0.9142	0.7616	1.3553
BMI	−0.0287	0.9717	0.0350	0.4124	0.9073	1.0407
physical activity	0.1496	1.1613	0.1708	0.3812	0.8309	1.6232
waist circumference	−0.0069	0.9931	0.0141	0.6241	0.9660	1.0210
coronary artery calcification	0.2355	1.2656	0.0386	< 0.0001	1.1733	1.3651

e1)

	coef	HR	se	p	lower	upper
rs4387287	0.0434	1.0444	0.1086	0.6893	0.8442	1.2919
sex	−0.1714	0.8425	0.1815	0.345	0.5903	1.2024
age	0.0682	1.0706	0.0098	< 0.0001	1.0502	1.0914
total cholesterol	0.0000	1.0000	0.0043	0.9966	0.9915	1.0085
HDL	−0.0063	0.9937	0.0058	0.2747	0.9825	1.0050
LDL	−0.0037	0.9963	0.0044	0.3986	0.9876	1.0050
triglycerides	0.0011	1.0011	0.0007	0.1472	0.9996	1.0025
diabetes	0.1307	1.1397	0.1012	0.1964	0.9346	1.3897
smoking	0.1780	1.1948	0.0860	0.0386	1.0094	1.4142
systolic bloodpressure	0.0077	1.0078	0.0043	0.0694	0.9994	1.0162
diastolic bloodpressure	−0.0042	0.9958	0.0083	0.6123	0.9798	1.0121
BMI	0.0379	1.0387	0.0280	0.1754	0.9832	1.0973
physical activity	0.1800	1.1972	0.1183	0.1281	0.9495	1.5096
waist circumference	−0.0165	0.9836	0.0105	0.1146	0.9637	1.0040
coronary artery calcification	0.2716	1.3121	0.0301	< 0.0001	1.2369	1.3918

e2)

	coef	HR	se	p	lower	upper
rs4387287	−0.0191	0.9811	0.2664	0.943	0.5821	1.6537
sex	0.619	1.8571	0.4506	0.1695	0.7679	4.4911
age	0.0947	1.0994	0.0247	0.0001	1.0474	1.154
total cholesterol	−0.0056	0.9944	0.013	0.6649	0.9693	1.0201
HDL	−0.0101	0.99	0.0168	0.5488	0.9579	1.0231
LDL	0.0059	1.0059	0.0123	0.633	0.9819	1.0305
triglycerides	−0.0037	0.9963	0.0027	0.1603	0.9911	1.0015
diabetes	0.347	1.4149	0.1968	0.0778	0.9621	2.0808
smoking	0.1807	1.1981	0.2104	0.3904	0.7932	1.8096
systolic bloodpressure	−0.0027	0.9973	0.0096	0.782	0.9787	1.0163
diastolic bloodpressure	0.0092	1.0093	0.0174	0.5975	0.9753	1.0443
BMI	−0.125	0.8825	0.0729	0.0864	0.7649	1.0181
physical activity	−0.5674	0.567	0.2822	0.0444	0.3261	0.9859
waist circumference	0.0571	1.0588	0.0293	0.0516	0.9996	1.1214
coronary artery calcification	0.2872	1.3327	0.0751	0.0001	1.1503	1.544

e3)

	coef	HR	se	p	lower	upper
rs4387287	−0.4942	0.6100	0.1920	0.01	0.4188	0.8887
sex	−0.2273	0.7967	0.3544	0.5214	0.3977	1.5959
age	0.0109	1.0110	0.0186	0.5569	0.9748	1.0486
total cholesterol	−0.0048	0.9952	0.0066	0.4685	0.9825	1.0082
HDL	0.0010	1.0010	0.0034	0.7643	0.9944	1.0076
LDL	0.0032	1.0032	0.0069	0.6442	0.9897	1.0169
triglycerides	0.0022	1.0022	0.0013	0.0855	0.9997	1.0047
diabetes	0.1741	1.1902	0.1719	0.3112	0.8497	1.6670
smoking	0.1619	1.1757	0.1580	0.3058	0.8625	1.6026
systolic bloodpressure	0.0070	1.0070	0.0073	0.3401	0.9926	1.0216
diastolic bloodpressure	−0.0189	0.9813	0.0150	0.2064	0.9529	1.0105
BMI	−0.0408	0.9600	0.0490	0.4052	0.8721	1.0568
physical activity	0.4308	1.5386	0.2433	0.0765	0.9551	2.4784
waist circumference	−0.0125	0.9876	0.0206	0.5436	0.9486	1.0282
coronary artery calcification	0.2857	1.3307	0.0588	< 0.0001	1.1858	1.4932

f1)

	coef	HR	se	p	lower	upper
rs4387287	0.2955	1.3438	0.1719	0.0857	0.9594	1.8822
sex	0.4056	1.5002	0.2716	0.1353	0.8811	2.5545
age	0.0755	1.0784	0.0161	< 0.0001	1.0449	1.1130
HDL	−0.0082	0.9918	0.0086	0.34	0.9753	1.0087
LDL	−0.0042	0.9958	0.0048	0.3796	0.9866	1.0052
triglycerides	0.0005	1.0005	0.0013	0.7345	0.9978	1.0031
diabetes	0.2298	1.2584	0.1524	0.1314	0.9335	1.6962
smoking	0.0113	1.0114	0.1333	0.9323	0.7789	1.3132
systolic bloodpressure	0.0030	1.0030	0.0067	0.6588	0.9899	1.0162
diastolic bloodpressure	−0.0017	0.9983	0.0131	0.8937	0.9730	1.0241
CRP	−0.0545	0.9469	0.1228	0.6571	0.7444	1.2046
BMI	−0.1031	0.9020	0.0435	0.0178	0.8283	0.9823
physical activity	0.2733	1.3143	0.1917	0.154	0.9026	1.9137
waist circumference	0.0282	1.0286	0.0161	0.0795	0.9967	1.0615
coronary artery calcification	0.3003	1.3502	0.0478	< 0.0001	1.2295	1.4828

f2)

	coef	HR	se	p	lower	upper
rs4387287	−0.2459	0.7820	0.1023	0.0162	0.6399	0.9556
sex	−0.3152	0.7296	0.1774	0.0756	0.5154	1.0330
age	0.0570	1.0586	0.0094	< 0.0001	1.0392	1.0784
HDL	−0.0061	0.9939	0.0044	0.165	0.9854	1.0025
LDL	−0.0031	0.9969	0.0020	0.1113	0.9931	1.0007
triglycerides	0.0007	1.0007	0.0005	0.1264	0.9998	1.0017
diabetes	0.1216	1.1293	0.0957	0.2039	0.9362	1.3622
smoking	0.2642	1.3024	0.0828	0.0014	1.1074	1.5317
systolic bloodpressure	0.0076	1.0076	0.0040	0.0569	0.9998	1.0155
diastolic bloodpressure	−0.0061	0.9939	0.0077	0.4234	0.9791	1.0089
CRP	0.0158	1.0159	0.0855	0.8538	0.8592	1.2012
BMI	0.0497	1.0509	0.0257	0.053	0.9994	1.1052
physical activity	0.1267	1.1351	0.1167	0.2774	0.9031	1.4268
waist circumference	−0.0231	0.9772	0.0102	0.0235	0.9578	0.9969
coronary artery calcification	0.2659	1.3046	0.0297	< 0.0001	1.2308	1.3829

g1)

	coef	HR	se	p	lower	upper
rs4387287	0.0908	1.0951	0.1916	0.6355	0.7522	1.5942
sex	0.1682	1.1832	0.2945	0.5678	0.6644	2.1072
age	0.0828	1.0863	0.0177	< 0.0001	1.0493	1.1246
total cholesterol	−0.0020	0.9980	0.0046	0.6594	0.9889	1.0071
HDL	−0.0014	0.9986	0.0074	0.8505	0.9843	1.0131
triglycerides	0.0018	1.0018	0.0007	0.0142	1.0004	1.0032
diabetes	0.2143	1.2390	0.1676	0.201	0.8921	1.7207
smoking	0.1905	1.2099	0.1437	0.185	0.9128	1.6035
systolic bloodpressure	0.0012	1.0012	0.0073	0.8652	0.9871	1.0156
diastolic bloodpressure	−0.0058	0.9942	0.0138	0.6767	0.9676	1.0216
CRP	−0.2766	0.7583	0.2317	0.2325	0.4816	1.1942
BMI	−0.0748	0.9279	0.0460	0.104	0.8479	1.0155
physical activity	0.4962	1.6425	0.2105	0.0184	1.0873	2.4811
waist circumference	0.0213	1.0215	0.0173	0.2193	0.9874	1.0568
coronary artery calcification	0.2673	1.3065	0.0516	< 0.0001	1.1809	1.4454

g2)

	coef	HR	se	p	lower	upper
rs4387287	−0.1176	0.8891	0.0978	0.2294	0.7340	1.0770
sex	−0.1899	0.8270	0.1719	0.2692	0.5904	1.1584
age	0.0548	1.0564	0.0092	< 0.0001	1.0374	1.0757
total cholesterol	−0.0031	0.9969	0.0019	0.091	0.9932	1.0005
HDL	−0.0041	0.9959	0.0047	0.3799	0.9869	1.0051
triglycerides	0.0010	1.0010	0.0007	0.1702	0.9996	1.0023
diabetes	0.1290	1.1377	0.0922	0.1618	0.9496	1.3632
smoking	0.1988	1.2199	0.0801	0.0131	1.0426	1.4274
systolic bloodpressure	0.0077	1.0077	0.0039	0.0478	1.0001	1.0154
diastolic bloodpressure	−0.0042	0.9958	0.0075	0.5784	0.9813	1.0106
CRP	0.0904	1.0946	0.0846	0.2855	0.9273	1.2922
BMI	0.0265	1.0269	0.0251	0.2903	0.9776	1.0786
physical activity	0.0813	1.0847	0.1127	0.4709	0.8697	1.3528
waist circumference	−0.0171	0.9831	0.0099	0.0849	0.9642	1.0023
coronary artery calcification	0.2754	1.3171	0.0290	< 0.0001	1.2443	1.3941

h1)

	coef	HR	se	p	lower	upper
rs4387287	−0.0776	0.9254	0.1013	0.444	0.7587	1.1287
sex	−0.1270	0.8807	0.1727	0.4621	0.6279	1.2355
age	0.0700	1.0725	0.0098	< 0.0001	1.0520	1.0933
total cholesterol	−0.0018	0.9982	0.0035	0.6034	0.9915	1.0050
LDL	−0.0006	0.9994	0.0036	0.8668	0.9924	1.0064
triglycerides	−0.0002	0.9998	0.0008	0.8526	0.9982	1.0015
diabetes	0.1683	1.1833	0.0983	0.0868	0.9760	1.4348
smoking	0.2370	1.2674	0.0827	0.0041	1.0779	1.4903
systolic bloodpressure	0.0094	1.0094	0.0039	0.0161	1.0017	1.0171
diastolic bloodpressure	−0.0040	0.9961	0.0076	0.6035	0.9813	1.0110
CRP	−0.0766	0.9263	0.0993	0.4407	0.7624	1.1254
BMI	0.0200	1.0202	0.0260	0.4419	0.9695	1.0736
physical activity	0.1787	1.1957	0.1147	0.1191	0.9550	1.4971
waist circumference	−0.0070	0.9930	0.0104	0.4997	0.9729	1.0135
coronary artery calcification	0.2548	1.2902	0.0285	< 0.0001	1.2201	1.3642

h2)

	coef	HR	se	p	lower	upper
rs4387287	−0.1254	0.8821	0.1732	0.4689	0.6282	1.2386
sex	−0.2866	0.7508	0.2810	0.3078	0.4328	1.3023
age	0.0348	1.0354	0.0147	0.0177	1.0060	1.0656
total cholesterol	−0.0045	0.9955	0.0058	0.4398	0.9842	1.0070
LDL	0.0008	1.0008	0.0063	0.9023	0.9885	1.0132
triglycerides	0.0021	1.0021	0.0008	0.0113	1.0005	1.0037
diabetes	0.0672	1.0695	0.1411	0.6341	0.8111	1.4102
smoking	0.0358	1.0364	0.1342	0.7897	0.7968	1.3481
systolic bloodpressure	0.0006	1.0006	0.0069	0.9281	0.9872	1.0142
diastolic bloodpressure	−0.0067	0.9933	0.0133	0.6152	0.9678	1.0195
CRP	0.1733	1.1892	0.1157	0.1344	0.9478	1.4920
BMI	−0.0343	0.9663	0.0429	0.4236	0.8885	1.0510
physical activity	0.1174	1.1245	0.1920	0.5411	0.7718	1.6384
waist circumference	−0.0155	0.9847	0.0162	0.3401	0.9539	1.0164
coronary artery calcification	0.3321	1.3939	0.0525	< 0.0001	1.2577	1.5449

i1)

	coef	HR	se	p	lower	upper
rs4387287	−0.1172	0.8894	0.1157	0.311	0.7089	1.1158
sex	−0.0956	0.9089	0.2007	0.6339	0.6133	1.3468
age	0.0730	1.0757	0.0113	< 0.0001	1.0521	1.0999
total cholesterol	−0.0012	0.9988	0.0053	0.8219	0.9886	1.0092
HDL	−0.0025	0.9975	0.0064	0.6991	0.9851	1.0101
LDL	−0.0030	0.9970	0.0054	0.5815	0.9866	1.0076
diabetes	0.0376	1.0383	0.1258	0.7653	0.8114	1.3286
smoking	0.3057	1.3576	0.0944	0.0012	1.1283	1.6335
systolic bloodpressure	0.0045	1.0045	0.0046	0.3303	0.9954	1.0137
diastolic bloodpressure	0.0127	1.0128	0.0091	0.1621	0.9949	1.0310
CRP	−0.0489	0.9523	0.0889	0.5824	0.8001	1.1335
BMI	0.0288	1.0292	0.0312	0.357	0.9681	1.0941
physical activity	0.3502	1.4193	0.1336	0.0088	1.0923	1.8442
waist circumference	−0.0149	0.9853	0.0119	0.2127	0.9625	1.0085
coronary artery calcification	0.2773	1.3196	0.0330	< 0.0001	1.2370	1.4077

i2)

	coef	HR	se	p	lower	upper
rs4387287	−0.0557	0.9459	0.1310	0.6709	0.7317	1.2228
sex	−0.1258	0.8818	0.2254	0.5768	0.5669	1.3716
age	0.0448	1.0458	0.0116	0.0001	1.0222	1.0699
total cholesterol	0.0020	1.0020	0.0034	0.5551	0.9954	1.0086
HDL	−0.0176	0.9826	0.0070	0.0124	0.9691	0.9962
LDL	−0.0039	0.9961	0.0035	0.2674	0.9892	1.0030
diabetes	0.2470	1.2802	0.1062	0.0201	1.0395	1.5765
smoking	0.0561	1.0577	0.1049	0.5931	0.8611	1.2991
systolic bloodpressure	0.0098	1.0099	0.0049	0.0452	1.0002	1.0196
diastolic bloodpressure	−0.0247	0.9756	0.0097	0.0111	0.9571	0.9944
CRP	0.1149	1.1218	0.1349	0.3943	0.8611	1.4613
BMI	−0.0228	0.9774	0.0325	0.4827	0.9170	1.0418
physical activity	−0.1225	0.8847	0.1458	0.4009	0.6649	1.1773
waist circumference	0.0000	1.0000	0.0129	0.997	0.9751	1.0257
coronary artery calcification	0.2671	1.3062	0.0386	< 0.0001	1.2110	1.4089

j1)

	coef	HR	se	p	lower	upper
rs4387287	−0.2788	0.7567	0.2142	0.1931	0.4973	1.1515
sex	−0.1222	0.8850	0.3596	0.7341	0.4374	1.7907
age	0.0593	1.0611	0.0188	0.0016	1.0227	1.1009
total cholesterol	0.0138	1.0139	0.0108	0.2009	0.9927	1.0356
HDL	−0.0241	0.9762	0.0133	0.0692	0.9512	1.0019
LDL	−0.0141	0.9860	0.0103	0.1705	0.9663	1.0061
triglycerides	0.0025	1.0025	0.0025	0.3035	0.9977	1.0074
diabetes	−0.0479	0.9533	0.2239	0.8308	0.6146	1.4785
smoking	0.2259	1.2535	0.1760	0.1992	0.8878	1.7698
CRP	0.1262	1.1345	0.1495	0.3985	0.8464	1.5207
BMI	0.0075	1.0075	0.0602	0.9015	0.8953	1.1338
physical activity	0.2404	1.2717	0.2639	0.3624	0.7582	2.1331
waist circumference	−0.0171	0.9830	0.0237	0.4713	0.9383	1.0299
coronary artery calcification	0.3643	1.4395	0.0623	< 0.0001	1.2740	1.6265

j2)

	coef	HR	se	p	lower	upper
rs4387287	−0.1248	0.8826	0.1465	0.3941	0.6624	1.1762
sex	−0.5796	0.5601	0.2638	0.028	0.3340	0.9394
age	0.0713	1.0739	0.0125	< 0.0001	1.0479	1.1005
total cholesterol	0.0014	1.0014	0.0055	0.8008	0.9907	1.0121
HDL	−0.0094	0.9907	0.0083	0.2612	0.9746	1.0070
LDL	−0.0037	0.9963	0.0056	0.5077	0.9855	1.0072
triglycerides	0.0004	1.0004	0.0012	0.723	0.9981	1.0028
diabetes	0.2560	1.2918	0.1319	0.0522	0.9976	1.6727
smoking	0.2474	1.2807	0.1177	0.0356	1.0168	1.6131
CRP	0.0687	1.0711	0.1615	0.6708	0.7804	1.4700
BMI	0.0671	1.0694	0.0370	0.07	0.9945	1.1498
physical activity	0.2241	1.2511	0.1644	0.1728	0.9066	1.7266
waist circumference	−0.0350	0.9656	0.0147	0.017	0.9382	0.9938
coronary artery calcification	0.2116	1.2357	0.0395	< 0.0001	1.1437	1.3351

j3)

	coef	HR	se	p	lower	upper
rs4387287	−0.0103	0.9898	0.1266	0.9353	0.7723	1.2685
sex	0.1506	1.1626	0.2064	0.4656	0.7757	1.7423
age	0.0614	1.0633	0.0115	< 0.0001	1.0396	1.0875
total cholesterol	−0.0073	0.9927	0.0044	0.0973	0.9842	1.0013
HDL	0.0019	1.0019	0.0022	0.3662	0.9977	1.0062
LDL	0.0025	1.0025	0.0047	0.5913	0.9933	1.0118
triglycerides	0.0014	1.0014	0.0007	0.0345	1.0001	1.0028
diabetes	0.1399	1.1501	0.1131	0.2162	0.9215	1.4356
smoking	0.1603	1.1739	0.1027	0.1187	0.9598	1.4357
CRP	−0.0499	0.9513	0.0962	0.604	0.7879	1.1487
BMI	−0.0351	0.9655	0.0321	0.2736	0.9067	1.0281
physical activity	0.0601	1.0619	0.1410	0.6702	0.8055	1.3999
waist circumference	0.0077	1.0078	0.0124	0.5328	0.9836	1.0326
coronary artery calcification	0.2973	1.3462	0.0379	< 0.0001	1.2497	1.4501

k1)

	coef	HR	se	p	lower	upper
rs4387287	−0.1086	0.8971	0.1005	0.2795	0.7367	1.0923
sex	−0.1146	0.8917	0.1708	0.5021	0.6381	1.2462
age	0.0653	1.0674	0.0094	< 0.0001	1.0480	1.0872
total cholesterol	0.0001	1.0001	0.0042	0.9853	0.9918	1.0084
HDL	−0.0073	0.9928	0.0058	0.2119	0.9815	1.0042
LDL	−0.0023	0.9977	0.0043	0.5894	0.9893	1.0061
triglycerides	0.0002	1.0002	0.0009	0.8411	0.9984	1.0019
smoking	0.2909	1.3376	0.0794	0.0003	1.1448	1.5630
systolic bloodpressure	0.0039	1.0039	0.0042	0.3559	0.9957	1.0121
diastolic bloodpressure	0.0035	1.0035	0.0080	0.6602	0.9879	1.0195
CRP	−0.1074	0.8982	0.1139	0.3455	0.7185	1.1227
BMI	0.0246	1.0249	0.0267	0.3555	0.9728	1.0799
physical activity	0.2041	1.2264	0.1138	0.0729	0.9813	1.5328
waist circumference	−0.0091	0.9909	0.0102	0.3715	0.9713	1.0109
coronary artery calcification	0.2619	1.2994	0.0283	< 0.0001	1.2293	1.3735

k2)

	coef	HR	se	p	lower	upper
rs4387287	−0.0481	0.9530	0.1725	0.7802	0.6796	1.3364
sex	−0.3610	0.6970	0.3199	0.2591	0.3723	1.3048
age	0.0469	1.0480	0.0168	0.0052	1.0141	1.0831
total cholesterol	−0.0053	0.9947	0.0091	0.5591	0.9771	1.0126
HDL	0.0056	1.0056	0.0101	0.5814	0.9858	1.0258
LDL	0.0013	1.0013	0.0092	0.8876	0.9834	1.0196
triglycerides	0.0021	1.0021	0.0011	0.0637	0.9999	1.0044
smoking	−0.1065	0.8989	0.1553	0.4928	0.6630	1.2188
systolic bloodpressure	0.0108	1.0109	0.0061	0.0776	0.9988	1.0230
diastolic bloodpressure	−0.0183	0.9818	0.0117	0.1164	0.9596	1.0046
CRP	0.1124	1.1190	0.0829	0.1749	0.9512	1.3163
BMI	−0.0192	0.9809	0.0442	0.6637	0.8995	1.0698
physical activity	−0.0949	0.9095	0.2017	0.6379	0.6125	1.3503
waist circumference	−0.0148	0.9853	0.0177	0.4024	0.9516	1.0201
coronary artery calcification	0.3182	1.3747	0.0564	< 0.0001	1.2309	1.5352

I1)

	coef	HR	se	p	lower	upper
rs4387287	−0.1998	0.8189	0.1465	0.1724	0.6145	1.0911
sex	−0.1581	0.8538	0.2332	0.4978	0.5406	1.3485
age	0.0890	1.0931	0.0155	< 0.0001	1.0604	1.1268
total cholesterol	−0.0036	0.9964	0.0067	0.5921	0.9833	1.0096
HDL	−0.0102	0.9899	0.0090	0.2588	0.9726	1.0075
LDL	−0.0018	0.9982	0.0068	0.7911	0.9850	1.0116
triglycerides	0.0014	1.0014	0.0015	0.3594	0.9984	1.0045
diabetes	0.3690	1.4464	0.1402	0.0085	1.0987	1.9039
systolic bloodpressure	0.0087	1.0087	0.0060	0.1473	0.9969	1.0207
diastolic bloodpressure	−0.0059	0.9942	0.0119	0.6224	0.9712	1.0176
CRP	0.0175	1.0176	0.1105	0.8744	0.8194	1.2637
BMI	−0.0078	0.9922	0.0350	0.8237	0.9265	1.0626
physical activity	0.0171	1.0173	0.1703	0.9198	0.7287	1.4203
waist circumference	−0.0035	0.9965	0.0140	0.8012	0.9694	1.0243
coronary artery calcification	0.2553	1.2909	0.0409	< 0.0001	1.1914	1.3987

l2)

	coef	HR	se	p	lower	upper
rs4387287	−0.1377	0.8714	0.1326	0.299	0.6720	1.1299
sex	−0.4495	0.6379	0.2802	0.1086	0.3684	1.1047
age	0.0549	1.0565	0.0126	< 0.0001	1.0306	1.0829
total cholesterol	−0.0015	0.9985	0.0064	0.8143	0.9860	1.0112
HDL	0.0045	1.0045	0.0080	0.5759	0.9889	1.0203
LDL	−0.0009	0.9991	0.0065	0.8935	0.9865	1.0119
triglycerides	0.0014	1.0014	0.0010	0.1899	0.9993	1.0034
diabetes	0.0852	1.0889	0.1205	0.4794	0.8599	1.3790
systolic bloodpressure	0.0063	1.0063	0.0051	0.2144	0.9964	1.0163
diastolic bloodpressure	−0.0089	0.9911	0.0098	0.3615	0.9722	1.0103
CRP	−0.0354	0.9652	0.1573	0.8219	0.7091	1.3138
BMI	0.0584	1.0602	0.0401	0.1455	0.9800	1.1469
physical activity	0.2398	1.2710	0.1540	0.1193	0.9399	1.7187
waist circumference	−0.0199	0.9803	0.0149	0.1807	0.9521	1.0093
coronary artery calcification	0.2723	1.3129	0.0403	< 0.0001	1.2131	1.4209

l3)

	coef	HR	se	p	lower	upper
rs4387287	0.1789	1.1959	0.1954	0.3599	0.8154	1.7539
sex	0.1718	1.1875	0.2931	0.5576	0.6686	2.1091
age	0.0401	1.0409	0.0161	0.0129	1.0085	1.0744
total cholesterol	−0.0017	0.9983	0.0065	0.7928	0.9856	1.0112
HDL	−0.0072	0.9928	0.0099	0.4671	0.9738	1.0123
LDL	0.0004	1.0004	0.0068	0.9585	0.9872	1.0137
triglycerides	0.0007	1.0007	0.0011	0.5021	0.9986	1.0029
diabetes	0.0203	1.0205	0.1747	0.9076	0.7245	1.4373
systolic bloodpressure	0.0079	1.0079	0.0076	0.3001	0.9930	1.0231
diastolic bloodpressure	−0.0030	0.9970	0.0138	0.827	0.9703	1.0244
CRP	−0.0103	0.9898	0.1115	0.9266	0.7954	1.2316
BMI	−0.0332	0.9673	0.0457	0.4671	0.8844	1.0580
physical activity	0.1059	1.1117	0.2054	0.6064	0.7432	1.6628
waist circumference	0.0034	1.0034	0.0183	0.8518	0.9680	1.0402
coronary artery calcification	0.3051	1.3568	0.0516	< 0.0001	1.2262	1.5013

m1)

	coef	HR	se	p	lower	upper
rs4387287	0.0126	1.0126	0.1872	0.9465	0.7017	1.4614
sex	0.1310	1.1400	0.2815	0.6417	0.6565	1.9794
age	0.0434	1.0443	0.0167	0.0094	1.0107	1.0791
total cholesterol	0.0085	1.0086	0.0076	0.2645	0.9936	1.0238
HDL	−0.0073	0.9928	0.0097	0.4531	0.9741	1.0118
LDL	−0.0090	0.9910	0.0077	0.2439	0.9761	1.0062
triglycerides	0.0002	1.0002	0.0013	0.8941	0.9975	1.0028
diabetes	0.3102	1.3637	0.1887	0.1001	0.9422	1.9739
smoking	0.1928	1.2127	0.1404	0.1697	0.9209	1.5969
systolic bloodpressure	0.0152	1.0153	0.0070	0.0294	1.0015	1.0293
diastolic bloodpressure	−0.0152	0.9849	0.0154	0.3222	0.9557	1.0150
CRP	0.1780	1.1948	0.1540	0.2478	0.8835	1.6159
BMI	0.0005	1.0005	0.0489	0.9911	0.9091	1.1012
physical activity	0.4902	1.6327	0.2124	0.021	1.0766	2.4759
coronary artery calcification	0.2919	1.3390	0.0506	< 0.0001	1.2125	1.4787

m2)

	coef	HR	se	p	lower	upper
rs4387287	−0.0829	0.9205	0.0986	0.4007	0.7587	1.1167
sex	−0.1125	0.8936	0.1393	0.4195	0.6800	1.1742
age	0.0633	1.0653	0.0092	< 0.0001	1.0463	1.0847
total cholesterol	−0.0046	0.9954	0.0042	0.2785	0.9872	1.0037
HDL	−0.0047	0.9954	0.0057	0.4172	0.9842	1.0066
LDL	0.0007	1.0007	0.0043	0.8709	0.9922	1.0092
triglycerides	0.0013	1.0013	0.0007	0.0876	0.9998	1.0027
diabetes	0.1127	1.1193	0.0844	0.1816	0.9487	1.3206
smoking	0.1469	1.1582	0.0808	0.069	0.9887	1.3569
systolic bloodpressure	0.0048	1.0048	0.0038	0.2089	0.9973	1.0123
diastolic bloodpressure	−0.0003	0.9997	0.0072	0.9664	0.9856	1.0140
CRP	−0.0336	0.9669	0.0842	0.6895	0.8199	1.1403
BMI	−0.0003	0.9997	0.0139	0.982	0.9727	1.0274
physical activity	0.0353	1.0359	0.1109	0.7504	0.8336	1.2873
coronary artery calcification	0.2658	1.3045	0.0288	< 0.0001	1.2329	1.3803

n1)

	coef	HR	se	p	lower	upper
rs4387287	0.0861	1.0899	0.2143	0.6879	0.7162	1.6586
sex	−0.0038	0.9962	0.3377	0.991	0.5139	1.9310
age	0.0645	1.0666	0.0194	0.0009	1.0269	1.1079
total cholesterol	0.0107	1.0108	0.0081	0.1843	0.9949	1.0269
HDL	−0.0156	0.9845	0.0104	0.1327	0.9647	1.0047
LDL	−0.0137	0.9864	0.0080	0.089	0.9710	1.0021
triglycerides	−0.0006	0.9994	0.0015	0.6937	0.9965	1.0023
diabetes	0.2168	1.2421	0.2401	0.3667	0.7758	1.9887
smoking	0.2362	1.2664	0.1459	0.1055	0.9514	1.6857
systolic bloodpressure	0.0069	1.0070	0.0076	0.3638	0.9920	1.0222
diastolic bloodpressure	−0.0015	0.9985	0.0163	0.9263	0.9670	1.0310
CRP	−0.0193	0.9808	0.1199	0.8718	0.7754	1.2408
physical activity	0.5864	1.7975	0.2354	0.0127	1.1332	2.8512
waist circumference	−0.0250	0.9753	0.0183	0.1715	0.9409	1.0109
coronary artery calcification	0.3776	1.4587	0.0601	< 0.0001	1.2965	1.6412

n2)

	coef	HR	se	p	lower	upper
rs4387287	−0.1124	0.8937	0.0952	0.2377	0.7416	1.0770
sex	−0.1671	0.8461	0.1404	0.2339	0.6426	1.1141
age	0.0587	1.0604	0.0089	< 0.0001	1.0421	1.0791
total cholesterol	−0.0051	0.9949	0.0041	0.214	0.9869	1.0030
HDL	−0.0019	0.9981	0.0056	0.7354	0.9873	1.0090
LDL	0.0017	1.0017	0.0042	0.6905	0.9934	1.0100
triglycerides	0.0015	1.0015	0.0007	0.0341	1.0001	1.0028
diabetes	0.1265	1.1348	0.0829	0.127	0.9647	1.3350
smoking	0.1633	1.1774	0.0787	0.0381	1.0090	1.3738
systolic bloodpressure	0.0061	1.0061	0.0038	0.1077	0.9987	1.0135
diastolic bloodpressure	−0.0058	0.9942	0.0072	0.4187	0.9803	1.0083
CRP	0.0077	1.0077	0.0849	0.928	0.8532	1.1902
physical activity	0.0426	1.0435	0.1088	0.6953	0.8431	1.2916
waist circumference	−0.0041	0.9959	0.0057	0.4714	0.9849	1.0071
coronary artery calcification	0.2505	1.2846	0.0275	< 0.0001	1.2172	1.3558

S4G Table. Results of Cox regression models for rs8105767.

coef: coefficient, HR: Hazard Ratio, se: Standard error,
lower/upper: lower/upper boundarie of the 95% confidence interval
a) crude, b) adjusted, c1) young age, c2) older age, d1) male, d2) female,
e1) low hsCRP, e2) intermediate hsCRP, e3) high hsCRP, f1) low total cholesterol,
f2) high total cholesterol, g1) low LDL, g2) high LDL, h1) normal HDL, h2) high HDL,
i1) low triglycerides, i2) high triglycerides, j1) ideal blood pressure,
j2) normal/high normal blood pressure, j3) hypertension, k1) no diabetes, k2) diabetes,
l1) never smoker, l2) former smoker, l3) current smoker, m1) normal waist circumference,
m2) high waist circumference, n1) normal BMI, n2) high BMI

a)

	coef	HR	se	p	lower	upper
rs8105767	−0.0661	0.9361	0.0714	0.3546	0.8139	1.0766

b)

	coef	HR	se	p	lower	upper
rs8105767	0.0226	1.0229	0.0750	0.763	0.8831	1.1848
sex	−0.0926	0.9116	0.1488	0.534	0.6810	1.2203
age	0.0592	1.0610	0.0081	< 0.0001	1.0442	1.0780
total cholesterol	−0.0030	0.9970	0.0038	0.4355	0.9896	1.0045
HDL	−0.0047	0.9953	0.0050	0.3491	0.9857	1.0051
LDL	−0.0001	0.9999	0.0039	0.977	0.9923	1.0075
triglycerides	0.0011	1.0011	0.0007	0.0785	0.9999	1.0024
diabetes	0.1467	1.1580	0.0804	0.0681	0.9892	1.3557
smoking	0.1810	1.1984	0.0696	0.0093	1.0457	1.3734
systolic bloodpressure	0.0076	1.0076	0.0034	0.0243	1.0010	1.0143
diastolic bloodpressure	−0.0054	0.9947	0.0066	0.4133	0.9820	1.0075
CRP	0.0039	1.0039	0.0701	0.9552	0.8750	1.1518
BMI	0.0020	1.0020	0.0222	0.9269	0.9593	1.0467
physical activity	0.1588	1.1721	0.0986	0.1071	0.9662	1.4219
waist circumference	−0.0081	0.9920	0.0087	0.356	0.9751	1.0091
coronary artery calcification	0.2749	1.3164	0.0250	< 0.0001	1.2534	1.3825

c1)

	coef	HR	se	p	lower	upper
rs8105767	0.0699	1.0724	0.1637	0.6693	0.7781	1.4780
sex	−0.0856	0.9180	0.2872	0.7657	0.5229	1.6116
total cholesterol	0.0016	1.0016	0.0068	0.8123	0.9883	1.0151
HDL	−0.0149	0.9852	0.0103	0.1453	0.9656	1.0052
LDL	−0.0011	0.9989	0.0070	0.8702	0.9853	1.0126
triglycerides	0.0002	1.0002	0.0014	0.8883	0.9975	1.0029
diabetes	−0.0622	0.9397	0.2070	0.7637	0.6263	1.4099
smoking	0.3034	1.3545	0.1345	0.0241	1.0405	1.7632
systolic bloodpressure	−0.0033	0.9967	0.0089	0.7078	0.9793	1.0143
diastolic bloodpressure	0.0201	1.0203	0.0163	0.2173	0.9882	1.0534
CRP	0.1043	1.1099	0.1649	0.527	0.8035	1.5332
BMI	0.0054	1.0054	0.0472	0.9085	0.9167	1.1028
physical activity	0.1919	1.2116	0.2145	0.371	0.7957	1.8448
waist circumference	−0.0137	0.9864	0.0184	0.4557	0.9514	1.0226
coronary artery calcification	0.2879	1.3337	0.0464	< 0.0001	1.2177	1.4607

c2)

	coef	HR	se	p	lower	upper
rs8105767	0.0299	1.0304	0.0848	0.724	0.8726	1.2168
sex	0.3591	1.4321	0.1749	0.04	1.0165	2.0175
total cholesterol	−0.0053	0.9947	0.0043	0.2189	0.9863	1.0032
HDL	−0.0006	0.9994	0.0049	0.8956	0.9898	1.0090
LDL	0.0011	1.0011	0.0044	0.8067	0.9924	1.0098
triglycerides	0.0012	1.0012	0.0007	0.0829	0.9998	1.0026
diabetes	0.1836	1.2016	0.0886	0.0382	1.0101	1.4294
smoking	0.0805	1.0838	0.0821	0.3269	0.9228	1.2729
systolic bloodpressure	0.0141	1.0142	0.0036	0.0001	1.0071	1.0213
diastolic bloodpressure	−0.0184	0.9817	0.0071	0.0089	0.9682	0.9954
CRP	−0.0030	0.9970	0.0736	0.9677	0.8631	1.1517
BMI	−0.0120	0.9881	0.0257	0.6409	0.9395	1.0392
physical activity	0.1420	1.1526	0.1114	0.2025	0.9264	1.4340
waist circumference	−0.0013	0.9987	0.0100	0.8997	0.9794	1.0185
coronary artery calcification	0.2894	1.3356	0.0295	< 0.0001	1.2606	1.4151

d1)

	coef	HR	se	p	lower	upper
rs8105767	0.0849	1.0886	0.0930	0.3614	0.9072	1.3064
age	0.0426	1.0435	0.0097	< 0.0001	1.0240	1.0634
total cholesterol	−0.0071	0.9929	0.0048	0.1421	0.9836	1.0024
HDL	0.0013	1.0014	0.0062	0.8277	0.9893	1.0136
LDL	0.0041	1.0041	0.0049	0.4012	0.9945	1.0139
triglycerides	0.0015	1.0015	0.0008	0.0443	1.0000	1.0030
diabetes	0.1609	1.1746	0.0917	0.0793	0.9814	1.4058
smoking	0.1093	1.1154	0.0894	0.2215	0.9362	1.3290
systolic bloodpressure	0.0058	1.0058	0.0043	0.1737	0.9975	1.0142
diastolic bloodpressure	−0.0033	0.9967	0.0080	0.6832	0.9813	1.0125
CRP	0.0041	1.0042	0.0791	0.9582	0.8599	1.1726
BMI	0.0298	1.0303	0.0292	0.3072	0.9730	1.0909
physical activity	0.1680	1.1829	0.1213	0.1662	0.9326	1.5004
waist circumference	−0.0119	0.9882	0.0112	0.2894	0.9666	1.0102
coronary artery calcification	0.2977	1.3468	0.0334	< 0.0001	1.2615	1.4379

d2)

	coef	HR	se	p	lower	upper
rs8105767	−0.0822	0.9211	0.1263	0.5152	0.7191	1.1799
age	0.0983	1.1032	0.0152	< 0.0001	1.0709	1.1366
total cholesterol	0.0017	1.0017	0.0056	0.7649	0.9907	1.0127
HDL	−0.0137	0.9864	0.0082	0.0935	0.9708	1.0023
LDL	−0.0055	0.9945	0.0057	0.3358	0.9834	1.0057
triglycerides	0.0011	1.0011	0.0014	0.3997	0.9985	1.0038
diabetes	0.1154	1.1224	0.1713	0.5004	0.8022	1.5702
smoking	0.3369	1.4005	0.1097	0.0021	1.1296	1.7364
systolic bloodpressure	0.0096	1.0096	0.0057	0.0919	0.9984	1.0209
diastolic bloodpressure	−0.0071	0.9930	0.0117	0.5464	0.9704	1.0160
CRP	0.0072	1.0072	0.1478	0.9612	0.7539	1.3456
BMI	−0.0278	0.9726	0.0349	0.4254	0.9083	1.0414
physical activity	0.1452	1.1563	0.1710	0.3957	0.8271	1.6166
waist circumference	−0.0073	0.9927	0.0141	0.6036	0.9657	1.0205
coronary artery calcification	0.2348	1.2647	0.0386	< 0.0001	1.1725	1.3642

e1)

	coef	HR	se	p	lower	upper
rs8105767	−0.0022	0.9978	0.0917	0.9808	0.8336	1.1943
sex	−0.1499	0.8608	0.1817	0.4095	0.6029	1.2291
age	0.0678	1.0702	0.0099	< 0.0001	1.0497	1.0910
total cholesterol	−0.0009	0.9991	0.0044	0.8336	0.9905	1.0077
HDL	−0.0067	0.9933	0.0059	0.2504	0.9820	1.0048
LDL	−0.0027	0.9973	0.0045	0.5499	0.9885	1.0062
triglycerides	0.0011	1.0011	0.0007	0.1249	0.9997	1.0026
diabetes	0.1203	1.1278	0.1023	0.2399	0.9228	1.3784
smoking	0.1727	1.1885	0.0860	0.0446	1.0041	1.4066
systolic bloodpressure	0.0085	1.0086	0.0043	0.0454	1.0002	1.0171
diastolic bloodpressure	−0.0050	0.9950	0.0083	0.546	0.9789	1.0113
BMI	0.0348	1.0354	0.0282	0.2176	0.9797	1.0942
physical activity	0.1774	1.1941	0.1187	0.1351	0.9462	1.5068
waist circumference	−0.0163	0.9838	0.0105	0.1197	0.9637	1.0043
coronary artery calcification	0.2694	1.3092	0.0301	< 0.0001	1.2342	1.3889

e2)

	coef	HR	se	p	lower	upper
rs8105767	0.1905	1.2099	0.2273	0.4018	0.775	1.8889
sex	0.6314	1.8803	0.4447	0.1556	0.7865	4.495
age	0.096	1.1008	0.0247	0.0001	1.0488	1.1553
total cholesterol	−0.0051	0.9949	0.013	0.6954	0.9698	1.0207
HDL	−0.0115	0.9886	0.0169	0.4983	0.9563	1.022
LDL	0.0051	1.0051	0.0123	0.6773	0.9812	1.0297
triglycerides	−0.0036	0.9964	0.0026	0.1742	0.9913	1.0016
diabetes	0.318	1.3744	0.193	0.0995	0.9414	2.0064
smoking	0.1748	1.1911	0.2092	0.4032	0.7905	1.7946
systolic bloodpressure	−0.0026	0.9974	0.0094	0.7835	0.9793	1.0159
diastolic bloodpressure	0.0094	1.0094	0.0173	0.5878	0.9758	1.0441
BMI	−0.1318	0.8765	0.0724	0.0688	0.7605	1.0102
physical activity	−0.5578	0.5725	0.2818	0.0478	0.3296	0.9945
waist circumference	0.0593	1.0611	0.0287	0.0386	1.0031	1.1224
coronary artery calcification	0.295	1.3431	0.0748	0.0001	1.1599	1.5552

e3)

	coef	HR	se	p	lower	upper
rs8105767	0.0009	1.0009	0.1714	0.9957	0.7153	1.4006
sex	−0.3089	0.7343	0.3539	0.3827	0.3670	1.4691
age	0.0080	1.0080	0.0185	0.6656	0.9721	1.0452
total cholesterol	−0.0048	0.9952	0.0066	0.4599	0.9825	1.0080
HDL	0.0008	1.0008	0.0035	0.8182	0.9940	1.0076
LDL	0.0031	1.0032	0.0068	0.6445	0.9898	1.0167
triglycerides	0.0022	1.0022	0.0013	0.0785	0.9997	1.0047
diabetes	0.1662	1.1808	0.1737	0.3388	0.8400	1.6598
smoking	0.1400	1.1503	0.1596	0.3804	0.8413	1.5728
systolic bloodpressure	0.0066	1.0066	0.0073	0.3636	0.9924	1.0211
diastolic bloodpressure	−0.0189	0.9813	0.0149	0.204	0.9531	1.0103
BMI	−0.0303	0.9702	0.0485	0.5322	0.8822	1.0669
physical activity	0.4705	1.6009	0.2421	0.0519	0.9961	2.5729
waist circumference	−0.0174	0.9827	0.0203	0.3907	0.9444	1.0226
coronary artery calcification	0.2913	1.3381	0.0584	< 0.0001	1.1933	1.5005

f1)

	coef	HR	se	p	lower	upper
rs8105767	−0.0845	0.9190	0.1416	0.5507	0.6963	1.2129
sex	0.3973	1.4878	0.2711	0.1427	0.8746	2.5309
age	0.0734	1.0762	0.0162	< 0.0001	1.0425	1.1110
HDL	−0.0062	0.9938	0.0086	0.4674	0.9773	1.0106
LDL	−0.0045	0.9955	0.0048	0.3495	0.9863	1.0049
triglycerides	0.0007	1.0007	0.0013	0.5774	0.9981	1.0034
diabetes	0.2047	1.2271	0.1499	0.1721	0.9148	1.6462
smoking	−0.0034	0.9966	0.1324	0.9795	0.7689	1.2918
systolic bloodpressure	0.0031	1.0031	0.0067	0.6495	0.9899	1.0164
diastolic bloodpressure	−0.0030	0.9970	0.0131	0.8174	0.9716	1.0230
CRP	−0.0399	0.9609	0.1216	0.7429	0.7572	1.2194
BMI	−0.1062	0.8992	0.0432	0.0139	0.8262	0.9786
physical activity	0.2535	1.2885	0.1916	0.186	0.8850	1.8758
waist circumference	0.0306	1.0311	0.0159	0.0542	0.9994	1.0637
coronary artery calcification	0.2973	1.3463	0.0480	< 0.0001	1.2254	1.4790

f2)

	coef	HR	se	p	lower	upper
rs8105767	0.0564	1.0580	0.0897	0.5295	0.8874	1.2614
sex	−0.2774	0.7577	0.1773	0.1176	0.5353	1.0725
age	0.0553	1.0568	0.0095	< 0.0001	1.0374	1.0766
HDL	−0.0072	0.9929	0.0045	0.1074	0.9842	1.0016
LDL	−0.0027	0.9973	0.0019	0.1591	0.9935	1.0011
triglycerides	0.0007	1.0007	0.0005	0.1722	0.9997	1.0016
diabetes	0.1113	1.1177	0.0964	0.2483	0.9253	1.3500
smoking	0.2624	1.3000	0.0827	0.0015	1.1055	1.5288
systolic bloodpressure	0.0088	1.0088	0.0040	0.0272	1.0010	1.0167
diastolic bloodpressure	−0.0072	0.9928	0.0077	0.3458	0.9780	1.0078
CRP	0.0174	1.0175	0.0847	0.8374	0.8619	1.2012
BMI	0.0434	1.0444	0.0256	0.0905	0.9932	1.0982
physical activity	0.1198	1.1273	0.1170	0.3056	0.8964	1.4178
waist circumference	−0.0213	0.9789	0.0102	0.036	0.9596	0.9986
coronary artery calcification	0.2659	1.3046	0.0297	< 0.0001	1.2307	1.3829

g1)

	coef	HR	se	p	lower	upper
rs8105767	−0.2852	0.7519	0.1535	0.0632	0.5565	1.0158
sex	0.1463	1.1575	0.2988	0.6245	0.6445	2.0789
age	0.0819	1.0854	0.0179	< 0.0001	1.0480	1.1242
total cholesterol	−0.0021	0.9979	0.0048	0.6665	0.9885	1.0074
HDL	−0.0029	0.9971	0.0084	0.7317	0.9808	1.0137
triglycerides	0.0019	1.0019	0.0007	0.009	1.0005	1.0034
diabetes	0.2054	1.2280	0.1678	0.2211	0.8838	1.7064
smoking	0.1826	1.2004	0.1436	0.2034	0.9059	1.5906
systolic bloodpressure	0.0011	1.0011	0.0074	0.8849	0.9867	1.0157
diastolic bloodpressure	−0.0089	0.9911	0.0140	0.5216	0.9644	1.0186
CRP	−0.2574	0.7730	0.2301	0.2633	0.4924	1.2137
BMI	−0.0828	0.9206	0.0467	0.0764	0.8400	1.0088
physical activity	0.5093	1.6642	0.2117	0.0161	1.0990	2.5198
waist circumference	0.0221	1.0223	0.0173	0.2015	0.9883	1.0575
coronary artery calcification	0.2627	1.3004	0.0522	< 0.0001	1.1741	1.4404

g2)

	coef	HR	se	p	lower	upper
rs8105767	0.0978	1.1028	0.0874	0.263	0.9292	1.3088
sex	−0.1710	0.8429	0.1714	0.3185	0.6024	1.1793
age	0.0547	1.0562	0.0092	< 0.0001	1.0372	1.0755
total cholesterol	−0.0032	0.9968	0.0019	0.0894	0.9932	1.0005
HDL	−0.0045	0.9956	0.0047	0.3393	0.9865	1.0047
triglycerides	0.0009	1.0009	0.0007	0.2043	0.9995	1.0023
diabetes	0.1171	1.1242	0.0929	0.2075	0.9371	1.3486
smoking	0.2012	1.2228	0.0801	0.012	1.0452	1.4306
systolic bloodpressure	0.0082	1.0082	0.0039	0.035	1.0006	1.0159
diastolic bloodpressure	−0.0043	0.9958	0.0075	0.5705	0.9812	1.0105
CRP	0.0911	1.0953	0.0850	0.2843	0.9272	1.2940
BMI	0.0225	1.0228	0.0250	0.3675	0.9739	1.0742
physical activity	0.0768	1.0799	0.1129	0.4961	0.8655	1.3473
waist circumference	−0.0157	0.9844	0.0099	0.1113	0.9655	1.0036
coronary artery calcification	0.2769	1.3191	0.0290	< 0.0001	1.2462	1.3963

h1)

	coef	HR	se	p	lower	upper
rs8105767	0.0646	1.0668	0.0890	0.4677	0.8960	1.2700
sex	−0.1093	0.8965	0.1726	0.5265	0.6392	1.2572
age	0.0688	1.0712	0.0098	< 0.0001	1.0507	1.0920
total cholesterol	−0.0029	0.9971	0.0035	0.4081	0.9903	1.0040
LDL	0.0006	1.0006	0.0036	0.8688	0.9935	1.0077
triglycerides	0.0000	1.0000	0.0008	0.9681	0.9983	1.0016
diabetes	0.1700	1.1854	0.0982	0.0834	0.9778	1.4370
smoking	0.2360	1.2661	0.0827	0.0043	1.0767	1.4888
systolic bloodpressure	0.0100	1.0100	0.0039	0.0102	1.0024	1.0178
diastolic bloodpressure	−0.0049	0.9952	0.0076	0.5247	0.9804	1.0101
CRP	−0.0772	0.9257	0.0995	0.4374	0.7617	1.1249
BMI	0.0187	1.0189	0.0260	0.4716	0.9683	1.0721
physical activity	0.1813	1.1987	0.1149	0.1147	0.9570	1.5016
waist circumference	−0.0071	0.9930	0.0104	0.498	0.9729	1.0134
coronary artery calcification	0.2557	1.2913	0.0285	< 0.0001	1.2212	1.3654

h2)

	coef	HR	se	p	lower	upper
rs8105767	−0.0695	0.9329	0.1392	0.6176	0.7102	1.2254
sex	−0.2494	0.7792	0.2806	0.3741	0.4496	1.3507
age	0.0361	1.0367	0.0147	0.014	1.0073	1.0670
total cholesterol	−0.0053	0.9947	0.0059	0.3672	0.9833	1.0063
LDL	0.0016	1.0016	0.0064	0.796	0.9892	1.0142
triglycerides	0.0022	1.0022	0.0008	0.0099	1.0005	1.0038
diabetes	0.0423	1.0432	0.1445	0.7698	0.7859	1.3846
smoking	0.0429	1.0439	0.1343	0.7494	0.8022	1.3583
systolic bloodpressure	0.0014	1.0014	0.0069	0.8417	0.9879	1.0150
diastolic bloodpressure	−0.0061	0.9940	0.0132	0.6469	0.9685	1.0201
CRP	0.1678	1.1826	0.1163	0.1493	0.9415	1.4855
BMI	−0.0417	0.9591	0.0433	0.3353	0.8811	1.0441
physical activity	0.1186	1.1259	0.1927	0.5385	0.7717	1.6426
waist circumference	−0.0136	0.9864	0.0163	0.4027	0.9554	1.0185
coronary artery calcification	0.3307	1.3920	0.0528	< 0.0001	1.2551	1.5437

i1)

	coef	HR	se	p	lower	upper
rs8105767	0.0029	1.0029	0.1029	0.9778	0.8196	1.2271
sex	−0.0790	0.9240	0.2008	0.6938	0.6234	1.3695
age	0.0721	1.0747	0.0114	< 0.0001	1.0511	1.0989
total cholesterol	−0.0023	0.9977	0.0052	0.6543	0.9875	1.0079
HDL	−0.0025	0.9975	0.0064	0.693	0.9850	1.0101
LDL	−0.0017	0.9983	0.0054	0.7562	0.9879	1.0089
diabetes	0.0452	1.0463	0.1258	0.7191	0.8177	1.3387
smoking	0.3034	1.3544	0.0943	0.0013	1.1258	1.6295
systolic bloodpressure	0.0051	1.0052	0.0046	0.2662	0.9961	1.0143
diastolic bloodpressure	0.0117	1.0118	0.0091	0.1982	0.9939	1.0300
CRP	−0.0484	0.9527	0.0891	0.587	0.8000	1.1346
BMI	0.0271	1.0275	0.0312	0.3847	0.9665	1.0923
physical activity	0.3517	1.4215	0.1342	0.0088	1.0927	1.8491
waist circumference	−0.0153	0.9848	0.0119	0.1993	0.9621	1.0081
coronary artery calcification	0.2781	1.3206	0.0331	< 0.0001	1.2377	1.4092

i2)

	coef	HR	se	p	lower	upper
rs8105767	0.0662	1.0684	0.1095	0.5455	0.8621	1.3243
sex	−0.1037	0.9015	0.2256	0.6457	0.5793	1.4028
age	0.0453	1.0464	0.0117	0.0001	1.0227	1.0706
total cholesterol	0.0015	1.0015	0.0034	0.6687	0.9948	1.0081
HDL	−0.0179	0.9823	0.0071	0.0112	0.9688	0.9959
LDL	−0.0033	0.9968	0.0036	0.3626	0.9898	1.0038
diabetes	0.2327	1.2619	0.1071	0.0299	1.0230	1.5568
smoking	0.0578	1.0595	0.1050	0.5817	0.8625	1.3015
systolic bloodpressure	0.0105	1.0106	0.0049	0.032	1.0009	1.0204
diastolic bloodpressure	−0.0246	0.9757	0.0097	0.0114	0.9573	0.9945
CRP	0.1146	1.1214	0.1349	0.3957	0.8608	1.4609
BMI	−0.0264	0.9739	0.0327	0.4189	0.9135	1.0384
physical activity	−0.1306	0.8776	0.1463	0.3722	0.6588	1.1691
waist circumference	0.0010	1.0010	0.0129	0.9398	0.9760	1.0266
coronary artery calcification	0.2662	1.3050	0.0386	< 0.0001	1.2098	1.4077

j1)

	coef	HR	se	p	lower	upper
rs8105767	0.0612	1.0632	0.2020	0.7617	0.7156	1.5795
sex	−0.0763	0.9266	0.3639	0.834	0.4541	1.8905
age	0.0588	1.0606	0.0190	0.0019	1.0219	1.1008
total cholesterol	0.0135	1.0136	0.0109	0.2163	0.9921	1.0354
HDL	−0.0247	0.9756	0.0133	0.0633	0.9505	1.0014
LDL	−0.0135	0.9866	0.0104	0.195	0.9666	1.0070
triglycerides	0.0022	1.0023	0.0025	0.3658	0.9974	1.0072
diabetes	−0.0978	0.9068	0.2345	0.6766	0.5727	1.4359
smoking	0.2179	1.2435	0.1769	0.218	0.8792	1.7587
CRP	0.1247	1.1329	0.1480	0.3993	0.8476	1.5141
BMI	0.0041	1.0041	0.0601	0.9455	0.8926	1.1295
physical activity	0.2297	1.2582	0.2654	0.3868	0.7479	2.1169
waist circumference	−0.0145	0.9856	0.0237	0.5407	0.9408	1.0325
coronary artery calcification	0.3622	1.4364	0.0619	< 0.0001	1.2722	1.6219

j2)

	coef	HR	se	p	lower	upper
rs8105767	0.0923	1.0967	0.1309	0.4808	0.8485	1.4175
sex	−0.5425	0.5813	0.2642	0.04	0.3464	0.9756
age	0.0702	1.0727	0.0126	< 0.0001	1.0466	1.0994
total cholesterol	0.0000	1.0000	0.0056	0.9946	0.9891	1.0109
HDL	−0.0106	0.9895	0.0085	0.2125	0.9732	1.0061
LDL	−0.0021	0.9979	0.0057	0.7129	0.9869	1.0091
triglycerides	0.0005	1.0005	0.0012	0.653	0.9982	1.0029
diabetes	0.2602	1.2972	0.1317	0.0482	1.0021	1.6792
smoking	0.2346	1.2645	0.1175	0.0459	1.0043	1.5920
CRP	0.0686	1.0710	0.1650	0.6777	0.7751	1.4797
BMI	0.0617	1.0636	0.0370	0.0952	0.9893	1.1435
physical activity	0.2322	1.2614	0.1651	0.1596	0.9127	1.7432
waist circumference	−0.0349	0.9657	0.0146	0.0168	0.9384	0.9937
coronary artery calcification	0.2132	1.2377	0.0395	< 0.0001	1.1455	1.3372

j3)

	coef	HR	se	p	lower	upper
rs8105767	−0.0002	0.9998	0.1054	0.9983	0.8132	1.2292
sex	0.1532	1.1656	0.2064	0.4579	0.7777	1.7468
age	0.0612	1.0631	0.0115	< 0.0001	1.0394	1.0873
total cholesterol	−0.0074	0.9926	0.0044	0.0941	0.9841	1.0013
HDL	0.0019	1.0019	0.0022	0.3801	0.9976	1.0062
LDL	0.0027	1.0027	0.0047	0.568	0.9935	1.0120
triglycerides	0.0015	1.0015	0.0007	0.0329	1.0001	1.0028
diabetes	0.1363	1.1460	0.1130	0.2278	0.9183	1.4301
smoking	0.1610	1.1747	0.1019	0.114	0.9621	1.4344
CRP	−0.0507	0.9506	0.0962	0.5983	0.7872	1.1478
BMI	−0.0363	0.9643	0.0321	0.2582	0.9055	1.0270
physical activity	0.0632	1.0653	0.1409	0.6535	0.8082	1.4041
waist circumference	0.0081	1.0081	0.0124	0.5158	0.9839	1.0329
coronary artery calcification	0.2985	1.3478	0.0380	< 0.0001	1.2510	1.4521

k1)

	coef	HR	se	p	lower	upper
rs8105767	−0.0123	0.9878	0.0870	0.8875	0.8329	1.1715
sex	−0.0910	0.9131	0.1706	0.594	0.6535	1.2757
age	0.0642	1.0663	0.0094	< 0.0001	1.0469	1.0861
total cholesterol	−0.0006	0.9994	0.0043	0.8825	0.9911	1.0077
HDL	−0.0078	0.9922	0.0059	0.1815	0.9809	1.0037
LDL	−0.0015	0.9985	0.0043	0.73	0.9901	1.0070
triglycerides	0.0003	1.0003	0.0009	0.7771	0.9985	1.0020
smoking	0.2876	1.3332	0.0794	0.0003	1.1410	1.5576
systolic bloodpressure	0.0045	1.0045	0.0042	0.2782	0.9964	1.0128
diastolic bloodpressure	0.0028	1.0028	0.0080	0.7266	0.9872	1.0187
CRP	−0.1069	0.8986	0.1140	0.3487	0.7187	1.1237
BMI	0.0220	1.0222	0.0267	0.4105	0.9701	1.0771
physical activity	0.2073	1.2303	0.1140	0.069	0.9840	1.5384
waist circumference	−0.0090	0.9910	0.0102	0.376	0.9714	1.0110
coronary artery calcification	0.2632	1.3011	0.0283	< 0.0001	1.2309	1.3753

k2)

	coef	HR	se	p	lower	upper
rs8105767	0.1221	1.1299	0.1520	0.4217	0.8388	1.5221
sex	−0.3367	0.7141	0.3206	0.2936	0.3810	1.3386
age	0.0488	1.0500	0.0169	0.0038	1.0158	1.0854
total cholesterol	−0.0073	0.9927	0.0093	0.4293	0.9748	1.0109
HDL	0.0068	1.0069	0.0103	0.5078	0.9867	1.0274
LDL	0.0033	1.0033	0.0094	0.7244	0.9850	1.0219
triglycerides	0.0023	1.0023	0.0012	0.0523	1.0000	1.0046
smoking	−0.0954	0.9090	0.1558	0.5401	0.6698	1.2335
systolic bloodpressure	0.0118	1.0118	0.0061	0.0547	0.9998	1.0241
diastolic bloodpressure	−0.0181	0.9821	0.0117	0.1216	0.9598	1.0048
CRP	0.1097	1.1160	0.0822	0.182	0.9499	1.3111
BMI	−0.0228	0.9774	0.0443	0.6065	0.8962	1.0661
physical activity	−0.1143	0.8920	0.2025	0.5724	0.5997	1.3266
waist circumference	−0.0141	0.9860	0.0177	0.4243	0.9524	1.0207
coronary artery calcification	0.3156	1.3710	0.0567	< 0.0001	1.2268	1.5322

I1)

	coef	HR	se	p	lower	upper
rs8105767	−0.1074	0.8981	0.1231	0.3827	0.7056	1.1432
sex	−0.1771	0.8377	0.2348	0.4508	0.5288	1.3273
age	0.0870	1.0909	0.0154	< 0.0001	1.0583	1.1244
total cholesterol	−0.0033	0.9967	0.0068	0.6288	0.9836	1.0101
HDL	−0.0103	0.9897	0.0091	0.2532	0.9723	1.0074
LDL	−0.0020	0.9980	0.0068	0.77	0.9848	1.0114
triglycerides	0.0013	1.0013	0.0016	0.4152	0.9982	1.0043
diabetes	0.3751	1.4551	0.1407	0.0077	1.1044	1.9171
systolic bloodpressure	0.0091	1.0091	0.0060	0.1297	0.9973	1.0211
diastolic bloodpressure	−0.0074	0.9927	0.0119	0.5367	0.9697	1.0161
CRP	0.0090	1.0090	0.1096	0.9347	0.8139	1.2509
BMI	−0.0053	0.9947	0.0349	0.8791	0.9289	1.0652
physical activity	0.0269	1.0272	0.1705	0.8749	0.7354	1.4349
waist circumference	−0.0041	0.9959	0.0140	0.7699	0.9690	1.0236
coronary artery calcification	0.2514	1.2859	0.0408	< 0.0001	1.1871	1.3929

l2)

	coef	HR	se	p	lower	upper
rs8105767	−0.0215	0.9787	0.1185	0.8558	0.7758	1.2347
sex	−0.4196	0.6573	0.2806	0.1349	0.3793	1.1393
age	0.0552	1.0567	0.0127	< 0.0001	1.0307	1.0834
total cholesterol	−0.0038	0.9962	0.0066	0.5635	0.9835	1.0091
HDL	0.0042	1.0043	0.0081	0.6002	0.9884	1.0203
LDL	0.0017	1.0017	0.0066	0.7953	0.9888	1.0148
triglycerides	0.0015	1.0015	0.0011	0.1431	0.9995	1.0036
diabetes	0.0728	1.0755	0.1221	0.551	0.8466	1.3664
systolic bloodpressure	0.0074	1.0074	0.0051	0.1448	0.9975	1.0174
diastolic bloodpressure	−0.0093	0.9907	0.0098	0.3446	0.9718	1.0100
CRP	−0.0370	0.9637	0.1578	0.8148	0.7074	1.3129
BMI	0.0526	1.0540	0.0406	0.195	0.9734	1.1413
physical activity	0.2335	1.2630	0.1551	0.1323	0.9319	1.7118
waist circumference	−0.0204	0.9798	0.0150	0.173	0.9515	1.0090
coronary artery calcification	0.2704	1.3105	0.0404	< 0.0001	1.2107	1.4185

l3)

	coef	HR	se	p	lower	upper
rs8105767	0.4094	1.5059	0.1705	0.0164	1.0781	2.1036
sex	0.0705	1.0731	0.2901	0.8079	0.6077	1.8948
age	0.0400	1.0408	0.0160	0.0127	1.0086	1.0741
total cholesterol	0.0000	1.0000	0.0066	0.9985	0.9871	1.0131
HDL	−0.0085	0.9916	0.0098	0.3906	0.9726	1.0109
LDL	−0.0013	0.9987	0.0068	0.8481	0.9854	1.0122
triglycerides	0.0005	1.0005	0.0011	0.6778	0.9983	1.0026
diabetes	0.0164	1.0166	0.1740	0.9247	0.7229	1.4296
systolic bloodpressure	0.0080	1.0081	0.0076	0.2925	0.9931	1.0232
diastolic bloodpressure	−0.0033	0.9967	0.0139	0.8093	0.9700	1.0241
CRP	−0.0289	0.9715	0.1124	0.7972	0.7794	1.2110
BMI	−0.0258	0.9745	0.0451	0.5674	0.8921	1.0646
physical activity	0.0927	1.0971	0.2052	0.6514	0.7339	1.6403
waist circumference	0.0005	1.0005	0.0184	0.9772	0.9651	1.0373
coronary artery calcification	0.3013	1.3516	0.0505	< 0.0001	1.2241	1.4923

m1)

	coef	HR	se	p	lower	upper
rs8105767	−0.1312	0.8771	0.1658	0.4288	0.6337	1.2138
sex	0.1264	1.1348	0.2815	0.6533	0.6535	1.9704
age	0.0443	1.0452	0.0168	0.0085	1.0114	1.0803
total cholesterol	0.0088	1.0088	0.0076	0.249	0.9939	1.0240
HDL	−0.0073	0.9928	0.0097	0.4546	0.9741	1.0118
LDL	−0.0092	0.9909	0.0077	0.2351	0.9760	1.0060
triglycerides	0.0002	1.0002	0.0014	0.8928	0.9975	1.0028
diabetes	0.3098	1.3631	0.1889	0.101	0.9413	1.9738
smoking	0.1925	1.2123	0.1403	0.1701	0.9208	1.5960
systolic bloodpressure	0.0150	1.0151	0.0070	0.0315	1.0013	1.0290
diastolic bloodpressure	−0.0152	0.9849	0.0153	0.3184	0.9559	1.0148
CRP	0.1759	1.1923	0.1513	0.2451	0.8863	1.6039
BMI	−0.0001	0.9999	0.0493	0.9977	0.9078	1.1012
physical activity	0.4858	1.6255	0.2124	0.0222	1.0720	2.4649
coronary artery calcification	0.2915	1.3384	0.0505	< 0.0001	1.2124	1.4776

m2)

	coef	HR	se	p	lower	upper
rs8105767	0.0596	1.0614	0.0838	0.4774	0.9006	1.2509
sex	−0.0883	0.9155	0.1398	0.5277	0.6961	1.2040
age	0.0629	1.0649	0.0092	< 0.0001	1.0458	1.0843
total cholesterol	−0.0057	0.9944	0.0042	0.183	0.9861	1.0027
HDL	−0.0052	0.9948	0.0058	0.3693	0.9836	1.0062
LDL	0.0020	1.0020	0.0043	0.6507	0.9935	1.0105
triglycerides	0.0013	1.0013	0.0007	0.0756	0.9999	1.0028
diabetes	0.1046	1.1103	0.0850	0.2184	0.9399	1.3115
smoking	0.1465	1.1578	0.0810	0.0704	0.9879	1.3569
systolic bloodpressure	0.0057	1.0057	0.0038	0.1362	0.9982	1.0132
diastolic bloodpressure	−0.0009	0.9991	0.0072	0.9056	0.9851	1.0134
CRP	−0.0315	0.9690	0.0840	0.7075	0.8220	1.1423
BMI	−0.0031	0.9969	0.0141	0.8261	0.9698	1.0248
physical activity	0.0323	1.0329	0.1112	0.7712	0.8306	1.2843
coronary artery calcification	0.2660	1.3048	0.0289	< 0.0001	1.2330	1.3808

n1)

	coef	HR	se	p	lower	upper
rs8105767	−0.1196	0.8873	0.1814	0.5096	0.6218	1.2660
sex	0.0047	1.0047	0.3370	0.9888	0.5190	1.9450
age	0.0657	1.0679	0.0195	0.0007	1.0279	1.1095
total cholesterol	0.0106	1.0107	0.0081	0.1922	0.9947	1.0269
HDL	−0.0153	0.9849	0.0104	0.1441	0.9649	1.0052
LDL	−0.0135	0.9866	0.0081	0.0948	0.9710	1.0023
triglycerides	−0.0005	0.9995	0.0015	0.7207	0.9965	1.0024
diabetes	0.2184	1.2440	0.2391	0.3611	0.7786	1.9877
smoking	0.2482	1.2817	0.1473	0.0919	0.9604	1.7106
systolic bloodpressure	0.0070	1.0070	0.0076	0.3632	0.9920	1.0222
diastolic bloodpressure	−0.0015	0.9985	0.0163	0.9263	0.9671	1.0309
CRP	−0.0162	0.9839	0.1212	0.8937	0.7759	1.2477
physical activity	0.5728	1.7733	0.2348	0.0147	1.1192	2.8095
waist circumference	−0.0252	0.9751	0.0183	0.1694	0.9408	1.0108
coronary artery calcification	0.3759	1.4563	0.0599	< 0.0001	1.2950	1.6378

n2)

	coef	HR	se	p	lower	upper
rs8105767	0.0473	1.0485	0.0822	0.565	0.8924	1.2318
sex	−0.1476	0.8628	0.1405	0.2936	0.6551	1.1363
age	0.0584	1.0601	0.0089	< 0.0001	1.0417	1.0788
total cholesterol	−0.0062	0.9938	0.0041	0.1315	0.9858	1.0019
HDL	−0.0023	0.9977	0.0056	0.6754	0.9868	1.0087
LDL	0.0029	1.0030	0.0042	0.4842	0.9947	1.0113
triglycerides	0.0015	1.0015	0.0007	0.0279	1.0002	1.0029
diabetes	0.1216	1.1293	0.0833	0.1446	0.9591	1.3296
smoking	0.1667	1.1814	0.0788	0.0344	1.0123	1.3787
systolic bloodpressure	0.0069	1.0069	0.0038	0.0666	0.9995	1.0143
diastolic bloodpressure	−0.0063	0.9937	0.0072	0.3787	0.9799	1.0078
CRP	0.0060	1.0060	0.0850	0.944	0.8516	1.1883
physical activity	0.0409	1.0417	0.1091	0.708	0.8412	1.2900
waist circumference	−0.0049	0.9951	0.0057	0.3865	0.9840	1.0063
coronary artery calcification	0.2507	1.2850	0.0275	< 0.0001	1.2175	1.3562

S4H Table. Results of Cox regression models for rs412658.

coef: coefficient, HR: Hazard Ratio, se: Standard error,
lower/upper: lower/upper boundarie of the 95% confidence interval
a) crude, b) adjusted, c1) young age, c2) older age, d1) male, d2) female,
e1) low hsCRP, e2) intermediate hsCRP, e3) high hsCRP, f1) low total cholesterol,
f2) high total cholesterol, g1) low LDL, g2) high LDL, h1) normal HDL, h2) high HDL,
i1) low triglycerides, i2) high triglycerides, j1) ideal blood pressure,
j2) normal/high normal blood pressure, j3) hypertension, k1) no diabetes, k2) diabetes,
l1) never smoker, l2) former smoker, l3) current smoker, m1) normal waist circumference,
m2) high waist circumference, n1) normal BMI, n2) high BMI

a)

	coef	HR	se	p	lower	upper
rs412658	−0.0693	0.933	0.0671	0.3016	0.818	1.0642

b)

	coef	HR	se	p	lower	upper
rs412658	−0.0205	0.9798	0.0705	0.7717	0.8533	1.1249
sex	−0.1137	0.8925	0.1488	0.4449	0.6668	1.1948
age	0.0594	1.0612	0.0081	< 0.0001	1.0445	1.0782
total cholesterol	−0.0023	0.9977	0.0038	0.5495	0.9904	1.0052
HDL	−0.0043	0.9957	0.0050	0.385	0.9861	1.0054
LDL	−0.0010	0.9990	0.0039	0.8051	0.9915	1.0066
triglycerides	0.0011	1.0011	0.0006	0.0883	0.9998	1.0024
diabetes	0.1554	1.1681	0.0799	0.0517	0.9988	1.3661
smoking	0.1823	1.2000	0.0695	0.0087	1.0471	1.3753
systolic bloodpressure	0.0070	1.0071	0.0034	0.0377	1.0004	1.0137
diastolic bloodpressure	−0.0049	0.9951	0.0065	0.4505	0.9824	1.0079
CRP	0.0012	1.0012	0.0701	0.9861	0.8727	1.1486
BMI	0.0048	1.0048	0.0222	0.8292	0.9621	1.0494
physical activity	0.1603	1.1739	0.0983	0.103	0.9681	1.4233
waist circumference	−0.0084	0.9916	0.0087	0.3336	0.9748	1.0087
coronary artery calcification	0.2750	1.3165	0.0250	< 0.0001	1.2536	1.3826

c1)

	coef	HR	se	p	lower	upper
rs412658	0.0195	1.0197	0.1507	0.8972	0.7588	1.3702
sex	−0.1087	0.8970	0.2861	0.704	0.5120	1.5715
total cholesterol	0.0021	1.0021	0.0068	0.7531	0.9889	1.0155
HDL	−0.0144	0.9857	0.0102	0.1567	0.9661	1.0056
LDL	−0.0018	0.9982	0.0069	0.7979	0.9848	1.0118
triglycerides	0.0003	1.0003	0.0013	0.8427	0.9977	1.0029
diabetes	−0.0020	0.9980	0.1974	0.992	0.6778	1.4696
smoking	0.2983	1.3475	0.1343	0.0264	1.0356	1.7534
systolic bloodpressure	−0.0043	0.9957	0.0089	0.6268	0.9784	1.0133
diastolic bloodpressure	0.0192	1.0194	0.0163	0.2384	0.9874	1.0524
CRP	0.0993	1.1044	0.1652	0.5478	0.7990	1.5266
BMI	0.0093	1.0093	0.0467	0.8428	0.9210	1.1061
physical activity	0.2126	1.2369	0.2139	0.3203	0.8133	1.8810
waist circumference	−0.0141	0.9860	0.0183	0.4399	0.9512	1.0220
coronary artery calcification	0.2915	1.3384	0.0463	< 0.0001	1.2223	1.4655

c2)

	coef	HR	se	p	lower	upper
rs412658	−0.0283	0.9721	0.0804	0.7253	0.8303	1.1381
sex	0.3411	1.4065	0.1747	0.0508	0.9988	1.9806
total cholesterol	−0.0045	0.9955	0.0042	0.2873	0.9873	1.0038
HDL	−0.0004	0.9996	0.0046	0.9336	0.9905	1.0088
LDL	0.0001	1.0001	0.0043	0.9903	0.9916	1.0086
triglycerides	0.0011	1.0011	0.0007	0.1001	0.9998	1.0025
diabetes	0.1837	1.2016	0.0885	0.038	1.0102	1.4293
smoking	0.0812	1.0845	0.0820	0.3221	0.9236	1.2735
systolic bloodpressure	0.0138	1.0139	0.0036	0.0001	1.0068	1.0210
diastolic bloodpressure	−0.0181	0.9820	0.0070	0.0102	0.9686	0.9957
CRP	−0.0059	0.9942	0.0735	0.9365	0.8607	1.1483
BMI	−0.0104	0.9896	0.0257	0.6852	0.9410	1.0408
physical activity	0.1404	1.1508	0.1112	0.2066	0.9254	1.4310
waist circumference	−0.0013	0.9987	0.0100	0.8998	0.9794	1.0185
coronary artery calcification	0.2882	1.3340	0.0294	< 0.0001	1.2592	1.4131

d1)

	coef	HR	se	p	lower	upper
rs412658	0.0074	1.0074	0.0869	0.9325	0.8496	1.1944
age	0.0433	1.0442	0.0096	< 0.0001	1.0247	1.0641
total cholesterol	−0.0059	0.9941	0.0048	0.2208	0.9848	1.0035
HDL	0.0019	1.0019	0.0061	0.7579	0.9899	1.0140
LDL	0.0027	1.0027	0.0049	0.582	0.9931	1.0123
triglycerides	0.0015	1.0015	0.0008	0.0548	1.0000	1.0030
diabetes	0.1715	1.1871	0.0910	0.0595	0.9932	1.4189
smoking	0.1080	1.1141	0.0892	0.2258	0.9354	1.3268
systolic bloodpressure	0.0048	1.0048	0.0043	0.2578	0.9965	1.0132
diastolic bloodpressure	−0.0026	0.9974	0.0080	0.7485	0.9820	1.0131
CRP	0.0025	1.0025	0.0787	0.9749	0.8592	1.1697
BMI	0.0342	1.0348	0.0290	0.2384	0.9776	1.0954
physical activity	0.1652	1.1797	0.1208	0.1715	0.9309	1.4949
waist circumference	−0.0125	0.9876	0.0112	0.2646	0.9661	1.0095
coronary artery calcification	0.2972	1.3461	0.0333	< 0.0001	1.2611	1.4368

d2)

	coef	HR	se	p	lower	upper
rs412658	−0.0565	0.9451	0.1197	0.6371	0.7474	1.1951
age	0.0987	1.1037	0.0152	< 0.0001	1.0713	1.1371
total cholesterol	0.0016	1.0016	0.0056	0.7818	0.9906	1.0126
HDL	−0.0134	0.9867	0.0082	0.0999	0.9710	1.0026
LDL	−0.0055	0.9946	0.0057	0.3403	0.9835	1.0058
triglycerides	0.0012	1.0012	0.0013	0.3745	0.9986	1.0038
diabetes	0.1175	1.1247	0.1711	0.4923	0.8042	1.5729
smoking	0.3353	1.3984	0.1099	0.0023	1.1274	1.7346
systolic bloodpressure	0.0094	1.0095	0.0057	0.0976	0.9983	1.0207
diastolic bloodpressure	−0.0067	0.9933	0.0117	0.5679	0.9708	1.0164
CRP	0.0065	1.0065	0.1489	0.9652	0.7518	1.3475
BMI	−0.0288	0.9716	0.0349	0.4084	0.9074	1.0403
physical activity	0.1537	1.1661	0.1709	0.3684	0.8342	1.6301
waist circumference	−0.0070	0.9931	0.0141	0.6209	0.9660	1.0208
coronary artery calcification	0.2347	1.2646	0.0387	< 0.0001	1.1721	1.3643

e1)

	coef	HR	se	p	lower	upper
rs412658	−0.0228	0.9774	0.0862	0.7913	0.8255	1.1574
sex	−0.1743	0.8401	0.1814	0.3367	0.5887	1.1988
age	0.0683	1.0707	0.0098	< 0.0001	1.0503	1.0915
total cholesterol	0.0001	1.0001	0.0043	0.9842	0.9917	1.0086
HDL	−0.0063	0.9937	0.0058	0.2733	0.9825	1.0050
LDL	−0.0039	0.9961	0.0044	0.3843	0.9875	1.0048
triglycerides	0.0010	1.0010	0.0007	0.1503	0.9996	1.0025
diabetes	0.1312	1.1402	0.1013	0.195	0.9350	1.3905
smoking	0.1761	1.1925	0.0859	0.0404	1.0077	1.4112
systolic bloodpressure	0.0077	1.0077	0.0043	0.0719	0.9993	1.0162
diastolic bloodpressure	−0.0042	0.9958	0.0083	0.6116	0.9797	1.0121
BMI	0.0380	1.0387	0.0280	0.1757	0.9832	1.0974
physical activity	0.1795	1.1966	0.1183	0.1292	0.9490	1.5088
waist circumference	−0.0165	0.9836	0.0105	0.115	0.9636	1.0040
coronary artery calcification	0.2710	1.3113	0.0301	< 0.0001	1.2362	1.3909

e2)

	coef	HR	se	p	lower	upper
rs412658	0.2217	1.2482	0.2111	0.2935	0.8253	1.8878
sex	0.602	1.8257	0.443	0.1742	0.7662	4.3505
age	0.0964	1.1012	0.0248	0.0001	1.0489	1.1561
total cholesterol	−0.0051	0.9949	0.013	0.6971	0.9698	1.0207
HDL	−0.0101	0.99	0.0169	0.5502	0.9578	1.0233
LDL	0.0049	1.0049	0.0124	0.695	0.9808	1.0295
triglycerides	−0.0037	0.9963	0.0026	0.1609	0.9912	1.0015
diabetes	0.3506	1.4199	0.1923	0.0683	0.974	2.0699
smoking	0.1843	1.2024	0.209	0.3779	0.7982	1.8113
systolic bloodpressure	−0.0029	0.9971	0.0094	0.7527	0.9789	1.0155
diastolic bloodpressure	0.0096	1.0097	0.0173	0.5774	0.9761	1.0444
BMI	−0.1245	0.883	0.0716	0.082	0.7674	1.0159
physical activity	−0.5698	0.5656	0.2817	0.0431	0.3256	0.9825
waist circumference	0.0576	1.0593	0.0284	0.0425	1.0019	1.1199
coronary artery calcification	0.2949	1.343	0.075	0.0001	1.1594	1.5558

e3)

	coef	HR	se	p	lower	upper
rs412658	−0.1428	0.8670	0.1650	0.387	0.6274	1.1980
sex	−0.3325	0.7171	0.3564	0.3509	0.3566	1.4421
age	0.0079	1.0079	0.0185	0.6686	0.9721	1.0451
total cholesterol	−0.0053	0.9947	0.0065	0.4201	0.9821	1.0076
HDL	0.0011	1.0011	0.0032	0.7435	0.9947	1.0074
LDL	0.0035	1.0035	0.0068	0.6072	0.9902	1.0170
triglycerides	0.0024	1.0024	0.0013	0.0587	0.9999	1.0049
diabetes	0.1670	1.1817	0.1735	0.3359	0.8410	1.6604
smoking	0.1428	1.1534	0.1598	0.3718	0.8432	1.5778
systolic bloodpressure	0.0063	1.0064	0.0073	0.3878	0.9920	1.0210
diastolic bloodpressure	−0.0183	0.9819	0.0150	0.2216	0.9535	1.0111
BMI	−0.0270	0.9734	0.0489	0.5814	0.8844	1.0713
physical activity	0.4782	1.6132	0.2424	0.0485	1.0031	2.5944
waist circumference	−0.0188	0.9814	0.0204	0.3565	0.9430	1.0214
coronary artery calcification	0.2903	1.3369	0.0584	< 0.0001	1.1923	1.4989

f1)

	coef	HR	se	p	lower	upper
rs412658	−0.0057	0.9943	0.1349	0.9664	0.7634	1.2952
sex	0.4089	1.5052	0.2703	0.1304	0.8861	2.5568
age	0.0731	1.0758	0.0162	< 0.0001	1.0421	1.1105
HDL	−0.0064	0.9936	0.0085	0.4529	0.9771	1.0104
LDL	−0.0047	0.9953	0.0048	0.3307	0.9860	1.0047
triglycerides	0.0007	1.0007	0.0013	0.6162	0.9980	1.0033
diabetes	0.2030	1.2251	0.1502	0.1765	0.9127	1.6445
smoking	−0.0053	0.9947	0.1323	0.968	0.7674	1.2893
systolic bloodpressure	0.0032	1.0032	0.0067	0.6345	0.9900	1.0166
diastolic bloodpressure	−0.0025	0.9975	0.0132	0.849	0.9720	1.0236
CRP	−0.0398	0.9609	0.1226	0.7453	0.7557	1.2220
BMI	−0.1059	0.8995	0.0431	0.0141	0.8266	0.9789
physical activity	0.2599	1.2968	0.1917	0.1751	0.8907	1.8882
waist circumference	0.0304	1.0308	0.0159	0.0566	0.9991	1.0635
coronary artery calcification	0.2988	1.3483	0.0481	< 0.0001	1.2271	1.4814

f2)

	coef	HR	se	p	lower	upper
rs412658	−0.0409	0.9599	0.0842	0.6274	0.8138	1.1323
sex	−0.3098	0.7336	0.1774	0.0808	0.5181	1.0387
age	0.0555	1.0571	0.0094	< 0.0001	1.0377	1.0768
HDL	−0.0058	0.9942	0.0044	0.1855	0.9857	1.0028
LDL	−0.0029	0.9971	0.0019	0.1301	0.9933	1.0009
triglycerides	0.0007	1.0007	0.0005	0.1354	0.9998	1.0017
diabetes	0.1252	1.1333	0.0955	0.1899	0.9399	1.3665
smoking	0.2645	1.3027	0.0827	0.0014	1.1078	1.5320
systolic bloodpressure	0.0079	1.0079	0.0040	0.0469	1.0001	1.0158
diastolic bloodpressure	−0.0064	0.9936	0.0076	0.4012	0.9788	1.0086
CRP	0.0137	1.0138	0.0847	0.8718	0.8586	1.1969
BMI	0.0481	1.0493	0.0256	0.0601	0.9980	1.1032
physical activity	0.1190	1.1264	0.1166	0.3073	0.8963	1.4157
waist circumference	−0.0223	0.9779	0.0102	0.0282	0.9586	0.9976
coronary artery calcification	0.2658	1.3045	0.0297	< 0.0001	1.2307	1.3828

g1)

	coef	HR	se	p	lower	upper
rs412658	−0.0863	0.9173	0.1463	0.5552	0.6885	1.2220
sex	0.1646	1.1789	0.2946	0.5763	0.6618	2.1001
age	0.0822	1.0857	0.0176	< 0.0001	1.0488	1.1238
total cholesterol	−0.0019	0.9981	0.0047	0.6864	0.9890	1.0073
HDL	−0.0014	0.9986	0.0074	0.8538	0.9842	1.0132
triglycerides	0.0018	1.0018	0.0007	0.0125	1.0004	1.0032
diabetes	0.2111	1.2350	0.1669	0.2058	0.8905	1.7128
smoking	0.1870	1.2056	0.1435	0.1926	0.9100	1.5972
systolic bloodpressure	0.0010	1.0010	0.0073	0.8907	0.9867	1.0155
diastolic bloodpressure	−0.0062	0.9938	0.0139	0.6547	0.9670	1.0213
CRP	−0.2680	0.7649	0.2301	0.2442	0.4872	1.2009
BMI	−0.0761	0.9268	0.0459	0.0977	0.8470	1.0141
physical activity	0.4940	1.6388	0.2103	0.0188	1.0852	2.4750
waist circumference	0.0219	1.0222	0.0172	0.2025	0.9883	1.0573
coronary artery calcification	0.2651	1.3036	0.0519	< 0.0001	1.1776	1.4431

g2)

	coef	HR	se	p	lower	upper
rs412658	−0.0156	0.9845	0.0812	0.8477	0.8397	1.1543
sex	−0.1879	0.8287	0.1719	0.2742	0.5917	1.1606
age	0.0543	1.0558	0.0092	< 0.0001	1.0369	1.0751
total cholesterol	−0.0032	0.9968	0.0019	0.0817	0.9931	1.0004
HDL	−0.0040	0.9960	0.0047	0.3948	0.9870	1.0052
triglycerides	0.0010	1.0010	0.0007	0.1711	0.9996	1.0023
diabetes	0.1334	1.1427	0.0921	0.1477	0.9539	1.3689
smoking	0.2006	1.2221	0.0802	0.0124	1.0443	1.4301
systolic bloodpressure	0.0078	1.0078	0.0039	0.0441	1.0002	1.0155
diastolic bloodpressure	−0.0042	0.9958	0.0075	0.5773	0.9813	1.0106
CRP	0.0889	1.0929	0.0847	0.2938	0.9258	1.2902
BMI	0.0258	1.0261	0.0250	0.3027	0.9770	1.0777
physical activity	0.0803	1.0836	0.1127	0.476	0.8689	1.3514
waist circumference	−0.0169	0.9833	0.0099	0.0883	0.9644	1.0025
coronary artery calcification	0.2764	1.3184	0.0290	< 0.0001	1.2456	1.3955

h1)

	coef	HR	se	p	lower	upper
rs412658	0.0233	1.0236	0.0834	0.7795	0.8693	1.2053
sex	−0.1273	0.8804	0.1727	0.4609	0.6276	1.2351
age	0.0699	1.0724	0.0098	< 0.0001	1.0519	1.0932
total cholesterol	−0.0019	0.9981	0.0035	0.5878	0.9914	1.0049
LDL	−0.0005	0.9995	0.0036	0.8854	0.9925	1.0065
triglycerides	−0.0001	0.9999	0.0008	0.8683	0.9982	1.0015
diabetes	0.1706	1.1861	0.0981	0.0821	0.9785	1.4376
smoking	0.2369	1.2673	0.0827	0.0042	1.0776	1.4903
systolic bloodpressure	0.0095	1.0095	0.0039	0.0149	1.0018	1.0172
diastolic bloodpressure	−0.0040	0.9960	0.0076	0.595	0.9812	1.0109
CRP	−0.0794	0.9236	0.0995	0.4248	0.7600	1.1226
BMI	0.0194	1.0196	0.0260	0.4552	0.9689	1.0730
physical activity	0.1801	1.1974	0.1147	0.1163	0.9563	1.4991
waist circumference	−0.0069	0.9931	0.0104	0.5051	0.9730	1.0136
coronary artery calcification	0.2554	1.2910	0.0285	< 0.0001	1.2209	1.3651

h2)

	coef	HR	se	p	lower	upper
rs412658	−0.1660	0.8470	0.1304	0.2029	0.6560	1.0937
sex	−0.2650	0.7672	0.2810	0.3457	0.4423	1.3308
age	0.0338	1.0343	0.0146	0.0212	1.0051	1.0644
total cholesterol	−0.0049	0.9951	0.0059	0.4007	0.9837	1.0066
LDL	0.0008	1.0008	0.0063	0.8965	0.9885	1.0133
triglycerides	0.0021	1.0021	0.0008	0.0098	1.0005	1.0037
diabetes	0.0744	1.0773	0.1419	0.6001	0.8157	1.4227
smoking	0.0397	1.0405	0.1341	0.7671	0.8000	1.3534
systolic bloodpressure	0.0006	1.0006	0.0069	0.9322	0.9871	1.0143
diastolic bloodpressure	−0.0063	0.9938	0.0133	0.637	0.9682	1.0200
CRP	0.1694	1.1845	0.1158	0.1437	0.9440	1.4864
BMI	−0.0348	0.9658	0.0426	0.4144	0.8884	1.0500
physical activity	0.1236	1.1315	0.1917	0.5193	0.7771	1.6476
waist circumference	−0.0155	0.9846	0.0162	0.3359	0.9539	1.0162
coronary artery calcification	0.3321	1.3939	0.0525	< 0.0001	1.2575	1.5450

i1)

	coef	HR	se	p	lower	upper
rs412658	−0.0689	0.9334	0.0953	0.4697	0.7743	1.1251
sex	−0.1028	0.9023	0.2008	0.6087	0.6087	1.3375
age	0.0735	1.0762	0.0114	< 0.0001	1.0525	1.1004
total cholesterol	−0.0016	0.9984	0.0052	0.761	0.9882	1.0087
HDL	−0.0023	0.9977	0.0063	0.7222	0.9854	1.0102
LDL	−0.0025	0.9975	0.0054	0.6357	0.9870	1.0080
diabetes	0.0345	1.0351	0.1262	0.7842	0.8084	1.3255
smoking	0.3080	1.3606	0.0943	0.0011	1.1310	1.6369
systolic bloodpressure	0.0045	1.0045	0.0046	0.3281	0.9955	1.0137
diastolic bloodpressure	0.0125	1.0125	0.0091	0.1694	0.9947	1.0307
CRP	−0.0528	0.9486	0.0890	0.553	0.7968	1.1293
BMI	0.0297	1.0301	0.0312	0.3419	0.9690	1.0951
physical activity	0.3455	1.4126	0.1337	0.0098	1.0870	1.8357
waist circumference	−0.0155	0.9846	0.0119	0.1936	0.9619	1.0079
coronary artery calcification	0.2768	1.3189	0.0330	< 0.0001	1.2362	1.4072

i2)

	coef	HR	se	p	lower	upper
rs412658	0.0357	1.0364	0.1046	0.7327	0.8443	1.2722
sex	−0.1233	0.8840	0.2253	0.5842	0.5684	1.3748
age	0.0444	1.0454	0.0116	0.0001	1.0219	1.0695
total cholesterol	0.0019	1.0019	0.0034	0.5727	0.9953	1.0085
HDL	−0.0175	0.9826	0.0070	0.0127	0.9692	0.9963
LDL	−0.0038	0.9962	0.0035	0.2811	0.9893	1.0031
diabetes	0.2469	1.2800	0.1065	0.0204	1.0389	1.5771
smoking	0.0561	1.0577	0.1050	0.5929	0.8611	1.2993
systolic bloodpressure	0.0100	1.0100	0.0049	0.0421	1.0004	1.0197
diastolic bloodpressure	−0.0248	0.9755	0.0097	0.0109	0.9571	0.9943
CRP	0.1129	1.1195	0.1351	0.4033	0.8591	1.4588
BMI	−0.0227	0.9776	0.0325	0.4851	0.9173	1.0418
physical activity	−0.1223	0.8848	0.1458	0.4014	0.6649	1.1775
waist circumference	0.0001	1.0001	0.0129	0.994	0.9752	1.0257
coronary artery calcification	0.2675	1.3066	0.0386	< 0.0001	1.2114	1.4094

j1)

	coef	HR	se	p	lower	upper
rs412658	−0.1136	0.8926	0.1733	0.512	0.6355	1.2536
sex	−0.1410	0.8685	0.3628	0.6975	0.4265	1.7683
age	0.0575	1.0592	0.0187	0.0021	1.0210	1.0988
total cholesterol	0.0140	1.0141	0.0108	0.1926	0.9930	1.0357
HDL	−0.0236	0.9766	0.0132	0.0724	0.9518	1.0022
LDL	−0.0143	0.9858	0.0103	0.1655	0.9662	1.0059
triglycerides	0.0023	1.0023	0.0025	0.3417	0.9975	1.0072
diabetes	−0.0473	0.9538	0.2259	0.8343	0.6127	1.4850
smoking	0.2341	1.2638	0.1760	0.1836	0.8950	1.7845
CRP	0.1195	1.1270	0.1475	0.4175	0.8441	1.5046
BMI	0.0105	1.0105	0.0597	0.8611	0.8989	1.1360
physical activity	0.2643	1.3025	0.2643	0.3173	0.7759	2.1863
waist circumference	−0.0179	0.9823	0.0238	0.4521	0.9375	1.0291
coronary artery calcification	0.3606	1.4342	0.0618	< 0.0001	1.2706	1.6190

j2)

	coef	HR	se	p	lower	upper
rs412658	0.0411	1.0420	0.1235	0.739	0.8180	1.3274
sex	−0.5829	0.5583	0.2639	0.0272	0.3328	0.9365
age	0.0711	1.0736	0.0125	< 0.0001	1.0476	1.1003
total cholesterol	0.0012	1.0012	0.0054	0.8195	0.9906	1.0120
HDL	−0.0095	0.9905	0.0083	0.2541	0.9745	1.0069
LDL	−0.0036	0.9964	0.0055	0.5188	0.9857	1.0073
triglycerides	0.0004	1.0004	0.0012	0.717	0.9981	1.0028
diabetes	0.2597	1.2966	0.1317	0.0486	1.0016	1.6783
smoking	0.2385	1.2694	0.1172	0.0418	1.0088	1.5971
CRP	0.0649	1.0671	0.1649	0.6938	0.7724	1.4742
BMI	0.0655	1.0677	0.0369	0.0758	0.9932	1.1478
physical activity	0.2190	1.2449	0.1642	0.1822	0.9023	1.7174
waist circumference	−0.0350	0.9656	0.0147	0.0169	0.9382	0.9937
coronary artery calcification	0.2128	1.2371	0.0394	< 0.0001	1.1452	1.3364

j3)

	coef	HR	se	p	lower	upper
rs412658	0.0143	1.0144	0.0999	0.8864	0.8340	1.2338
sex	0.1520	1.1642	0.2066	0.4619	0.7766	1.7452
age	0.0614	1.0633	0.0115	< 0.0001	1.0396	1.0875
total cholesterol	−0.0073	0.9927	0.0044	0.0976	0.9842	1.0013
HDL	0.0019	1.0019	0.0022	0.3697	0.9977	1.0062
LDL	0.0025	1.0025	0.0047	0.5946	0.9933	1.0118
triglycerides	0.0014	1.0014	0.0007	0.0352	1.0001	1.0028
diabetes	0.1405	1.1508	0.1129	0.2134	0.9224	1.4358
smoking	0.1609	1.1746	0.1021	0.1151	0.9615	1.4348
CRP	−0.0506	0.9506	0.0963	0.5989	0.7872	1.1480
BMI	−0.0351	0.9655	0.0320	0.273	0.9068	1.0280
physical activity	0.0608	1.0627	0.1409	0.6659	0.8063	1.4007
waist circumference	0.0078	1.0078	0.0124	0.5304	0.9836	1.0326
coronary artery calcification	0.2977	1.3468	0.0380	< 0.0001	1.2501	1.4510

k1)

	coef	HR	se	p	lower	upper
rs412658	−0.0827	0.9206	0.0814	0.3098	0.7848	1.0799
sex	−0.1129	0.8933	0.1708	0.5088	0.6391	1.2485
age	0.0653	1.0675	0.0094	< 0.0001	1.0480	1.0873
total cholesterol	−0.0002	0.9998	0.0042	0.9623	0.9915	1.0082
HDL	−0.0071	0.9929	0.0058	0.2249	0.9816	1.0044
LDL	−0.0021	0.9979	0.0043	0.6289	0.9895	1.0064
triglycerides	0.0002	1.0002	0.0009	0.846	0.9984	1.0019
smoking	0.2953	1.3435	0.0795	0.0002	1.1497	1.5699
systolic bloodpressure	0.0038	1.0039	0.0042	0.3562	0.9957	1.0121
diastolic bloodpressure	0.0037	1.0037	0.0080	0.6457	0.9880	1.0196
CRP	−0.1095	0.8963	0.1137	0.3353	0.7173	1.1199
BMI	0.0252	1.0255	0.0267	0.3447	0.9733	1.0806
physical activity	0.2023	1.2243	0.1138	0.0753	0.9796	1.5301
waist circumference	−0.0094	0.9906	0.0102	0.3549	0.9710	1.0106
coronary artery calcification	0.2620	1.2995	0.0283	< 0.0001	1.2294	1.3736

k2)

	coef	HR	se	p	lower	upper
rs412658	0.1280	1.1366	0.1474	0.3853	0.8513	1.5174
sex	−0.3694	0.6911	0.3192	0.2471	0.3697	1.2919
age	0.0471	1.0482	0.0168	0.005	1.0143	1.0832
total cholesterol	−0.0054	0.9946	0.0091	0.5549	0.9770	1.0126
HDL	0.0055	1.0055	0.0102	0.5873	0.9857	1.0258
LDL	0.0012	1.0012	0.0092	0.8936	0.9833	1.0195
triglycerides	0.0021	1.0021	0.0012	0.0685	0.9998	1.0044
smoking	−0.1062	0.8992	0.1546	0.4921	0.6642	1.2175
systolic bloodpressure	0.0107	1.0108	0.0061	0.0789	0.9988	1.0229
diastolic bloodpressure	−0.0180	0.9822	0.0117	0.1235	0.9600	1.0049
CRP	0.1100	1.1163	0.0826	0.1827	0.9495	1.3124
BMI	−0.0158	0.9843	0.0440	0.7193	0.9031	1.0729
physical activity	−0.0931	0.9111	0.2015	0.6443	0.6138	1.3525
waist circumference	−0.0155	0.9846	0.0176	0.3783	0.9512	1.0192
coronary artery calcification	0.3200	1.3772	0.0566	< 0.0001	1.2326	1.5387

I1)

	coef	HR	se	p	lower	upper
rs412658	−0.0861	0.9175	0.1160	0.4582	0.7309	1.1518
sex	−0.1712	0.8426	0.2343	0.4648	0.5324	1.3337
age	0.0873	1.0913	0.0155	< 0.0001	1.0587	1.1248
total cholesterol	−0.0037	0.9963	0.0068	0.5833	0.9831	1.0096
HDL	−0.0099	0.9902	0.0090	0.2744	0.9728	1.0079
LDL	−0.0016	0.9984	0.0068	0.8143	0.9851	1.0119
triglycerides	0.0014	1.0014	0.0016	0.3729	0.9983	1.0044
diabetes	0.3780	1.4593	0.1408	0.0073	1.1074	1.9231
systolic bloodpressure	0.0091	1.0091	0.0060	0.13	0.9973	1.0211
diastolic bloodpressure	−0.0071	0.9929	0.0119	0.5526	0.9700	1.0164
CRP	0.0115	1.0115	0.1094	0.9166	0.8163	1.2535
BMI	−0.0065	0.9935	0.0348	0.851	0.9279	1.0637
physical activity	0.0237	1.0240	0.1706	0.8895	0.7330	1.4304
waist circumference	−0.0038	0.9962	0.0140	0.7849	0.9692	1.0239
coronary artery calcification	0.2513	1.2857	0.0409	< 0.0001	1.1867	1.3929

l2)

	coef	HR	se	p	lower	upper
rs412658	−0.0040	0.9960	0.1127	0.972	0.7986	1.2422
sex	−0.4693	0.6254	0.2800	0.0937	0.3613	1.0827
age	0.0554	1.0569	0.0126	< 0.0001	1.0311	1.0834
total cholesterol	−0.0017	0.9983	0.0064	0.7879	0.9858	1.0109
HDL	0.0045	1.0045	0.0079	0.575	0.9889	1.0202
LDL	−0.0006	0.9994	0.0065	0.9239	0.9868	1.0121
triglycerides	0.0014	1.0014	0.0010	0.1905	0.9993	1.0034
diabetes	0.0897	1.0938	0.1200	0.4548	0.8646	1.3838
systolic bloodpressure	0.0063	1.0063	0.0051	0.2143	0.9964	1.0163
diastolic bloodpressure	−0.0087	0.9913	0.0098	0.3743	0.9725	1.0105
CRP	−0.0425	0.9584	0.1580	0.7878	0.7031	1.3063
BMI	0.0594	1.0612	0.0402	0.1399	0.9807	1.1483
physical activity	0.2364	1.2667	0.1540	0.1249	0.9366	1.7131
waist circumference	−0.0208	0.9794	0.0149	0.1616	0.9512	1.0084
coronary artery calcification	0.2716	1.3121	0.0403	< 0.0001	1.2125	1.4198

l3)

	coef	HR	se	p	lower	upper
rs412658	0.1138	1.1205	0.1511	0.4514	0.8333	1.5066
sex	0.1172	1.1243	0.2914	0.6876	0.6352	1.9902
age	0.0407	1.0415	0.0161	0.0116	1.0091	1.0750
total cholesterol	−0.0010	0.9990	0.0066	0.8791	0.9862	1.0120
HDL	−0.0072	0.9928	0.0099	0.4625	0.9738	1.0121
LDL	−0.0002	0.9998	0.0068	0.9731	0.9865	1.0132
triglycerides	0.0007	1.0007	0.0011	0.5343	0.9985	1.0028
diabetes	0.0099	1.0099	0.1748	0.9549	0.7169	1.4227
systolic bloodpressure	0.0076	1.0076	0.0076	0.319	0.9927	1.0228
diastolic bloodpressure	−0.0034	0.9966	0.0139	0.8064	0.9699	1.0240
CRP	−0.0086	0.9914	0.1120	0.9387	0.7961	1.2347
BMI	−0.0325	0.9681	0.0456	0.4769	0.8852	1.0586
physical activity	0.0987	1.1037	0.2052	0.6307	0.7382	1.6501
waist circumference	0.0030	1.0030	0.0184	0.872	0.9675	1.0398
coronary artery calcification	0.2981	1.3473	0.0507	< 0.0001	1.2199	1.4881

m1)

	coef	HR	se	p	lower	upper
rs412658	−0.0749	0.9278	0.1579	0.6352	0.6809	1.2643
sex	0.1285	1.1371	0.2814	0.648	0.6551	1.9738
age	0.0438	1.0448	0.0168	0.0089	1.0110	1.0797
total cholesterol	0.0085	1.0085	0.0077	0.2679	0.9935	1.0238
HDL	−0.0072	0.9928	0.0097	0.4607	0.9741	1.0120
LDL	−0.0089	0.9911	0.0078	0.2506	0.9761	1.0063
triglycerides	0.0002	1.0002	0.0013	0.9055	0.9975	1.0028
diabetes	0.3087	1.3616	0.1884	0.1014	0.9412	1.9699
smoking	0.1942	1.2143	0.1404	0.1666	0.9222	1.5990
systolic bloodpressure	0.0151	1.0152	0.0070	0.0307	1.0014	1.0291
diastolic bloodpressure	−0.0153	0.9848	0.0153	0.3177	0.9557	1.0148
CRP	0.1764	1.1930	0.1528	0.2483	0.8842	1.6097
BMI	0.0007	1.0007	0.0492	0.9891	0.9087	1.1020
physical activity	0.4852	1.6245	0.2125	0.0224	1.0712	2.4637
coronary artery calcification	0.2920	1.3391	0.0505	< 0.0001	1.2128	1.4785

m2)

	coef	HR	se	p	lower	upper
rs412658	0.0014	1.0014	0.0788	0.9854	0.8582	1.1687
sex	−0.1110	0.8949	0.1396	0.4263	0.6808	1.1765
age	0.0630	1.0650	0.0092	< 0.0001	1.0460	1.0844
total cholesterol	−0.0048	0.9952	0.0042	0.2616	0.9870	1.0036
HDL	−0.0046	0.9954	0.0057	0.4227	0.9843	1.0067
LDL	0.0009	1.0009	0.0043	0.842	0.9924	1.0094
triglycerides	0.0013	1.0013	0.0007	0.0861	0.9998	1.0027
diabetes	0.1144	1.1212	0.0844	0.1751	0.9503	1.3230
smoking	0.1479	1.1594	0.0809	0.0676	0.9893	1.3588
systolic bloodpressure	0.0049	1.0049	0.0038	0.1976	0.9975	1.0124
diastolic bloodpressure	−0.0003	0.9997	0.0072	0.9663	0.9857	1.0139
CRP	−0.0355	0.9651	0.0841	0.6727	0.8184	1.1381
BMI	−0.0004	0.9996	0.0139	0.9771	0.9726	1.0273
physical activity	0.0354	1.0360	0.1108	0.7494	0.8338	1.2873
coronary artery calcification	0.2660	1.3047	0.0288	< 0.0001	1.2330	1.3806

n1)

	coef	HR	se	p	lower	upper
rs412658	−0.1754	0.8391	0.1692	0.3	0.6022	1.1692
sex	0.0103	1.0104	0.3369	0.9755	0.5221	1.9554
age	0.0657	1.0680	0.0194	0.0007	1.0281	1.1093
total cholesterol	0.0106	1.0106	0.0082	0.1963	0.9946	1.0270
HDL	−0.0153	0.9848	0.0105	0.144	0.9648	1.0052
LDL	−0.0136	0.9865	0.0082	0.0962	0.9709	1.0024
triglycerides	−0.0006	0.9994	0.0015	0.683	0.9964	1.0023
diabetes	0.2299	1.2585	0.2374	0.3328	0.7903	2.0040
smoking	0.2517	1.2862	0.1471	0.0871	0.9640	1.7161
systolic bloodpressure	0.0067	1.0067	0.0076	0.379	0.9918	1.0219
diastolic bloodpressure	−0.0015	0.9985	0.0163	0.9275	0.9672	1.0309
CRP	−0.0271	0.9733	0.1231	0.8259	0.7647	1.2388
physical activity	0.5691	1.7666	0.2348	0.0154	1.1150	2.7990
waist circumference	−0.0255	0.9748	0.0183	0.1638	0.9405	1.0105
coronary artery calcification	0.3792	1.4611	0.0601	< 0.0001	1.2987	1.6439

n2)

	coef	HR	se	p	lower	upper
rs412658	0.0125	1.0126	0.0780	0.8729	0.8690	1.1799
sex	−0.1628	0.8498	0.1404	0.2462	0.6454	1.1189
age	0.0584	1.0602	0.0089	< 0.0001	1.0418	1.0788
total cholesterol	−0.0053	0.9947	0.0041	0.1967	0.9867	1.0028
HDL	−0.0019	0.9981	0.0056	0.7328	0.9873	1.0090
LDL	0.0019	1.0019	0.0042	0.6529	0.9937	1.0102
triglycerides	0.0015	1.0015	0.0007	0.0337	1.0001	1.0029
diabetes	0.1307	1.1397	0.0828	0.1143	0.9690	1.3404
smoking	0.1654	1.1799	0.0788	0.0357	1.0111	1.3769
systolic bloodpressure	0.0062	1.0062	0.0038	0.1007	0.9988	1.0136
diastolic bloodpressure	−0.0057	0.9943	0.0072	0.4224	0.9804	1.0083
CRP	0.0037	1.0037	0.0851	0.9657	0.8494	1.1859
physical activity	0.0435	1.0445	0.1087	0.6891	0.8440	1.2926
waist circumference	−0.0042	0.9958	0.0057	0.4623	0.9848	1.0070
coronary artery calcification	0.2512	1.2856	0.0275	< 0.0001	1.2182	1.3568

S4I Table. Results of Cox regression models for rs755017.

coef: coefficient, HR: Hazard Ratio, se: Standard error,
lower/upper: lower/upper boundarie of the 95% confidence interval
a) crude, b) adjusted, c1) young age, c2) older age, d1) male, d2) female,
e1) low hsCRP, e2) intermediate hsCRP, e3) high hsCRP, f1) low total cholesterol,
f2) high total cholesterol, g1) low LDL, g2) high LDL, h1) normal HDL, h2) high HDL,
i1) low triglycerides, i2) high triglycerides, j1) ideal blood pressure,
j2) normal/high normal blood pressure, j3) hypertension, k1) no diabetes, k2) diabetes,
l1) never smoker, l2) former smoker, l3) current smoker, m1) normal waist circumference,
m2) high waist circumference, n1) normal BMI, n2) high BMI

a)

	coef	HR	se	p	lower	upper
rs755017	−0.0323	0.9682	0.1009	0.7486	0.7945	1.1799

b)

	coef	HR	se	p	lower	upper
rs755017	0.0084	1.0085	0.1049	0.9359	0.8211	1.2386
sex	−0.1119	0.8941	0.1490	0.4526	0.6677	1.1973
age	0.0593	1.0611	0.0081	< 0.0001	1.0444	1.0781
total cholesterol	−0.0022	0.9978	0.0038	0.5547	0.9904	1.0052
HDL	−0.0044	0.9956	0.0050	0.379	0.9860	1.0054
LDL	−0.0010	0.9990	0.0038	0.7987	0.9915	1.0066
triglycerides	0.0011	1.0011	0.0006	0.0893	0.9998	1.0024
diabetes	0.1551	1.1678	0.0799	0.0522	0.9985	1.3657
smoking	0.1816	1.1992	0.0695	0.009	1.0464	1.3742
systolic bloodpressure	0.0071	1.0071	0.0034	0.0368	1.0004	1.0138
diastolic bloodpressure	−0.0049	0.9951	0.0065	0.4498	0.9824	1.0079
CRP	0.0010	1.0010	0.0702	0.9883	0.8724	1.1486
BMI	0.0046	1.0046	0.0221	0.8367	0.9619	1.0491
physical activity	0.1602	1.1737	0.0984	0.1035	0.9679	1.4233
waist circumference	−0.0083	0.9917	0.0087	0.3398	0.9749	1.0088
coronary artery calcification	0.2753	1.3169	0.0250	< 0.0001	1.2540	1.3830

c1)

	coef	HR	se	p	lower	upper
rs755017	−0.2248	0.7987	0.2036	0.2696	0.5358	1.1904
sex	−0.1157	0.8908	0.2847	0.6845	0.5098	1.5563
total cholesterol	0.0019	1.0019	0.0068	0.7845	0.9886	1.0153
HDL	−0.0138	0.9863	0.0102	0.1764	0.9667	1.0062
LDL	−0.0012	0.9988	0.0069	0.864	0.9853	1.0125
triglycerides	0.0004	1.0004	0.0013	0.7738	0.9978	1.0030
diabetes	0.0023	1.0023	0.1973	0.9906	0.6808	1.4756
smoking	0.2969	1.3457	0.1345	0.0272	1.0339	1.7515
systolic bloodpressure	−0.0048	0.9952	0.0089	0.5922	0.9780	1.0128
diastolic bloodpressure	0.0194	1.0196	0.0163	0.2324	0.9876	1.0527
CRP	0.0995	1.1047	0.1669	0.551	0.7964	1.5323
BMI	0.0097	1.0097	0.0467	0.8362	0.9214	1.1064
physical activity	0.2275	1.2555	0.2142	0.2882	0.8250	1.9107
waist circumference	−0.0149	0.9852	0.0183	0.416	0.9505	1.0212
coronary artery calcification	0.2901	1.3366	0.0461	< 0.0001	1.2211	1.4630

c2)

	coef	HR	se	p	lower	upper
rs755017	0.0785	1.0816	0.1234	0.5249	0.8492	1.3777
sex	0.3479	1.4160	0.1749	0.0467	1.0051	1.9948
total cholesterol	−0.0045	0.9956	0.0042	0.2942	0.9873	1.0039
HDL	−0.0005	0.9995	0.0047	0.9125	0.9902	1.0088
LDL	0.0000	1.0000	0.0044	0.9932	0.9915	1.0086
triglycerides	0.0011	1.0011	0.0007	0.0999	0.9998	1.0025
diabetes	0.1833	1.2012	0.0885	0.0384	1.0099	1.4287
smoking	0.0803	1.0837	0.0819	0.3267	0.9229	1.2724
systolic bloodpressure	0.0139	1.0140	0.0036	0.0001	1.0069	1.0211
diastolic bloodpressure	−0.0181	0.9821	0.0071	0.0104	0.9686	0.9958
CRP	−0.0067	0.9933	0.0736	0.9276	0.8599	1.1475
BMI	−0.0104	0.9897	0.0257	0.686	0.9411	1.0408
physical activity	0.1383	1.1483	0.1112	0.2139	0.9233	1.4281
waist circumference	−0.0011	0.9989	0.0100	0.9135	0.9796	1.0186
coronary artery calcification	0.2891	1.3352	0.0294	< 0.0001	1.2604	1.4144

d1)

	coef	HR	se	p	lower	upper
rs755017	−0.0198	0.9804	0.1283	0.8776	0.7624	1.2608
age	0.0433	1.0443	0.0096	< 0.0001	1.0248	1.0642
total cholesterol	−0.0059	0.9941	0.0048	0.2182	0.9848	1.0035
HDL	0.0019	1.0019	0.0062	0.7523	0.9899	1.0141
LDL	0.0027	1.0027	0.0049	0.5774	0.9932	1.0124
triglycerides	0.0015	1.0015	0.0008	0.0543	1.0000	1.0030
diabetes	0.1714	1.1870	0.0910	0.0596	0.9931	1.4187
smoking	0.1085	1.1146	0.0892	0.2238	0.9358	1.3275
systolic bloodpressure	0.0048	1.0048	0.0043	0.2607	0.9965	1.0132
diastolic bloodpressure	−0.0025	0.9975	0.0080	0.7503	0.9820	1.0132
CRP	0.0029	1.0029	0.0786	0.9709	0.8597	1.1699
BMI	0.0345	1.0351	0.0291	0.2349	0.9778	1.0958
physical activity	0.1660	1.1806	0.1210	0.17	0.9314	1.4965
waist circumference	−0.0126	0.9874	0.0112	0.26	0.9660	1.0094
coronary artery calcification	0.2972	1.3461	0.0333	< 0.0001	1.2612	1.4368

d2)

	coef	HR	se	p	lower	upper
rs755017	0.0145	1.0146	0.1825	0.9366	0.7095	1.4510
age	0.0985	1.1035	0.0152	< 0.0001	1.0711	1.1369
total cholesterol	0.0016	1.0016	0.0056	0.7736	0.9908	1.0126
HDL	−0.0136	0.9865	0.0081	0.094	0.9709	1.0023
LDL	−0.0055	0.9945	0.0057	0.3296	0.9835	1.0056
triglycerides	0.0012	1.0012	0.0013	0.3754	0.9986	1.0038
diabetes	0.1164	1.1235	0.1717	0.4976	0.8025	1.5729
smoking	0.3316	1.3932	0.1096	0.0025	1.1239	1.7271
systolic bloodpressure	0.0095	1.0095	0.0057	0.0941	0.9984	1.0208
diastolic bloodpressure	−0.0066	0.9935	0.0117	0.575	0.9709	1.0165
CRP	0.0098	1.0098	0.1478	0.9473	0.7558	1.3492
BMI	−0.0287	0.9717	0.0348	0.4095	0.9075	1.0403
physical activity	0.1512	1.1632	0.1708	0.376	0.8323	1.6255
waist circumference	−0.0069	0.9931	0.0141	0.6224	0.9661	1.0209
coronary artery calcification	0.2361	1.2663	0.0387	< 0.0001	1.1739	1.3660

e1)

	coef	HR	se	p	lower	upper
rs755017	−0.0612	0.9407	0.1238	0.6211	0.7380	1.1989
sex	−0.1781	0.8368	0.1815	0.3265	0.5863	1.1944
age	0.0683	1.0707	0.0098	< 0.0001	1.0503	1.0915
total cholesterol	0.0000	1.0000	0.0043	0.9915	0.9916	1.0086
HDL	−0.0062	0.9938	0.0058	0.2828	0.9826	1.0051
LDL	−0.0038	0.9962	0.0044	0.3893	0.9876	1.0049
triglycerides	0.0011	1.0011	0.0007	0.1465	0.9996	1.0025
diabetes	0.1293	1.1381	0.1013	0.2018	0.9331	1.3880
smoking	0.1753	1.1916	0.0859	0.0414	1.0069	1.4102
systolic bloodpressure	0.0076	1.0077	0.0043	0.0736	0.9993	1.0161
diastolic bloodpressure	−0.0043	0.9957	0.0083	0.6025	0.9797	1.0120
BMI	0.0386	1.0394	0.0280	0.1685	0.9838	1.0981
physical activity	0.1813	1.1988	0.1184	0.1256	0.9506	1.5118
waist circumference	−0.0166	0.9835	0.0105	0.1124	0.9635	1.0039
coronary artery calcification	0.2713	1.3117	0.0301	< 0.0001	1.2366	1.3913

e2)

	coef	HR	se	p	lower	upper
rs755017	0.4362	1.5469	0.3279	0.1834	0.8135	2.9414
sex	0.7351	2.0857	0.4664	0.115	0.8361	5.2028
age	0.0946	1.0992	0.025	0.0002	1.0467	1.1543
total cholesterol	−0.0061	0.9939	0.013	0.6387	0.9688	1.0196
HDL	−0.0116	0.9884	0.0169	0.4909	0.9563	1.0217
LDL	0.006	1.006	0.0123	0.6275	0.982	1.0306
triglycerides	−0.0038	0.9962	0.0027	0.1541	0.991	1.0014
diabetes	0.3347	1.3975	0.1914	0.0804	0.9603	2.0337
smoking	0.1677	1.1826	0.211	0.4268	0.782	1.7883
systolic bloodpressure	−0.0046	0.9954	0.0096	0.6313	0.977	1.0142
diastolic bloodpressure	0.0104	1.0105	0.0172	0.5438	0.977	1.0452
BMI	−0.133	0.8755	0.0728	0.0678	0.759	1.0098
physical activity	−0.5759	0.5622	0.2808	0.0403	0.3243	0.9748
waist circumference	0.0609	1.0628	0.0292	0.037	1.0037	1.1254
coronary artery calcification	0.3038	1.355	0.0764	0.0001	1.1665	1.5739

e3)

	coef	HR	se	p	lower	upper
rs755017	0.0205	1.0207	0.2555	0.936	0.6187	1.6841
sex	−0.3125	0.7316	0.3537	0.377	0.3657	1.4634
age	0.0078	1.0078	0.0186	0.6747	0.9718	1.0452
total cholesterol	−0.0048	0.9952	0.0066	0.4669	0.9825	1.0081
HDL	0.0008	1.0008	0.0035	0.8188	0.9940	1.0076
LDL	0.0031	1.0031	0.0068	0.6493	0.9898	1.0166
triglycerides	0.0022	1.0022	0.0013	0.0752	0.9998	1.0047
diabetes	0.1658	1.1803	0.1741	0.3409	0.8391	1.6602
smoking	0.1364	1.1461	0.1594	0.3922	0.8386	1.5663
systolic bloodpressure	0.0066	1.0066	0.0073	0.3641	0.9923	1.0212
diastolic bloodpressure	−0.0189	0.9813	0.0149	0.2059	0.9531	1.0104
BMI	−0.0312	0.9693	0.0484	0.5188	0.8816	1.0657
physical activity	0.4797	1.6156	0.2438	0.0491	1.0018	2.6055
waist circumference	−0.0173	0.9829	0.0203	0.394	0.9446	1.0227
coronary artery calcification	0.2921	1.3392	0.0585	< 0.0001	1.1941	1.5020

f1)

	coef	HR	se	p	lower	upper
rs755017	0.0983	1.1033	0.2123	0.6435	0.7277	1.6727
sex	0.4197	1.5216	0.2715	0.1222	0.8936	2.5908
age	0.0726	1.0753	0.0162	< 0.0001	1.0417	1.1099
HDL	−0.0065	0.9935	0.0085	0.4439	0.9770	1.0103
LDL	−0.0046	0.9954	0.0048	0.3292	0.9861	1.0047
triglycerides	0.0007	1.0007	0.0013	0.6105	0.9981	1.0033
diabetes	0.1999	1.2212	0.1501	0.1829	0.9101	1.6388
smoking	−0.0024	0.9976	0.1322	0.9857	0.7699	1.2928
systolic bloodpressure	0.0035	1.0035	0.0068	0.6059	0.9903	1.0169
diastolic bloodpressure	−0.0027	0.9973	0.0132	0.8372	0.9719	1.0233
CRP	−0.0395	0.9612	0.1219	0.7457	0.7569	1.2207
BMI	−0.1060	0.8994	0.0432	0.0141	0.8263	0.9789
physical activity	0.2524	1.2871	0.1921	0.1889	0.8833	1.8757
waist circumference	0.0304	1.0308	0.0160	0.0571	0.9991	1.0636
coronary artery calcification	0.2990	1.3485	0.0480	< 0.0001	1.2274	1.4816

f2)

	coef	HR	se	p	lower	upper
rs755017	−0.0414	0.9594	0.1213	0.7328	0.7564	1.2170
sex	−0.3100	0.7335	0.1775	0.0807	0.5180	1.0386
age	0.0556	1.0571	0.0094	< 0.0001	1.0378	1.0769
HDL	−0.0058	0.9942	0.0044	0.1847	0.9856	1.0028
LDL	−0.0029	0.9971	0.0019	0.1306	0.9933	1.0009
triglycerides	0.0007	1.0007	0.0005	0.1343	0.9998	1.0017
diabetes	0.1226	1.1305	0.0956	0.1993	0.9374	1.3633
smoking	0.2637	1.3018	0.0827	0.0014	1.1070	1.5308
systolic bloodpressure	0.0079	1.0080	0.0040	0.0458	1.0001	1.0158
diastolic bloodpressure	−0.0066	0.9935	0.0076	0.3898	0.9787	1.0084
CRP	0.0145	1.0146	0.0849	0.8645	0.8591	1.1983
BMI	0.0477	1.0489	0.0255	0.0618	0.9977	1.1027
physical activity	0.1220	1.1297	0.1166	0.2956	0.8989	1.4199
waist circumference	−0.0222	0.9781	0.0102	0.0291	0.9588	0.9977
coronary artery calcification	0.2661	1.3049	0.0297	< 0.0001	1.2311	1.3830

g1)

	coef	HR	se	p	lower	upper
rs755017	0.0459	1.0469	0.2235	0.8374	0.6756	1.6225
sex	0.1769	1.1935	0.2961	0.5501	0.6681	2.1323
age	0.0814	1.0849	0.0177	< 0.0001	1.0480	1.1230
total cholesterol	−0.0019	0.9981	0.0046	0.6817	0.9891	1.0072
HDL	−0.0012	0.9988	0.0072	0.8643	0.9848	1.0130
triglycerides	0.0017	1.0017	0.0007	0.0147	1.0003	1.0032
diabetes	0.2125	1.2367	0.1674	0.2043	0.8909	1.7169
smoking	0.1865	1.2051	0.1433	0.1929	0.9101	1.5957
systolic bloodpressure	0.0014	1.0014	0.0073	0.8472	0.9871	1.0159
diastolic bloodpressure	−0.0056	0.9944	0.0139	0.6865	0.9676	1.0219
CRP	−0.2685	0.7645	0.2299	0.2429	0.4872	1.1998
BMI	−0.0766	0.9262	0.0459	0.0948	0.8466	1.0134
physical activity	0.4850	1.6242	0.2115	0.0219	1.0730	2.4586
waist circumference	0.0223	1.0226	0.0173	0.1963	0.9885	1.0578
coronary artery calcification	0.2685	1.3080	0.0516	< 0.0001	1.1821	1.4472

g2)

	coef	HR	se	p	lower	upper
rs755017	−0.0251	0.9752	0.1191	0.8331	0.7721	1.2317
sex	−0.1887	0.8281	0.1719	0.2725	0.5912	1.1599
age	0.0543	1.0558	0.0092	< 0.0001	1.0369	1.0751
total cholesterol	−0.0032	0.9968	0.0019	0.0835	0.9932	1.0004
HDL	−0.0040	0.9960	0.0047	0.3939	0.9870	1.0052
triglycerides	0.0010	1.0010	0.0007	0.1718	0.9996	1.0023
diabetes	0.1328	1.1420	0.0922	0.1496	0.9533	1.3681
smoking	0.2002	1.2216	0.0801	0.0125	1.0441	1.4294
systolic bloodpressure	0.0078	1.0078	0.0039	0.0443	1.0002	1.0155
diastolic bloodpressure	−0.0042	0.9958	0.0075	0.5764	0.9813	1.0106
CRP	0.0895	1.0936	0.0848	0.2911	0.9262	1.2913
BMI	0.0257	1.0260	0.0250	0.3041	0.9770	1.0775
physical activity	0.0813	1.0847	0.1127	0.4707	0.8697	1.3528
waist circumference	−0.0168	0.9833	0.0099	0.0889	0.9644	1.0026
coronary artery calcification	0.2765	1.3185	0.0290	< 0.0001	1.2457	1.3955

h1)

	coef	HR	se	p	lower	upper
rs755017	−0.0234	0.9769	0.1213	0.847	0.7702	1.2390
sex	−0.1307	0.8774	0.1728	0.4493	0.6254	1.2312
age	0.0700	1.0725	0.0098	< 0.0001	1.0521	1.0934
total cholesterol	−0.0018	0.9982	0.0035	0.5944	0.9914	1.0050
LDL	−0.0005	0.9995	0.0036	0.8802	0.9925	1.0065
triglycerides	−0.0001	0.9999	0.0008	0.8581	0.9982	1.0015
diabetes	0.1712	1.1868	0.0981	0.0811	0.9791	1.4385
smoking	0.2386	1.2695	0.0827	0.0039	1.0796	1.4927
systolic bloodpressure	0.0094	1.0094	0.0039	0.0157	1.0018	1.0172
diastolic bloodpressure	−0.0040	0.9960	0.0076	0.6008	0.9813	1.0110
CRP	−0.0787	0.9243	0.0993	0.4281	0.7608	1.1229
BMI	0.0201	1.0203	0.0260	0.4408	0.9695	1.0736
physical activity	0.1795	1.1966	0.1147	0.1174	0.9558	1.4982
waist circumference	−0.0072	0.9928	0.0104	0.489	0.9728	1.0133
coronary artery calcification	0.2550	1.2905	0.0284	< 0.0001	1.2205	1.3645

h2)

	coef	HR	se	p	lower	upper
rs755017	0.0387	1.0394	0.2145	0.8569	0.6827	1.5827
sex	−0.2654	0.7669	0.2806	0.3443	0.4425	1.3292
age	0.0343	1.0349	0.0147	0.0193	1.0056	1.0651
total cholesterol	−0.0046	0.9954	0.0058	0.4335	0.9841	1.0069
LDL	0.0006	1.0006	0.0063	0.9281	0.9883	1.0130
triglycerides	0.0021	1.0021	0.0008	0.0122	1.0005	1.0037
diabetes	0.0731	1.0758	0.1425	0.608	0.8137	1.4224
smoking	0.0442	1.0452	0.1349	0.7431	0.8024	1.3615
systolic bloodpressure	0.0007	1.0007	0.0069	0.9225	0.9873	1.0143
diastolic bloodpressure	−0.0061	0.9939	0.0133	0.6473	0.9683	1.0202
CRP	0.1728	1.1887	0.1152	0.1337	0.9483	1.4899
BMI	−0.0331	0.9674	0.0426	0.4364	0.8899	1.0516
physical activity	0.1119	1.1184	0.1935	0.563	0.7654	1.6343
waist circumference	−0.0154	0.9847	0.0162	0.342	0.9540	1.0165
coronary artery calcification	0.3319	1.3936	0.0526	< 0.0001	1.2571	1.5449

i1)

	coef	HR	se	p	lower	upper
rs755017	−0.0083	0.9917	0.1457	0.9545	0.7453	1.3196
sex	−0.0971	0.9074	0.2006	0.6282	0.6125	1.3445
age	0.0732	1.0759	0.0113	< 0.0001	1.0523	1.1001
total cholesterol	−0.0014	0.9986	0.0052	0.7934	0.9884	1.0089
HDL	−0.0025	0.9975	0.0064	0.7007	0.9851	1.0101
LDL	−0.0028	0.9972	0.0054	0.602	0.9867	1.0078
diabetes	0.0410	1.0418	0.1258	0.7448	0.8141	1.3331
smoking	0.3057	1.3576	0.0943	0.0012	1.1286	1.6332
systolic bloodpressure	0.0046	1.0046	0.0046	0.3251	0.9955	1.0137
diastolic bloodpressure	0.0126	1.0127	0.0091	0.1637	0.9949	1.0309
CRP	−0.0533	0.9481	0.0893	0.5503	0.7959	1.1293
BMI	0.0283	1.0287	0.0311	0.3629	0.9678	1.0935
physical activity	0.3496	1.4184	0.1335	0.0089	1.0918	1.8428
waist circumference	−0.0150	0.9851	0.0119	0.2073	0.9624	1.0084
coronary artery calcification	0.2781	1.3206	0.0330	< 0.0001	1.2379	1.4088

i2)

	coef	HR	se	p	lower	upper
rs755017	0.0361	1.0368	0.1538	0.8143	0.7669	1.4016
sex	−0.1199	0.8870	0.2267	0.5968	0.5688	1.3831
age	0.0443	1.0453	0.0116	0.0001	1.0218	1.0695
total cholesterol	0.0019	1.0019	0.0034	0.5623	0.9954	1.0086
HDL	−0.0175	0.9826	0.0070	0.0126	0.9692	0.9962
LDL	−0.0039	0.9961	0.0035	0.2708	0.9892	1.0030
diabetes	0.2511	1.2855	0.1061	0.018	1.0440	1.5827
smoking	0.0568	1.0585	0.1050	0.5881	0.8617	1.3002
systolic bloodpressure	0.0099	1.0100	0.0049	0.0429	1.0003	1.0197
diastolic bloodpressure	−0.0247	0.9756	0.0097	0.0113	0.9572	0.9944
CRP	0.1161	1.1231	0.1348	0.3891	0.8623	1.4629
BMI	−0.0235	0.9768	0.0326	0.471	0.9163	1.0412
physical activity	−0.1227	0.8845	0.1459	0.4002	0.6645	1.1773
waist circumference	0.0003	1.0003	0.0129	0.9802	0.9753	1.0260
coronary artery calcification	0.2676	1.3068	0.0386	< 0.0001	1.2115	1.4096

j1)

	coef	HR	se	p	lower	upper
rs755017	0.1768	1.1934	0.2898	0.5418	0.6763	2.1059
sex	−0.1076	0.8980	0.3629	0.7669	0.4410	1.8288
age	0.0558	1.0574	0.0188	0.0029	1.0193	1.0970
total cholesterol	0.0147	1.0148	0.0107	0.1718	0.9937	1.0363
HDL	−0.0244	0.9759	0.0131	0.0633	0.9511	1.0014
LDL	−0.0150	0.9852	0.0103	0.1448	0.9655	1.0052
triglycerides	0.0024	1.0024	0.0024	0.33	0.9976	1.0072
diabetes	−0.0621	0.9398	0.2251	0.7827	0.6046	1.4609
smoking	0.2250	1.2524	0.1753	0.1993	0.8882	1.7659
CRP	0.1147	1.1215	0.1483	0.4392	0.8387	1.4998
BMI	0.0085	1.0086	0.0593	0.8855	0.8979	1.1329
physical activity	0.2469	1.2800	0.2638	0.3493	0.7633	2.1466
waist circumference	−0.0152	0.9849	0.0237	0.5202	0.9402	1.0317
coronary artery calcification	0.3623	1.4367	0.0619	< 0.0001	1.2726	1.6218

j2)

	coef	HR	se	p	lower	upper
rs755017	−0.1310	0.8772	0.1825	0.4729	0.6135	1.2544
sex	−0.5938	0.5522	0.2644	0.0247	0.3289	0.9272
age	0.0713	1.0739	0.0126	< 0.0001	1.0478	1.1007
total cholesterol	0.0013	1.0013	0.0055	0.806	0.9907	1.0121
HDL	−0.0093	0.9907	0.0083	0.2654	0.9747	1.0071
LDL	−0.0038	0.9962	0.0056	0.4977	0.9854	1.0072
triglycerides	0.0005	1.0005	0.0012	0.7016	0.9981	1.0028
diabetes	0.2555	1.2911	0.1316	0.0523	0.9975	1.6712
smoking	0.2453	1.2781	0.1179	0.0375	1.0143	1.6104
CRP	0.0659	1.0681	0.1648	0.6893	0.7733	1.4752
BMI	0.0666	1.0688	0.0369	0.071	0.9943	1.1490
physical activity	0.2287	1.2570	0.1648	0.1651	0.9101	1.7362
waist circumference	−0.0353	0.9653	0.0147	0.016	0.9379	0.9934
coronary artery calcification	0.2119	1.2361	0.0393	< 0.0001	1.1444	1.3351

j3)

	coef	HR	se	p	lower	upper
rs755017	0.0330	1.0335	0.1436	0.8184	0.7800	1.3694
sex	0.1519	1.1640	0.2065	0.462	0.7766	1.7447
age	0.0614	1.0633	0.0115	< 0.0001	1.0396	1.0875
total cholesterol	−0.0073	0.9927	0.0044	0.0965	0.9841	1.0013
HDL	0.0019	1.0019	0.0022	0.3714	0.9977	1.0062
LDL	0.0025	1.0025	0.0047	0.5903	0.9933	1.0118
triglycerides	0.0014	1.0014	0.0007	0.0341	1.0001	1.0028
diabetes	0.1408	1.1512	0.1128	0.212	0.9228	1.4362
smoking	0.1616	1.1753	0.1021	0.1135	0.9622	1.4357
CRP	−0.0509	0.9504	0.0962	0.597	0.7871	1.1476
BMI	−0.0351	0.9655	0.0320	0.2732	0.9068	1.0281
physical activity	0.0606	1.0625	0.1408	0.667	0.8062	1.4002
waist circumference	0.0077	1.0077	0.0124	0.5334	0.9836	1.0325
coronary artery calcification	0.2973	1.3462	0.0379	< 0.0001	1.2498	1.4502

k1)

	coef	HR	se	p	lower	upper
rs755017	0.0466	1.0477	0.1248	0.7088	0.8204	1.3380
sex	−0.1048	0.9005	0.1707	0.5395	0.6444	1.2585
age	0.0649	1.0671	0.0094	< 0.0001	1.0477	1.0869
total cholesterol	−0.0001	0.9999	0.0042	0.9842	0.9917	1.0082
HDL	−0.0073	0.9927	0.0058	0.2092	0.9814	1.0041
LDL	−0.0022	0.9978	0.0043	0.609	0.9895	1.0062
triglycerides	0.0002	1.0002	0.0009	0.8405	0.9984	1.0019
smoking	0.2925	1.3397	0.0794	0.0002	1.1466	1.5654
systolic bloodpressure	0.0040	1.0040	0.0042	0.3364	0.9958	1.0122
diastolic bloodpressure	0.0036	1.0036	0.0080	0.6513	0.9880	1.0196
CRP	−0.1117	0.8943	0.1141	0.3274	0.7151	1.1184
BMI	0.0241	1.0244	0.0266	0.3638	0.9724	1.0792
physical activity	0.2031	1.2253	0.1138	0.0742	0.9804	1.5313
waist circumference	−0.0090	0.9911	0.0102	0.3786	0.9715	1.0111
coronary artery calcification	0.2630	1.3008	0.0283	< 0.0001	1.2307	1.3750

k2)

	coef	HR	se	p	lower	upper
rs755017	0.0023	1.0023	0.1999	0.9906	0.6774	1.4832
sex	−0.3646	0.6945	0.3204	0.2552	0.3706	1.3014
age	0.0470	1.0481	0.0168	0.0051	1.0142	1.0831
total cholesterol	−0.0053	0.9947	0.0092	0.5618	0.9769	1.0128
HDL	0.0055	1.0055	0.0103	0.5915	0.9855	1.0259
LDL	0.0013	1.0013	0.0093	0.8887	0.9833	1.0196
triglycerides	0.0021	1.0021	0.0012	0.0652	0.9999	1.0044
smoking	−0.1050	0.9003	0.1552	0.4986	0.6642	1.2204
systolic bloodpressure	0.0108	1.0109	0.0061	0.0781	0.9988	1.0231
diastolic bloodpressure	−0.0184	0.9818	0.0117	0.1158	0.9595	1.0045
CRP	0.1110	1.1174	0.0828	0.1802	0.9500	1.3143
BMI	−0.0184	0.9818	0.0444	0.6783	0.9000	1.0710
physical activity	−0.0935	0.9108	0.2022	0.6439	0.6128	1.3537
waist circumference	−0.0152	0.9849	0.0177	0.3906	0.9513	1.0197
coronary artery calcification	0.3180	1.3743	0.0564	< 0.0001	1.2306	1.5349

I1)

	coef	HR	se	p	lower	upper
rs755017	0.0136	1.0136	0.1890	0.9428	0.6999	1.4681
sex	−0.1545	0.8569	0.2339	0.5089	0.5418	1.3551
age	0.0876	1.0915	0.0155	< 0.0001	1.0589	1.1252
total cholesterol	−0.0038	0.9962	0.0067	0.5744	0.9831	1.0095
HDL	−0.0099	0.9901	0.0090	0.272	0.9728	1.0078
LDL	−0.0015	0.9985	0.0068	0.8216	0.9852	1.0119
triglycerides	0.0014	1.0014	0.0015	0.358	0.9984	1.0045
diabetes	0.3776	1.4588	0.1409	0.0074	1.1067	1.9230
systolic bloodpressure	0.0089	1.0089	0.0060	0.1385	0.9971	1.0208
diastolic bloodpressure	−0.0066	0.9934	0.0119	0.5794	0.9705	1.0169
CRP	0.0151	1.0152	0.1087	0.8896	0.8204	1.2563
BMI	−0.0090	0.9911	0.0346	0.796	0.9261	1.0607
physical activity	0.0217	1.0220	0.1705	0.8986	0.7316	1.4276
waist circumference	−0.0028	0.9972	0.0139	0.8403	0.9704	1.0248
coronary artery calcification	0.2533	1.2882	0.0408	< 0.0001	1.1892	1.3955

l2)

	coef	HR	se	p	lower	upper
rs755017	0.0470	1.0482	0.1590	0.7674	0.7675	1.4314
sex	−0.4634	0.6291	0.2804	0.0984	0.3631	1.0900
age	0.0553	1.0568	0.0126	< 0.0001	1.0310	1.0833
total cholesterol	−0.0017	0.9983	0.0064	0.7897	0.9858	1.0109
HDL	0.0043	1.0043	0.0080	0.5862	0.9888	1.0201
LDL	−0.0007	0.9993	0.0065	0.9176	0.9867	1.0121
triglycerides	0.0014	1.0014	0.0010	0.191	0.9993	1.0034
diabetes	0.0906	1.0948	0.1201	0.4504	0.8653	1.3853
systolic bloodpressure	0.0063	1.0063	0.0050	0.2124	0.9964	1.0163
diastolic bloodpressure	−0.0087	0.9914	0.0098	0.3761	0.9725	1.0106
CRP	−0.0434	0.9576	0.1577	0.7834	0.7029	1.3045
BMI	0.0591	1.0609	0.0402	0.1421	0.9804	1.1479
physical activity	0.2341	1.2637	0.1542	0.1289	0.9342	1.7095
waist circumference	−0.0207	0.9795	0.0149	0.165	0.9514	1.0086
coronary artery calcification	0.2719	1.3125	0.0403	< 0.0001	1.2129	1.4203

I3)

	coef	HR	se	p	lower	upper
rs755017	−0.1480	0.8624	0.2154	0.4918	0.5654	1.3153
sex	0.1131	1.1198	0.2911	0.6976	0.6329	1.9811
age	0.0421	1.0430	0.0161	0.0089	1.0106	1.0764
total cholesterol	−0.0019	0.9981	0.0066	0.772	0.9853	1.0110
HDL	−0.0061	0.9940	0.0099	0.5381	0.9749	1.0133
LDL	0.0007	1.0007	0.0068	0.9214	0.9874	1.0141
triglycerides	0.0008	1.0008	0.0011	0.4698	0.9987	1.0029
diabetes	0.0204	1.0206	0.1748	0.9069	0.7246	1.4376
systolic bloodpressure	0.0070	1.0070	0.0076	0.3585	0.9921	1.0221
diastolic bloodpressure	−0.0033	0.9967	0.0138	0.8096	0.9701	1.0240
CRP	−0.0081	0.9919	0.1115	0.9422	0.7973	1.2341
BMI	−0.0322	0.9683	0.0459	0.4827	0.8851	1.0594
physical activity	0.0992	1.1042	0.2051	0.6288	0.7387	1.6508
waist circumference	0.0030	1.0030	0.0184	0.8726	0.9674	1.0398
coronary artery calcification	0.2999	1.3497	0.0507	< 0.0001	1.2219	1.4909

m1)

	coef	HR	se	p	lower	upper
rs755017	−0.1623	0.8502	0.2267	0.4741	0.5452	1.3258
sex	0.1258	1.1340	0.2816	0.6552	0.6530	1.9693
age	0.0437	1.0447	0.0167	0.0087	1.0111	1.0794
total cholesterol	0.0085	1.0085	0.0077	0.2677	0.9935	1.0238
HDL	−0.0070	0.9930	0.0097	0.4718	0.9743	1.0121
LDL	−0.0089	0.9911	0.0078	0.2511	0.9761	1.0063
triglycerides	0.0002	1.0002	0.0014	0.8612	0.9976	1.0029
diabetes	0.3152	1.3705	0.1890	0.0954	0.9462	1.9850
smoking	0.1971	1.2179	0.1407	0.1612	0.9244	1.6046
systolic bloodpressure	0.0150	1.0151	0.0070	0.0319	1.0013	1.0290
diastolic bloodpressure	−0.0152	0.9850	0.0153	0.3222	0.9558	1.0150
CRP	0.1730	1.1888	0.1551	0.2648	0.8772	1.6112
BMI	0.0026	1.0026	0.0491	0.9584	0.9105	1.1039
physical activity	0.5036	1.6547	0.2135	0.0183	1.0889	2.5146
coronary artery calcification	0.2896	1.3359	0.0505	< 0.0001	1.2100	1.4748

m2)

	coef	HR	se	p	lower	upper
rs755017	0.0594	1.0612	0.1194	0.6188	0.8398	1.3410
sex	−0.1064	0.8991	0.1400	0.4472	0.6833	1.1829
age	0.0629	1.0649	0.0092	< 0.0001	1.0459	1.0843
total cholesterol	−0.0047	0.9953	0.0042	0.266	0.9871	1.0036
HDL	−0.0048	0.9952	0.0057	0.4064	0.9841	1.0065
LDL	0.0008	1.0008	0.0043	0.8537	0.9924	1.0093
triglycerides	0.0013	1.0013	0.0007	0.0853	0.9998	1.0027
diabetes	0.1151	1.1219	0.0844	0.1728	0.9509	1.3238
smoking	0.1482	1.1598	0.0808	0.0666	0.9899	1.3588
systolic bloodpressure	0.0049	1.0050	0.0038	0.1934	0.9975	1.0125
diastolic bloodpressure	−0.0003	0.9997	0.0072	0.9673	0.9857	1.0140
CRP	−0.0363	0.9643	0.0841	0.6658	0.8177	1.1372
BMI	−0.0003	0.9997	0.0139	0.9811	0.9727	1.0274
physical activity	0.0342	1.0348	0.1108	0.7578	0.8327	1.2858
coronary artery calcification	0.2660	1.3048	0.0288	< 0.0001	1.2331	1.3806

n1)

	coef	HR	se	p	lower	upper
rs755017	0.2242	1.2513	0.2848	0.4312	0.7160	2.1866
sex	0.0095	1.0096	0.3372	0.9774	0.5213	1.9552
age	0.0635	1.0656	0.0194	0.0011	1.0258	1.1069
total cholesterol	0.0106	1.0106	0.0081	0.1915	0.9947	1.0268
HDL	−0.0159	0.9842	0.0104	0.1263	0.9644	1.0045
LDL	−0.0135	0.9866	0.0080	0.0919	0.9712	1.0022
triglycerides	−0.0006	0.9994	0.0015	0.6689	0.9964	1.0023
diabetes	0.2145	1.2392	0.2405	0.3725	0.7735	1.9854
smoking	0.2343	1.2640	0.1454	0.1071	0.9506	1.6809
systolic bloodpressure	0.0076	1.0076	0.0077	0.3262	0.9925	1.0229
diastolic bloodpressure	−0.0013	0.9987	0.0163	0.9381	0.9673	1.0312
CRP	−0.0194	0.9808	0.1202	0.872	0.7749	1.2414
physical activity	0.5697	1.7677	0.2350	0.0153	1.1152	2.8019
waist circumference	−0.0252	0.9751	0.0183	0.1675	0.9407	1.0107
coronary artery calcification	0.3796	1.4617	0.0605	< 0.0001	1.2983	1.6456

n2)

	coef	HR	se	p	lower	upper
rs755017	−0.0267	0.9737	0.1123	0.8123	0.7813	1.2135
sex	−0.1651	0.8478	0.1405	0.24	0.6436	1.1166
age	0.0585	1.0602	0.0089	< 0.0001	1.0419	1.0789
total cholesterol	−0.0054	0.9947	0.0041	0.1934	0.9866	1.0027
HDL	−0.0018	0.9982	0.0056	0.7452	0.9874	1.0091
LDL	0.0019	1.0019	0.0042	0.6452	0.9937	1.0102
triglycerides	0.0015	1.0015	0.0007	0.0334	1.0001	1.0029
diabetes	0.1304	1.1393	0.0828	0.1153	0.9686	1.3400
smoking	0.1659	1.1804	0.0788	0.0352	1.0116	1.3774
systolic bloodpressure	0.0061	1.0062	0.0038	0.102	0.9988	1.0136
diastolic bloodpressure	−0.0057	0.9943	0.0072	0.4219	0.9804	1.0083
CRP	0.0044	1.0044	0.0851	0.959	0.8501	1.1866
physical activity	0.0444	1.0455	0.1088	0.6829	0.8447	1.2939
waist circumference	−0.0042	0.9958	0.0057	0.4565	0.9847	1.0069
coronary artery calcification	0.2510	1.2853	0.0275	< 0.0001	1.2180	1.3564

S4J Table. Results of Cox regression models for rsgenetic risk score.

coef: coefficient, HR: Hazard Ratio, se: Standard error,
lower/upper: lower/upper boundarie of the 95% confidence interval
a) crude, b) adjusted, c1) young age, c2) older age, d1) male, d2) female,
e1) low hsCRP, e2) intermediate hsCRP, e3) high hsCRP, f1) low total cholesterol,
f2) high total cholesterol, g1) low LDL, g2) high LDL, h1) normal HDL, h2) high HDL,
i1) low triglycerides, i2) high triglycerides, j1) ideal blood pressure,
j2) normal/high normal blood pressure, j3) hypertension, k1) no diabetes, k2) diabetes,
l1) never smoker, l2) former smoker, l3) current smoker, m1) normal waist circumference,
m2) high waist circumference, n1) normal BMI, n2) high BMI

a)

	coef	HR	se	p	lower	upper
genetic risk score	−0.0283	0.972	0.0464	0.5412	0.8876	1.0646

b)

	coef	HR	se	p	lower	upper
genetic risk score	0.0139	1.0140	0.0478	0.7707	0.9233	1.1137
sex	−0.1105	0.8954	0.1489	0.4581	0.6688	1.1988
age	0.0593	1.0611	0.0081	< 0.0001	1.0444	1.0781
total cholesterol	−0.0022	0.9978	0.0038	0.5525	0.9904	1.0052
HDL	−0.0044	0.9956	0.0050	0.3753	0.9860	1.0053
LDL	−0.0010	0.9990	0.0038	0.7999	0.9915	1.0066
triglycerides	0.0011	1.0011	0.0006	0.0886	0.9998	1.0024
diabetes	0.1553	1.1681	0.0799	0.0519	0.9987	1.3661
smoking	0.1820	1.1997	0.0695	0.0088	1.0468	1.3748
systolic bloodpressure	0.0071	1.0071	0.0034	0.036	1.0005	1.0138
diastolic bloodpressure	−0.0050	0.9950	0.0065	0.4482	0.9824	1.0079
CRP	0.0008	1.0008	0.0701	0.9906	0.8723	1.1483
BMI	0.0044	1.0044	0.0221	0.8421	0.9618	1.0489
physical activity	0.1609	1.1745	0.0983	0.1018	0.9686	1.4241
waist circumference	−0.0083	0.9918	0.0087	0.3431	0.9750	1.0089
coronary artery calcification	0.2753	1.3169	0.0250	< 0.0001	1.2541	1.3830

c1)

	coef	HR	se	p	lower	upper
genetic risk score	0.1753	1.1916	0.1021	0.0858	0.9756	1.4555
sex	−0.0990	0.9057	0.2861	0.7293	0.5170	1.5869
total cholesterol	0.0030	1.0030	0.0067	0.6538	0.9899	1.0164
HDL	−0.0157	0.9844	0.0102	0.122	0.9650	1.0042
LDL	−0.0028	0.9972	0.0069	0.6858	0.9839	1.0107
triglycerides	0.0002	1.0002	0.0013	0.8689	0.9976	1.0028
diabetes	−0.0026	0.9974	0.1979	0.9894	0.6767	1.4700
smoking	0.3073	1.3598	0.1347	0.0225	1.0443	1.7706
systolic bloodpressure	−0.0032	0.9968	0.0090	0.7177	0.9794	1.0144
diastolic bloodpressure	0.0176	1.0178	0.0162	0.2769	0.9860	1.0506
CRP	0.1195	1.1269	0.1662	0.4721	0.8137	1.5608
BMI	0.0072	1.0072	0.0465	0.8772	0.9194	1.1034
physical activity	0.1977	1.2186	0.2140	0.3556	0.8011	1.8538
waist circumference	−0.0144	0.9857	0.0182	0.4291	0.9512	1.0215
coronary artery calcification	0.2920	1.3391	0.0462	< 0.0001	1.2231	1.4661

c2)

	coef	HR	se	p	lower	upper
genetic risk score	−0.0364	0.9643	0.0541	0.5013	0.8673	1.0721
sex	0.3361	1.3995	0.1748	0.0546	0.9934	1.9714
total cholesterol	−0.0044	0.9956	0.0042	0.2972	0.9875	1.0039
HDL	−0.0004	0.9996	0.0046	0.9391	0.9907	1.0087
LDL	0.0000	1.0000	0.0043	0.9947	0.9915	1.0085
triglycerides	0.0011	1.0011	0.0007	0.1017	0.9998	1.0025
diabetes	0.1831	1.2010	0.0885	0.0384	1.0098	1.4284
smoking	0.0793	1.0825	0.0819	0.3331	0.9220	1.2709
systolic bloodpressure	0.0137	1.0138	0.0036	0.0001	1.0067	1.0210
diastolic bloodpressure	−0.0181	0.9820	0.0070	0.0101	0.9686	0.9957
CRP	−0.0048	0.9952	0.0737	0.9479	0.8613	1.1499
BMI	−0.0102	0.9898	0.0257	0.6917	0.9411	1.0411
physical activity	0.1385	1.1486	0.1112	0.2128	0.9236	1.4283
waist circumference	−0.0014	0.9986	0.0100	0.8878	0.9792	1.0183
coronary artery calcification	0.2883	1.3341	0.0294	< 0.0001	1.2594	1.4132

d1)

	coef	HR	se	p	lower	upper
genetic risk score	−0.0155	0.9846	0.0585	0.7912	0.8780	1.1043
age	0.0433	1.0443	0.0096	< 0.0001	1.0248	1.0642
total cholesterol	−0.0059	0.9942	0.0048	0.2234	0.9848	1.0036
HDL	0.0019	1.0019	0.0061	0.7544	0.9899	1.0141
LDL	0.0027	1.0027	0.0049	0.587	0.9931	1.0123
triglycerides	0.0015	1.0015	0.0008	0.0556	1.0000	1.0030
diabetes	0.1708	1.1863	0.0910	0.0606	0.9924	1.4180
smoking	0.1079	1.1140	0.0891	0.2259	0.9354	1.3266
systolic bloodpressure	0.0048	1.0048	0.0043	0.2598	0.9965	1.0132
diastolic bloodpressure	−0.0025	0.9975	0.0080	0.7491	0.9820	1.0131
CRP	0.0034	1.0034	0.0786	0.9655	0.8601	1.1706
BMI	0.0347	1.0353	0.0291	0.2331	0.9779	1.0960
physical activity	0.1644	1.1787	0.1208	0.1736	0.9302	1.4938
waist circumference	−0.0127	0.9874	0.0112	0.2583	0.9659	1.0094
coronary artery calcification	0.2971	1.3460	0.0333	< 0.0001	1.2611	1.4366

d2)

	coef	HR	se	p	lower	upper
genetic risk score	0.0805	1.0838	0.0829	0.3321	0.9212	1.2751
age	0.0986	1.1036	0.0152	< 0.0001	1.0712	1.1370
total cholesterol	0.0017	1.0017	0.0055	0.7523	0.9911	1.0125
HDL	−0.0140	0.9861	0.0081	0.083	0.9707	1.0018
LDL	−0.0058	0.9942	0.0056	0.2971	0.9834	1.0051
triglycerides	0.0012	1.0012	0.0013	0.3689	0.9986	1.0038
diabetes	0.1052	1.1109	0.1725	0.5418	0.7923	1.5577
smoking	0.3317	1.3933	0.1098	0.0025	1.1237	1.7277
systolic bloodpressure	0.0097	1.0098	0.0056	0.084	0.9987	1.0210
diastolic bloodpressure	−0.0065	0.9936	0.0117	0.5802	0.9711	1.0166
CRP	0.0146	1.0148	0.1477	0.921	0.7597	1.3554
BMI	−0.0285	0.9719	0.0348	0.4131	0.9077	1.0406
physical activity	0.1469	1.1582	0.1708	0.3899	0.8287	1.6188
waist circumference	−0.0067	0.9934	0.0141	0.6359	0.9664	1.0211
coronary artery calcification	0.2372	1.2677	0.0386	< 0.0001	1.1752	1.3674

e1)

	coef	HR	se	p	lower	upper
genetic risk score	−0.0061	0.9939	0.0579	0.9155	0.8873	1.1132
sex	−0.1753	0.8392	0.1816	0.3342	0.5879	1.1978
age	0.0683	1.0707	0.0098	< 0.0001	1.0503	1.0915
total cholesterol	0.0001	1.0001	0.0043	0.9847	0.9917	1.0086
HDL	−0.0063	0.9937	0.0058	0.2744	0.9825	1.0050
LDL	−0.0039	0.9962	0.0044	0.3846	0.9875	1.0048
triglycerides	0.0011	1.0011	0.0007	0.1491	0.9996	1.0025
diabetes	0.1308	1.1397	0.1012	0.1965	0.9346	1.3898
smoking	0.1753	1.1916	0.0860	0.0416	1.0067	1.4105
systolic bloodpressure	0.0077	1.0077	0.0043	0.0703	0.9994	1.0162
diastolic bloodpressure	−0.0043	0.9958	0.0083	0.6078	0.9797	1.0121
BMI	0.0382	1.0389	0.0280	0.1728	0.9834	1.0976
physical activity	0.1794	1.1965	0.1183	0.1294	0.9489	1.5087
waist circumference	−0.0165	0.9836	0.0105	0.1151	0.9636	1.0040
coronary artery calcification	0.2711	1.3114	0.0301	< 0.0001	1.2364	1.3910

e2)

	coef	HR	se	p	lower	upper
genetic risk score	0.2552	1.2907	0.1488	0.0863	0.9643	1.7276
sex	0.732	2.0792	0.4524	0.1057	0.8567	5.0467
age	0.1001	1.1053	0.0251	0.0001	1.0523	1.161
total cholesterol	−0.0056	0.9944	0.0131	0.6663	0.9692	1.0202
HDL	−0.0127	0.9874	0.0171	0.4564	0.9549	1.0209
LDL	0.0049	1.0049	0.0125	0.697	0.9806	1.0298
triglycerides	−0.0036	0.9964	0.0026	0.1671	0.9913	1.0015
diabetes	0.3614	1.4354	0.1919	0.0596	0.9855	2.0906
smoking	0.1968	1.2175	0.2101	0.3488	0.8066	1.8378
systolic bloodpressure	0.0006	1.0006	0.0095	0.948	0.9821	1.0195
diastolic bloodpressure	0.0038	1.0038	0.0174	0.8288	0.9702	1.0385
BMI	−0.1454	0.8647	0.0736	0.0483	0.7486	0.9989
physical activity	−0.592	0.5532	0.2808	0.035	0.3191	0.9591
waist circumference	0.0688	1.0713	0.0298	0.0208	1.0105	1.1356
coronary artery calcification	0.2823	1.3262	0.0735	0.0001	1.1482	1.5318

e3)

	coef	HR	se	p	lower	upper
genetic risk score	0.0379	1.0386	0.1118	0.7347	0.8342	1.2932
sex	−0.3168	0.7285	0.3536	0.3703	0.3643	1.4567
age	0.0077	1.0077	0.0185	0.6775	0.9718	1.0450
total cholesterol	−0.0047	0.9953	0.0065	0.4698	0.9826	1.0081
HDL	0.0007	1.0007	0.0036	0.8353	0.9938	1.0077
LDL	0.0031	1.0031	0.0068	0.6508	0.9898	1.0166
triglycerides	0.0022	1.0022	0.0012	0.0757	0.9998	1.0047
diabetes	0.1671	1.1819	0.1743	0.3376	0.8399	1.6631
smoking	0.1309	1.1399	0.1602	0.4137	0.8328	1.5602
systolic bloodpressure	0.0066	1.0066	0.0073	0.3631	0.9924	1.0211
diastolic bloodpressure	−0.0190	0.9811	0.0149	0.2018	0.9529	1.0102
BMI	−0.0320	0.9685	0.0484	0.509	0.8809	1.0649
physical activity	0.4886	1.6300	0.2424	0.0438	1.0137	2.6212
waist circumference	−0.0175	0.9827	0.0203	0.3889	0.9444	1.0225
coronary artery calcification	0.2937	1.3413	0.0588	< 0.0001	1.1954	1.5051

f1)

	coef	HR	se	p	lower	upper
genetic risk score	0.1097	1.1159	0.0896	0.2208	0.9362	1.3301
sex	0.4195	1.5212	0.2705	0.121	0.8951	2.5850
age	0.0727	1.0754	0.0161	< 0.0001	1.0421	1.1098
HDL	−0.0080	0.9920	0.0086	0.3514	0.9753	1.0089
LDL	−0.0049	0.9951	0.0048	0.3017	0.9858	1.0044
triglycerides	0.0005	1.0005	0.0013	0.7342	0.9978	1.0031
diabetes	0.2043	1.2267	0.1515	0.1773	0.9116	1.6507
smoking	0.0058	1.0058	0.1334	0.9656	0.7744	1.3063
systolic bloodpressure	0.0035	1.0035	0.0067	0.604	0.9904	1.0167
diastolic bloodpressure	−0.0025	0.9975	0.0131	0.8457	0.9722	1.0233
CRP	−0.0420	0.9589	0.1222	0.7313	0.7547	1.2183
BMI	−0.1049	0.9004	0.0432	0.0151	0.8274	0.9799
physical activity	0.2667	1.3057	0.1920	0.1648	0.8962	1.9022
waist circumference	0.0294	1.0299	0.0160	0.0651	0.9982	1.0626
coronary artery calcification	0.2995	1.3491	0.0479	< 0.0001	1.2283	1.4819

f2)

	coef	HR	se	p	lower	upper
genetic risk score	−0.0368	0.9638	0.0572	0.5196	0.8616	1.0782
sex	−0.3135	0.7309	0.1776	0.0776	0.5160	1.0353
age	0.0556	1.0572	0.0094	< 0.0001	1.0378	1.0769
HDL	−0.0058	0.9942	0.0044	0.1841	0.9856	1.0028
LDL	−0.0029	0.9971	0.0019	0.1298	0.9933	1.0009
triglycerides	0.0007	1.0007	0.0005	0.1405	0.9998	1.0017
diabetes	0.1229	1.1307	0.0955	0.1984	0.9376	1.3636
smoking	0.2641	1.3023	0.0826	0.0014	1.1075	1.5313
systolic bloodpressure	0.0078	1.0079	0.0040	0.049	1.0000	1.0157
diastolic bloodpressure	−0.0064	0.9936	0.0076	0.3984	0.9788	1.0086
CRP	0.0149	1.0150	0.0851	0.8611	0.8591	1.1992
BMI	0.0485	1.0497	0.0256	0.0581	0.9983	1.1037
physical activity	0.1203	1.1278	0.1166	0.3022	0.8974	1.4173
waist circumference	−0.0225	0.9777	0.0102	0.0271	0.9584	0.9975
coronary artery calcification	0.2663	1.3051	0.0297	< 0.0001	1.2313	1.3833

g1)

	coef	HR	se	p	lower	upper
genetic risk score	0.0315	1.0320	0.1033	0.7603	0.8429	1.2636
sex	0.1718	1.1875	0.2943	0.5593	0.6670	2.1141
age	0.0818	1.0853	0.0176	< 0.0001	1.0485	1.1233
total cholesterol	−0.0020	0.9980	0.0046	0.67	0.9890	1.0072
HDL	−0.0014	0.9986	0.0074	0.8519	0.9842	1.0132
triglycerides	0.0017	1.0017	0.0007	0.015	1.0003	1.0032
diabetes	0.2089	1.2323	0.1678	0.2133	0.8869	1.7122
smoking	0.1887	1.2077	0.1439	0.1896	0.9110	1.6011
systolic bloodpressure	0.0014	1.0014	0.0073	0.8483	0.9872	1.0158
diastolic bloodpressure	−0.0058	0.9943	0.0139	0.6786	0.9676	1.0217
CRP	−0.2694	0.7638	0.2297	0.2408	0.4869	1.1981
BMI	−0.0754	0.9274	0.0458	0.1	0.8478	1.0145
physical activity	0.4903	1.6327	0.2102	0.0197	1.0814	2.4652
waist circumference	0.0218	1.0220	0.0172	0.2068	0.9880	1.0571
coronary artery calcification	0.2682	1.3076	0.0515	< 0.0001	1.1819	1.4466

g2)

	coef	HR	se	p	lower	upper
genetic risk score	−0.0012	0.9988	0.0546	0.9831	0.8975	1.1116
sex	−0.1874	0.8291	0.1720	0.2759	0.5919	1.1614
age	0.0543	1.0558	0.0092	< 0.0001	1.0369	1.0751
total cholesterol	−0.0032	0.9968	0.0019	0.0827	0.9931	1.0004
HDL	−0.0040	0.9960	0.0047	0.3918	0.9870	1.0052
triglycerides	0.0010	1.0010	0.0007	0.1705	0.9996	1.0023
diabetes	0.1330	1.1423	0.0922	0.149	0.9535	1.3684
smoking	0.1998	1.2211	0.0801	0.0126	1.0437	1.4286
systolic bloodpressure	0.0078	1.0078	0.0039	0.044	1.0002	1.0155
diastolic bloodpressure	−0.0042	0.9958	0.0075	0.5747	0.9813	1.0105
CRP	0.0890	1.0930	0.0847	0.2937	0.9258	1.2905
BMI	0.0256	1.0259	0.0250	0.3061	0.9768	1.0775
physical activity	0.0807	1.0840	0.1127	0.474	0.8692	1.3519
waist circumference	−0.0168	0.9833	0.0099	0.0898	0.9644	1.0026
coronary artery calcification	0.2765	1.3185	0.0290	< 0.0001	1.2457	1.3956

h1)

	coef	HR	se	p	lower	upper
genetic risk score	0.0310	1.0315	0.0569	0.585	0.9228	1.1531
sex	−0.1280	0.8799	0.1726	0.4583	0.6274	1.2340
age	0.0700	1.0725	0.0098	< 0.0001	1.0520	1.0933
total cholesterol	−0.0019	0.9981	0.0035	0.5764	0.9913	1.0049
LDL	−0.0005	0.9995	0.0036	0.8987	0.9926	1.0066
triglycerides	−0.0001	0.9999	0.0008	0.8928	0.9983	1.0015
diabetes	0.1717	1.1873	0.0981	0.0801	0.9796	1.4391
smoking	0.2379	1.2686	0.0826	0.004	1.0789	1.4916
systolic bloodpressure	0.0095	1.0096	0.0039	0.0142	1.0019	1.0173
diastolic bloodpressure	−0.0042	0.9958	0.0076	0.585	0.9811	1.0108
CRP	−0.0799	0.9232	0.0993	0.4213	0.7599	1.1216
BMI	0.0195	1.0197	0.0260	0.454	0.9690	1.0729
physical activity	0.1814	1.1989	0.1147	0.1137	0.9575	1.5011
waist circumference	−0.0070	0.9930	0.0104	0.4989	0.9730	1.0134
coronary artery calcification	0.2547	1.2901	0.0285	< 0.0001	1.2201	1.3641

h2)

	coef	HR	se	p	lower	upper
genetic risk score	−0.0456	0.9554	0.0917	0.6187	0.7983	1.1435
sex	−0.2859	0.7514	0.2827	0.3119	0.4318	1.3076
age	0.0344	1.0350	0.0147	0.019	1.0057	1.0652
total cholesterol	−0.0047	0.9953	0.0058	0.4201	0.9840	1.0068
LDL	0.0008	1.0009	0.0063	0.8927	0.9886	1.0133
triglycerides	0.0021	1.0021	0.0008	0.011	1.0005	1.0037
diabetes	0.0707	1.0732	0.1414	0.617	0.8135	1.4159
smoking	0.0367	1.0374	0.1343	0.7846	0.7973	1.3497
systolic bloodpressure	0.0007	1.0007	0.0069	0.9157	0.9873	1.0144
diastolic bloodpressure	−0.0066	0.9934	0.0133	0.6171	0.9679	1.0196
CRP	0.1744	1.1906	0.1162	0.1333	0.9481	1.4951
BMI	−0.0324	0.9681	0.0427	0.4484	0.8904	1.0527
physical activity	0.1177	1.1249	0.1918	0.5395	0.7724	1.6384
waist circumference	−0.0163	0.9839	0.0162	0.316	0.9531	1.0156
coronary artery calcification	0.3314	1.3929	0.0525	< 0.0001	1.2567	1.5439

i1)

	coef	HR	se	p	lower	upper
genetic risk score	−0.0249	0.9754	0.0651	0.7019	0.8585	1.1082
sex	−0.0997	0.9051	0.2008	0.6194	0.6107	1.3415
age	0.0732	1.0760	0.0113	< 0.0001	1.0523	1.1001
total cholesterol	−0.0014	0.9986	0.0052	0.7941	0.9884	1.0089
HDL	−0.0024	0.9976	0.0064	0.7098	0.9852	1.0102
LDL	−0.0028	0.9972	0.0054	0.6033	0.9867	1.0078
diabetes	0.0401	1.0409	0.1257	0.7499	0.8135	1.3318
smoking	0.3054	1.3572	0.0942	0.0012	1.1283	1.6326
systolic bloodpressure	0.0045	1.0045	0.0046	0.3321	0.9954	1.0137
diastolic bloodpressure	0.0128	1.0128	0.0091	0.1596	0.9950	1.0310
CRP	−0.0526	0.9487	0.0893	0.5558	0.7963	1.1303
BMI	0.0288	1.0292	0.0312	0.356	0.9682	1.0940
physical activity	0.3479	1.4161	0.1336	0.0092	1.0899	1.8400
waist circumference	−0.0151	0.9850	0.0119	0.2043	0.9623	1.0083
coronary artery calcification	0.2779	1.3204	0.0330	< 0.0001	1.2378	1.4086

i2)

	coef	HR	se	p	lower	upper
genetic risk score	0.0463	1.0474	0.0722	0.5217	0.9091	1.2066
sex	−0.1156	0.8908	0.2258	0.6087	0.5722	1.3869
age	0.0443	1.0453	0.0116	0.0001	1.0218	1.0694
total cholesterol	0.0019	1.0019	0.0034	0.5807	0.9953	1.0085
HDL	−0.0176	0.9826	0.0070	0.0124	0.9692	0.9962
LDL	−0.0038	0.9962	0.0035	0.2836	0.9894	1.0031
diabetes	0.2512	1.2856	0.1062	0.018	1.0441	1.5829
smoking	0.0571	1.0587	0.1052	0.5874	0.8615	1.3012
systolic bloodpressure	0.0100	1.0100	0.0049	0.0411	1.0004	1.0198
diastolic bloodpressure	−0.0245	0.9758	0.0097	0.0117	0.9573	0.9945
CRP	0.1157	1.1226	0.1348	0.3907	0.8620	1.4620
BMI	−0.0236	0.9766	0.0325	0.4669	0.9164	1.0408
physical activity	−0.1219	0.8852	0.1458	0.403	0.6652	1.1780
waist circumference	0.0005	1.0005	0.0129	0.9676	0.9756	1.0261
coronary artery calcification	0.2672	1.3063	0.0386	< 0.0001	1.2112	1.4090

j1)

	coef	HR	se	p	lower	upper
genetic risk score	0.0917	1.0961	0.1230	0.4557	0.8613	1.3949
sex	−0.1095	0.8963	0.3632	0.7632	0.4398	1.8266
age	0.0568	1.0584	0.0187	0.0024	1.0204	1.0979
total cholesterol	0.0146	1.0147	0.0107	0.1748	0.9935	1.0363
HDL	−0.0249	0.9754	0.0132	0.0591	0.9505	1.0010
LDL	−0.0148	0.9853	0.0103	0.1491	0.9656	1.0053
triglycerides	0.0023	1.0023	0.0024	0.3373	0.9976	1.0072
diabetes	−0.0781	0.9249	0.2273	0.7311	0.5923	1.4441
smoking	0.2166	1.2418	0.1760	0.2185	0.8795	1.7533
CRP	0.1268	1.1352	0.1471	0.3889	0.8508	1.5146
BMI	0.0087	1.0087	0.0594	0.8837	0.8979	1.1332
physical activity	0.2590	1.2957	0.2636	0.3258	0.7729	2.1720
waist circumference	−0.0153	0.9849	0.0236	0.5186	0.9403	1.0315
coronary artery calcification	0.3625	1.4369	0.0615	< 0.0001	1.2737	1.6209

j2)

	coef	HR	se	p	lower	upper
genetic risk score	−0.0201	0.9801	0.0813	0.8048	0.8357	1.1495
sex	−0.5828	0.5583	0.2641	0.0273	0.3327	0.9369
age	0.0711	1.0737	0.0125	< 0.0001	1.0476	1.1004
total cholesterol	0.0013	1.0013	0.0055	0.8101	0.9907	1.0121
HDL	−0.0095	0.9906	0.0083	0.2547	0.9745	1.0069
LDL	−0.0036	0.9964	0.0056	0.5114	0.9856	1.0073
triglycerides	0.0004	1.0004	0.0012	0.7245	0.9981	1.0028
diabetes	0.2607	1.2978	0.1316	0.0476	1.0028	1.6796
smoking	0.2399	1.2711	0.1172	0.0406	1.0103	1.5992
CRP	0.0662	1.0684	0.1634	0.6855	0.7756	1.4717
BMI	0.0662	1.0684	0.0370	0.0738	0.9936	1.1489
physical activity	0.2193	1.2452	0.1642	0.1817	0.9025	1.7179
waist circumference	−0.0351	0.9655	0.0147	0.017	0.9381	0.9937
coronary artery calcification	0.2126	1.2369	0.0394	< 0.0001	1.1450	1.3362

j3)

	coef	HR	se	p	lower	upper
genetic risk score	0.0124	1.0125	0.0678	0.855	0.8864	1.1564
sex	0.1520	1.1641	0.2065	0.4617	0.7767	1.7448
age	0.0614	1.0634	0.0115	< 0.0001	1.0397	1.0876
total cholesterol	−0.0074	0.9927	0.0044	0.0954	0.9841	1.0013
HDL	0.0019	1.0019	0.0022	0.3686	0.9977	1.0062
LDL	0.0026	1.0026	0.0047	0.5852	0.9934	1.0118
triglycerides	0.0014	1.0014	0.0007	0.0343	1.0001	1.0028
diabetes	0.1422	1.1528	0.1133	0.2095	0.9232	1.4394
smoking	0.1624	1.1763	0.1023	0.1124	0.9626	1.4374
CRP	−0.0508	0.9504	0.0961	0.597	0.7873	1.1475
BMI	−0.0351	0.9656	0.0320	0.2737	0.9068	1.0281
physical activity	0.0614	1.0633	0.1409	0.663	0.8067	1.4017
waist circumference	0.0077	1.0077	0.0124	0.5339	0.9836	1.0325
coronary artery calcification	0.2974	1.3463	0.0379	< 0.0001	1.2499	1.4502

k1)

	coef	HR	se	p	lower	upper
genetic risk score	−0.0426	0.9583	0.0569	0.4542	0.8572	1.0714
sex	−0.1177	0.8890	0.1712	0.4919	0.6356	1.2434
age	0.0651	1.0673	0.0094	< 0.0001	1.0479	1.0871
total cholesterol	0.0000	1.0000	0.0042	0.9926	0.9917	1.0083
HDL	−0.0071	0.9929	0.0058	0.2216	0.9816	1.0043
LDL	−0.0022	0.9978	0.0043	0.6118	0.9894	1.0063
triglycerides	0.0002	1.0002	0.0009	0.8518	0.9984	1.0019
smoking	0.2922	1.3394	0.0794	0.0002	1.1464	1.5648
systolic bloodpressure	0.0039	1.0040	0.0042	0.3441	0.9958	1.0122
diastolic bloodpressure	0.0035	1.0035	0.0080	0.6617	0.9879	1.0194
CRP	−0.1100	0.8958	0.1142	0.3353	0.7163	1.1205
BMI	0.0248	1.0251	0.0267	0.3527	0.9729	1.0801
physical activity	0.2030	1.2250	0.1138	0.0744	0.9802	1.5311
waist circumference	−0.0093	0.9907	0.0102	0.3608	0.9711	1.0107
coronary artery calcification	0.2622	1.2998	0.0283	< 0.0001	1.2297	1.3739

k2)

	coef	HR	se	p	lower	upper
genetic risk score	0.1271	1.1356	0.0881	0.149	0.9555	1.3495
sex	−0.3752	0.6872	0.3207	0.242	0.3665	1.2884
age	0.0472	1.0483	0.0167	0.0048	1.0145	1.0833
total cholesterol	−0.0050	0.9950	0.0092	0.5822	0.9773	1.0130
HDL	0.0048	1.0048	0.0102	0.6411	0.9849	1.0250
LDL	0.0010	1.0010	0.0093	0.9143	0.9830	1.0193
triglycerides	0.0021	1.0021	0.0011	0.0713	0.9998	1.0043
smoking	−0.0899	0.9140	0.1555	0.5632	0.6739	1.2397
systolic bloodpressure	0.0114	1.0115	0.0061	0.0592	0.9996	1.0236
diastolic bloodpressure	−0.0191	0.9811	0.0117	0.1027	0.9588	1.0039
CRP	0.1096	1.1158	0.0823	0.1831	0.9496	1.3111
BMI	−0.0169	0.9833	0.0437	0.6996	0.9025	1.0713
physical activity	−0.0832	0.9202	0.2018	0.6802	0.6195	1.3667
waist circumference	−0.0158	0.9843	0.0175	0.3651	0.9512	1.0186
coronary artery calcification	0.3122	1.3664	0.0567	< 0.0001	1.2228	1.5269

I1)

	coef	HR	se	p	lower	upper
genetic risk score	−0.0152	0.9849	0.0803	0.8502	0.8415	1.1529
sex	−0.1587	0.8533	0.2338	0.4973	0.5396	1.3493
age	0.0876	1.0916	0.0154	< 0.0001	1.0590	1.1251
total cholesterol	−0.0038	0.9962	0.0067	0.5778	0.9832	1.0095
HDL	−0.0099	0.9901	0.0090	0.2721	0.9728	1.0078
LDL	−0.0016	0.9984	0.0068	0.8164	0.9852	1.0118
triglycerides	0.0014	1.0014	0.0016	0.363	0.9984	1.0045
diabetes	0.3778	1.4590	0.1408	0.0073	1.1072	1.9227
systolic bloodpressure	0.0089	1.0089	0.0060	0.1384	0.9971	1.0208
diastolic bloodpressure	−0.0066	0.9935	0.0119	0.5801	0.9706	1.0168
CRP	0.0149	1.0150	0.1090	0.8913	0.8198	1.2567
BMI	−0.0084	0.9916	0.0347	0.8084	0.9264	1.0615
physical activity	0.0218	1.0220	0.1705	0.8983	0.7317	1.4274
waist circumference	−0.0031	0.9969	0.0140	0.8252	0.9700	1.0246
coronary artery calcification	0.2533	1.2883	0.0408	< 0.0001	1.1893	1.3956

l2)

	coef	HR	se	p	lower	upper
genetic risk score	−0.1226	0.8847	0.0773	0.1129	0.7603	1.0294
sex	−0.4601	0.6312	0.2805	0.101	0.3642	1.0939
age	0.0548	1.0563	0.0126	< 0.0001	1.0305	1.0828
total cholesterol	−0.0013	0.9987	0.0065	0.8437	0.9862	1.0114
HDL	0.0045	1.0045	0.0080	0.5713	0.9890	1.0203
LDL	−0.0011	0.9989	0.0065	0.8598	0.9862	1.0117
triglycerides	0.0013	1.0013	0.0010	0.2073	0.9993	1.0034
diabetes	0.0855	1.0893	0.1203	0.4774	0.8604	1.3790
systolic bloodpressure	0.0061	1.0061	0.0051	0.2292	0.9962	1.0162
diastolic bloodpressure	−0.0088	0.9913	0.0098	0.3713	0.9724	1.0105
CRP	−0.0354	0.9652	0.1573	0.822	0.7091	1.3138
BMI	0.0579	1.0597	0.0401	0.149	0.9795	1.1464
physical activity	0.2374	1.2679	0.1539	0.123	0.9377	1.7144
waist circumference	−0.0201	0.9801	0.0149	0.1752	0.9520	1.0090
coronary artery calcification	0.2735	1.3146	0.0403	< 0.0001	1.2148	1.4226

I3)

	coef	HR	se	p	lower	upper
genetic risk score	0.3155	1.3709	0.1054	0.0028	1.1151	1.6853
sex	0.2298	1.2584	0.2899	0.4279	0.7129	2.2210
age	0.0365	1.0372	0.0161	0.0231	1.0050	1.0704
total cholesterol	0.0003	1.0003	0.0065	0.9664	0.9876	1.0131
HDL	−0.0107	0.9894	0.0100	0.2861	0.9701	1.0090
LDL	−0.0023	0.9977	0.0067	0.733	0.9846	1.0110
triglycerides	0.0005	1.0005	0.0011	0.6472	0.9984	1.0026
diabetes	0.0226	1.0229	0.1751	0.8971	0.7257	1.4417
systolic bloodpressure	0.0101	1.0101	0.0076	0.188	0.9951	1.0253
diastolic bloodpressure	−0.0055	0.9945	0.0140	0.693	0.9676	1.0222
CRP	−0.0209	0.9793	0.1122	0.852	0.7860	1.2201
BMI	−0.0413	0.9596	0.0445	0.354	0.8794	1.0471
physical activity	0.1072	1.1132	0.2051	0.6011	0.7447	1.6638
waist circumference	0.0068	1.0069	0.0180	0.7038	0.9720	1.0429
coronary artery calcification	0.3140	1.3689	0.0516	< 0.0001	1.2373	1.5145

m1)

	coef	HR	se	p	lower	upper
genetic risk score	0.1797	1.1968	0.1007	0.0745	0.9824	1.4580
sex	0.1682	1.1832	0.2814	0.5499	0.6817	2.0538
age	0.0411	1.0419	0.0167	0.0142	1.0083	1.0767
total cholesterol	0.0090	1.0091	0.0076	0.2373	0.9941	1.0243
HDL	−0.0078	0.9922	0.0096	0.4173	0.9737	1.0111
LDL	−0.0097	0.9903	0.0077	0.2066	0.9755	1.0054
triglycerides	0.0001	1.0001	0.0013	0.9565	0.9975	1.0027
diabetes	0.3077	1.3603	0.1880	0.1017	0.9411	1.9663
smoking	0.2051	1.2276	0.1394	0.1414	0.9341	1.6135
systolic bloodpressure	0.0162	1.0163	0.0070	0.0202	1.0025	1.0303
diastolic bloodpressure	−0.0180	0.9822	0.0156	0.2498	0.9525	1.0127
CRP	0.1948	1.2150	0.1537	0.205	0.8990	1.6420
BMI	0.0020	1.0020	0.0480	0.967	0.9121	1.1007
physical activity	0.4956	1.6414	0.2116	0.0192	1.0842	2.4852
coronary artery calcification	0.3004	1.3504	0.0510	< 0.0001	1.2219	1.4925

m2)

	coef	HR	se	p	lower	upper
genetic risk score	−0.0108	0.9893	0.0538	0.8411	0.8903	1.0992
sex	−0.1118	0.8942	0.1396	0.423	0.6802	1.1755
age	0.0630	1.0650	0.0092	< 0.0001	1.0460	1.0844
total cholesterol	−0.0048	0.9953	0.0042	0.263	0.9870	1.0036
HDL	−0.0046	0.9954	0.0057	0.4269	0.9843	1.0067
LDL	0.0009	1.0009	0.0043	0.8438	0.9924	1.0094
triglycerides	0.0013	1.0013	0.0007	0.0869	0.9998	1.0027
diabetes	0.1143	1.1211	0.0844	0.1756	0.9502	1.3227
smoking	0.1479	1.1594	0.0808	0.0673	0.9895	1.3583
systolic bloodpressure	0.0049	1.0049	0.0038	0.2008	0.9974	1.0124
diastolic bloodpressure	−0.0003	0.9997	0.0072	0.9666	0.9857	1.0139
CRP	−0.0354	0.9652	0.0842	0.6741	0.8185	1.1383
BMI	−0.0004	0.9996	0.0140	0.9798	0.9727	1.0274
physical activity	0.0352	1.0358	0.1108	0.751	0.8336	1.2870
coronary artery calcification	0.2660	1.3048	0.0288	< 0.0001	1.2332	1.3806

n1)

	coef	HR	se	p	lower	upper
genetic risk score	0.1081	1.1141	0.1167	0.3544	0.8863	1.4005
sex	0.0158	1.0159	0.3370	0.9626	0.5248	1.9666
age	0.0629	1.0649	0.0194	0.0012	1.0251	1.1063
total cholesterol	0.0110	1.0111	0.0081	0.1719	0.9952	1.0273
HDL	−0.0161	0.9841	0.0103	0.1204	0.9643	1.0042
LDL	−0.0141	0.9860	0.0080	0.0797	0.9707	1.0017
triglycerides	−0.0006	0.9994	0.0015	0.6783	0.9965	1.0023
diabetes	0.2101	1.2338	0.2410	0.3834	0.7693	1.9787
smoking	0.2247	1.2519	0.1460	0.1239	0.9403	1.6668
systolic bloodpressure	0.0071	1.0071	0.0076	0.3529	0.9921	1.0223
diastolic bloodpressure	−0.0022	0.9978	0.0164	0.8948	0.9662	1.0304
CRP	−0.0164	0.9837	0.1172	0.8888	0.7818	1.2379
physical activity	0.5957	1.8144	0.2357	0.0115	1.1432	2.8795
waist circumference	−0.0240	0.9763	0.0182	0.1875	0.9420	1.0118
coronary artery calcification	0.3811	1.4640	0.0605	< 0.0001	1.3003	1.6482

n2)

	coef	HR	se	p	lower	upper
genetic risk score	−0.0024	0.9976	0.0523	0.9637	0.9004	1.1053
sex	−0.1635	0.8492	0.1405	0.2446	0.6448	1.1184
age	0.0584	1.0602	0.0089	< 0.0001	1.0418	1.0788
total cholesterol	−0.0053	0.9947	0.0041	0.1953	0.9867	1.0027
HDL	−0.0019	0.9981	0.0056	0.737	0.9873	1.0091
LDL	0.0019	1.0019	0.0042	0.6504	0.9937	1.0102
triglycerides	0.0015	1.0015	0.0007	0.0334	1.0001	1.0029
diabetes	0.1307	1.1396	0.0828	0.1146	0.9689	1.3404
smoking	0.1656	1.1801	0.0788	0.0355	1.0113	1.3771
systolic bloodpressure	0.0061	1.0062	0.0038	0.1017	0.9988	1.0136
diastolic bloodpressure	−0.0057	0.9943	0.0072	0.4226	0.9804	1.0083
CRP	0.0042	1.0042	0.0851	0.961	0.8500	1.1863
physical activity	0.0435	1.0445	0.1087	0.689	0.8440	1.2926
waist circumference	−0.0042	0.9958	0.0057	0.4601	0.9848	1.0070
coronary artery calcification	0.2511	1.2854	0.0275	< 0.0001	1.2180	1.3565
