# Supplementary material for: Genetic polymorphisms affecting telomere length and their association with cardiovascular disease in the Heinz-Nixdorf-Recall study
Source: PLoS One. 2024 May 14;19(5):e0303357. doi: 10.1371/journal.pone.0303357 (PMC11093374; doi:10.1371/journal.pone.0303357)
Supplement: S1 File — (DOCX) [file pone.0303357.s005.docx]

**S1 File. Formulas as used in R**

**SNPs**

1. All study participants

Crude

Time to first CVD-Event ~ SNP

Adjusted

Time to first CVD-Event ~ SNP + Age + Sex + total cholesterol + HDL cholesterol + LDL cholesterol + triglycerides + diabetes + smoking state + systolic blood pressure + diastolic blood pressure + CRP + BMI + physical activity (categorized) + waist circumference + coronary artery calcification

1. Female participants younger than 65 years and male participants younger than 55 years

Time to first CVD-Event ~ SNP + Sex + total cholesterol + HDL cholesterol + LDL cholesterol + triglycerides + diabetes + smoking state + systolic blood pressure + diastolic blood pressure + CRP + BMI + physical activity (categorized) + waist circumference + coronary artery calcification

1. Female participant older or equal 65 years and male participants older or equal 55 years

Time to first CVD-Event ~ SNP + Sex + total cholesterol + HDL cholesterol + LDL cholesterol + triglycerides + diabetes + smoking state + systolic blood pressure + diastolic blood pressure + CRP + BMI + physical activity (categorized) + waist circumference + coronary artery calcification

1. Male participants

Time to first CVD-Event ~ SNP + Age + total cholesterol + HDL cholesterol + LDL cholesterol + triglycerides + diabetes + smoking state + systolic blood pressure + diastolic blood pressure + CRP + BMI + physical activity (categorized) + waist circumference + coronary artery calcification

1. Female participants

Time to first CVD-Event ~ SNP + Age + total cholesterol + HDL cholesterol + LDL cholesterol + triglycerides + diabetes + smoking state + systolic blood pressure + diastolic blood pressure + CRP + BMI + physical activity (categorized) + waist circumference + coronary artery calcification

1. Participants with high sensitivity CRP ≤ 0.3 mg/dl

Time to first CVD-Event ~ SNP + Age + Sex + total cholesterol + HDL cholesterol + LDL cholesterol + triglycerides + diabetes + smoking state + systolic blood pressure + diastolic blood pressure + BMI + physical activity (categorized) + waist circumference + coronary artery calcification

1. Participants with high sensitivity CRP > 0.3 mg/dl but ≤ 0.5 mg/dl

Time to first CVD-Event ~ SNP + Age + Sex + total cholesterol + HDL cholesterol + LDL cholesterol + triglycerides + diabetes + smoking state + systolic blood pressure + diastolic blood pressure + BMI + physical activity (categorized) + waist circumference + coronary artery calcification

1. Participants with high sensitivity CRP > 0.5 mg/dl

Time to first CVD-Event ~ SNP + Age + Sex + total cholesterol + HDL cholesterol + LDL cholesterol + triglycerides + diabetes + smoking state + systolic blood pressure + diastolic blood pressure + BMI + physical activity (categorized) + waist circumference + coronary artery calcification

1. Participants with total cholesterol ≤ 200 mg/dl

Time to first CVD-Event ~ SNP + Age + Sex + HDL cholesterol + LDL cholesterol + triglycerides + diabetes + smoking state + systolic blood pressure + diastolic blood pressure + CRP + BMI + physical activity (categorized) + waist circumference + coronary artery calcification

1. Participants with total cholesterol > 200 mg/dl

Time to first CVD-Event ~ SNP + Age + Sex + HDL cholesterol + LDL cholesterol + triglycerides + diabetes + smoking state + systolic blood pressure + diastolic blood pressure + CRP + BMI + physical activity (categorized) + waist circumference + coronary artery calcification

1. Participants with LDL cholesterol ≤ 115 mg/dl

Time to first CVD-Event ~ SNP + Age + Sex + total cholesterol + HDL cholesterol + triglycerides + diabetes + smoking state + systolic blood pressure + diastolic blood pressure + CRP + BMI + physical activity (categorized) + waist circumference + coronary artery calcification

1. Participants with LDL cholesterol > 115 mg/dl

Time to first CVD-Event ~ SNP + Age + Sex + total cholesterol + HDL cholesterol + triglycerides + diabetes + smoking state + systolic blood pressure + diastolic blood pressure + CRP + BMI + physical activity (categorized) + waist circumference + coronary artery calcification

1. Female participants with HDL ≤ 50 mg/dl and male pariticpants with HDL ≤ 40 mg/dl

Time to first CVD-Event ~ SNP + Age + Sex + total cholesterol + LDL cholesterol + triglycerides + diabetes + smoking state + systolic blood pressure + diastolic blood pressure + CRP + BMI + physical activity (categorized) + waist circumference + coronary artery calcification

1. Female participants with HDL > 50 mg/dl and male pariticpants with HDL > 40 mg/dl

Time to first CVD-Event ~ SNP + Age + Sex + total cholesterol + LDL cholesterol + triglycerides + diabetes + smoking state + systolic blood pressure + diastolic blood pressure + CRP + BMI + physical activity (categorized) + waist circumference + coronary artery calcification

1. Participants with triglyceride level < 150 mg/dl

Time to first CVD-Event ~ SNP + Age + Sex + total cholesterol + HDL cholesterol + LDL cholesterol + diabetes + smoking state + systolic blood pressure + diastolic blood pressure + CRP + BMI + physical activity (categorized) + waist circumference + coronary artery calcification

1. Participants with triglyceride level ≥ 150 mg/dl

Time to first CVD-Event ~ SNP + Age + Sex + total cholesterol + HDL cholesterol + LDL cholesterol + diabetes + smoking state + systolic blood pressure + diastolic blood pressure + CRP + BMI + physical activity (categorized) + waist circumference + coronary artery calcification

1. Participants with systolic blood pressure < 120 and diastolic blood pressure < 80

Time to first CVD-Event ~ SNP + Age + Sex + total cholesterol + HDL cholesterol + LDL cholesterol + triglycerides + diabetes + smoking state + systolic blood pressure + CRP + BMI + physical activity (categorized) + waist circumference + coronary artery calcification

1. Participants with systolic blood pressure ≥ 120 and < 140 and/or diastolic blood pressure ≥ 80 and < 90

Time to first CVD-Event ~ SNP + Age + Sex + total cholesterol + HDL cholesterol + LDL cholesterol + triglycerides + diabetes + smoking state + systolic blood pressure + CRP + BMI + physical activity (categorized) + waist circumference + coronary artery calcification

1. Participants with systolic blood pressure ≥ 140 and diastolic blood pressure ≥ 90

Time to first CVD-Event ~ SNP + Age + Sex + total cholesterol + HDL cholesterol + LDL cholesterol + triglycerides + diabetes + smoking state + systolic blood pressure + CRP + BMI + physical activity (categorized) + waist circumference + coronary artery calcification

1. Participants without diabetes

Time to first CVD-Event ~ SNP + Age + Sex + total cholesterol + HDL cholesterol + LDL cholesterol + triglycerides + smoking state + systolic blood pressure + diastolic blood pressure + CRP + BMI + physical activity (categorized) + waist circumference + coronary artery calcification

1. Participants with diabetes

Time to first CVD-Event ~ SNP + Age + Sex + total cholesterol + HDL cholesterol + LDL cholesterol + triglycerides + smoking state + systolic blood pressure + diastolic blood pressure + CRP + BMI + physical activity (categorized) + waist circumference + coronary artery calcification

1. Former smoker

Time to first CVD-Event ~ SNP + Age + Sex + total cholesterol + HDL cholesterol + LDL cholesterol + triglycerides + diabetes + systolic blood pressure + diastolic blood pressure + CRP + BMI + physical activity (categorized) + waist circumference + coronary artery calcification

1. Current smoker

Time to first CVD-Event ~ SNP + Age + Sex + total cholesterol + HDL cholesterol + LDL cholesterol + triglycerides + diabetes + systolic blood pressure + diastolic blood pressure + CRP + BMI + physical activity (categorized) + waist circumference + coronary artery calcification

1. Never smoker

Time to first CVD-Event ~ SNP + Age + Sex + total cholesterol + HDL cholesterol + LDL cholesterol + triglycerides + diabetes + systolic blood pressure + diastolic blood pressure + CRP + BMI + physical activity (categorized) + waist circumference + coronary artery calcification

1. Female participants with waist circumference ≤ 80 cm and male participants with waist circumference ≤ 94 cm

Time to first CVD-Event ~ SNP + Age + Sex + total cholesterol + HDL cholesterol + LDL cholesterol + triglycerides + diabetes + smoking state + systolic blood pressure + diastolic blood pressure + CRP + BMI + physical activity (categorized) + coronary artery calcification

1. Female participants with waist circumference ≤ 80 cm and male participants with waist circumference ≤ 94 cm

Time to first CVD-Event ~ SNP + Age + Sex + total cholesterol + HDL cholesterol + LDL cholesterol + triglycerides + diabetes + smoking state + systolic blood pressure + diastolic blood pressure + CRP + BMI + physical activity (categorized) + coronary artery calcification

1. Participants with BMI < 25 kg/m²

Time to first CVD-Event ~ SNP + Age + Sex + total cholesterol + HDL cholesterol + LDL cholesterol + triglycerides + diabetes + smoking state + systolic blood pressure + diastolic blood pressure + CRP + physical activity (categorized) + waist circumference + coronary artery calcification

1. Participants with BMI ≥ 25 kg/m²

Time to first CVD-Event ~ SNP + Age + Sex + total cholesterol + HDL cholesterol + LDL cholesterol + triglycerides + diabetes + smoking state + systolic blood pressure + diastolic blood pressure + CRP + physical activity (categorized) + waist circumference + coronary artery calcification

**GRS**

- 1. All study participants

GRS Crude

Time to first CVD-Event ~ GRS

GRS Adjusted

Time to first CVD-Event ~ GRS + Age + Sex + total cholesterol + HDL cholesterol + LDL cholesterol + triglycerides + diabetes + smoking state + systolic blood pressure + diastolic blood pressure + CRP + BMI + physical activity (categorized) + waist circumference + coronary artery calcification

1. GRS Female participants younger than 65 years and male participants younger than 55 years

Time to first CVD-Event ~ GRS + Sex + total cholesterol + HDL cholesterol + LDL cholesterol + triglycerides + diabetes + smoking state + systolic blood pressure + diastolic blood pressure + CRP + BMI + physical activity (categorized) + waist circumference + coronary artery calcification

1. Female participant older or equal 65 years and male participants older or equal 55 years

Time to first CVD-Event ~ GRS + Sex + total cholesterol + HDL cholesterol + LDL cholesterol + triglycerides + diabetes + smoking state + systolic blood pressure + diastolic blood pressure + CRP + BMI + physical activity (categorized) + waist circumference + coronary artery calcification

1. GRS Male participants

Time to first CVD-Event ~ GRS + Age + total cholesterol + HDL cholesterol + LDL cholesterol + triglycerides + diabetes + smoking state + systolic blood pressure + diastolic blood pressure + CRP + BMI + physical activity (categorized) + waist circumference + coronary artery calcification

1. GRS Female participants

Time to first CVD-Event ~ GRS + Age + total cholesterol + HDL cholesterol + LDL cholesterol + triglycerides + diabetes + smoking state + systolic blood pressure + diastolic blood pressure + CRP + BMI + physical activity (categorized) + waist circumference + coronary artery calcification

1. GRS Participants with high sensitivity CRP ≤ 0.3 mg/dl

Time to first CVD-Event ~ GRS + Age + Sex + total cholesterol + HDL cholesterol + LDL cholesterol + triglycerides + diabetes + smoking state + systolic blood pressure + diastolic blood pressure + BMI + physical activity (categorized) + waist circumference + coronary artery calcification

1. GRS Participants with high sensitivity CRP > 0.3 mg/dl but ≤ 0.5 mg/dl

Time to first CVD-Event ~ GRS + Age + Sex + total cholesterol + HDL cholesterol + LDL cholesterol + triglycerides + diabetes + smoking state + systolic blood pressure + diastolic blood pressure + BMI + physical activity (categorized) + waist circumference + coronary artery calcification

1. GRS Participants with high sensitivity CRP > 0.5 mg/dl

Time to first CVD-Event ~ GRS + Age + Sex + total cholesterol + HDL cholesterol + LDL cholesterol + triglycerides + diabetes + smoking state + systolic blood pressure + diastolic blood pressure + BMI + physical activity (categorized) + waist circumference + coronary artery calcification

1. GRS Participants with total cholesterol ≤ 200 mg/dl

Time to first CVD-Event ~ GRS + Age + Sex + HDL cholesterol + LDL cholesterol + triglycerides + diabetes + smoking state + systolic blood pressure + diastolic blood pressure + CRP + BMI + physical activity (categorized) + waist circumference + coronary artery calcification

1. GRS Participants with total cholesterol > 200 mg/dl

Time to first CVD-Event ~ GRS + Age + Sex + HDL cholesterol + LDL cholesterol + triglycerides + diabetes + smoking state + systolic blood pressure + diastolic blood pressure + CRP + BMI + physical activity (categorized) + waist circumference + coronary artery calcification

1. GRS Participants with LDL cholesterol ≤ 115 mg/dl

Time to first CVD-Event ~ GRS + Age + Sex + total cholesterol + HDL cholesterol + triglycerides + diabetes + smoking state + systolic blood pressure + diastolic blood pressure + CRP + BMI + physical activity (categorized) + waist circumference + coronary artery calcification

1. GRS Participants with LDL cholesterol > 115 mg/dl

Time to first CVD-Event ~ GRS + Age + Sex + total cholesterol + HDL cholesterol + triglycerides + diabetes + smoking state + systolic blood pressure + diastolic blood pressure + CRP + BMI + physical activity (categorized) + waist circumference + coronary artery calcification

1. GRS Female participants with HDL ≤ 50 mg/dl and male pariticpants with HDL ≤ 40 mg/dl

Time to first CVD-Event ~ GRS + Age + Sex + total cholesterol + LDL cholesterol + triglycerides + diabetes + smoking state + systolic blood pressure + diastolic blood pressure + CRP + BMI + physical activity (categorized) + waist circumference + coronary artery calcification

1. GRS Female participants with HDL > 50 mg/dl and male pariticpants with HDL > 40 mg/dl

Time to first CVD-Event ~ GRS + Age + Sex + total cholesterol + LDL cholesterol + triglycerides + diabetes + smoking state + systolic blood pressure + diastolic blood pressure + CRP + BMI + physical activity (categorized) + waist circumference + coronary artery calcification

1. GRS Participants wiht triglyceride level < 150 mg/dl

Time to first CVD-Event ~ GRS + Age + Sex + total cholesterol + HDL cholesterol + LDL cholesterol + diabetes + smoking state + systolic blood pressure + diastolic blood pressure + CRP + BMI + physical activity (categorized) + waist circumference + coronary artery calcification

1. GRS Participants wiht triglyceride level ≥ 150 mg/dl

Time to first CVD-Event ~ GRS + Age + Sex + total cholesterol + HDL cholesterol + LDL cholesterol + diabetes + smoking state + systolic blood pressure + diastolic blood pressure + CRP + BMI + physical activity (categorized) + waist circumference + coronary artery calcification

1. GRS Participants with systolic blood pressure < 120 and diastolic blood pressure < 80

Time to first CVD-Event ~ GRS + Age + Sex + total cholesterol + HDL cholesterol + LDL cholesterol + triglycerides + diabetes + smoking state + systolic blood pressure + CRP + BMI + physical activity (categorized) + waist circumference + coronary artery calcification

1. GRS Participants with systolic blood pressure ≥ 120 and < 140 and/or diastolic blood pressure ≥ 80 and < 90

Time to first CVD-Event ~ GRS + Age + Sex + total cholesterol + HDL cholesterol + LDL cholesterol + triglycerides + diabetes + smoking state + systolic blood pressure + CRP + BMI + physical activity (categorized) + waist circumference + coronary artery calcification

1. GRS Participants with systolic blood pressure ≥ 140 and diastolic blood pressure ≥ 90

Time to first CVD-Event ~ GRS + Age + Sex + total cholesterol + HDL cholesterol + LDL cholesterol + triglycerides + diabetes + smoking state + systolic blood pressure + CRP + BMI + physical activity (categorized) + waist circumference + coronary artery calcification

1. GRS Participants without diabetes

Time to first CVD-Event ~ GRS + Age + Sex + total cholesterol + HDL cholesterol + LDL cholesterol + triglycerides + smoking state + systolic blood pressure + diastolic blood pressure + CRP + BMI + physical activity (categorized) + waist circumference + coronary artery calcification

1. GRS Participants with diabetes

Time to first CVD-Event ~ GRS + Age + Sex + total cholesterol + HDL cholesterol + LDL cholesterol + triglycerides + smoking state + systolic blood pressure + diastolic blood pressure + CRP + BMI + physical activity (categorized) + waist circumference + coronary artery calcification

1. GRS Former smoker

Time to first CVD-Event ~ GRS + Age + Sex + total cholesterol + HDL cholesterol + LDL cholesterol + triglycerides + diabetes + systolic blood pressure + diastolic blood pressure + CRP + BMI + physical activity (categorized) + waist circumference + coronary artery calcification

1. GRS Current smoker

Time to first CVD-Event ~ GRS + Age + Sex + total cholesterol + HDL cholesterol + LDL cholesterol + triglycerides + diabetes + systolic blood pressure + diastolic blood pressure + CRP + BMI + physical activity (categorized) + waist circumference + coronary artery calcification

1. GRS Never smoker

Time to first CVD-Event ~ GRS + Age + Sex + total cholesterol + HDL cholesterol + LDL cholesterol + triglycerides + diabetes + systolic blood pressure + diastolic blood pressure + CRP + BMI + physical activity (categorized) + waist circumference + coronary artery calcification

1. GRS Female participants with waist circumference ≤ 80 cm and male participants with waist circumference ≤ 94 cm

Time to first CVD-Event ~ GRS + Age + Sex + total cholesterol + HDL cholesterol + LDL cholesterol + triglycerides + diabetes + smoking state + systolic blood pressure + diastolic blood pressure + CRP + BMI + physical activity (categorized) + coronary artery calcification

1. GRS Female participants with waist circumference ≤ 80 cm and male participants with waist circumference ≤ 94 cm

Time to first CVD-Event ~ GRS + Age + Sex + total cholesterol + HDL cholesterol + LDL cholesterol + triglycerides + diabetes + smoking state + systolic blood pressure + diastolic blood pressure + CRP + BMI + physical activity (categorized) + coronary artery calcification

1. GRS Participants with BMI < 25 kg/m²

Time to first CVD-Event ~ GRS + Age + Sex + total cholesterol + HDL cholesterol + LDL cholesterol + triglycerides + diabetes + smoking state + systolic blood pressure + diastolic blood pressure + CRP + physical activity (categorized) + waist circumference + coronary artery calcification

1. GRS Participants with BMI ≥ 25 kg/m²

Time to first CVD-Event ~ GRS + Age + Sex + total cholesterol + HDL cholesterol + LDL cholesterol + triglycerides + diabetes + smoking state + systolic blood pressure + diastolic blood pressure + CRP + physical activity (categorized) + waist circumference + coronary artery calcification
